# Consequences of Lineage-Specific Gene Loss on Functional Evolution of Surviving Paralogs: ALDH1A and Retinoic Acid Signaling in Vertebrate Genomes

**DOI:** 10.1371/journal.pgen.1000496

**Published:** 2009-05-29

**Authors:** Cristian Cañestro, Julian M. Catchen, Adriana Rodríguez-Marí, Hayato Yokoi, John H. Postlethwait

**Affiliations:** Institute of Neuroscience, University of Oregon, Eugene, Oregon, United States of America; National Institute of Genetics, Japan

## Abstract

Genome duplications increase genetic diversity and may facilitate the evolution of gene subfunctions. Little attention, however, has focused on the evolutionary impact of lineage-specific gene loss. Here, we show that identifying lineage-specific gene loss after genome duplication is important for understanding the evolution of gene subfunctions in surviving paralogs and for improving functional connectivity among human and model organism genomes. We examine the general principles of gene loss following duplication, coupled with expression analysis of the retinaldehyde dehydrogenase *Aldh1a* gene family during retinoic acid signaling in eye development as a case study. Humans have three *ALDH1A* genes, but teleosts have just one or two. We used comparative genomics and conserved syntenies to identify loss of ohnologs (paralogs derived from genome duplication) and to clarify uncertain phylogenies. Analysis showed that *Aldh1a1* and *Aldh1a2* form a clade that is sister to *Aldh1a3*-related genes. Genome comparisons showed secondarily loss of *aldh1a1* in teleosts, revealing that *Aldh1a1* is not a tetrapod innovation and that *aldh1a3* was recently lost in medaka, making it the first known vertebrate with a single *aldh1a* gene. Interestingly, results revealed asymmetric distribution of surviving ohnologs between co-orthologous teleost chromosome segments, suggesting that local genome architecture can influence ohnolog survival. We propose a model that reconstructs the chromosomal history of the *Aldh1a* family in the ancestral vertebrate genome, coupled with the evolution of gene functions in surviving *Aldh1a* ohnologs after R1, R2, and R3 genome duplications. Results provide evidence for early subfunctionalization and late subfunction-partitioning and suggest a mechanistic model based on altered regulation leading to heterochronic gene expression to explain the acquisition or modification of subfunctions by surviving ohnologs that preserve unaltered ancestral developmental programs in the face of gene loss.

## Author Summary

Gene duplication may facilitate the acquisition of genetic diversity. Little is known, however, about the impact of gene loss on the functions of surviving genes. When a gene is lost, can other closely related genes evolve to perform the functions of the lost gene? Answering this question can be difficult because the proof for gene loss is based on negative evidence and thus can easily pass unnoticed. Here, we illustrate how the comparison of genomic neighborhoods in different species can help reconstruct the chromosomal history of a gene family and provide robust evidence for gene loss, even without an appropriate early-diverging comparator group. Identifying gene loss is important because it helps distinguish between gene gain as a lineage-specific innovation and gene loss as a lineage-specific simplification. As a case study, we investigated the expression of the *Aldh1a* family, which is crucial for retinoic acid signaling in development of eyes, limbs, the brain, and in cancer. Results showed that gene loss is indeed associated with the evolution of functional change in surviving gene family members. Our results highlight the relevance of comparative genomics for identifying gene loss and improving the functional connectivity among human and model organism genomes.

## Introduction

Understanding the evolution of gene functions during vertebrate evolution is important for the proper interpretation of comparative analyses, especially when using model organisms to understand human gene functions. Gene duplication has been proposed to facilitate the evolution of gene functions [1], and the mechanisms of neofunctionalization and subfunctionalization may play a role [1]–[3] (reviewed in [4]). Human gene families show the signatures of two rounds of whole genome duplication (R1 and R2) that occurred during early vertebrate evolution [1], [5]–[14] (but see [15]). Mutations in gene copies that arose in these R1 and R2 events often cause related diseases (for example, osteogenesis imperfecta (*COL1A1*) and spondyloepiphyseal dysplasia (*COL2A1*), bullous erythroderma ichthyosiformis (*KRT1*) and epidermolysis bullosa (*KRT5*), and syndactyly type II (*HOXD13*) and hand-foot-uterus syndrome (*HOXA13*)). Comparative analysis shows that fish genomes have two co-orthologs for many human genes as a result of a third round of genome duplication (R3) that occurred at the base of the teleost radiation [16]–[28]. Early on, S. Ohno [1] recognized the relevance of increased genetic diversity after genome duplication, and in his honor, gene duplicates originated by genome duplication are called “ohnologs” [29]. This term is useful because of the special properties that ohnologs possess at their birth compared to duplications that arise by other mechanisms such as unequal crossing-over, tandem gene duplication, or retrotransposition.

While many studies focus on how gene duplications can facilitate the acquisition of evolutionary innovations during vertebrate evolution, less attention has been focused on the evolutionary impact of lineage-specific gene losses. Differential ohnolog loss is important because it decreases genetic diversity within a species but increases genetic diversity between species. Loss of one copy of a pair of fully redundant gene duplicates should not usually have significant impact, but duplicate loss after functional divergence can have evolutionary consequences. Reciprocal paralog loss in different lineages can affect a species' biology, decrease evolvability, and diminish adaptability to changing environments [30]–[34]. In other cases, gene loss can be adaptive, and thus relevant for a species' evolution, perhaps even for human origins [35]. Furthermore, reciprocal loss of even fully redundant gene duplicates in two populations may contribute to speciation [33],[36].

Global estimations of gene loss in fully sequenced vertebrate genomes have been inferred by massive phylogenetic reconstructions of gene families [37]–[39]. Large-scale analyses, however, are sensitive to uncertainties of phylogenetic analysis, for example, asymmetric rates of evolution among paralogs can affect tree topologies and generate gene phylogenies that are not congruent with the species phylogenies of which they are a part [40],[41]. Furthermore, published genome-wide studies have not addressed gene function. In principle, gene functions that are associated exclusively with a certain gene may disappear if the gene is lost. It is possible, however, that exclusive gene functions might not disappear in situations in which a surviving paralog might acquire or maintain the expression domain of the lost paralog, and thereby the ancestral developmental or physiological program can remain unaltered [42],[43]. Because the evidence for gene loss is negative and can pass unnoticed and is subject to uncertainties in the completion or assembly of sequenced genomes and in copy number polymorphisms [44],[45], the impact of gene loss in the evolution of function of surviving paralogs is under-investigated. Identification of gene loss is especially important to avoid misinterpretations when human gene functions are inferred from the study of model organisms that might have suffered lineage-specific paralog loss, so that the model has no true ortholog of the phylogenetically most closely related human gene, or vice versa.

To evaluate the evolutionary relevance of gene loss on the functions of surviving paralogs, it is first important to understand gene phylogeny. For genes lost following large-scale genome duplications, conserved syntenies can identify duplicated genomic regions and provide evidence for gene loss, often even in situations lacking a proper outgroup [46]. Genes lost after genome duplication events have been called “ohnologs gone missing” (ogm), and their identification is important to properly distinguish orthologs from other types of paralogs [32],[47],[48]. We propose that identification of gene loss by automated comparative genomic analysis of conserved syntenies can: (1) help resolve uncertain gene phylogenies; (2) help discriminate cases of evolutionary innovations from evolutionary simplifications; (3) facilitate understanding of the diversification of gene functions among species; and, importantly, (4) improve functional connectivity of human and model organism genomes.

To explore the roles of gene loss in a functional context, work reported here focuses on the vertebrate *Aldh1a* retinaldehyde dehydrogenase gene family (formerly known as *Raldh*) as a case study. Understanding the evolution of *Aldh1a* genes is important because this family encodes enzymes responsible for the synthesis of retinoic acid (RA), the active derivative of vitamin A (retinol). In humans, as in other vertebrates, RA plays important roles during embryogenesis, for example, in axial patterning, limb development, and differentiation of eyes and nervous system, as well as functioning in adult organ homeostasis (recently reviewed in [49],[50]). Alterations of RA metabolism can lead to human pathologies including breast and prostate cancers, osteoporosis, rheumatoid arthritis, dermatologic diseases, developmental anomalies and premature births.

The evolutionary origin of the *Aldh1a* family probably predates the origin of stem bilaterians [51],[52], but the ability of the Aldh1a enzyme of basally diverging bilaterians to synthesize RA remains unknown. *Aldh1a* likely arose by duplication of an ancestral gene related to the *Aldh2* gene family, which encodes a mitochondrial Aldh that plays a major role in acetaldehyde oxidation and is broadly represented in most extant organisms from bacteria to humans [53]. Humans and many other vertebrates have three genes that encode Aldh1a family enzymes: *ALDH1A1*, *ALDH1A2* and *ALDH1A3* [54]. Studies of model organisms such as mouse, chicken, frog and zebrafish have provided insights into the roles of each *Aldh1a* gene in the synthesis of RA (reviewed in [49], [50], [55]–[58]). Variation in *Aldh1a* gene number in different animal lineages has been hypothesized to be relevant to animal evolution due to potential effects of RA metabolism on the mechanisms of development [59]–[61]; reviewed in [62].

Rodents have a fourth *Aldh1a* paralog that is mostly expressed in kidney (termed, *Aldh1a4* in rat [63], and its ortholog *Aldh1a7* in mouse [64]); these genes originated by a tandem gene duplication in the rodent lineage after it diverged from the human lineage. Experiments using a heterologous *Xenopus* system to express mouse *Aldh1a7* suggested that Aldh1a7 might not be involved in RA synthesis [64]. In contrast to rodents with four *Aldh1* genes, most teleost fish have just two, *aldh1a2* and *aldh1a3*, but they lack *aldh1a1* [59],[65]. Phylogenetic relationships of vertebrate *Aldh1a1* genes are still controversial, and whether *Aldh1a1* is a tetrapod innovation or its absence from teleosts is due to gene loss is still unknown. Furthermore, the functional consequences of these gene copy number variations have not yet been investigated.

Here, we show how comprehensive comparative genomic analyses of syntenic conservation provides a framework necessary for the examination of the general mechanisms by which lineage-specific gene loss can impact the functions of surviving paralogs. This work reveals multiple losses of *Aldh1a* ohnologs and proposes an evolutionary genomic model that reconstructs the history of *Aldh1a*-related vertebrate chromosomes and the evolution of *Aldh1a* gene functions during and subsequent to the R1, R2, and R3 genome duplications. Results show that acquisition or modification of expression domains by surviving paralogs may lead to lineage-specific innovations that preserve unaltered ancestral developmental programs in the face of gene loss. This work highlights the importance of comparative genomics for understanding the historical basis of gene loss, and to improve functional connectivity between model organism and human genomes.

## Results

### Phylogenetic Analysis of the Vertebrate *ALDH1A* Gene Family

**Figure 1:**
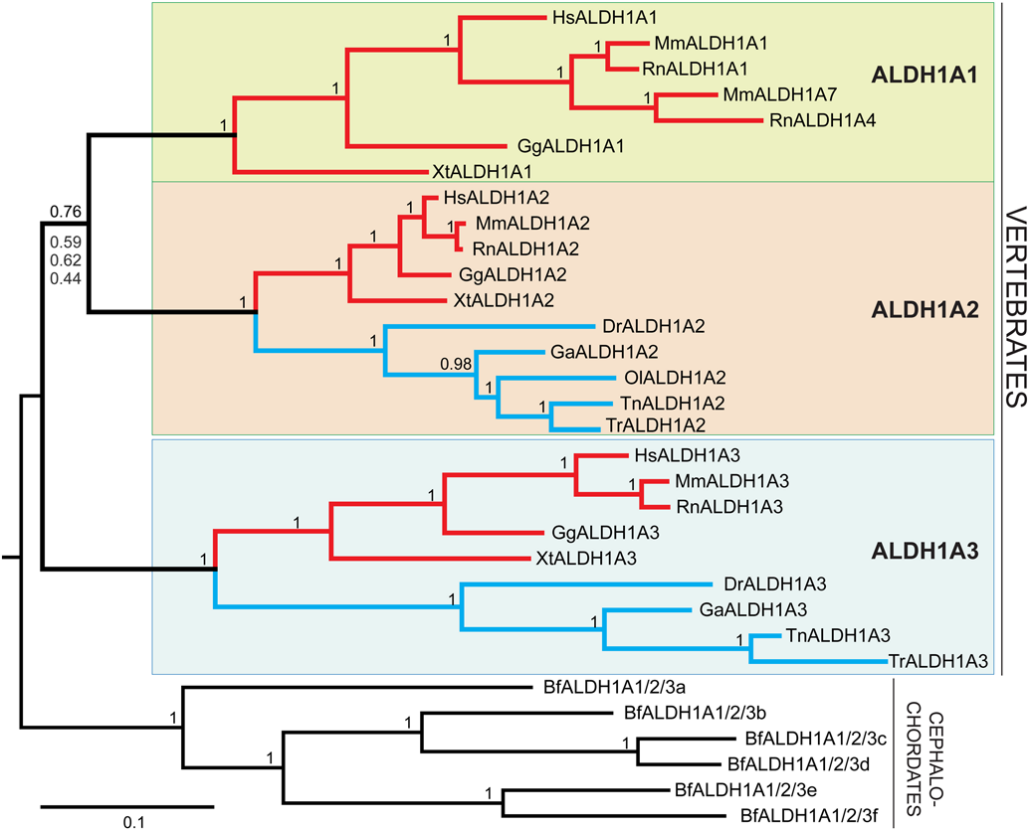
Phylogenetic tree of the vertebrate Aldh1A gene family. All phylogenetic methodologies (Bayesian, Maximum-likelihood, Neighbor-joining and Maximum-parsimony; included in Figure S1) agreed on a unique gene topology in which Aldh1a1 (green background) and Aldh1a2 (tan background) are the closest sister clades, while Aldh1a3 (blue background) diverged basally: ((Aldh1a1, Aldh1a2), Aldh1a3). Values at nodes correspond to the posterior probabilities inferred from the Bayesian method and generally show a highly supported tree topology. The only exception is a moderately high value of 0.76 for the Aldh1a1-Aldh1a2 node (for this node, the ML, NJ and MP supporting values are also shown). While Aldh1a2 and Aldh1a3 are present in both tetrapods (red lines) and teleosts (blue lines), Aldh1a1 is absent from teleost genomes. Scale bar indicates amino-acid substitutions. Tetrapods: Hs, *Homo sapiens*; Mm, *Mus musculus*; Rn, *Rattus novergicus*; Gg, *Gallus gallus*; Xt, *Xenopus tropicalis*; Teleosts: Dr, *Danio rerio*; Ga, *Gasterosteus aculeatus*; Ol, *Oryzias latipes*; Tn, *Tetraodon nigroviridis*; Tr, *Takifugu rubripes*; Cephalochordates: Bf, *Branchiostoma floridae*.

To understand the history of gene gain and loss in the *Aldh1a* family, it is important to first understand the phylogeny of family members. Unfortunately, evolutionary relationships among vertebrate Aldh1a paralogs are currently unclear. In one analysis, the three vertebrate Aldh1a clades collapsed to an unresolved trichotomy [59], and in another, Aldh1a2 and Aldh1a3 appeared as sister groups (Aldh1a1, (Aldh1a2, Aldh1a3)), supported by low bootstrap values [65]. These problems may stem from sequence similarities among the Aldh1a1, Aldh1a2 and Aldh1a3 proteins and the use of the evolutionarily distant mitochondrial Aldh2 family to root the tree. To overcome this uncertainty, we turned to a chordate outgroup, the cephalochordate amphioxus, whose lineage diverged from that of the vertebrates before the R1 and R2 events [66],[67]. Amphioxus has both *Aldh1a* and *Aldh2* gene families [59], and hence its *Aldh1a* genes are much more closely related to vertebrate *Aldh1a1* genes than is the *Aldh2* gene family. We found that several different phylogenetic methodologies, including Bayesian inference, Maximum-likelihood, gamma-corrected Neighbor-Joining and Maximum-Parsimony all agreed on the same tree topology ((Aldh1a1, Aldh1a2), Aldh1a3)), with Aldh1a1 and Aldh1a2 as sister groups (Figures 1 and S1). This phylogeny differs from both published results: the trichotomy result and the view of Aldh1a2 and Aldh1a3 as sister clades [59],[65]. Our results still provided only a moderately high probability of 0.76 supporting the Aldh1a1/2 clade under the Bayesian phylogenetic inference (Figure 1); thus, phylogenetic analysis alone is insufficient to definitively resolve Aldh1a relationships. To further test historical relationships among Aldh1a paralogs, we examined a data set independent of *Aldh1a* gene sequence by conducting comparative genomic analyses of the entire genomic neighborhoods (GN) surrounding *Aldh1a* genes in the genomes of humans and other vertebrates.

### Analysis of Conserved Syntenies for the Human *ALDH1A* Gene Family

The results of our phylogenetic analysis ((Aldh1a1, Aldh1a2), Aldh1a3) (Figure 1) implies that the duplication event that gave rise to *Aldh1a1* and *Aldh1a2* was more recent than the duplication event that gave rise to *Aldh1a3* and the ancestral *Aldh1a1/2* gene. If the duplication events that produced the Aldh1a family involved whole genomes or large chromosomal segments, then the phylogenic hypothesis of relationships (Figure 1) predicts more syntenic conservation between the genomic neighborhoods (GN) surrounding *Aldh1a1* and *Aldh1a2* than between the genomic neighborhood of *Aldh1a3* and either *Aldh1a1* or *Aldh1a2*. To test this hypothesis, we conducted a comparative genomic analysis of conserved synteny among the genomic neighborhoods of each *ALDH1A* paralog in the human genome.

**Figure 2:**
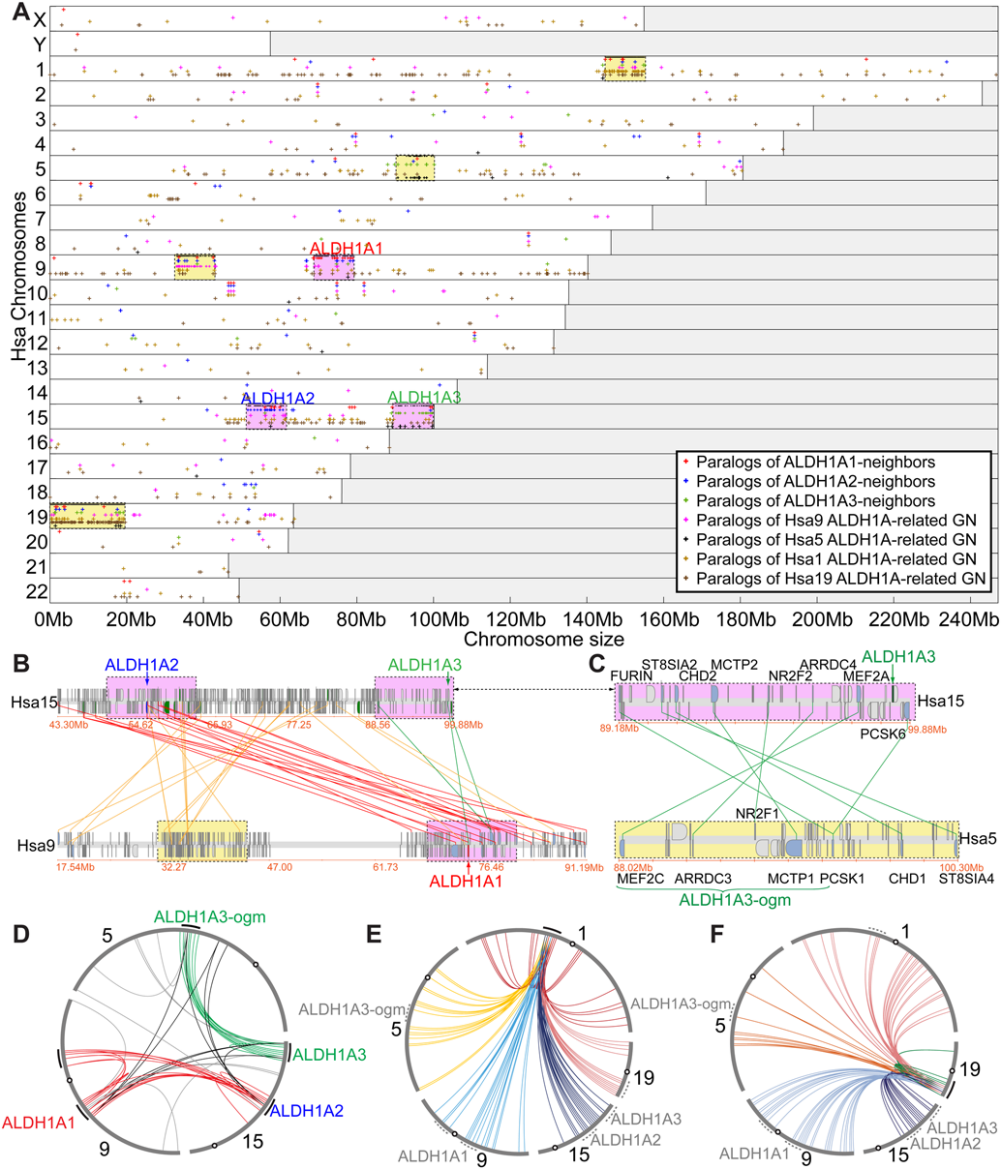
Paralogous syntenic conservation among *ALDH1A* genomic neighborhoods (GN) in the human genome. (A) Composite dotplot representing the distribution of paralogs of genes (black dots) within a 10 Mb-window surrounding each member of the *ALDH1A* family throughout the human genome (red crosses: *ALDH1A*-neighbor paralogs; dark blue crosses: *ALDH1A2*-neighbor paralogs, and light-green crosses: *ALDH1A3*-neighbor paralogs. The genomes of *Ciona intestinalis* and *Branchiostoma floridae*, which represent urochordates and cephalochordates, respectively, the two closest vertebrate relatives [66],[110], were used as outgroups to define paralogy groups in the human genome. Gene accession numbers and genomic information for each group of paralogy represented in the dotplot is provided in Table S1. Human chromosomes are represented in the y-axis, and drawn to scale in the x-axis with the p-terminus of each chromosome at the left and the q-terminus at the right of each white row. Chromosomal regions that appear enriched in *Aldh1a*-neighbor paralogs are indicated with colored boxes, highlighted in pink if *ALDH1A* genes are present, and in yellow if no *ALDH1A* genes are present. The distribution of paralogs of genes located in the yellow boxes is also represented in the dotplot (golden crosses: Hsa1 *ALDH1A*-related GN; black crosses: Hsa5 *ALDH1A*-related GN; pink crosses: Hsa9 *ALDH1A*-related GN; brown crosses: Hsa15 *ALDH1A*-related GN). (B) Two clusters of genes in Hsa15 and Hsa9 display a substantial number of conserved syntenies between the *ALDH1A1* and *ALDH1A2* gene neighborhoods (red lines), but fewer conserved syntenies with the *ALDH1A3* GN (green lines), supporting the idea that *ALDH1A1* and *ALDH1A2* are the closest sister paralogs, consistent with the phylogenetic tree ((ALDH1A1, ALDH1A2), ALDH1A3) in Figure 1. Golden lines show conserved synteny between Hsa15 and parts of Hsa9 probably due to a local transposition that moved material between *ALDH1A2* and *ALDH1A3* from its original location to the right of *ALDH1A1* to the left of *ALDH1A1* (or vice versa). Colored boxes correspond to regions shown in A. Figure S2 provides high-resolution images including the name of conserved syntenic genes. (C) Representation of a pair of paralogous gene clusters in Hsa15 and Hsa5 displaying high amounts of conserved synteny between the *ALDH1A3* and *ALDH1A3*-ogm GNs (green lines). (D–F) Circleplots display the patterns of conserved synteny between the *ALDH1A* GN (labeled with black arcs outside of each chromosome) revealed by the dotplot in A for Hsa1, Hsa5, Hsa9, Hsa15 and Hsa19 (see main text for explanations). While the patterns of conserved synteny between *ALDH1A1* and *ALDH1A2* GNs (red lines) and between *ALDH1A3* and *ALDH1A3*-ogm GNs (green lines) are restricted to defined dense bundles (D), lines originating from Hsa1 (E) and Hsa19 (F) are not restricted to any particular *ALDH1A* GN (the different colors of the lines in E and F label various chromosomes). Circles represent chromosome centromeres, and dotted arcs label the approximate *ALDH1A* GN positions in the chromosomes.

The three human *ALDH1A* genes are located on two chromosomes: *ALDH1A1* is on Hsa9 (human chromosome 9), while *ALDH1A2* and *ALDH1A3* are on Hsa15 separated by 43 megabases (Mb). We first made a composite dotplot to represent the genome-wide distribution of the paralogs of all genes within a 10 Mb-window surrounding each human *ALDH1A* gene throughout the 23 human chromosomes (y-axis) (we refer to this set of genes as *ALDH1A*-neighbor paralogs (red, blue and green crosses in Figure 2A)). Table S1 lists gene names, reference numbers, genomic positions and outgroup (i.e. *Branchiostoma floridae* and *Ciona intestinalis*) gene information used to construct each paralogy group in the dotplot. This plot showed that while some *ALDH1A*-neighbor paralogs appeared randomly scattered throughout the genome, some chromosomal regions contained a concentration of *ALDH1A*-neighbor paralogs (yellow and pink boxes in Figure 2A). These chromosome regions with syntenic conservation to *ALDH1A*-neighbor paralogs likely represent chromosome fragments that were duplicated during the whole genome duplication events R1 and R2 and are historically related to the expansion of the Aldh1a family. The presence of *ALDH1A*-neighbor genes conserved among *ALDH1A* genomic neighborhoods (pink-shaded dotted boxes) suggests that the *ALDH1A* family expanded by large-scale genome duplications rather than by local tandem gene duplications. The dotplot analysis also identified four genomic regions that share syntenic conservation with *ALDH1A* genomic neighborhoods, but do not contain *ALDH1A* genes (yellow-shaded boxes on Hsa1, Hsa5, Hsa9 and Hsa19). The paralogs of each gene contained in these four yellow boxes were also included in the dotplot (Figure 2A: golden, black, pink and brown crosses). In principle, the existence of the yellow-boxed regions that lack *ALDH1A* paralogs but show syntenic conservation with the *ALDH1A* genomic neighborhood could be explained by genome duplications followed by a loss of the *ALDH1A* paralog (i.e. *ALDH1A* ohnologs gone missing), or alternatively by the translocation of a portion of the genomic neighborhood away from the *ALDH1A* gene itself.

#### Syntenic analysis supports the hypothesis that *ALDH1A1* and *ALDH1A2* are the closest sister paralogs, and suggests an *ALDH1A3* ohnolog gone missing

The dotplot (Figure 2A) revealed a region about 36 Mb from *ALDH1A1* on Hsa9 that contained *ALDH1A2*- and *ALDH1A1*-neighbor paralogs but had no *ALDH1A* gene (yellow in Figure 2A). The proximity of this region to *ALDH1A1* mirrored the proximity of *ALDH1A2* and *ALDH1A3*, suggesting it as a candidate region paralogous to the *ALDH1A3* genomic neighborhood. To decrease possible effects of local chromosome rearrangements (i.e. inversions, translocations and local duplications), we looked for *ALDH1A*-neighbor paralogs shared by Hsa9 and Hsa15 using the Synteny Database and a 100-gene sliding window [48] (Figure 2B). Analysis identified a cluster that clearly showed that the *ALDH1A2* and *ALDH1A1* genomic neighborhoods share more paralog conservation to each other (red lines (n = 15) in Figure 2B), than either does to *ALDH1A3* genomic neighborhood (green lines (n = 3) in Figure 2B). Paralogs shared between the *ALDH1A1* and *ALDH1A2* genomic neighborhoods include members of the *FOXB*, *ROR*, *VPS13*, *GCNT* and *TRPM* gene families. Figure S2 provides high-resolution images including the name of each conserved syntenic *ALDH1A*-neighbor in the gene clusters shown in Figure 2. This analysis provides robust evidence independent of ALDH1A sequence that ALDH1A1 and ALDH1A2 are sister clades, and supports the tree topology obtained in our phylogenetic analysis ((ALDH1A1, ALDH1A2), ALDH1A3) (Figure 1).

The small number of paralogs shared by the *ALDH1A3* genomic neighborhood and the region 36 Mb upstream of *ALDH1A1* made it unlikely that they are evolutionarily related by the R1 and R2 genomic duplication events. The dotplot in Figure 2A, however, also revealed a region on Hsa5 that lacks an *ALDH1A3* paralog but was enriched in *ALDH1A3*-neighbor paralogs (Figure 2A, green crosses in yellow-shaded box on Hsa5). This region is a candidate for a chromosome segment that is the sister of the *ALDH1A3*-containing region (pink-shaded box on Hsa15) but that subsequent to the duplication event, lost the *ALDH1A* gene copy. We refer to the hypothesized absent *ALDH1A3* paralog on Hsa5 as an *ALDH1A3* ohnolog gone missing (*ALDH1A3*-ogm) from the human genome. Neighboring genes in the Hsa5 and Hsa15 regions showed clear syntenic conservation with gene order also preserved (note that the arbitrary convention of chromosome orientation displays them as inverted) (Figure 2C). This arrangement is consistent with these regions being paralogons that both contained *ALDH1A3* paralogs before *ALDH1A3*-ogm was lost (Figure 2C). Genes including *MEF2C*, *ARRDC3*, *NR2F1*, *MCTP1* and *PCSK1* are neighbors of the *ALDH1A3*-ogm that have been preserved in Hsa5 after the genomic duplication, with their paralogs *MEF2A*, *ARRDC4*, *NR2F2*, *MCTP2*, *and* PCSK3 and PCSK6 on Hsa15 near *ALDH1A3* (Figure 2C).

To further investigate spatial relationships along chromosomes, we used circleplots connecting genes on a chromosome to their paralogs positioned on other chromosomes [23]. The circleplot of Figure 2D shows connections found among paralogs from the *ALDH1A* genomic neighborhoods on Hsa5, Hsa9 and Hsa15. This representation highlighted two well-defined dense bundles: one mostly restricted to *ALDH1A3* and *ALDH1A3*-ogm genomic neighborhoods (green lines between Hsa15 and Hsa5 in Figure 2D), and one mostly restricted to *ALDH1A2* and *ALDH1A1* genomic neighborhoods (red lines between Hsa15 and Hsa9 in Figure 2D). The presence of red lines linking the *ALDH1A1* genomic neighborhood and the 36 Mb upstream region of *ALDH1A1* in Hsa9 are likely due to local gene duplications followed by inversions within Hsa9, including duplicates of the *DNAJ*, *ZFAND* and *TLE* families. The fact that most of the Hsa15 paralogs are located between *ALDH1A2* and *ALDH1A3* supports this argument (gold lines in Figure 2B). The circleplot of Figure 2D also showed a few scattered lines that likely represent inversions of *ALDH1A*-neighbor genes to other regions (grey lines in Figure 2D). Interestingly some lines also connect the *ALDH1A1*/*ALDH1A2* and the *ALDH1A3*/*ALDH1A3*-ogm groups, (black lines in Figure 2D); these lines may connect conserved paralogs (e.g. GCNT, CCNB, FAM81 and *PCSK* paralogs) derived from genes already present in the genomic neighborhood of the original *ALDH1A*1/2/3 gene before the expansion of the ALDH1A gene family.

Besides the genomic neighborhood of the *ALDH1A3-ogm* on Hsa5, the dotplot revealed two additional chromosome regions, one on Hsa1 and one on Hsa19, that also lacked an *ALDH1A* paralog but displayed syntenic conservation with *ALDH1A* genomic neighborhoods (Figure 2A, yellow-shaded boxes in Hsa1 and Hsa19). Circleplots, however, showed that while the neighbor-paralogs of the *ADH1A1*, *ALDH1A2*, *ALDH1A3* and *ALDH1A3*-ogm genomic neighborhoods formed tight clusters (red and green bundles in Figure 2D), the pattern of conserved syntenies between the paralogous regions of Hsa1 (Figure 2E) and Hsa19 (Figure 2F) was not specifically restricted to *ALDH1A*-related genomic neighborhoods, but were broadly distributed over most of each chromosome (Hsa1, Hsa5, Hsa9, Hsa15 and Hsa19, colored arcs in Figure 2E, F). Thus, our data, in agreement with previous work [26],[68], suggest that portions of Hsa1, Hsa5, Hsa9, Hsa15 and Hsa19 form part of a paralogy group with high syntenic conservation that evolved during the R1 and R2 genome duplications. Our results suggest that *ALDH1A* genes may have been duplicated but not preserved in ancestral chromosomes leading to parts of today's Hsa1 and Hsa19.

In summary, syntenic analysis of the genomic neighborhoods of the human *ALDH1A* gene family supported the phylogenetic results of Figure 1 in showing that ALDH1A1 and ALDH1A2 are sisters, and furthermore, suggested the location of an ALDH1A ohnolog gone missing on Hsa5 (ALDH1A3-ogm). All evidence thus supports the historical relationship: ((ALDH1A1, ALDH2), (ALDH1A3, ALDH1A3-ogm)).

### Are Murine and Human *ALDH1A3* Genes Orthologs or Paralogs?

When gene functions are compared among different organisms, it is important to distinguish whether the compared genes are orthologs or paralogs. In some cases, reciprocal loss of paralogs in different organisms can lead to the misinterpretation of paralogs as orthologs. Intriguingly, while in most vertebrates *Aldh1a2* and *Aldh1a3* are on the same chromosome separated by an intervening region of few tens of megabases, in rodents *Aldh1a2* and *Aldh1a3* are on different chromosomes [63],[64]. In rats, for instance, *Aldh1a3* is on the same chromosome as *Aldh1a1* rather than being on the same chromosome as *Aldh1a2* as in human. This arrangement would be expected if the rat *Aldh1a3* gene were a paralog rather than an ortholog of human *ALDH1A3*. Phylogenetic analysis provided strong support for the conclusion that all vertebrate *Aldh1a3* genes are orthologs [59],[65],[69], but evidence for an *ALDH1A3* ohnolog gone missing from the human genome raises the possibility of reciprocal paralog loss that would have caused human and rodent Aldh1a3 genes to be paralogs rather than orthologs.

**Figure 3:**
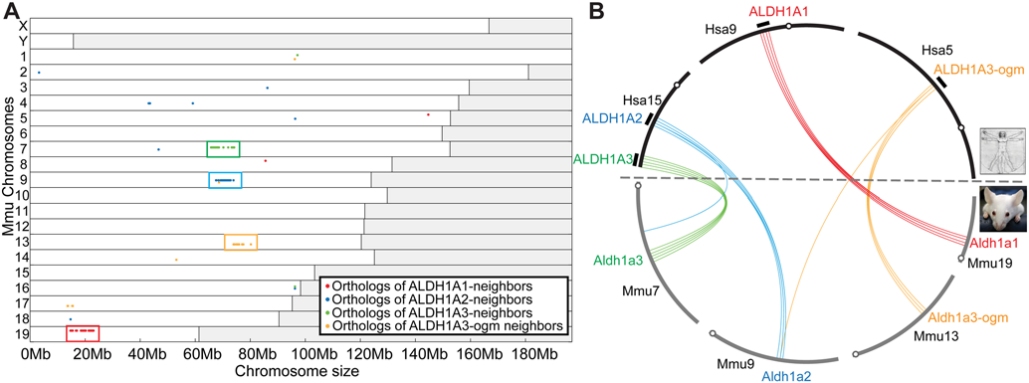
Orthologous syntenic conservation between human and mouse *ALDH1A* genomic neighborhoods. (A) Dotplot displays of the genomic distribution of the mouse orthologs (crosses) of the human *ALDH1A*-neighbor genes within a 10 Mb-window of the human *ALDH1A* gene (red: *ALDH1A1*-neighbor genes; blue: *ALDH1A2*-neighbor genes; green: *ALDH13*-neighbor genes; and yellow: *ALDH1A3-ogm*-neighbor genes), and reveals the presence of four regions (colored boxes) in Mmu7, Mmu9, Mmu13 and Mmu19 orthologous to the human *ALDH1A* genomic neighborhoods (GN). Mouse chromosomes are represented in the y-axis, and chromosome position is in the x-axis. Table S2 provides gene accession numbers and genomic information for each orthology group represented in the dotplot. (B) A circle-plot shows the pattern of syntenic correspondence between the human *ALDH1A* GNs (black arcs outside chromosomes) and the candidate mouse orthologous *ALDH1A*-related chromosomes revealed in A. Figure S2 provide high resolution images of pair-wise gene clusters identified in the orthologous syntenic database [48] showing orthologous regions of high syntenic conservation between the human and mouse *Aldh1a* GNs shown in A and B. The correspondence of *ALDH1A3* GNs in Hsa 15 and Mmu7 (green lines), and *ALDH1A3*-ogm GNs in Hsa5 and Mmu13 (golden) strongly supports the conclusion that *ALDH1A3* in human is an ortholog, not a paralog, of *Aldh1a3* in mouse. These results show that the loss of *ALDH1A3*-ogm predated the divergence of mouse and human. Circles represent chromosome centromeres.

To see whether the mouse *Aldh1a3* gene is orthologous to human *ALDH1A3* or to *ALDH1A3-ogm*, we first constructed a dotplot that displayed the distribution of the mouse orthologs of human genes within 10 Mb of *ALDH1A1*, *ALDH1A2*, *ALDH1A3* and *ALDH1A3*-ogm (Figure 3A and Table S2). The dotplot revealed that most mouse orthologs of the human *ALDH1A*-neighbor genes tightly clustered on four mouse chromosomes (Mmu7, Mmu9, Mmu13, and Mmu19) (Figure 3A). Next, we compared these four mouse chromosomes to their orthologons on human chromosomes Hsa5, Hsa9 and Hsa15 in a circleplot (Figure 3B). These analyses identified four clusters of orthology in the Synteny database [48] that unequivocally related mouse orthologs of human *ALDH1A1*, *ALDH1A2*, *ALDH1A3*, and *ALDH1A3*-ogm genome neighborhoods (Figure S2). The identification of a genomic region on Mmu13 that lacks any *Aldh1a* gene but that nevertheless possesses orthologous syntenic conservation to *ALDH1A3*-ogm genomic neighborhood on Hsa5 (golden bundle in Figure 3B) provides strong evidence that the loss of *Aldh1a3*-ogm predated the split between the lineages leading to humans and rodents, and discards the hypothesis of reciprocal paralog loss. These results conclusively rule out the hypothesis that the rodent Aldh1a3 is an ortholog of human ALDH1A3-ogm, and independently supports orthologous relationships between human and mouse Aldh1a3 genes inferred by phylogenetic methods (Figure 1).

### Evolution of the *Aldh1a* Family in Teleosts

**Figure 4:**
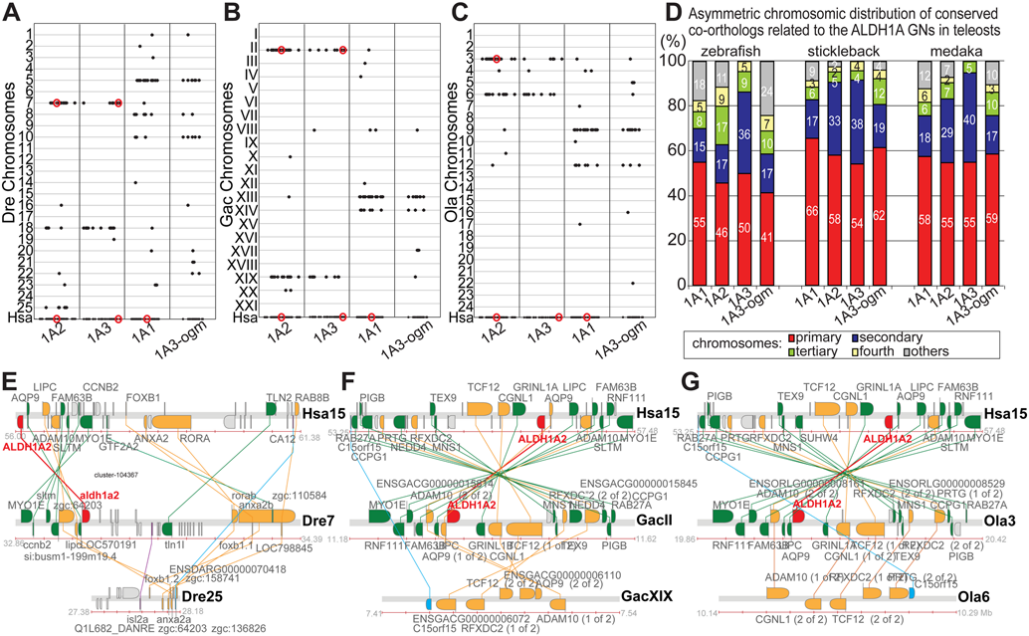
Syntenic conservation between human and fish *ALDH1A* genomic neighborhoods and asymmetric distribution of surviving fish co-orthologs. (A–C) Dotplots display the distribution of zebrafish (A), stickleback (B) and medaka (C) orthologs (red dots for Aldh1a paralogs and black crosses for neighbor genes on teleost chromosomes in y-axis) of human *ALDH1A* genes, and their neighbor genes (red dots and black dots on x-axis, respectively) within a 10 Mb-window. The dotplot reveals that all genomic neighborhoods (GN) related to the *Aldh1a* family were duplicated during R3 in teleosts, but no additional *Aldh1a*-ohnologs from R3 (e.g. *Aldh1a2′*) currently survive. Gene loci that are close to each other may appear overlapped as single crosses in the plot due to the selected graph resolution. Table S3 provides gene accession numbers and genomic information for all genes and each orthology group shown in the dotplot. (D) Bar-graph representing the asymmetric distribution of conserved co-orthologs in different chromosomes resulting from the analysis of twelve genomic neighborhoods related to the *ALDH1A* family (a1: *ALDH1A1*; a2: *Aldh1a2*; a3: *Aldh1a3*; a3-ogm: *Aldh1a3-ogm*). Values represent the percentage of conserved *ALDH1A* gene neighbor co-orthologs in each chromosome. (E–G) Co-orthologous gene clusters of zebrafish (E), stickleback (F), and medaka (G) related to the human *ALDH1A2* genomic neighborhood exemplify the asymmetric distribution of conserved co-orthologs after R3. Fish co-orthologs conserved between both fish clusters and the human cluster are colored gold, fish co-orthologs preserved only in the primary chromosome are in green, and fish co-orthologs preserved only in the secondary chromosome are in blue. *ALDH1A2* orthologs are highlighted in red, and co-orthologs present in both fish chromosomes, but absent in the human *ALDH1A2* genomic neighborhood are in purple.

Because the number of *Aldh1a* paralogs detected in genome databases is lower in teleosts than in tetrapods [59],[65], we performed a comparative genomic analysis of conserved synteny between *Aldh1a* genomic neighborhoods in the genomes of three teleosts and human to learn the historical basis of different numbers of gene family members (Figure 4).

#### Teleost *aldh1a*-ohnologs gone missing

Dotplot representations comparing the 10 Mb genomic neighborhood surrounding each human *ALDH1A* gene to the genomes of three teleosts (zebrafish (*Danio rerio*), stickleback (*Gasterosteus aculeatus*), and medaka (*Oryzias latipes*)) revealed that most fish orthologs of human *ALDH1A* neighbor genes were not randomly scattered throughout the genome, but were mostly confined to two main chromosomes in each fish species for each human *ALDH1A* genomic neighborhood (Figure 4A–C). This finding is consistent with an extra round of genome duplication (R3) that occurred in the teleost lineage after diverging from the tetrapod lineage [17]–[26]. The distribution of conserved co-orthologs between the pair of chromosomes in each teleost, however, was asymmetric. In general, one chromosome (the primary chromosome) showed considerably more syntenic conservation than the other chromosome (the secondary chromosome) (red and blue bars, respectively, in Figure 4D). Among a total of twelve chromosomal distributions of conserved orthologs related to the four human ALDH1A genomic neighborhoods analyzed in three teleost species (zebrafish, stickleback and medaka), on average 55%±7 of orthologs were in a primary chromosome, 25%±10 in a secondary chromosome, 8%±4 in a tertiary chromosome, 4%±2 in a fourth chromosome, and 8%±8 distributed throughout the rest of the genome (Figure 4D and Table S3). Fish orthologs of conserved neighbor genes surrounding human *ALDH1A2*, for instance, were mostly on a primary chromosome that also contained *aldh1a2*: in zebrafish, 46% of neighbor genes were located on chromosome Dre7; in stickleback 58% were in groupII; and in medaka 55% were on chromosome Ola3. Most of the remaining conserved synteny appeared in secondary chromosomes in which no *aldh1a* gene was present: in zebrafish 17% of genes were located on Dre18 and 17% on Dre25; in stickleback 33% were in groupXIX; and in medaka 29% were in Ola6 (Figure 4D). This asymmetric pattern of co-ortholog distribution is exemplified in the comparative genomic analysis of the human and teleost co-orthologous gene clusters with high conserved synteny related to the *ALDH1A2* genomic neighborhood (Figure 4E–G). In addition to the co-orthologs conserved in both gene clusters in fishes (shaded gold), we found that a majority of fish orthologs (shaded green) was preserved only on the primary chromosome, and just a few were preserved only on the secondary chromosome (shaded blue, Figure 4E–G).

The results of Figure 4 provide strong evidence that all *ALDH1A*-related genomic regions were duplicated in R3 during the evolution of teleosts but that each *aldh1a* gene reverted to single copy status. The fact that pairs of *aldh1a* genes in each teleost are in orthologous genomic neighborhoods, as revealed by the high syntenic conservation shared among them (e.g. *aldh1a2* orthologs are in zebrafish Dre7, stickleback groupII and medaka Ola3), suggests that the loss of the *aldh1a-ogm* probably occurred in stem teleosts prior to the teleost radiation. Because asymmetric distribution of preserved duplicates appeared as a common feature of most of *aldh1a* genomic neighborhoods analyzed, the preservation of duplicated genes might be subject to functional constraints that depend on the local architecture of the genomic neighborhood. For instance, enhancers shared by several genes or embedded in distant genes [70] or coordinated epigenomic regulation based on chromatin architecture of the genomic neighborhood, as in the *Hox* clusters [71],[72], could bias paralog retention – loss from the first paralogon might be without penalty due to redundancy, but then the second paralogon would have to maintain certain aspects of its gene content.

#### *Aldh1a1* was lost during teleost evolution

**Figure 5:**
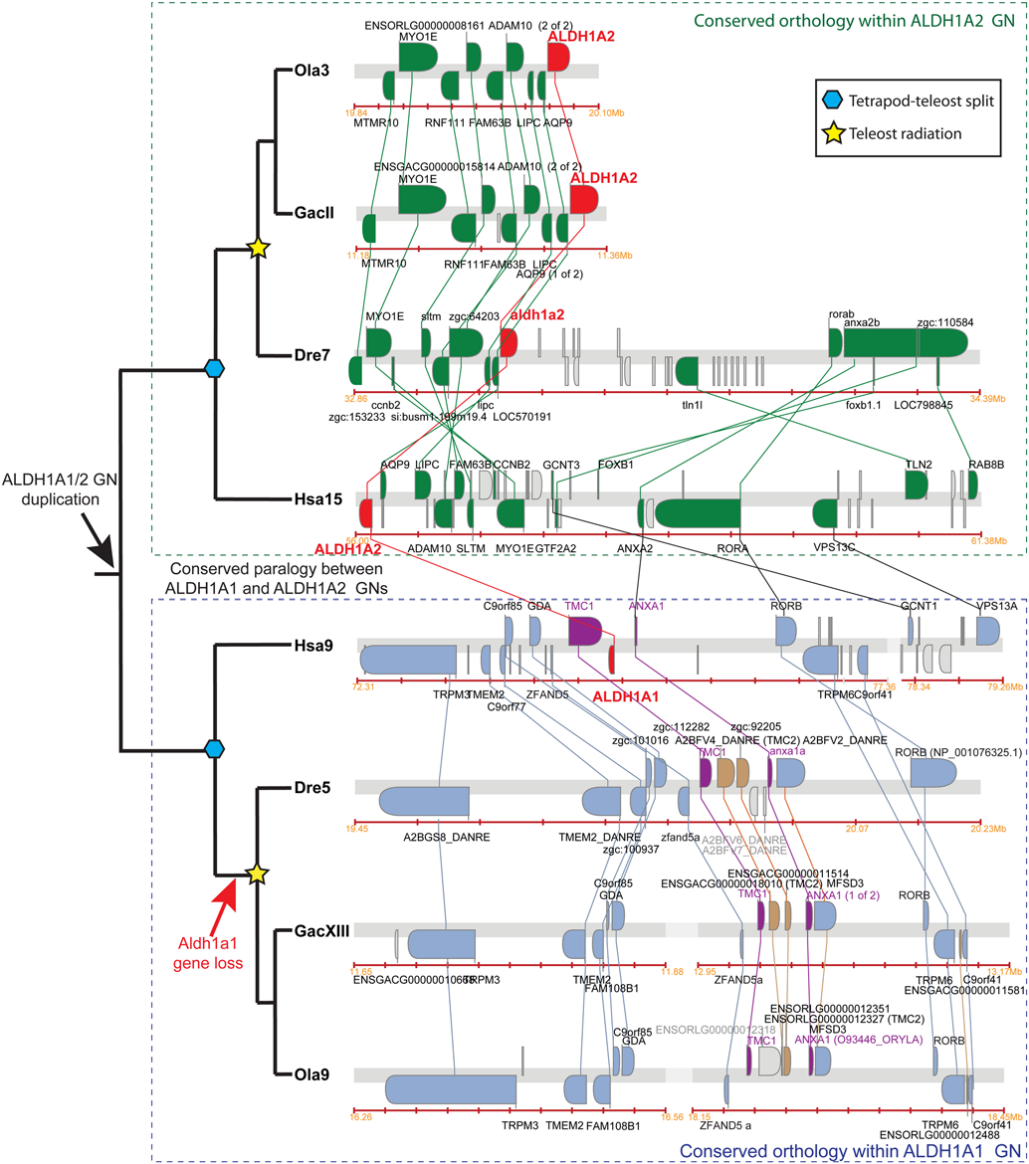
Conserved syntenies provide evidence that *aldh1a1* was lost in stem teleosts. This analysis provides evidence that the genomic duplication that generated *Aldh1a1* and *Aldh1a2* from an ancestral *Aldh1a1/2* gene predated the tetrapod-teleost divergence. *Aldh1a* gene family members are highlighted in red, and the nearest *Aldh1a1* conserved syntenic genes are labeled in purple. Teleost-specific *tmc* tandem duplicates located in the position predicted for the lost *aldh1a1* are brown. This analysis suggests that the loss of the *aldh1a1* ortholog probably occurred before the teleost radiation, which rules out the hypothesis that *Aldh1a1* is a tetrapod innovation. Additional paralogous clusters with low conserved synteny with the Hsa ALDH1A1 genomic neighborhood were found in secondary chromosomal regions in teleost genomes (e.g. Dre8, GacXIV, and Ola12; included in Table S3 and Figure S2).

Despite the absence of *Aldh1a1* orthologs in teleost genomes, dotplots in Figure 4A–C revealed candidate primary chromosome regions that conserved synteny with the human *ALDH1A1* genomic neighborhood on zebrafish Dre5, stickleback groupXIII and medaka Ola9. Screening of these candidate orthologous chromosomal regions identified pair-wise clusters of human and teleost genes in the Synteny Database [48], and pointed to the putative chromosome location where *aldh1a1* would have been before it was lost (Figure 5). The lower clade of Figure 5 shows that in the human genome, *ALDH1A1* lies between *TMC1* and *ANXA1* (purple in Figure 5). In zebrafish, stickleback, and medaka, *tmc1* and *anxa1* are near neighbors, transcribed in the same direction as in human, but with no *aldh1a* gene between them. Instead, teleost-specific *tmc* tandemly duplicated genes occupy this gap, suggesting that local genomic reorganizations, including tandem *tmc* duplications, might be functionally related to the loss of the teleost *aldh1a1* gene.

The identification of conserved paralogous synteny between the *ALDH1A1* and the *ALDH1A2* genomic neighborhoods in human and teleosts (Figure 5) supports the hypothesis that *Aldh1a1* and *Aldh1a2* originated by a large-scale genomic duplication event before the divergence of tetrapods and teleosts, and therefore that *Aldh1a1* was lost during teleost evolution. Thus, we can conclude that *Aldh1a1* is not a tetrapod innovation, but its absence from teleost genomes is due to an evolutionary simplification in this lineage (Figure 5).

#### Evidence for loss of *aldh1a3* in medaka

**Figure 6:**
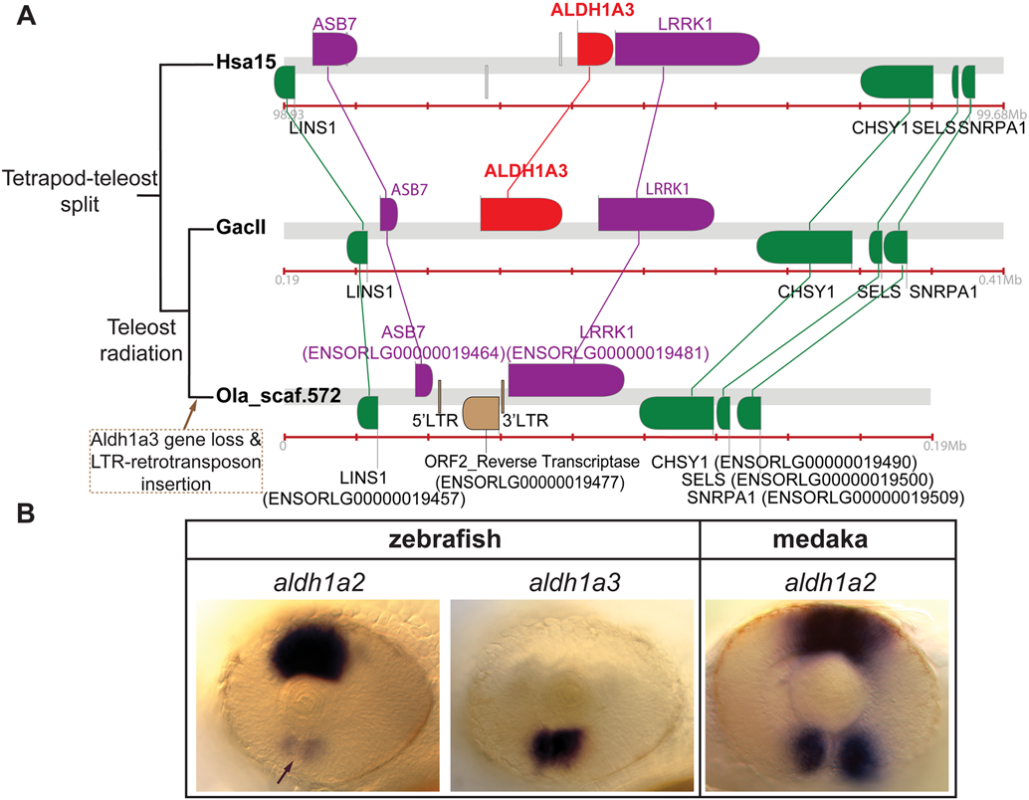
Conserved syntenies provide evidence that *aldh1a3* was secondarily lost in medaka. (A) Comparative syntenic analysis of *ALDH1A3* genomic neighborhoods in human, stickleback, and medaka. These results show that *aldh1a3* was lost in the medaka lineage. *ALDH1A3* orthologs are highlighted in red, and *ALDH1A3* nearest neighbors are labeled in purple. The presence of one LTR-flanked retrotransposon including an ORF2 reverse transcriptase (in brown) in the putative locus of the lost *aldh1a3* gene suggests the hypothesis that the insertion of the retrotransposon was related to the *aldh1a3* loss. (B) Comparative analysis by in situ hybridization of the expression of *aldh1a* gene family members in the developing eye of zebrafish and medaka reveals that the medaka *aldh1a2* gene recapitulates both the dorsal expression of *aldh1a2* and the ventral expression of *aldh1a3* in zebrafish. This result suggests that in medaka, *aldh1a2* provides a ventral RA source after the loss of *aldh1a3*.

Just as *aldh1a1* is missing in the teleost lineage, dotplot analysis (Figure 4C) showed that *aldh1a3* appeared to be missing from medaka but not from other percomorphs (Figure 4A–B). RT-PCR experiments with degenerate primers and *in silico* genomic surveys failed to identify *aldh1a3* in the medaka *Oryzia latipes*. BLAST searches of the medaka genome database, which has 9-fold coverage (http://dolphin.lab.nig.ac.jp/medaka), and EST databases, which have a total 584,144 sequences deposited in NCBI at http://www.ncbi.nlm.nih.gov/UniGene/UGOrg.cgi?TAXID=8090, (including the sequences from University of Tokyo at http://medaka.lab.nig.ac.jp/est_index.html [73] and the sequences from the National Bioresource Project at http://www.shigen.nig.ac.jp/medaka) did not identify any Aldh1a3 hits, while we found 28 EST sequences of the Aldh1a2, which appeared therefore as the only member of the Aldh1a family in medaka. Important evidence for the loss of *aldh1a3* from medaka comes from BLAST analysis that identified an unassembled medaka scaffold (scaffold572) containing genes that immediately flank *aldh1a3* in other species (i.e. *lins1*, *asb7*, *lrrk1*, *chsy1*, *sels* and *snrpa1*; Figure 6A and Table S3). We did not find evidence of a segment duplication of the genomic region in the scaffold572 that could be hiding a putative *aldh1a3* as an artifact of whole genome shotgun sequence assemblies [44]. No *aldh1a3*-like sequences could be identified to suggest the presence of an *aldh1a3* pseudogene in the 20-kilobase (Kb) long intergenic region between the *asb7* and *lrrk1* genes, where the *aldh1a3* should have been located according to syntenic conservation data from other species (Figure 6). Significantly, however, this intergenic region contains a 17 Kb genomic segment that is flanked by 140 bp long terminal repeats (LTR) (scaffold572 positions 43,994–44,136 and 60,837–60,972) and contains a retrotranscriptase sequence (ENSORLG00000019477) similar to the ORF2 of retrotransposons related to LINE elements of the CR1 family in fugu and zebrafish [74]–[76]. BLAST comparisons against the medaka genome revealed the presence of the same type of ORF2 associated with the same type of LTR sequences in other genomic locations where LTR retrotransposons have been predicted, such as the MHC Class I Region (GenBank accession number BA000027). The identification of an LTR retrotransposon at a site orthologous to the location of *aldh1a3* genes in other teleosts and in humans (Figure 6A) is exactly what would be expected under the hypothesis that the insertion of an LTR retrotransposon disrupted or silenced the *aldh1a3* gene in the medaka lineage after it diverged from the stickleback lineage. This molecular event may have led to the loss of *aldh1a3* in medaka. According to the results of the dotplot (Figure 4C), scaffold572 is likely to assemble eventually into medaka Ola3. Further sequencing of this genomic location or genetic mapping of a marker in this scaffold is necessary to corroborate this prediction.

#### Consequences of gene loss for the function of surviving paralogs

The apparent loss of *aldh1a3* in medaka made us wonder about the functional consequences of this loss and its potential effect on evolution in this lineage. Aldh1a3 plays a crucial role in the synthesis of retinoic acid, and it is especially important for the development of the ventral part of the eye, where a strongly conserved *Aldh1a3* expression domain has been described in all vertebrates studied so far, including mouse, chicken, *Xenopus* and zebrafish [58], [65], [77]–[84]. In mouse, for instance, homozygous *Aldh1a3* mutant embryos fail to complete the formation of the ventral cup of the eye and the closure of the chorioid fissure, resembling aberrations observed in colobomas in human retinas of patients with cat eye syndrome [82],[85].

In medaka, two alternative hypotheses might explain the effects of *aldh1a3* gene loss in the development of the eye: Under hypothesis 1, the development of the ventral part of the eye in medaka may have become independent of RA; under hypothesis 2, medaka might have an alternative mechanism to supply RA ventrally during eye development. To test these hypotheses, we cloned medaka *aldh1a2* cDNA (submitted to GenBank FJ516380), used it as probe to study expression patterns during eye development in medaka embryos, and compared its expression pattern to that of *aldh1a2* and *aldh1a3* in zebrafish embryos. In zebrafish, *aldh1a2* and *aldh1a3* are expressed in the neural retina in two sectors along the dorso-ventral axis in 1.5 day post-fertilization (dpf) embryos (Figure 6B). The *aldh1a2* gene is strongly expressed dorsally and *aldh1a3* is expressed ventrally (Figure 6B), confirming earlier results [65],[84],[86],[87]. Low signal of *aldh1a2* ventral expression was still observed remaining from earlier stages before optic cup invagination is completed, but the signal disappeared at later stages. We found that in medaka embryos, after optic cup invagination, *aldh1a2* was expressed strongly both dorsally and ventrally, a pattern that recapitulates the sum of the expression domains of *aldh1a2* and *aldh1a3* genes in zebrafish (Figure 6B). The ventral *aldh1a2* expression domain does not disappear at later stages in medaka (data not shown), in contrast to zebrafish or mouse, in which *aldh1a2* is down-regulated by the time that optic cup formation is completed [88]. Thus, our findings support hypothesis 2, in which medaka Aldh1a2 provides an alternative ventral source of RA in the absence of *aldh1a3*, in contrast to zebrafish in which ventral RA is supplied by Aldh1a3. This situation could have arisen by the evolutionary gain of the ability to express *aldh1a2* at high levels in the ventral domain of the developing eye after optic cup invagination in the medaka lineage.

## Discussion

This work illustrates how comparative analysis of whole genomes is important for functional connectivities between humans and model organisms. Analysis of conserved syntenies related to individual gene families helps identify lineage-specific gene gains and losses that can translate to evolving developmental mechanisms. Using the evolution of the Aldh1a family as a case study, we sought to probe the general mechanisms underlying the impact of gene loss on the functional fate of surviving paralogs after genome duplications while preserving unaltered ancestral developmental programs.

### Gene Loss in Teleosts Followed Expansion of the *Aldh1a* Family in Stem Vertebrates

Retinoic acid plays important morphogenetic roles in chordate embryonic development. The recent identification of components of the RA genetic machinery in non-chordate deuterostomes and in protostomes opens the possibility that expansion and reduction in RA-related gene families could have played a role in the developmental diversification of bilaterians [51],[52]. The *Aldh1a* gene family, which encodes enzymes that synthesize RA, has expanded independently several times during the evolution of the three chordate subphyla, the Cephalochordata, Urochordata and Vertebrata [59]. Within vertebrates, the expansion of the *Aldh1a* family generated three main paralogs - *Aldh1a1*, *Aldh1a2* and *Aldh1a3* - but the phylogenetic relationships and origins of these genes remained uncertain [59],[65].

To identify gene gains and losses, one must first reconstruct the evolutionary genomic history of a gene family. We undertook a combination of phylogenetic and comparative genomic analyses of conserved syntenies that clarified the evolutionary history of the Aldh1a family. Phylogenetic results showed that Aldh1a1 and Aldh1a2 form sister clades and Aldh1a3 occupies a basal position in the phylogenetic tree rooted on cephalochordate *Aldh1a* genes (Figure 1). This analysis breaks the trichotomy observed in one previous analysis [59] and is opposite to the topology rooted on the far more distant Aldh2 gene family in another analysis [65].

Further support for the new understanding of *Aldh1a* family member relationships ((Aldh1a1, Aldh1a2) Aldh1a3) comes from comparative genomic analyses of conserved syntenies in the genomic neighborhoods of Aldh1a paralogs in human, mouse, zebrafish, stickleback and medaka, which showed extensive conservation of syntenies between *Aldh1a1* and *Aldh1a2* genetic neighborhoods (Figure 2B). The congruency of the inferred historical relationships that arise from the new phylogeny and conserved syntenies, which are independent datasets, forces the conclusion that *Aldh1a1* and *Aldh1a2* are sisters and both are cousins to the *Aldh1a3* gene.

**Figure 7:**
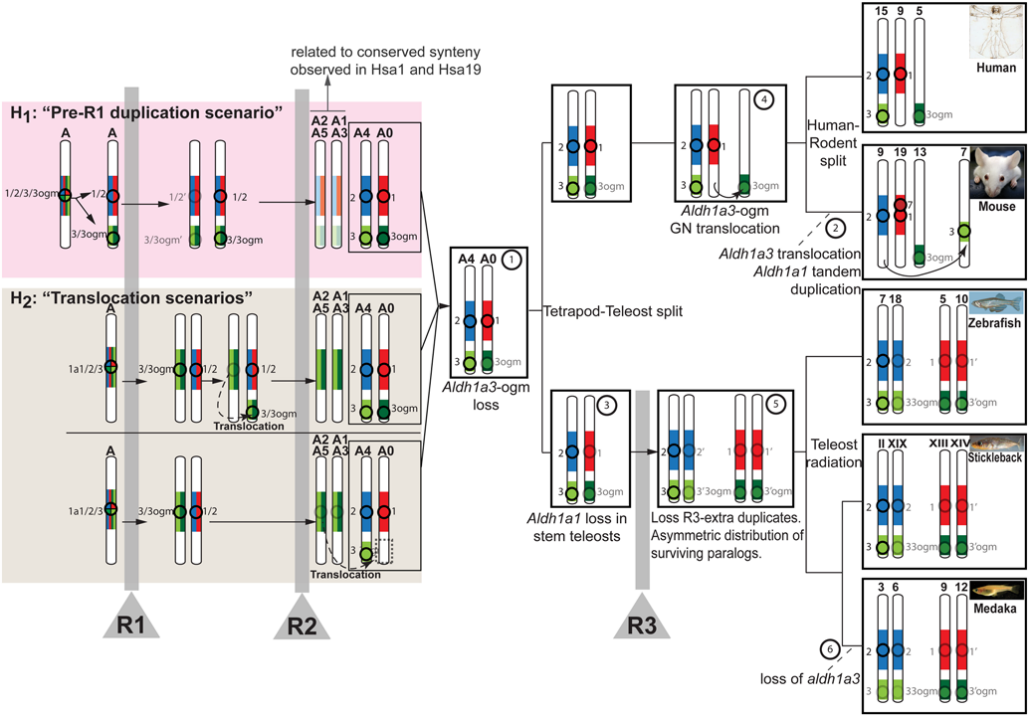
Evolutionary model reconstructs the history of the *Aldh1a* genomic neighborhoods from ancestral vertebrate chromosomes. Circles and numbers near chromosomes label Aldh1a paralogs, and their genomic neighborhoods are color-coded (*Aldh1a1*: red; *Aldh1a2*: blue; *Aldh1a3*: light green; and *Aldh1a3*-ogm: dark green). Duplication, preservation, losses and translocation of *Aldh1* gene paralogs are inferred in ancestral vertebrate chromosomes (e.g. A0–A5 [26]). Step numbers in circles label chromosome rearrangements. Vertical gray bars signify rounds of whole genome duplication events (R1, R2 and R3). Transparent images signify lost genes. In addition to the ancestral status inferred directly from comparative genomic analysis of conserved syntenies (white background; see main text for details), the figure shows two hypotheses (pink and tan boxes) to explain the mechanisms by which the *Aldh1a1/2/3/3-ogm* gene precursor located in the pre-R1 chromosome “A” generated the genome neighborhoods of *Aldh1a2* and *Aldh1a3* in chromosome “A4”, and *Aldh1a1* and *Aldh1a3-ogm* in “A0” inferred after R2 (step 1). Under hypothesis 1 (“pre-R1 duplication scenario” in the pink box), a segment from Nakatani et al.'s ancestral chromosome “A” including the original *Aldh1a1/2/3/3-ogm* gene was tandemly duplicated prior to R1 and gave rise to the *Aldh1a1/2* and *Aldh1a3/3-ogm* genes. Considering the most parsimonious situation, after R1, one of the two homeologs preserved both *Aldh1a1/2* and *Aldh1a3/3-ogm*, and the other homeolog lost both duplicated copies. After R2, the chromosome preserving the *Aldh1a* genes gave rise to “A4” and “A0”, from which today's *Aldh1a* gene family members have evolved. After R2, the chromosome that did not preserve an *Aldh1a* gene gave rise to “A2–A5” and “A1–A3”, explaining conserved syntenies related to the *Aldh1a* family observed in today's Hsa1 and Hsa19 (see Figure 2). An alternative hypothesis to explain the ancestral synteny of *Aldh1a* genomic neighborhoods inferred in A4 and A0 (hypothesis 2, the “translocation scenario” in the tan box) proposes a translocation event, which may have occurred either before R2 (top half of tan box) or after R2 (bottom half of tan box). In these scenarios, and in contrast to hypothesis 1, a single original gene *Aldh1a1/2/3/3-ogm* was present in the ancestral chromosome “A”, and after R1, *aldh1a1/2* and *aldh1a3/3-ogm* genes originated in duplicated chromosomes. One possibility (top half in tan box) is that, before R2, a small chromosomal translocation placed *Aldh1a1/2* and *Aldh1a3/3-ogm* on the same chromosome (dotted arrow in tan box). After R2, the chromosome “recipient” of the translocation gave rise to “A4” and “A0”, which contained all Aldh1a ancestral genes from today's Aldh1a family members, while the chromosome “donor” gave rise to “A2–A5” and “A1–A3”, which lacked any *Aldh1a* gene but still preserved syntenies for Aldh1a gene neighborhoods. The possibility that the translocation carrying *Aldh1a3* to the same chromosome as *Aldh1a2* could have occurred after R2 cannot be discarded (dotted arrow bottom half in tan box), and would be consistent with the absence of any *Aldh1a* paralog in Hsa5 (white box at the bottom on chromosome A0). In this case, however, we would not expect to find paralogs of genes that are tightly linked to *ALDH1A2* or *ALDH1A1* on Hsa5. We found, however, genes including *CCNB1*, *GCNT4*, *FAM81B* in Hsa5, whose paralogs *CNB2*, *GCNT3* and *FAM81A* are located near *ALDH1A2* in Hsa15, and *GCNT1*, a third *GCNT3* paralog, is close to *ALDH1A1* in Hsa9. Further gene translocations, however, could explain the presence of those genes in Hsa5, and therefore a hypothetical translocation after R2 cannot be discarded.

Based on results obtained from the analysis of synteny conservation of the *Aldh1a1* genomic neighborhoods across human and model organism genomes, we infer an evolutionary model that reconstructs the genomic history of the Aldh1a family, and integrates previous work by Nakatani et al., (2007) [26] that had reconstructed the re-organization of the ancestral chromosomes (named A to J) of the last common ancestor of vertebrates through R1, R2 and R3 genome duplications (Figure 7). Because *Aldh1a2* and *Aldh1a3* are syntenic (on the same chromosome) in human, zebrafish, and stickleback genomes, we conclude that this was the state in their last common ancestor (Figure 7 step 1). According to Nakatani's reconstruction, Hsa15 mostly derives from the post-R2 ancestral chromosome “A4”, which allows us to infer that Aldh1a2 and Aldh1a3 were syntenic in the ancestral chromosome A4 (Figure 7 step 1). After our comparative analysis of synteny conservation between human and mouse, which ruled out the possibility of reciprocal Aldh1a3 paralog losses (Figure 3) and showed that *Aldh1a3* genes are actual orthologs (Figure 1), we conclude that the *Aldh1a3*-ogm was already absent in the last common ancestor of tetrapods and teleosts (Figure 7 step 1). If *Aldh1a2* and *Aldh1a3* were syntenic in the ancestral state, we reason that a chromosomal translocation might have occurred during the evolution of the rodent lineage to separate them into different chromosomes (e.g Mmu9 and Mmu7 in Figure 7 step 2). Because the fourth *Aldh1a* paralog of rodents (i.e. *Aldh1a7* in mouse) is adjacent and oppositely oriented to *Aldh1a1*, separated only by 0.5 Mb with no intervening genes, we conclude that the fourth *Aldh1a* rodent paralog originated by a rodent-specific tandem gene duplication associated with a local inversion (Figure 7 step 2) that was probably followed by subsequent amino acid sequence changes that destroyed its ability to synthesize RA [64].

In contrast to tetrapods, teleosts lack an *Aldh1a1* ortholog, and whether this is due to a gene loss in teleosts, or a gene gain by tetrapods was previously unknown. Our whole-genome comparisons of conserved synteny answer this question by identifying genomic neighborhoods orthologous to the human ALDH1A1 genomic neighborhood in zebrafish, stickleback and medaka (Figure 5). This finding is consistent with the new *Aldh1a* phylogeny (Figure 1) and provides strong evidence supporting the conclusion that *Aldh1a1* was present in the last common ancestor before the tetrapod and teleost lineages split (Figure 7 step 1). Thus, we conclude that the absence of *Aldh1a1* in teleosts is due to gene loss, probably in stem teleosts or ealier in stem actinopterygians (Figure 7 step 3), and discards the hypothesis that *Aldh1a1* is a tetrapod innovation. This finding illustrates the power of comparative genomics to discern cases of gene losses from cases of gene gains, even in situations in which no proper outgroup is available.

In human and mouse, Aldh1a1 and Aldh1a3-ogm genomic neighborhoods are not syntenic (e.g. on Hsa9 and Hsa5, respectively). Interestingly, however, in zebrafish, stickleback and medaka, genomic neighborhoods orthologous to those of human *ALDH1A1* and *ALDH1A3-ogm* are syntenic (e.g., on Dre5, GacXIII and Ola9, see Figure 4A–C and right panels of Figure 7). Thus, just as *Aldh1a2* and *Aldh1a3* were syntenic after R2, it is likely that *Aldh1a1* and *Aldh1a3-ogm* were also syntenic before the tetrapod-teleost split (Figure 7 step 1). This reasoning lead us to conclude that there might be a chromosomal translocation event that separated the *Aldh1a1* and *Aldh1a3-ogm* genomic neighborhoods to two different chromosomes during the evolution of the tetrapod lineage (Figure 7 step 4). This predicted translocation event is supported by the reconstruction of ancestral chromosomes made by Nakatani et al. (2007), [26], in which a post-R2 ancestral chromosome named “A0” split into two main pieces that are today on Hsa5 and Hsa9. Thus, we conclude that *Aldh1a1* and *Aldh1a3-ogm* were syntenic in the ancestral chromosome A0 (Figure 7 step 1), which broke apart in tetrapods (Figure 7 step 4) but remained intact in the teleost lineage (Figure 7 step 3). Consistent with our finding that Hsa1 and Hsa9 are related to the *ALDH1A* genomic neighborhoods in the human genome despite their lack of *ALDH1A* genes (Figure 2A yellow boxes), Nakatani's reconstruction also shows that most of Hsa1 and Hsa19 derive from “A2–A5” and “A1–A3”, respectively, which are the other post-R2 homeologs derived from the ancestral chromosome “A” present in the genome of the last common pre-R1 vertebrate ancestor. Therefore, we conclude that the conserved synteny related to *ALDH1A* we detected in Hsa1, Hsa5, Hsa9, Hsa15 and Hsa19 originated by R1 and R2 from the ancestral chromosome “A” in the genome of the last common ancestor of vertebrates.

In Figure 7, we propose two hypotheses to explain how a single gene located in pre-duplication chromosome “A” generated *Aldh1a2* and *Aldh1a3* in ancestral chromosome “A4”, and Aldh1a1 and *Aldh1a3-ogm* in ancestral chromosome “A0” inferred after R2 (Figure 7 step 1). The first hypothesis suggests Aldh1a duplication before R1 (pink box in Figure 7), and the second hypothesis invokes a translocation either before or after R2 (tan box in Figure 7) (see legend in Figure 7 for details). Independently of the order of events, however, both scenarios agree that the first duplication generated *Aldh1a1/2* and *Aldh3/3-ogm* ancestral genes from the precursor *Aldh1a1/2/3/3-ogm* gene in the ancestral chromosome “A”. Because the available genomic databases of basally divergent vertebrates such as cartilaginous fishes (e.g. dogfish, little skate or elephant shark), or from basally divergent craniates (e.g. lampreys or hagfish), are still too fragmented to perform a comprehensive analysis of conserved synteny, testing the hypothetical “pre-R1 duplication” or “translocation” scenarios must be delayed.

### Asymmetric Chromosomal Distribution of Fish Surviving Co-Orthologs after R3

Supporting the postulated R3 teleost-specific genome duplication, our analysis of conserved synteny between the ALDH1A genomic neighborhoods and teleost genomes (Figure 4) revealed that fish orthologs of human *ALDH1A* neighbors are mostly confined to two main chromosomes in each fish species, and no extra R3-generated *aldh1a* ohnologs have been preserved in duplicated copies (Figure 7 step 5). Analysis of conserved synteny (Figure 4) supports the conclusion that each preserved duplicated *Aldh1a* gene is an actual ortholog of its partners within teleosts, and no evidence supports the complementary loss of *aldh1a* paralogs after R3 in different teleost lineages.

The distribution of conserved co-orthologs in teleost paralogons, however, was asymmetric. In each of four genomic regions for three teleost species, one homeolog (the primary chromosome) conserved substantially more genes in the observed region than the other chromosome (the secondary chromosome) (Figure 4D). This asymmetric distribution of syntenic gene conservation appears to be a common characteristic for R3-generated genomic neighborhoods, in agreement with previous observations of the analysis of the *hox* and parahox genomic neighborhoods in teleosts [17], [89]–[91] and the analysis of syntenic blocs formed following tetraploidy in *Arabidopsis* [92]. Evolutionary sequence divergence among paralogs also often display asymmetry, with one paralog evolving at a rate similar to its tetrapod ortholog and the other paralog evolving at an accelerated rate, suggesting neofunctionalization [39], [93]–[95]. During the analysis of the *hox* cluster it was noted that the fastest evolving *hox* genes belong to clusters that tend to lose their *hox* genes faster [89],[96]. Furthermore, the asymmetric distribution of synteny conservation between *parahox* cluster paralogons in teleosts, was accompanied by asymmetric accumulation of introns and repetitive DNA elements in type III RTK genes, and asymmetric conservation of potential regulatory elements [91]. Thus, our observation of asymmetric chromosomal distribution of surviving co-orthologs in the *aldh1a* genomic neighborhoods extend previous observations in the *hox* and *parahox* genomic regions, to genomic neighborhoods with a great variety of gene types, suggesting that the probability of duplicate gene preservation depends not only on inherent evolutionary forces depending on gene function (i.e. subfunctionalization and neofunctionalization), but also on properties pertaining to the architecture of the local genomic neighborhood. The R3 *Aldh1a*-ogm genes appear to represent cases in which, once gene organization had become altered in one of the duplicated regions, constraints that preserve genes became more relaxed, and therefore the chances of additional gene losses and further chromosomal rearrangements in the secondary chromosome were increased.

At least two possible mechanisms could explain asymmetric co-ortholog retention: first, enhancers shared or embedded in genes at distant sites [70],[91], or second, epigenetic regulatory mechanisms based on chromatin architecture [71],[72]. In principle, shared or distant enhancers or epigenetic regulatory signals must be retained in one homeolog, thus facilitating neighborhood gene retention, but can be lost from the other, allowing more gene loss and more rapid gene evolution due to greater relaxation of evolutionary constraints.

### Effects of Lineage-Specific Gene Loss on the Functional Fate of the Surviving Paralogs

In addition to the loss of *aldh1a1* in stem teleosts (Figure 7 step 3), our genomic surveys revealed that *aldh1a3* is absent from the genomic database of medaka fish (Figure 7 step 6). Identification of a genomic neighborhood in medaka that shows conserved orthologous synteny with the stickleback and human Aldh1a3 genomic neighborhoods (Figure 6) provides evidence that *aldh1a3* was lost in the medaka lineage after it diverged from the stickleback lineage (Figure 7 step 6). This finding illustrates again the power of comparative analysis of conserved synteny to provide evidence of gene loss. The finding of an apparent LTR-retrotransposon in the orthologous position occupied by *aldh1a3* in stickleback and human suggests that the insertion of this mobile element may have caused the loss of *aldh1a3* in medaka. Genomic data from medaka species phylogenetically close to *Oryzias latipes* is not yet available to more narrowly define the timing of this insertion event.

**Figure 8:**
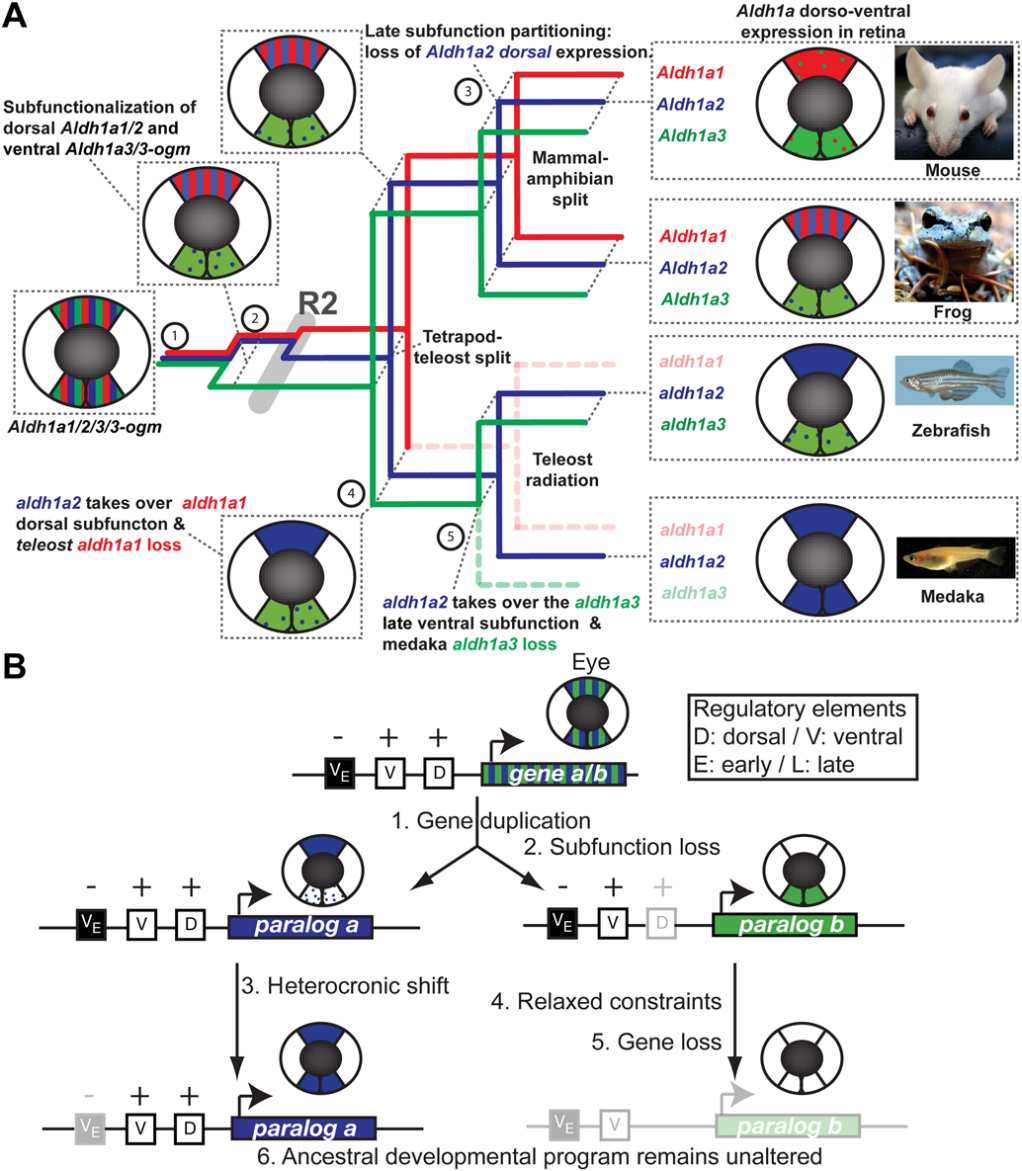
Consequences of lineage-specific gene loss on functional evolution of surviving paralogs. (A) Evolutionary model reconstructing the evolution of *Aldh1a* gene subfunctions in the developing retina. Mechanisms of early subfunctionalization, late subfunction partitioning, and acquisition or modification of ancestral subfunctions associated to events of gene duplication or gene loss (dotted lines) are indicated in the horizontal plane of a three-dimensional tree, in which events of vertebrate diversification are indicated in the vertical plane. A schematic retina at the stage of complete cup invagination represents dorso ventral (DV) expression domains of Aldh1a genes in different colors is indicated for present species and inferred for ancestral conditions. Bars indicate co-expression in the same dorso-ventral domains (or expression of ancestral genes), and dots refer to weak or remaining expression from earlier developmental stages. *Aldh1a1*: red, *Aldh1a2*: blue and *Aldh1a3*: green. *Aldh1a* expression data have been obtained from [58], [65], [69], [77], [79], [80], [84]–[87], [111]–[120] and this work Figure 6B). (B) Evolutionary mechanistic model to explain how the ancestral developmental program can remain unaltered after gene loss. This general model, extrapolated from our findings on the evolution of the expression of *Aldh1a* paralogs during eye development, is based on how heterochronic expression could facilitate the loss of a paralog, while leading to an apparent shuffling of functions between a lost paralog and a surviving paralog without the gain of new regulatory elements, but the loss of negative regulators.

The finding of the loss of *aldh1a3* in medaka makes this organism the first known vertebrate with a single surviving *Aldh1a* paralog (i.e. *aldh1a2*), and made us wonder about the functional implications of gene loss. As a measure of gene function, consider expression patterns of *Aldh1a* genes. In the developing retina of mouse, frog, zebrafish and medaka, *Aldh1a* genes are expressed in a dorsal sector and in a ventral sector at the completion of optic cup invagination (about E11.5 in mouse, stage 35 in frog, and 1.5 days post fertilization in zebrafish and medaka; Figure 8A). Different vertebrates express different *Aldh1a* genes in different dorso-ventral sectors of the eye. The right column of Figure 8 summarizes the main expression patterns of the Aldh1a family in the retina of different animals (*Aldh1a1* in red, *Aldh1a2* in blue, and *Aldh1a3* in green). *Aldh1a* paralogs expressed in the dorsal sector of the retina include *Aldh1a1* (but not *Aldh1a2*) in mouse; both *Aldh1a1* and *Aldh1a2* in frogs and birds (e.g. chicken and quail, not included in Figure 8A for simplicity); and *Aldh1a2* (but not *Aldh1a1*) in teleosts (e.g. zebrafish and medaka). The main *Aldh1a* paralog expressed in the ventral sector of the retina is *Aldh1a3* both in tetrapods (e.g. mouse, frog and birds) and in at least one teleost (e.g. zebrafish). In contrast, in medaka, which lacks an *aldh1a3* paralog, we found strong expression of *aldh1a2* ventrally (Figure 6). Dotted regions depict weak expression of *Aldh1a* genes in a small part of each dorso-ventral sector or from earlier developmental stages prior to the complete invagination of the optic cup in Figure 8A.

The rules of ancestral reconstruction imply that the retina of the last common vertebrate ancestor probably had a dorsal and a ventral sector, and the original *Aldh1a1/2/3/3-ogm* gene prior to the expansion of the *Aldh1a* family gene was likely expressed in both dorsal and ventral sectors (Figure 8A step 1). According to the evolutionary model proposed in Figure 7, the first expansion of the Aldh1a family occurred before R2 and generated *Aldh1a1/2* and *Aldh1a3/3-ogm*. Because *Aldh1a3* is the major paralog in the ventral sector of the retina in extant tetrapods and teleosts, and because *Aldh1a1* or *Aldh1a2* are the major paralogs in the dorsal sector of the retina in both tetrapods and teleosts, we infer that after the first duplication prior to R2, *Aldh1a1/2* inherited the subfunction leading to expression in the dorsal sector of the retina, and *Aldh1a3/3-ogm* inherited the subfunction causing expression in the ventral sector (Figure 8A step 2). It is probable that this subfunctionalization event contributed to the preservation of both paralogs as expected under the duplication-degeneration-complementation (DDC) model, in which the summation of the subfunctions that were split between gene duplicates equals the ancestral function prior the duplication event [3]. After R2, *Aldh1a3-ogm* was lost and *Aldh1a3* became the main ventral source of RA in the retina. On the other hand, both *Aldh1a1* and *Aldh1a2* retained expression in the dorsal sector because it is preserved in frog, chicken and quail, but not in mouse. Thus we conclude that the absence of *Aldh1a2* dorsal expression in mouse retina is due to a loss of an ancestral expression domain, which can be interpreted as an evolutionary innovation due to late subfunction partitioning [3], in which a function that was originally possessed by both Aldh1a1 and Aldh1a2 became partitioned exclusively to Aldh1a1 (Figure 8A step 3). Analysis of the *ALDH1A2* expression pattern in the human retina will help elucidate whether the loss of the *Aldh1a2* dorsal expression domain occurred before the split of lineages leading to human and rodents, or if it is a feature that has been acquired specifically in the rodent lineage.

An important question is how gene loss can impact the evolution of gene regulation and gene function in surviving paralogs. After the loss of *Aldh1a1* in teleosts, Aldh1a2 became the only source of RA in the dorsal retina, taking full responsibility for subfunctions originally shared with *Aldh1a1*. Natural selection would have gradually increased the strength of the ancestral dorsal domain of *Aldh1a2* (Figure 8A step 4). Medaka lacks both *aldh1a1* and *aldh1a3* orthologs, and the only surviving *Aldh1a* gene is *aldh1a2*, which is expressed in both the dorsal and ventral domains of the retina (Figure 8A step 5). The fact that in zebrafish and mouse, *Aldh1a2* is expressed early in the ventral retina prior to the closure of the optic cup and becomes progressively down-regulated until the completion of optic cup invagination (arrow in Figure 6B) [88], suggests that early expression followed by down-regulation of *Aldh1a2* is an ancestral feature and that medaka evolved an innovative heterochronic mechanism to avoid the ventral down-regulation of *aldh1a2* and to increase its ventral expression at later stages. Thus, it is likely that the dorsal and ventral paracrine sources of RA that have been suggested to regulate the development of perioptic mesenchimal derivative structures [56] is an ancestral feature that might be still preserved in teleosts. Comparative and functional analysis of the regulation of *aldh1a* paralogs during the development of the eye and other tissues in medaka, zebrafish and in other fishes, particularly outgroups, will be required to test this hypothesis.

The evolution of functions among Aldh1a paralogs illustrates what may be a general phenomenon associated with evolution after genome duplication: gene loss without altering developmental programs due to the preservation of functions in surviving paralogs. In our case study, the unaltered ancestral program provides both a dorsal and ventral supply of Aldh1a enzyme and hence dorsal and ventral sources of RA during retinal development. Comparative analysis shows that different paralogs can perform equivalent functions in different species. For instance, the ventral sector of the retina expresses *aldh1a2* in medaka and *aldh1a3* in zebrafish; and the dorsal sector of the retina expresses *Aldh1a1* in mouse and *aldh1a2* in zebrafish. Similar cases of what has been called *function shuffling* have been described for *Hox* genes [42]; *Bmp* genes [97], and *Twist* genes [43]. Gitelman (2007) proposed the term *synfunctionalization* to describe the process by which a paralog acquires the expression pattern of another paralog by gaining new regulatory elements, and thereby allowing losses of genes without changing the ancestral developmental program [43]. The acquisition of enhanced ventral expression by *aldh1a2* in the face of *aldh1a3* loss in medaka suggests several possible mechanisms for the apparent shuffling of functions between *aldh1a3* and *aldh1a2* that do not require the evolutionary gain of new regulatory elements (Figure 8B). Based on our findings, we propose a general mechanistic model to explain the loss of a paralog without altering the ancestral developmental program. After gene duplication from an ancestral *gene a/b* (Figure 8B Step 1), *paralog b* (e.g. *aldh1a3*) could lose the dorsal subfunction without penalty (Step 2) because it is covered by *paralog a* (e.g. *aldh1a2*). Next, mutations in negative regulatory elements or in upstream negative regulators that normally down-regulate *paralog a* expression in later developmental stages (e.g., after retina cup invagination) would facilitate an innovative heterochronic *paralog a* expression (Step 3). Finally, natural selection or genetic drift could act on natural variation that positively strengthens *paralog a* expression in the ventral domain (Step 3), while allowing relaxed selection for *paralog b* expression (Step 4), thereby facilitating the loss of *paralog b* (Step 5) without loss of the ancestral developmental program (Step 6).

Overall, our results illustrate how comparative genomic analyses of conserved synteny, coupled with reconstruction of ancestral chromosomes, can provide a phylogenetic framework necessary for the identification of lineage-specific gene losses. Our analysis provides evidence for early subfunctionalization and late subfunction-partitioning, and for the acquisition or modification of subfunctions by surviving paralogs that preserve unaltered ancestral developmental programs in the face of gene loss. Understanding the evolution of gene functions is fundamental for the proper interpretation of comparative analyses, especially when using model organisms to understand human gene functions. In the case of the Aldh1a family, although RA is important in human disease, we still know little about the spatio-temporal dynamics of the expression domains and functions of *ALDH1A1*, *ALDH1A2* and *ALDH1A3* genes during human development and adult organ homeostasis, other than RT-PCR studies [98], which do not provide enough resolution at the single cell level to understand how the sources of RA regulate physiological action. The evolutionary framework defined here provides information essential for the functional connectivity of human and model organism genomes, not only for RA signaling in eye development, but for the many organs in which RA plays important functions, including axis and limb development and cancer biology.

## Materials and Methods

### Ethics Statement

All animals were handled in strict accordance with good animal practice as defined by the relevant animal welfare bodies, and all animal work was approved by the University of Oregon Institutional Animal Care and Use Committee (A-3009-01, IACUC protocol #08-13).

### Phylogenetic Analysis

Alignments of ALDH1A proteins from vertebrates and cephalochordates were generated with clustalX [99] and corrected by eye. Only the unambiguous part of the alignment was considered for phylogenetic tree reconstructions (Figure S1 provides sequence alignments). The ProtTest tool was used to choose the best-fit protein evolutionary model [100], resulting in the LG+I+G [101] and the JTT+I+G [102] as the top two selected, with a relatively low value of deltaAIC = 92.92 (AIC = 18797.45 and 18890.37, respectively). Because different phylogenetic methods have different limitations [103], we compared results from four phylogenetic approaches: i) Bayesian phylogenetic inferences were calculated with MrBayes [104], using the JTT model as well as a gamma distribution for rate variation (divided into four categories) and a proportion of invariant sites. We ran two chains for 5 million generations, sampling every 100 iterations with a 25% burn-in. ii) Maximum-likelihood (ML) analysis was conducted using PHYML [105], with an LG+I+G and JTT+I+G model. The alpha parameter of the gamma distribution (1.41) and the proportion of invariable sites (0.19) were estimated from the sample, considering four categories of substitution rates. The topology, branch lengths, and rate parameters of the tree were required to be optimized. iii) Maximum-parsimony (MP) analysis (MEGA package, [106] used the close-neighbor-interchange approach with one level of search, and added 10 replicas of random trees, and 100 replications to calculate the bootstrap value that supports each node of the tree. iv) Neighbor-joining phylogenetic (NJ) tree (MEGA package, [106] was inferred taking into account among-site rate heterogeneity with four gamma-distributed categories. This approach has been previously shown to provide equivalent results to those obtained by ML under conditions of low sequence divergence, with the advantage of a low computing-time cost [107]. The alpha parameter 1.41 was estimated from the sample using PHYML under a JTT substitution model. Concordance of trees from each of the different methods, bootstrap proportions and posterior probability estimates were used to examine the robustness of nodes. Aldh1a1/2/3 proteins predicted from gene sequence data from the cephalochordate *Branchiostoma floridae* were used to root the phylogenetic tree of the vertebrate Aldh1a family. Tunicate Aldh1a1/2/3 proteins were not included to avoid possible artifacts arising from long branches shown previously for *Aldh* genes [59].

### Comparative Genomic Tools: Dotplots, Circleplots, and Orthologous and Paralogous Syntenic Gene Cluster Database

The automatic tools developed by Catchen et al. [48] to detect synteny conservation allowed us to perform comprehensive genomic comparisons between the human genome and other fully or partially assembled genomes from a wide variety of model organisms. These automatic tools use a reciprocal best hit BLAST analysis pipeline to define groups of paralogy between a primary genome and an outgroup genome. For instance, when the human genome is compared with outgroup genomes that diverged prior the two rounds of genome duplication R1 and R2 (i.e. the urochordate *Ciona intestinallis* or the cephalochordate *Branchiostoma floridae* in Figure 2A), each human gene will belong to a group of paralogy that is anchored to a gene from the outgroup genome. Use of multiple outgroup genomes and merging clusters anchored by outgroup paralogs help to minimize errors derived from the automatic reciprocal best hit BLAST pipeline due to the effect of losses, duplications or sequence divergence of outgroup genes (for details on best hit BLAST pipeline analysis, see [48]).

Dotplots graphically represent the distribution of paralogous genes (crosses) within the primary genome (e.g. Figure 2A), or the distribution of orthologous genes between the primary and outgroup genomes (e.g. Figure 3A), using the results generated with the automatic BLAST analysis pipeline. In the case of an orthology dotplot, genes belonging to a selected chromosome in the outgroup are displayed along the x-axis of the plot in the order they appear in that genome. Orthologs of those genes are displayed on their respective chromosomes in the primary genome directly above the location of the gene on the selected chromosome in the outgroup, not in their order in the second genome. Scaled dotplots represent a variant in which the paralogs (or orthologs) of genes on the selected chromosome are displayed according to their natural chromosomal positions in the genome (e.g. Figure 2A). For instance, given an orthologous dotplot with *Danio rerio* as the primary genome and human as the outgroup (Figure 4A), each two paralog genes originated by R3 in *Danio* will be aligned above their human ortholog on the x-axis. Composite dotplots overlap multiple dotplots from the analyses of various regions of interest (crosses labeled with different colors) and different outgroup genomes (e.g. Figure 2A). Circleplots represent user-selected chromosomes as arcs along the circumference of a circle. The origins of lines connecting positions along the arcs represent pairs of paralogous genes within the same species (Figure 2D–F) or orthologous genes between two different species (Figure 3B). Gene loci that are close to each other may appear overlapped as single crosses in the dotplot or a single connecting line in circle-plots due to the selected graph resolution.

Clusters in the Synteny Database were created by coupling results from the reciprocal best hit BLAST pipeline with the use of a sliding window analysis that links chromosome segments with conserved synteny (for details see [48]). Clusters that link chromosomal segments within the same species represent paralogous syntenic conservation (e.g. Figure 2B–C), and clusters that link chromosomal segments between different species represent orthologous syntenic conservation (e.g. Figure 4E–G). The Synteny Database provides clusters produced using several different sliding window sizes measured in terms of contiguous gene number. Smaller window sizes identify tightly-conserved syntenic regions in which gene order and orientation are well preserved while larger window sizes can accommodate chromosomal rearrangements (inversions, transpositions, translocations, and small duplications). The Synteny Database is especially useful to provide evidence of ohnologs gone missing (ogm) by uncovering the putative chromosomal region that still preserves paralogous syntenic conservation, but lacks a certain ohnolog of interest (e.g. Figures 5 and 6).

### Gene Cloning and Expression Analysis

Full coding sequence of *aldh1a2* cDNA from Medaka *Oryzias latipes* (Cab strain) and the *aldh1a3* cDNA from zebrafish *Danio rerio* were cloned after being amplified from cDNA by PCR with specific primers designed from genomic scaffold sequence data (medaka: 200506-scaffold21 and zebrafish: Zv5Scaffold1492 and NA2068) (Ola1a2F: 5′ATGACTTCCAGTAAGATCGAGATCCC3′ and Ola1a2R: 5′CATTAACGTTTCATCCATTACTGTCC3′; Dre1a3F: 5′GTCCACACAATAATCTACTCTACAGC3′; Dre1a3R 5′CATATGTTTGCGCTTAGCTGCCATG3′). Full length cDNA sequences were submitted to GenBank (medaka *aldh1a2*: FJ516380, and zebrafish *aldh1a3*: DQ300198). A zebrafish *adh1a2* clone [86], a clone containing a zebrafish *aldh1a3* 800 nt-fragment from exon 7 to exon 13 (cloning primers: 5′GGAGCTGCGATCGCTGGTCACATG3′ and 5′CTGAGTTTGATAGTGATGGCTTTGAC3′), and a clone containing a medaka *aldh1a2* 527-nt fragment from exon 12 (cloning primers: 5′GGAGGATACAAAATGTCTGGGAATGG3′) to the 3′UTR (5′CATTAACGTTTCATCCATTACTGTCC3′) were used to synthesize riboprobes for whole-mount in situ hybridization using standard procedures [108],[109], with slight variations: NBT and BCIP were used instead of the BM purple.

## Supporting Information

Figure S1 Phylogenetic trees of the vertebrate *Aldh1A* gene family, inferred by maximum-likelihood, neighbor-joining, maximum-parsimony, and Bayesian methods.
(4.46 MB PDF)

Figure S2 High-resolution images of the clusters of the Synteny Database.
(0.17 MB PDF)

Table S1 Supplementary information for dot-plot on Figure 2A.
(0.58 MB PDF)

Table S2 Supplementary information for dot-plot on Figure 3A.
(0.05 MB PDF)

Table S3 Supplementary information for dot-plots on Figure 4A–C.
(0.16 MB PDF)
