# Supplementary material for: Consequences of Lineage-Specific Gene Loss on Functional Evolution of Surviving Paralogs: ALDH1A and Retinoic Acid Signaling in Vertebrate Genomes
Source: PLoS Genet. 2009 May 29;5(5):e1000496. doi: 10.1371/journal.pgen.1000496 (PMC2682703; doi:10.1371/journal.pgen.1000496)
Supplement: Figure S1 — Phylogenetic trees of the vertebrate Aldh1A gene family, inferred by maximum-likelihood, neighbor-joining, maximum-parsimony, and Bayesian methods. (4.46 MB PDF) [file pgen.1000496.s001.pdf]

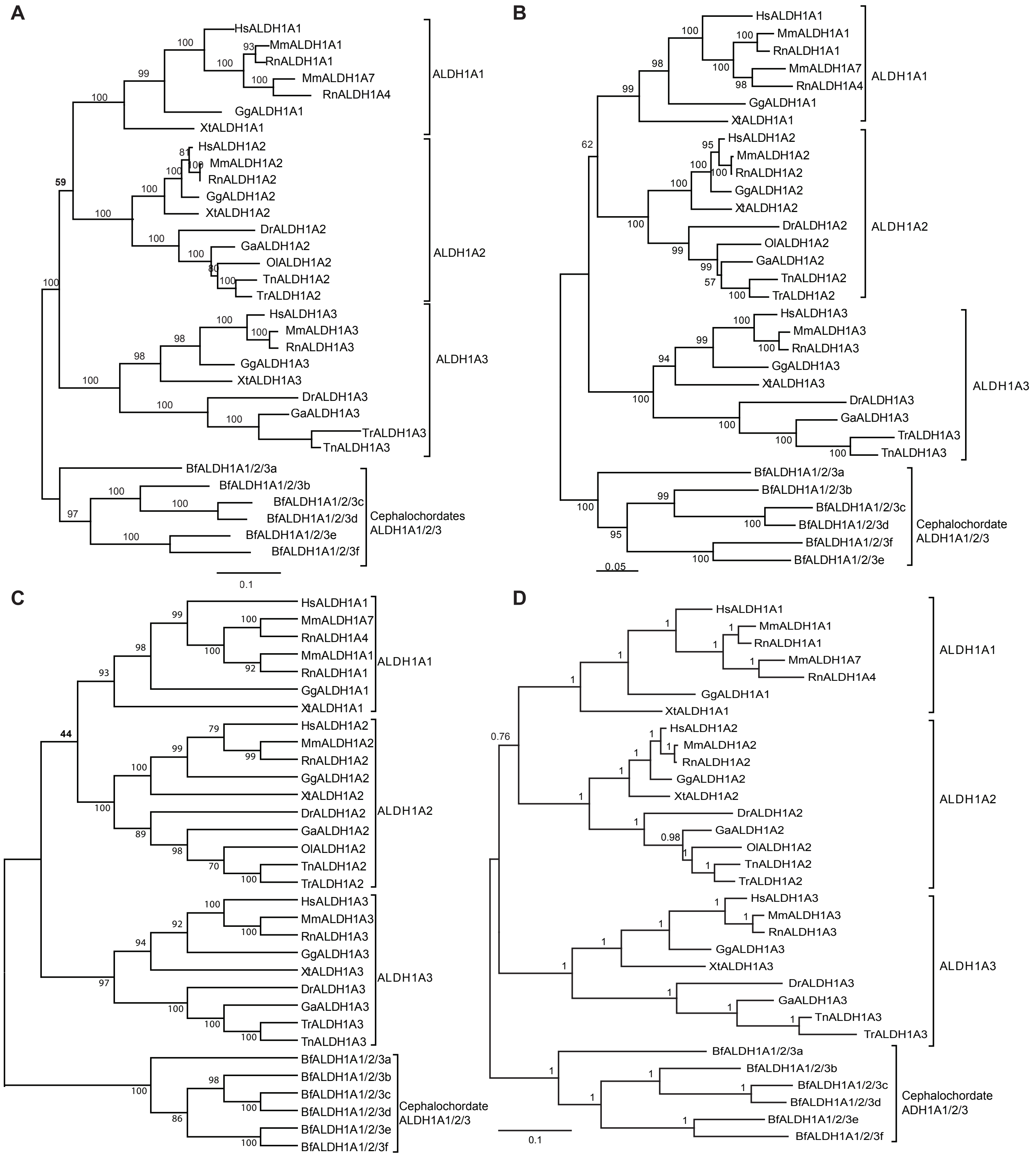

Figure S1. Phylogenetic trees of the vertebrate Aldh1A gene family, using the cephalochordate Aldh1A pro-orthologs to root the tree. The tree topology ((Aldh1a1, Aldh1a2), Aldh1a3) was obtained in all used phylogenetic methodologies: A: maximum-likelihood (ML); B: neighbor-joining (NJ); C: maximum-parsimony (MP); and D: bayesian method. Values in nodes indicate the boostrap values (A-C) and posterior probabilities (D) supporting each node of the trees. LG and JTT models rendered the same tree topology and minor differences in node support. Scale bar indicates amino-acid substitutions of branch lengths. Tetrapods: Hs, *Homo sapiens*; Mm, *Mus musculus*; Rn, *Rattus novergicus*; Gg, *Gallus gallus*; Xt, *Xenopus tropicalis*; Teleosts: Dr, *Danio rerio*; Ga, *Gasterosteus aculeatus*; Tn, *Tetraodon nigroviridis*; Tr, *Takifugu rubripes*; Cephalochordates: Bf, *Branchiostoma floridae*. Protein alignment is provided below. Accession numbers correspond to those in Cañestro et al., (2006) [59]

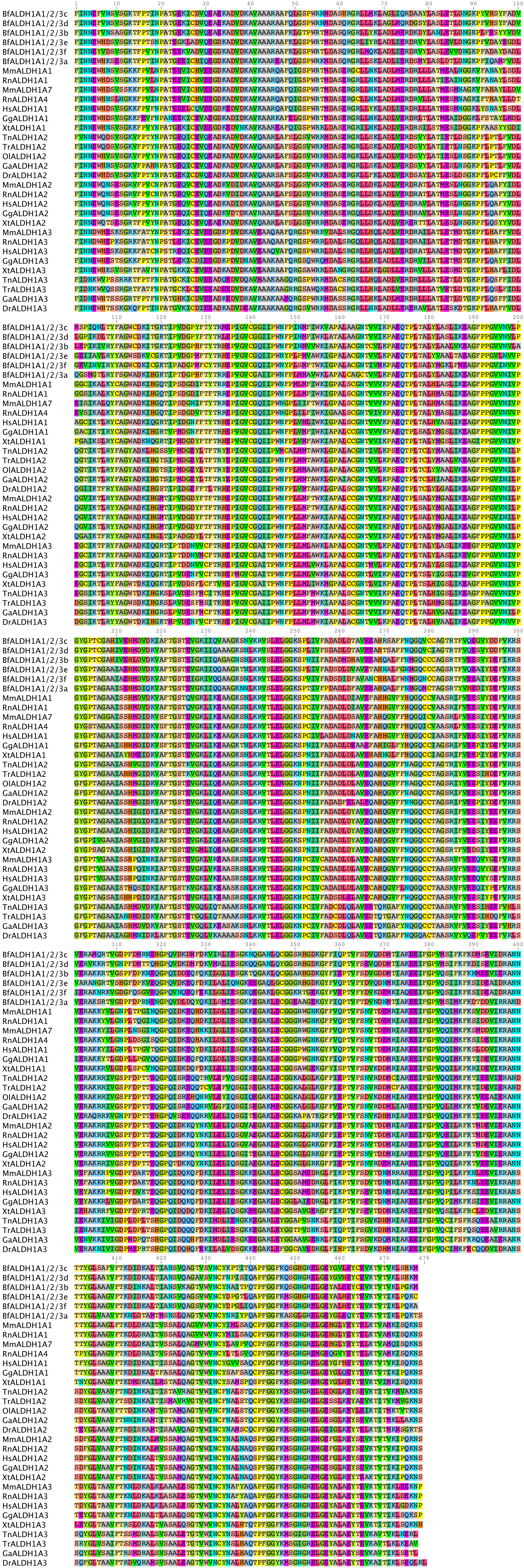

1 10 20 30 40 50 60 70 80 90 100
110 120 130 140 150 160 170 180 190 200
210 220 230 240 250 260 270 280 290 300
310 320 330 340 350 360 370 380 390 400
410 420 430 440 450 460 470 479
BfALDH1A1/2/3c
BfALDH1A1/2/3d
BfALDH1A1/2/3b
BfALDH1A1/2/3e
BfALDH1A1/2/3f
BfALDH1A1/2/3a
MmALDH1A1
RnALDH1A1
MmALDH1A7
RnALDH1A4
HsALDH1A1
GgALDH1A1
XtALDH1A1
TnALDH1A2
TrALDH1A2
OlALDH1A2
GaALDH1A2
DrALDH1A2
MmALDH1A2
RnALDH1A2
HsALDH1A2
GgALDH1A2
XtALDH1A2
MmALDH1A3
RnALDH1A3
HsALDH1A3
GgALDH1A3
XtALDH1A3
TnALDH1A3
TrALDH1A3
GaALDH1A3
DrALDH1A3
