# Supplementary material for: Consequences of Lineage-Specific Gene Loss on Functional Evolution of Surviving Paralogs: ALDH1A and Retinoic Acid Signaling in Vertebrate Genomes
Source: PLoS Genet. 2009 May 29;5(5):e1000496. doi: 10.1371/journal.pgen.1000496 (PMC2682703; doi:10.1371/journal.pgen.1000496)
Supplement: Figure S2 — High-resolution images of the clusters of the Synteny Database. (0.17 MB PDF) [file pgen.1000496.s002.pdf]

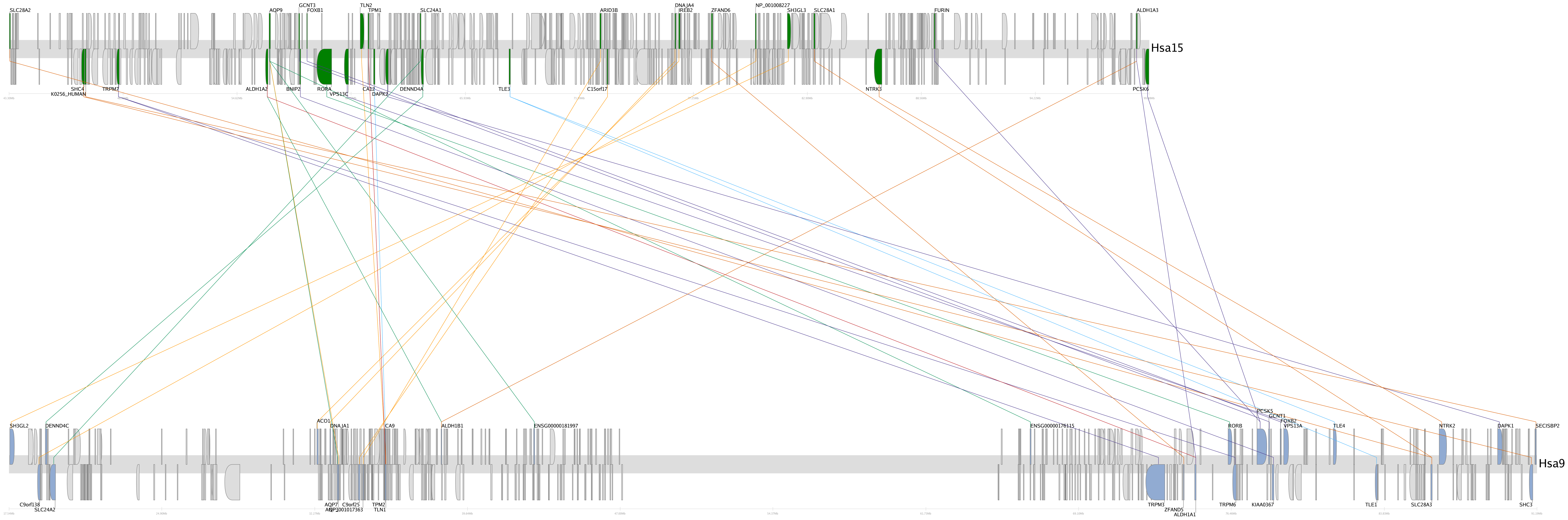


High resolution image shown on figure 2B: pair-wise gene cluster on Hsa15 and Hsa9 showing paralogous high syntenic conservation between ALDH1A1 and ALDH1A2 GNs, but low with ALDH1A3 GN.

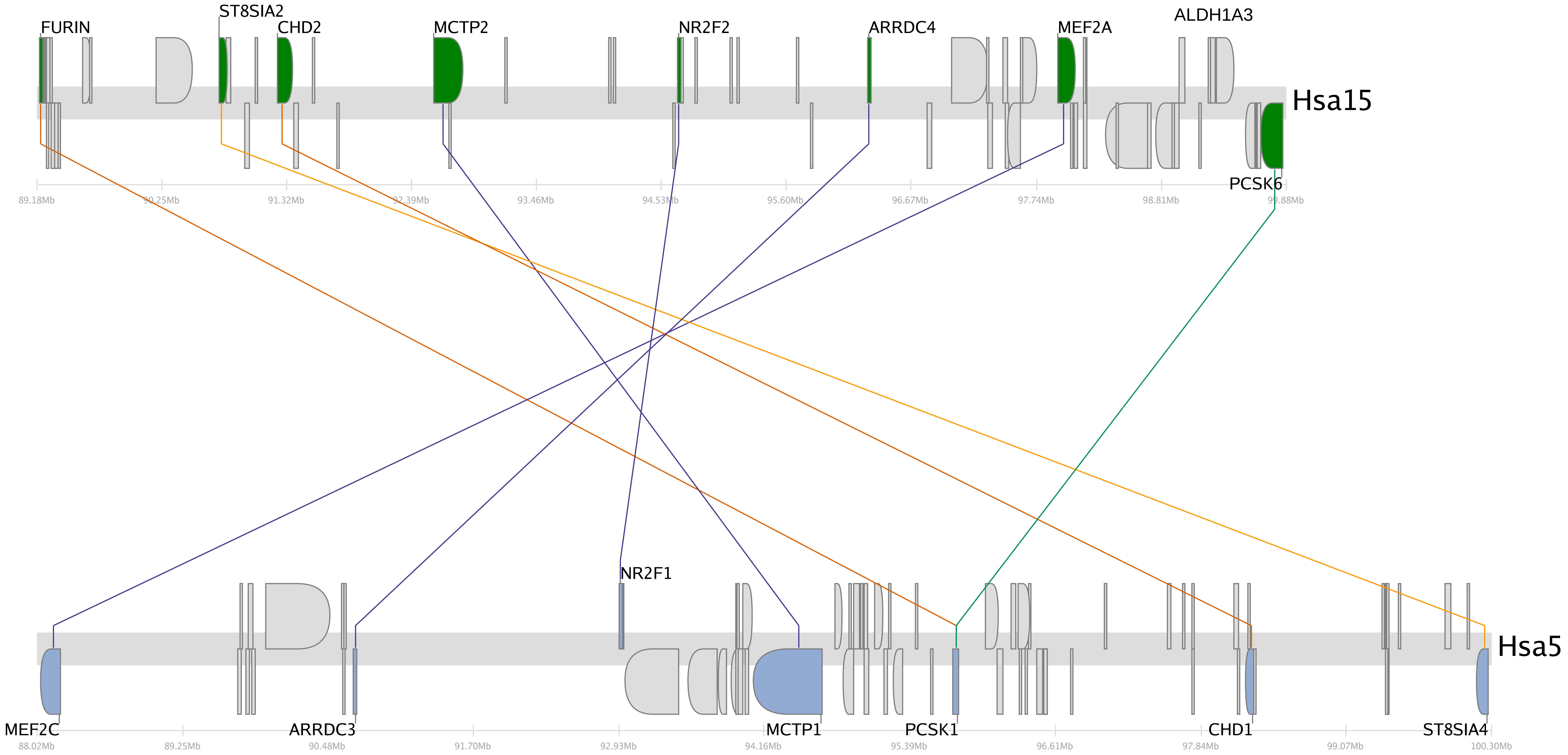


High resolution image shown on figure 2C: pair-wise gene cluster on Hsa15 and Hsa5 showing paralogous high syntenic conservation between ALDH1A3 and ALDH1A3-ogm GNs.

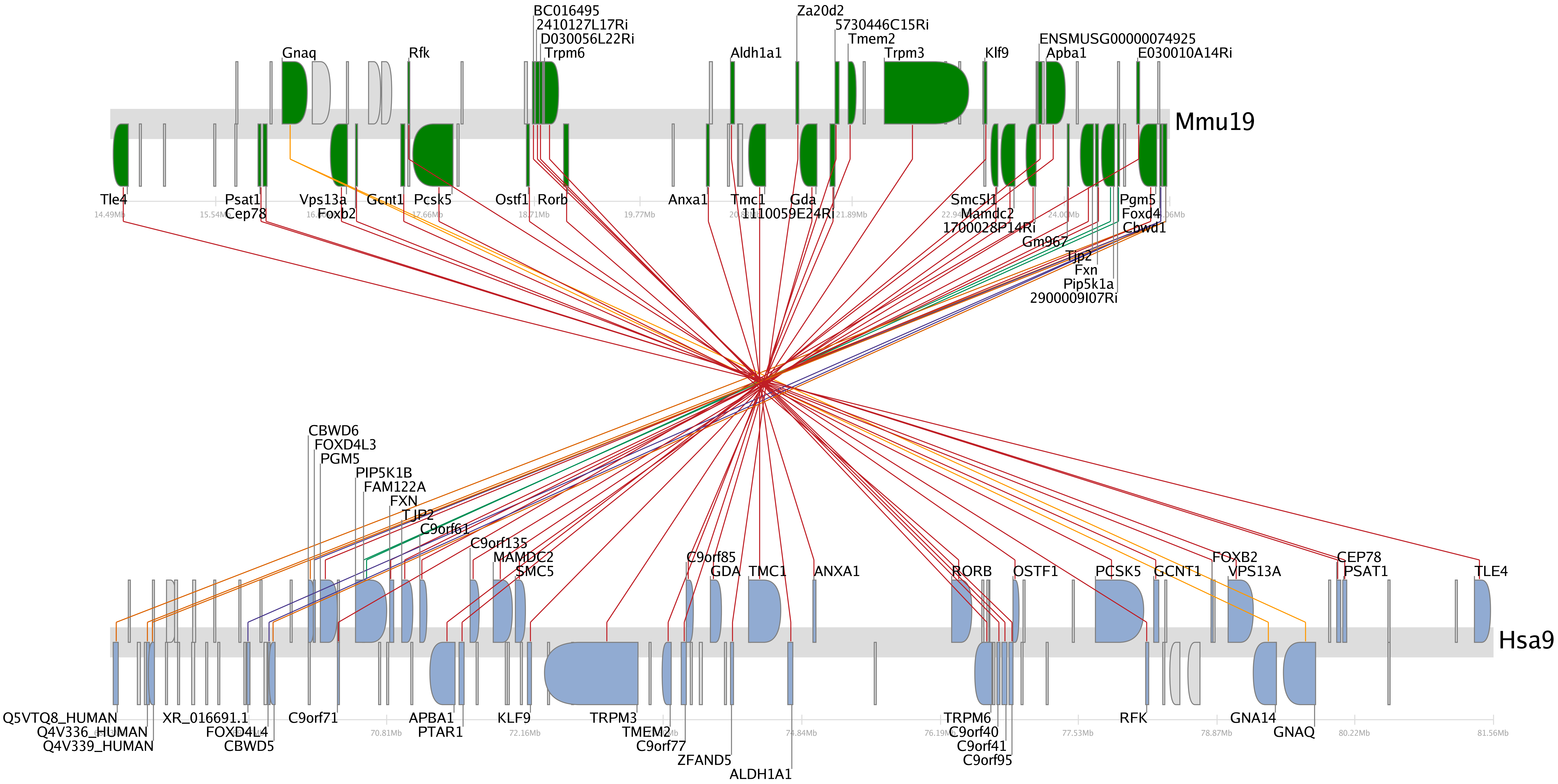


Pair-wise gene cluster on Mmu19 and Hsa9 showing orthologous high syntenic conservation between 46 genes from the human and mouse ALDH1A1 GNs.

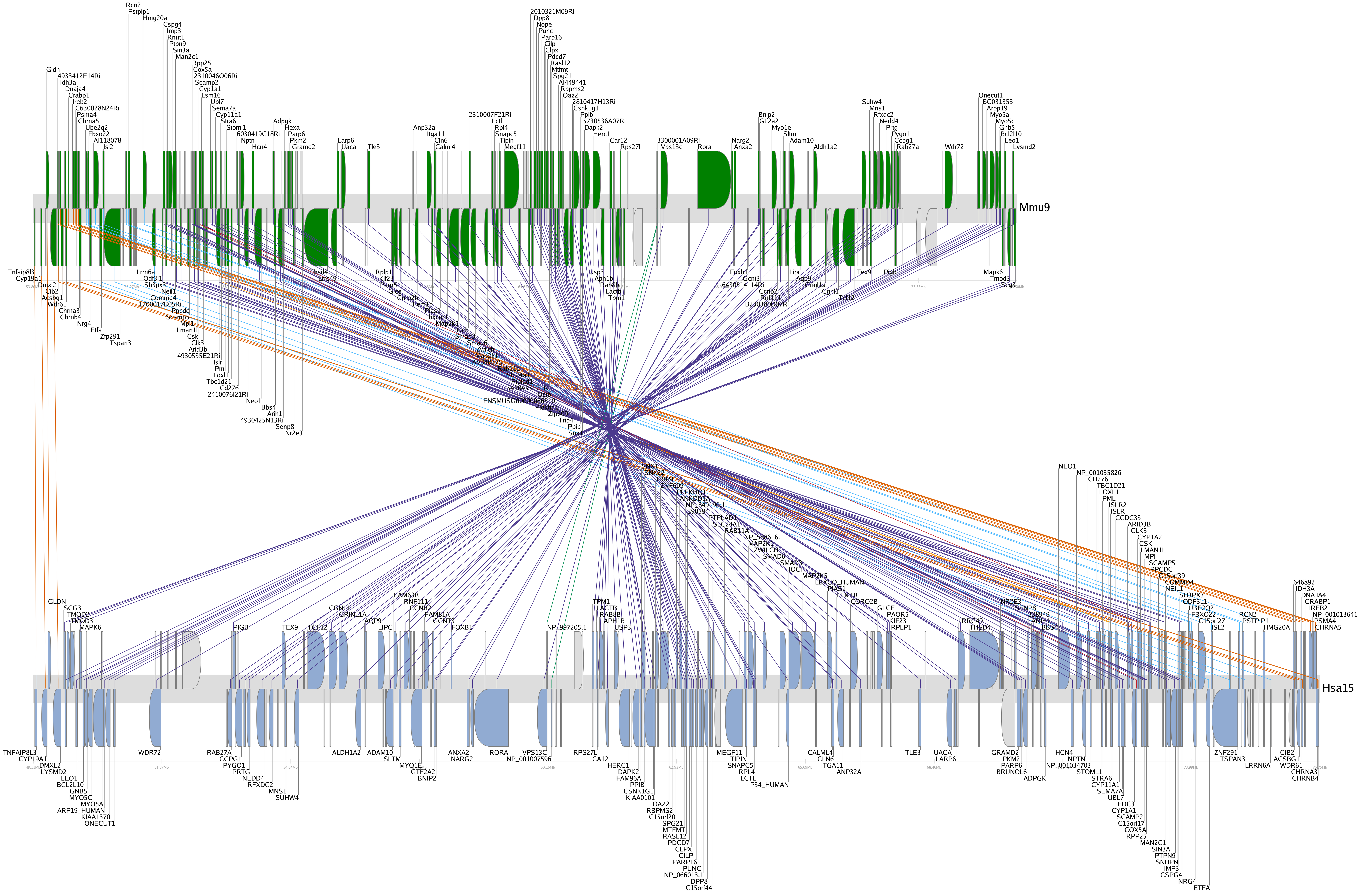


Pair-wise gene cluster on Mmu9 and Hsa9 showing orthologous high syntenic conservation between 196 genes from the human and mouse ALDH1A2 GNs.

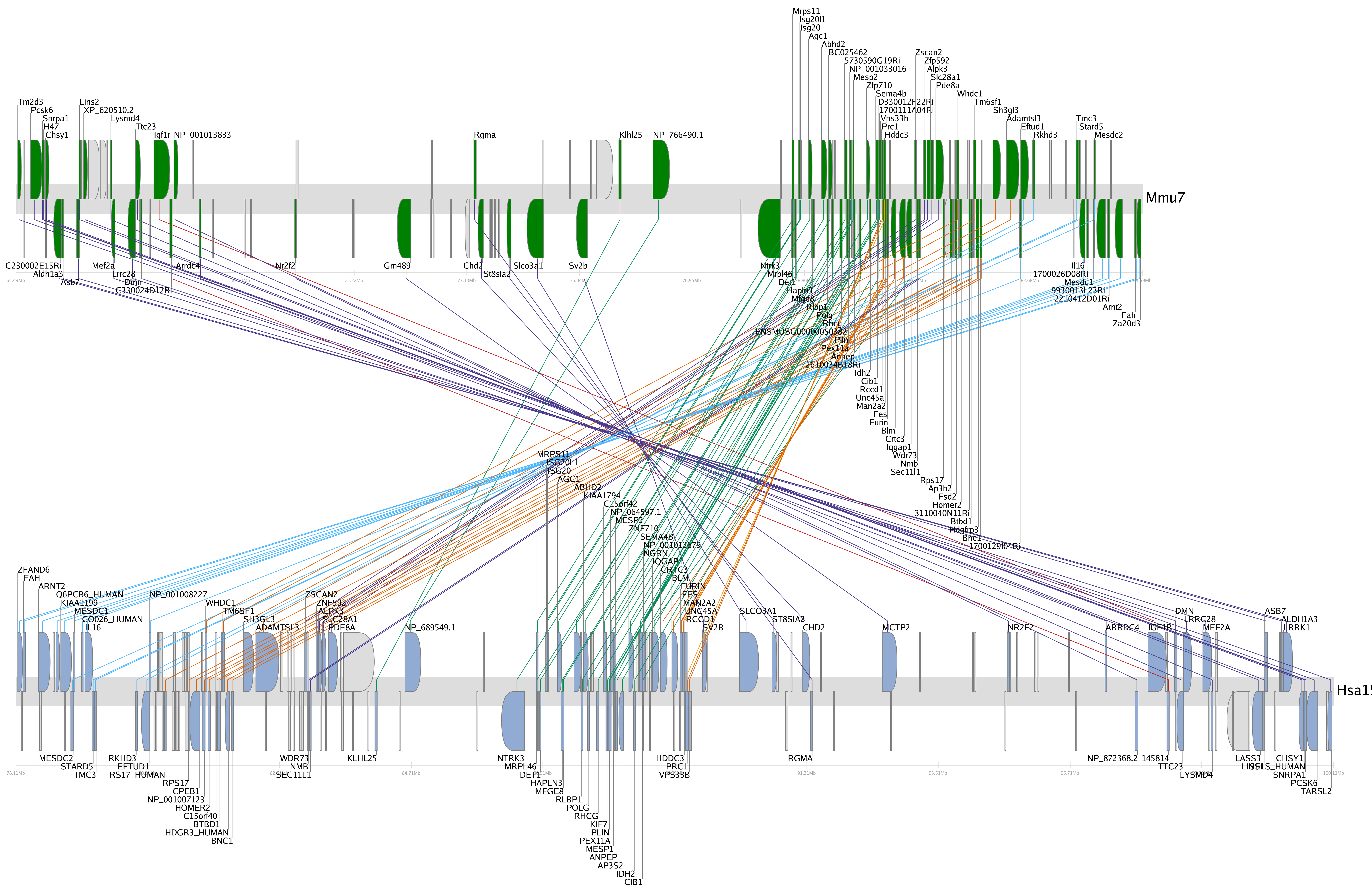


Pair-wise gene cluster on Mmu7 and Hsa15 showing orthologous high syntenic conservation between 103 genes from the human and mouse ALDH1A3 GNs.

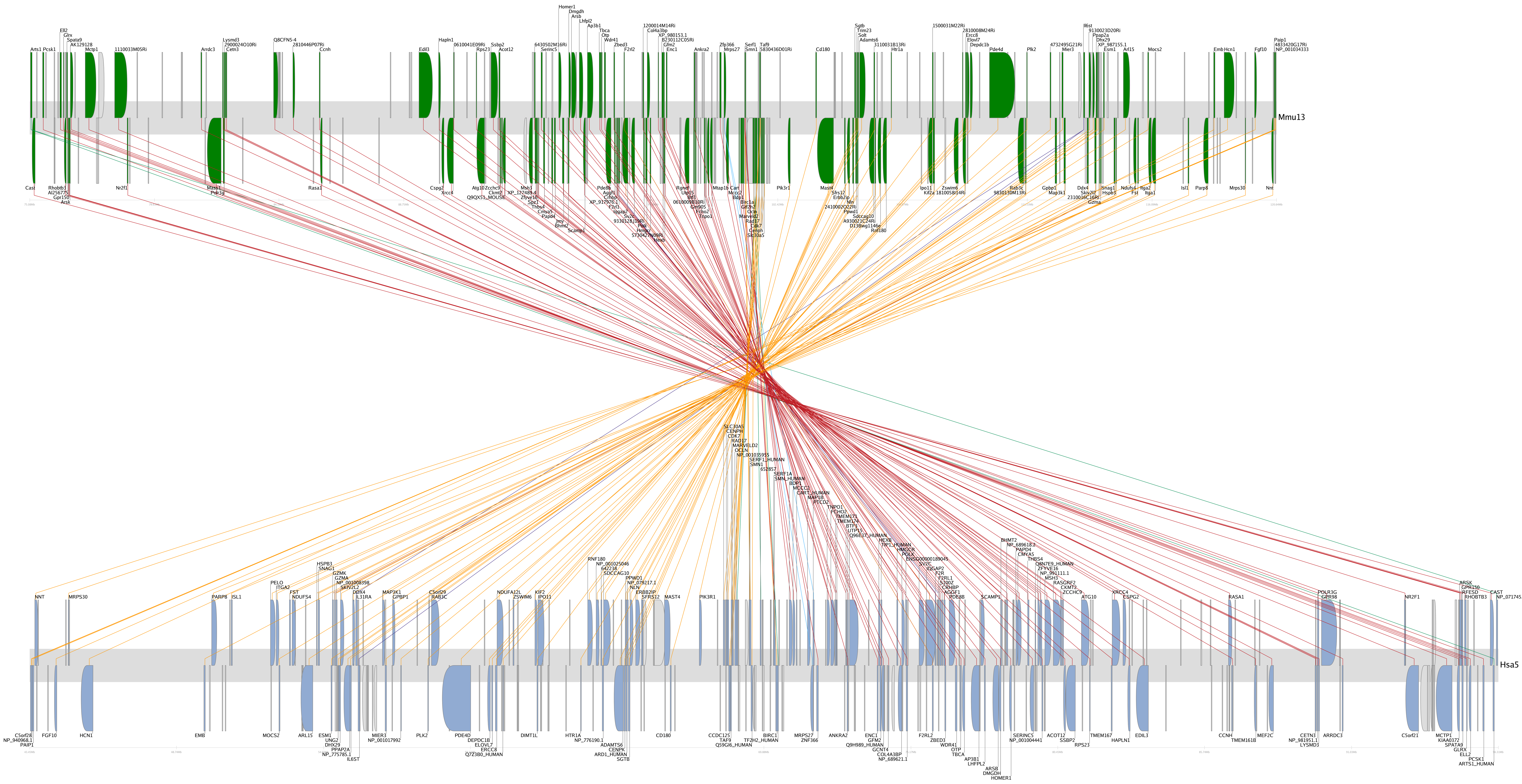


Pair-wise gene cluster on Mmu13 and Hsa5 showing orthologous high syntenic conservation between 175 genes from the human and mouse ALDH1A3-ogm GNs.

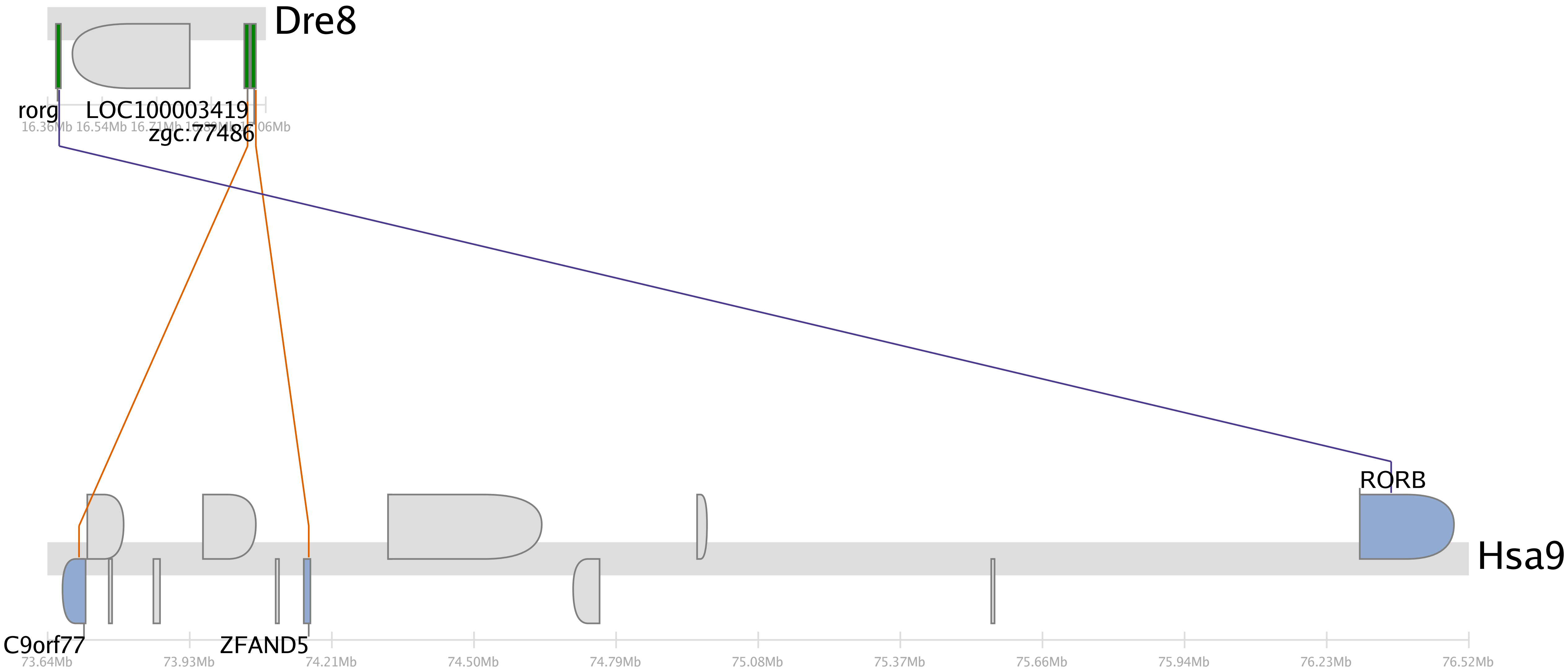


Orthologous syntenic cluster between the ALDH1A1 GN on Hsa9 and a secondary co-orthologous chromosomal region in zebrafish Dre8.

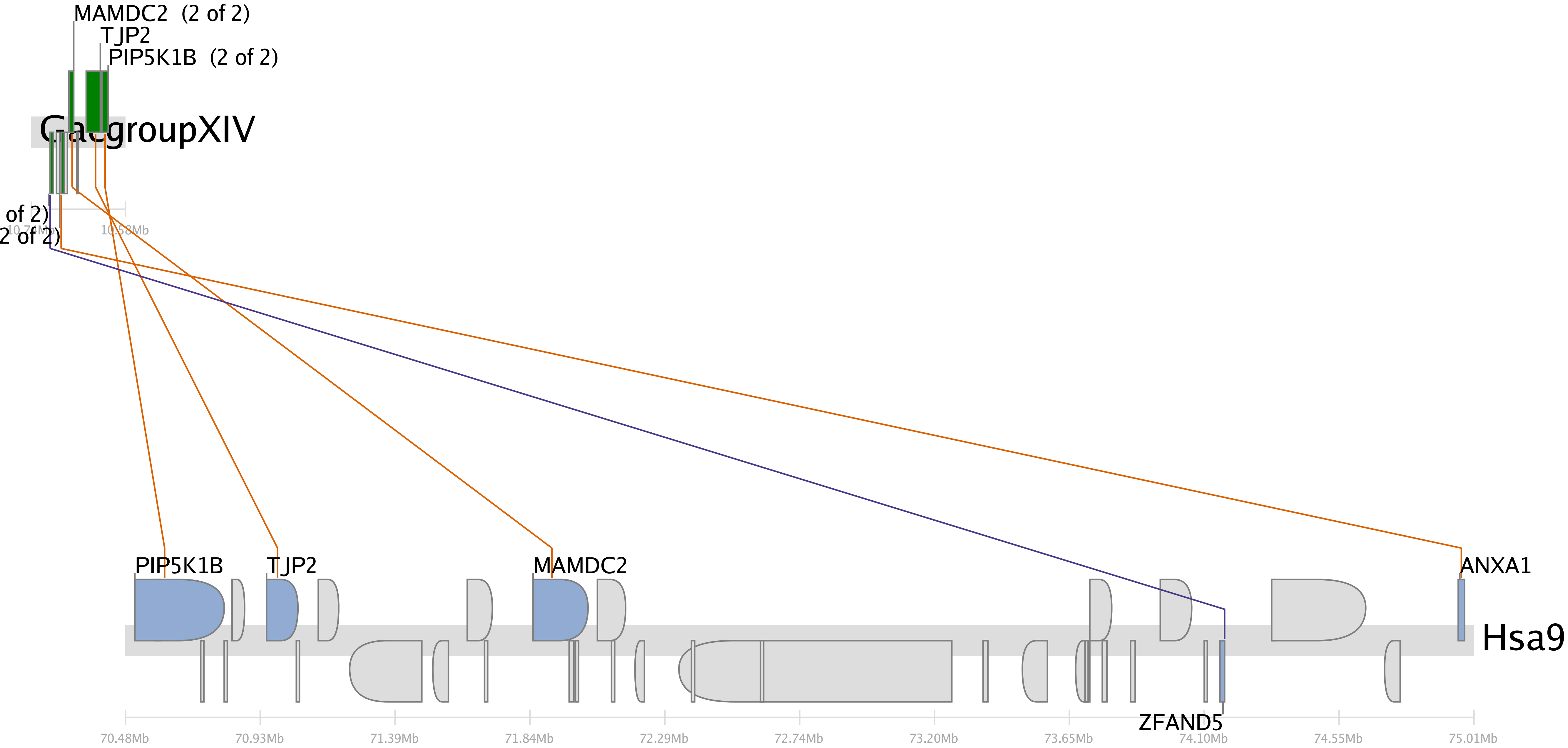


Orthologous syntenic cluster between the ALDH1A1 GN on Hsa9 and a secondary co-orthologous chromosomal region in stickleback GacXIV.

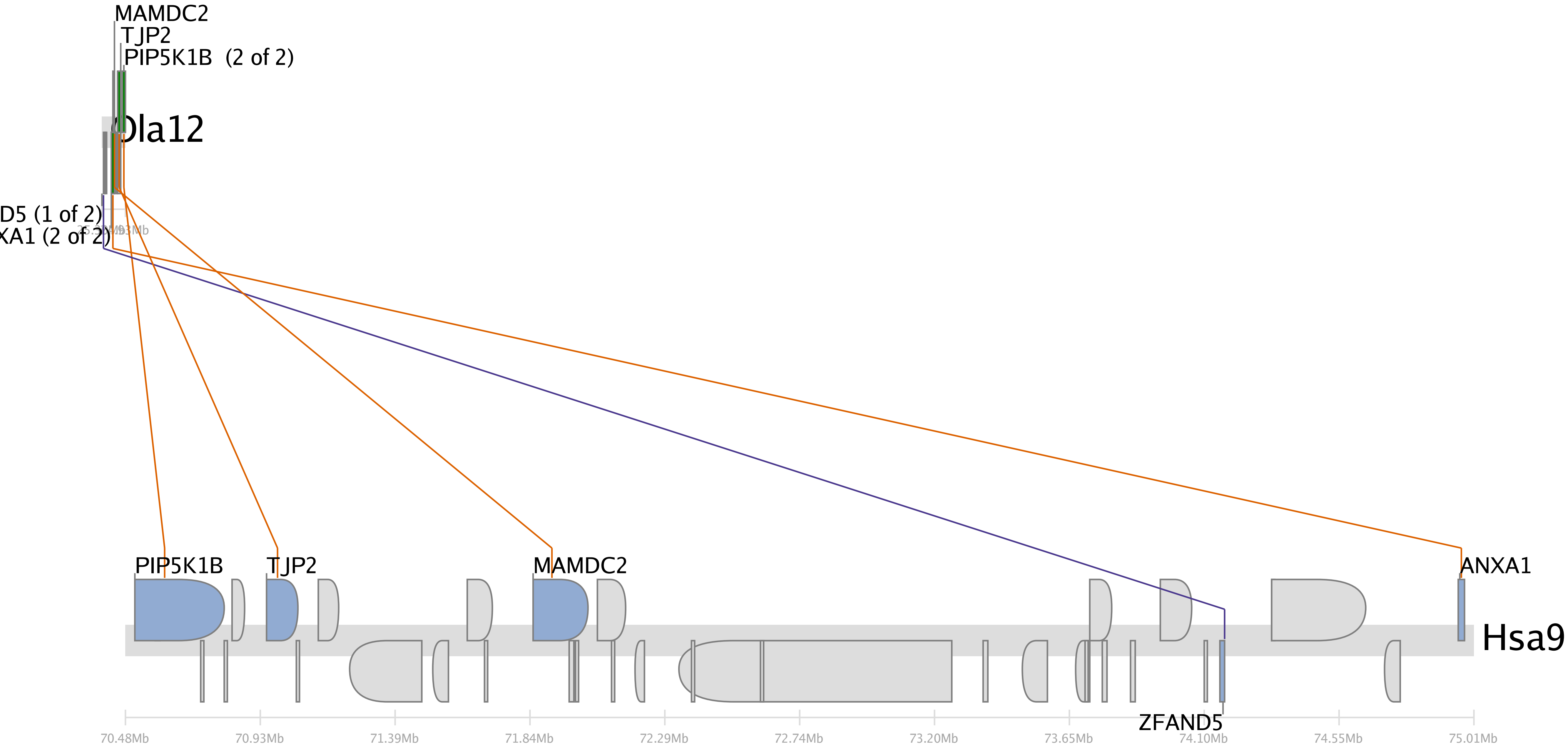


Orthologous syntenic cluster between the ALDH1A1 GN on Hsa9 and a secondary co-orthologous chromosomal region in medaka Ola12.
