# Supplementary material for: Consequences of Lineage-Specific Gene Loss on Functional Evolution of Surviving Paralogs: ALDH1A and Retinoic Acid Signaling in Vertebrate Genomes
Source: PLoS Genet. 2009 May 29;5(5):e1000496. doi: 10.1371/journal.pgen.1000496 (PMC2682703; doi:10.1371/journal.pgen.1000496)
Supplement: Table S1 — Supplementary information for dot-plot on Figure 2A. (0.58 MB PDF) [file pgen.1000496.s003.pdf]

Table S1. Supplementary information for dot-plot on Figure 2A

Genomic distribution of the paralog groups of the surrounding 10-Mb window including the ALDH1A1, using the Branchiostoma floridae genomic database as the outgroup for the best reciprocal BLAST hit analysis								
Genomic regiona analyzed: human chromosome 9 (69.000 KB - 79.000 KB)								
Group ID	Query Gene ID	Query Ext ID	Query Chromosome	Query Start Base	Outg Gene ID	Outg Ext ID	Outg Chromosome	Outg Start Base
6385	ENSG00000079739	PGM1	Hsa1	63831535	205366	e_gw.26.99.1	BflBf_V2_215	1524772
5487	ENSG00000198658	Q5RGM9_HUMAN	Hsa1	144787932	112094	fgenesh2_pg.scaffold_1036000002	Bflscaffold_1036	21125
5487	ENSG00000203835	FAM108A2	Hsa1	146084969	112094	fgenesh2_pg.scaffold_1036000002	Bflscaffold_1036	21125
1322	ENSG00000143412	ANXA9	Hsa1	149221178	282940	estExt_gwp.C_2780035	Bflscaffold_278	411470
1026	ENSG00000143363	PRUNE	Hsa1	149247577	239767	e_gw.373.23.1	BflBf_V2_88	1731434
919	ENSG00000143398	PIP5K1A	Hsa1	149437651	216408	e_gw.66.2.1	BflBf_V2_216	1914305
5487	ENSG00000203704	648359	Hsa1	212845423	112094	fgenesh2_pg.scaffold_1036000002	Bflscaffold_1036	21125
5895	ENSG00000128918	ALDH1A2	Hsa15	56032920	113974	estExt_fgenesh2_pm.C_310003	BflBf_V2_204	475020
1857	ENSG00000140297	GCNT3	Hsa15	57691415	138210	gw.347.7.1	BflBf_V2_161	1280693
4726	ENSG00000069667	RORA	Hsa15	58576755	174225	gw.311.78.1	BflBf_V2_153	627125
717	ENSG00000086666	ZFAND6	Hsa15	78139076	226485	e_gw.237.119.1	BflBf_V2_106	147212
5487	ENSG00000136379	Q6PCB6_HUMAN	Hsa15	78774737	112094	fgenesh2_pg.scaffold_1036000002	Bflscaffold_1036	21125
2220	ENSG00000188869	TMC3	Hsa15	79417038	199456	e_gw.3.131.1	BflBf_V2_192	814159
5895	ENSG00000184254	ALDH1A3	Hsa15	99237580	113974	estExt_fgenesh2_pm.C_310003	BflBf_V2_204	475020
5487	ENSG00000129968	FAM108A1	Hsa19	1827976	112094	fgenesh2_pg.scaffold_1036000002	Bflscaffold_1036	21125
919	ENSG00000186111	PIP5K1C	Hsa19	3581182	216408	e_gw.66.2.1	BflBf_V2_216	1914305
763	ENSG00000077009	ITGB1BP3	Hsa19	3884101	128651	estExt_fgenesh2_pg.C_3240012	BflBf_V2_169	576995
6377	ENSG00000072062	PRKACA	Hsa19	14063509	119299	estExt_fgenesh2_pg.C_260217	Bflscaffold_26	3501662
1322	ENSG00000196975	ANXA4	Hsa2	69801427	282940	estExt_gwp.C_2780035	Bflscaffold_278	411470
2314	ENSG00000136682	CBWD2	Hsa2	113911853	93862	fgenesh2_pg.scaffold_244000068	BflBf_V2_113	844459
2220	ENSG00000149488	TMC2	Hsa20	2465253	199456	e_gw.3.131.1	BflBf_V2_192	814159
1857	ENSG00000124091	Q9HCV8_HUMAN	Hsa20	54499955	138210	gw.347.7.1	BflBf_V2_161	1280693
5487	ENSG00000182556		Hsa22	19352115	112094	fgenesh2_pg.scaffold_1036000002	Bflscaffold_1036	21125
5487	ENSG00000182502	150207	Hsa22	20799236	112094	fgenesh2_pg.scaffold_1036000002	Bflscaffold_1036	21125
1322	ENSG00000109511	ANXA10	Hsa4	169250310	282940	estExt_gwp.C_2780035	Bflscaffold_278	411470
1857	ENSG00000176928	GCNT4	Hsa5	74360257	138210	gw.347.7.1	BflBf_V2_161	1280693
919	ENSG00000173780	Q8TBY6_HUMAN	Hsa6	7931335	216408	e_gw.66.2.1	BflBf_V2_216	1914305
1857	ENSG00000111846	GCNT2	Hsa6	10636575	138210	gw.347.7.1	BflBf_V2_161	1280693
1857	ENSG00000205318	Q5T4J0_HUMAN	Hsa6	10742144	138210	gw.347.7.1	BflBf_V2_161	1280693
1322	ENSG00000104537	ANXA13	Hsa8	124762216	282940	estExt_gwp.C_2780035	Bflscaffold_278	411470
2314	ENSG00000147996	CBWD1	Hsa9	111039	93862	fgenesh2_pg.scaffold_244000068	BflBf_V2_113	844459
625	ENSG00000204827	Q6ZQT2_HUMAN	Hsa9	42787521	241679	e_gw.400.53.1	BflBf_V2_287	579876
6385	ENSG00000204794	Q5VTQ8_HUMAN	Hsa9	68155593	205366	e_gw.26.99.1	BflBf_V2_215	1524772
2314	ENSG00000204790	Q4V339_HUMAN	Hsa9	68494355	93862	fgenesh2_pg.scaffold_244000068	BflBf_V2_113	844459
625	ENSG00000204781	Q6ZQT2_HUMAN	Hsa9	69387663	241679	e_gw.400.53.1	BflBf_V2_287	579876
625	ENSG00000204777	Q6ZQT2_HUMAN	Hsa9	69588393	241679	e_gw.400.53.1	BflBf_V2_287	579876
2314	ENSG00000172785	CBWD5	Hsa9	69671821	93862	fgenesh2_pg.scaffold_244000068	BflBf_V2_113	844459
2314	ENSG00000196873	CBWD6	Hsa9	70046217	93862	fgenesh2_pg.scaffold_244000068	BflBf_V2_113	844459
6385	ENSG00000154330	PGM5	Hsa9	70161635	205366	e_gw.26.99.1	BflBf_V2_215	1524772
919	ENSG00000107242	PIP5K1B	Hsa9	70510436	216408	e_gw.66.2.1	BflBf_V2_216	1914305
6377	ENSG00000165059	PRKACG	Hsa9	70817241	119299	estExt_fgenesh2_pg.C_260217	Bflscaffold_26	3501662
5879	ENSG00000165060	FXN	Hsa9	70839995	250265	e_gw.560.8.1	Bflscaffold_560	360464
713	ENSG00000119139	TJP2	Hsa9	70956382	93411	fgenesh2_pg.scaffold_237000056	BflBf_V2_106	202014
554	ENSG00000107282	APBA1	Hsa9	71235022	123207	estExt_fgenesh2_pg.C_1130066	Bflscaffold_113	1415087
1441	ENSG00000188647	PTAR1	Hsa9	71514261	217651	e_gw.71.75.1	BflBf_V2_297	43735
3745	ENSG00000204711	C9orf135	Hsa9	71625529	123532	estExt_fgenesh2_pg.C_1220055	BflBf_V2_210	918324
1564	ENSG00000198887	SMC5	Hsa9	72063757	286107	estExt_gwp.C_4210064	Bflscaffold_421	601256
5473	ENSG00000135048	TMEM2	Hsa9	73488102	102620	fgenesh2_pg.scaffold_413000026	BflBf_V2_283	556686
5487	ENSG00000107362	C9orf77	Hsa9	73667188	112094	fgenesh2_pg.scaffold_1036000002	Bflscaffold_1036	21125
6592	ENSG00000119125	GDA	Hsa9	73954113	116233	estExt_fgenesh2_pm.C_4520006	BflBf_V2_88	1242900
717	ENSG00000107372	ZFAND5	Hsa9	74159194	226485	e_gw.237.119.1	BflBf_V2_106	147212
2220	ENSG00000165091	TMC1	Hsa9	74326537	199456	e_gw.3.131.1	BflBf_V2_192	814159
5895	ENSG00000165092	ALDH1A1	Hsa9	74705408	113974	estExt_fgenesh2_pm.C_310003	BflBf_V2_204	475020
1322	ENSG00000135046	ANXA1	Hsa9	74956493	282940	estExt_gwp.C_2780035	Bflscaffold_278	411470
4726	ENSG00000198963	RORB	Hsa9	76302072	174225	gw.311.78.1	BflBf_V2_153	627125
909	ENSG00000156017	C9orf41	Hsa9	76785754	76676	fgenesh2_pg.scaffold_66000001	BflBf_V2_216	5424
763	ENSG00000106733	C9orf95	Hsa9	76865309	128651	estExt_fgenesh2_pg.C_3240012	BflBf_V2_169	576995
1440	ENSG00000134996	OSTF1	Hsa9	76893210	279820	estExt_gwp.C_710036	Bflscaffold_71	591563
5079	ENSG00000189225		Hsa9	77222244	238706	e_gw.359.76.1	BflBf_V2_177	557744
4725	ENSG00000135002	RFK	Hsa9	78190253	283709	estExt_gwp.C_3110085	Bflscaffold_311	788545
1857	ENSG00000187210	GCNT1	Hsa9	78263966	138210	gw.347.7.1	BflBf_V2_161	1280693
1026	ENSG00000156035	C9orf65	Hsa9	78593080	239767	e_gw.373.23.1	BflBf_V2_88	1731434
715	ENSG00000197969	VPS13A	Hsa9	78982181	226506	e_gw.237.138.1	BflBf_V2_106	18024
6377	ENSG00000183943	PRKX	HsaX	3532411	119299	estExt_fgenesh2_pg.C_260217	Bflscaffold_26	3501662

Genomic distribution of the paralog groups of the surrounding 10-Mb window including the ALDH1A1, using the Ciona intestinalis genomic database as the outgroup for the best rec								
Genomic regiona analyzed: human chromosome 9 (69.000 KB - 79.000 KB)								
Group ID	Query Gene ID	Query Ext ID	Query Chromosome	Query Start Base	Outg Gene ID	Outg Ext ID	Outg Chromosome	Outg Start Base
1327	ENSG00000079739	PGM1	Hsa1	63831535	ENSCING00000001122		Cinscaffold_71	368590
3524	ENSG00000142875	PRKACB	Hsa1	84316329	ENSCING00000006522		Cin8q	3989626
2801	ENSG00000198658	Q5RGM9_HUMAN	Hsa1	144787932	ENSCING00000008539		Cin2q	4528155
2801	ENSG00000203835	FAM108A2	Hsa1	146084969	ENSCING00000008539		Cin2q	4528155
4661	ENSG00000143412	ANXA9	Hsa1	149221178	ENSCING00000004236		Cin9q	155680
2420	ENSG00000143363	PRUNE	Hsa1	149247577	ENSCING00000003006		Cinscaffold_55	572929
4328	ENSG00000143398	PIP5K1A	Hsa1	149437651	ENSCING00000009558		Cin3q	1417104
2801	ENSG00000203704	648359	Hsa1	212845423	ENSCING00000008539		Cin2q	4528155
4661	ENSG00000150165	ANXA8L1	Hsa10	46577989	ENSCING00000004236		Cin9q	155680
4661	ENSG00000186807	ANXA8L2	Hsa10	47216942	ENSCING00000004236		Cin9q	155680
4661	ENSG00000165390	ANXA8	Hsa10	47875259	ENSCING00000004236		Cin9q	155680
4661	ENSG00000138279	ANXA7	Hsa10	74805209	ENSCING00000004236		Cin9q	155680
4661	ENSG00000122359	ANXA11	Hsa10	81900625	ENSCING00000004236		Cin9q	155680
4888	ENSG00000111275	ALDH2	Hsa12	110688729	ENSCING00000007853		Cin7q	3337864
4888	ENSG00000128918	ALDH1A2	Hsa15	56032920	ENSCING00000007853		Cin7q	3337864
3225	ENSG00000140297	GCNT3	Hsa15	57691415	ENSCING00000013219		Cin8q	4106459
4661	ENSG00000182718	ANXA2	Hsa15	58426643	ENSCING00000004236		Cin9q	155680
1252	ENSG00000069667	RORA	Hsa15	58576755	ENSCING00000002152	Q4H2V2_CIOIN	Cin3p	1346821
434	ENSG00000129003	VPS13C	Hsa15	59931884	ENSCING00000001843		Cin1p	4970219
1274	ENSG00000086666	ZFAND6	Hsa15	78139076	ENSCING00000004420		Cin3p	536621
2801	ENSG00000136379	Q6PCB6_HUMAN	Hsa15	78774737	ENSCING00000008539		Cin2q	4528155
2255	ENSG00000188869	TMC3	Hsa15	79417038	ENSCING00000000728		Cin14p	347093
2317	ENSG00000140564	FURIN	Hsa15	89212889	ENSCING00000005033		Cinscaffold_152	114187
4888	ENSG00000184254	ALDH1A3	Hsa15	99237580	ENSCING00000007853		Cin7q	3337864
2317	ENSG00000140479	PCSK6	Hsa15	99661657	ENSCING00000005033		Cinscaffold_152	114187
2317	ENSG00000115257	PCSK4	Hsa19	1432427	ENSCING00000005033		Cinscaffold_152	114187
2801	ENSG00000129968	FAM108A1	Hsa19	1827976	ENSCING00000008539		Cin2q	4528155
4328	ENSG00000186111	PIP5K1C	Hsa19	3581182	ENSCING00000009558		Cin3q	1417104
3524	ENSG00000072062	PRKACA	Hsa19	14063509	ENSCING00000006522		Cin8q	3989626
4661	ENSG00000196975	ANXA4	Hsa2	69801427	ENSCING00000004236		Cin9q	155680
2255	ENSG00000149488	TMC2	Hsa20	2465253	ENSCING00000000728		Cin14p	347093
3225	ENSG00000124091	Q9HCV8_HUMAN	Hsa20	54499955	ENSCING00000013219		Cin8q	4106459
2801	ENSG00000182556		Hsa22	19352115	ENSCING00000008539		Cin2q	4528155
2801	ENSG00000182502	150207	Hsa22	20799236	ENSCING00000008539		Cin2q	4528155
4661	ENSG00000138772	ANXA3	Hsa4	79694613	ENSCING00000004236		Cin9q	155680
4661	ENSG00000164111	ANXA5	Hsa4	122808598	ENSCING00000004236		Cin9q	155680
4661	ENSG00000109511	ANXA10	Hsa4	169250310	ENSCING00000004236		Cin9q	155680
3225	ENSG00000176928	GCNT4	Hsa5	74360257	ENSCING00000013219		Cin8q	4106459
2317	ENSG00000175426	PCSK1	Hsa5	95751875	ENSCING00000005033		Cinscaffold_152	114187
4328	ENSG00000173780	Q8TBY6_HUMAN	Hsa6	7931335	ENSCING00000009558		Cin3q	1417104
3225	ENSG00000111846	GCNT2	Hsa6	10636575	ENSCING00000013219		Cin8q	4106459
3225	ENSG00000205318	Q5T4J0_HUMAN	Hsa6	10742144	ENSCING00000013219		Cin8q	4106459
1274	ENSG00000156639	ZFAND3	Hsa6	37895285	ENSCING00000004420		Cin3p	536621
4661	ENSG00000104537	ANXA13	Hsa8	124762216	ENSCING00000004236		Cin9q	155680
4661	ENSG00000183059	ANXA2P2	Hsa9	33614223	ENSCING00000004236		Cin9q	155680
4888	ENSG00000137124	ALDH1B1	Hsa9	38382661	ENSCING00000007853		Cin7q	3337864
1327	ENSG00000204794	Q5VTQ8_HUMAN	Hsa9	68155593	ENSCING00000001122		Cinscaffold_71	368590
1327	ENSG00000154330	PGM5	Hsa9	70161635	ENSCING00000001122		Cinscaffold_71	368590
4328	ENSG00000107242	PIP5K1B	Hsa9	70510436	ENSCING00000009558		Cin3q	1417104
3524	ENSG00000165059	PRKACG	Hsa9	70817241	ENSCING00000006522		Cin8q	3989626
1681	ENSG00000135063	C9orf61	Hsa9	71130168	ENSCING00000007086		Cin1q	3177789
2938	ENSG00000204711	C9orf135	Hsa9	71625529	ENSCING00000003931		Cin12q	1801648
863	ENSG00000165072	MAMDC2	Hsa9	71848317	ENSCING00000000241		Cin4q	125019
1997	ENSG00000198887	SMC5	Hsa9	72063757	ENSCING00000008151		Cin12p	1765299
2801	ENSG00000107362	C9orf77	Hsa9	73667188	ENSCING00000008539		Cin2q	4528155
1274	ENSG00000107372	ZFAND5	Hsa9	74159194	ENSCING00000004420		Cin3p	536621
2255	ENSG00000165091	TMC1	Hsa9	74326537	ENSCING00000000728		Cin14p	347093
4888	ENSG00000165092	ALDH1A1	Hsa9	74705408	ENSCING00000007853		Cin7q	3337864
4661	ENSG00000135046	ANXA1	Hsa9	74956493	ENSCING00000004236		Cin9q	155680
1252	ENSG00000198963	RORB	Hsa9	76302072	ENSCING00000002152	Q4H2V2_CIOIN	Cin3p	1346821
1387	ENSG00000156017	C9orf41	Hsa9	76785754	ENSCING00000003355		Cinscaffold_69	362208
4261	ENSG00000134996	OSTF1	Hsa9	76893210	ENSCING00000004989		Cin3q	3586388
2317	ENSG00000099139	PCSK5	Hsa9	77695406	ENSCING00000005033		Cinscaffold_152	114187
3225	ENSG00000187210	GCNT1	Hsa9	78263966	ENSCING00000013219		Cin8q	4106459
2418	ENSG00000106772	KIAA0367	Hsa9	78419138	ENSCING00000016431		Cinscaffold_55	562345
2420	ENSG00000156035	C9orf65	Hsa9	78593080	ENSCING00000003006		Cinscaffold_55	572929
434	ENSG00000197969	VPS13A	Hsa9	78982181	ENSCING00000001843		Cin1p	4970219
863	ENSG00000177943	MAMDC4	Hsa9	138866640	ENSCING00000000241		Cin4q	125019
3524	ENSG00000183943	PRKX	HsaX	3532411	ENSCING00000006522		Cin8q	3989626
3524	ENSG00000099725	PRKY	HsaY	7202013	ENSCING00000006522		Cin8q	3989626

Genomic distribution of the paralog groups of the surrounding 10-Mb window including the ALDH1A2, using the Branchiostoma floridae genomic database as the outgroup for the best reciprocal BLAST hit analysis									
Genomic regiona analyzed: human chromosome 15 (51.000 KB - 61.000 KB)									
Group ID	Query Gene ID	Query Ext ID	Query Chromosome	Query Start Base	Outg Gene ID	Outg Ext ID	Outg Chromosome	Outg Start Base	
2362	ENSG00000172380	GNG12	Hsa1	67939737	251807	e_gw.595.39.1	Bflscaffold_595	232299	
1027	ENSG00000163141	BNIPL	Hsa1	149275670	239803	e_gw.373.4.1	BflBf_V2_88	1754013	
4828	ENSG00000143595	AQP10	Hsa1	152560180	288680	estExt_gwp.C_6430015	Bflscaffold_643	275173	
2362	ENSG00000168243	GNG4	Hsa1	233780755	251807	e_gw.595.39.1	Bflscaffold_595	232299	
1610	ENSG00000176244	ACBD7	Hsa10	15157481	98944	fgenesh2_pg.scaffold_332000020	BflBf_V2_118	211011	
494	ENSG00000138279	ANXA7	Hsa10	74805209	120055	estExt_fgenesh2_pg.C_380096	BflBf_V2_27	1632320	
5139	ENSG00000134333	LDHA	Hsa11	18372683	114460	estExt_fgenesh2_pm.C_770009	BflBf_V2_162	1612550	
5139	ENSG00000166796	LDHC	Hsa11	18390429	114460	estExt_fgenesh2_pm.C_770009	BflBf_V2_162	1612550	
5139	ENSG00000166800	LDHAL6A	Hsa11	18434007	114460	estExt_fgenesh2_pm.C_770009	BflBf_V2_162	1612550	
2362	ENSG00000162188	GNG3	Hsa11	62231706	251807	e_gw.595.39.1	Bflscaffold_595	232299	
5139	ENSG00000111716	LDHB	Hsa12	21679543	114460	estExt_fgenesh2_pm.C_770009	BflBf_V2_162	1612550	
5139	ENSG00000178001		Hsa12	61683600	114460	estExt_fgenesh2_pm.C_770009	BflBf_V2_162	1612550	
5994	ENSG00000133101	CCNA1	Hsa13	35904495	69715	fgenesh2_pg.scaffold_24000220	BflBf_V2_150	3731640	
2362	ENSG00000186469	GNG2	Hsa14	51383702	251807	e_gw.595.39.1	Bflscaffold_595	232299	
5984	ENSG00000205871	648659	Hsa15	41195189	69722	fgenesh2_pg.scaffold_24000227	BflBf_V2_150	3873865	
1139	ENSG00000166415	WDR72	Hsa15	51594673	131766	estExt_fgenesh2_pg.C_5820012	Bflscaffold_582	246963	
6857	ENSG00000137766	UNC13C	Hsa15	52314521	102486	fgenesh2_pg.scaffold_410000031	BflBf_V2_32	3039460	
3399	ENSG00000137876	C15orf15	Hsa15	53260813	273508	estExt_GenewiseH_1.C_6810009	BflBf_V2_321	187556	
716	ENSG00000069974	RAB27A	Hsa15	53283094	281741	estExt_gwp.C_2370096	BflBf_V2_106	137537	
2072	ENSG00000069943	PIGB	Hsa15	53398741	273190	estExt_GenewiseH_1.C_6290019	Bflscaffold_629	123619	
1655	ENSG00000205548		Hsa15	53488038	119941	estExt_fgenesh2_pg.C_370052	Bflscaffold_37	924956	
917	ENSG00000166450	PRTG	Hsa15	53699353	216524	e_gw.66.57.1	BflBf_V2_216	1379900	
864	ENSG00000166466	440280	Hsa15	54085203	130644	estExt_fgenesh2_pg.C_4510011	BflBf_V2_108	39666	
1216	ENSG00000151575	TEX9	Hsa15	54444936	275559	estExt_gwp.C_60178	BflBf_V2_187	5778078	
6573	ENSG00000138587	MNS1	Hsa15	54508299	126810	estExt_fgenesh2_pg.C_2300048	Bflscaffold_230	1093406	
1610	ENSG00000140238	650875	Hsa15	54925830	98944	fgenesh2_pg.scaffold_332000020	BflBf_V2_118	211011	
5895	ENSG00000128918	ALDH1A2	Hsa15	56032920	113974	estExt_fgenesh2_pm.C_310003	BflBf_V2_204	475020	
4828	ENSG00000103569	AQP9	Hsa15	56217771	288680	estExt_gwp.C_6430015	Bflscaffold_643	275173	
2176	ENSG00000137845	ADAM10	Hsa15	56675802	247442	e_gw.491.21.1	Bflscaffold_491	178549	
2362	ENSG00000182625		Hsa15	56765902	251807	e_gw.595.39.1	Bflscaffold_595	232299	
3721	ENSG00000137776	SLTM	Hsa15	56958537	129391	estExt_fgenesh2_pg.C_3630023	Bflscaffold_363	462718	
5994	ENSG00000157456	CCNB2	Hsa15	57184612	69715	fgenesh2_pg.scaffold_24000220	BflBf_V2_150	3731640	
2491	ENSG00000157483	MYO1E	Hsa15	57215461	219333	e_gw.78.36.1	BflBf_V2_32	3765987	
5139	ENSG00000171989	LDHAL6B	Hsa15	57286314	114460	estExt_fgenesh2_pm.C_770009	BflBf_V2_162	1612550	
757	ENSG00000157470	FAM81A	Hsa15	57517664	98567	fgenesh2_pg.scaffold_324000044	BflBf_V2_169	144465	
1857	ENSG00000140297	GCNT3	Hsa15	57691415	138210	gw.347.7.1	BflBf_V2_161	1280693	
1027	ENSG00000140299	BNIP2	Hsa15	57742356	239803	e_gw.373.4.1	BflBf_V2_88	1754013	
5984	ENSG00000187899		Hsa15	57847843	69722	fgenesh2_pg.scaffold_24000227	BflBf_V2_150	3873865	
494	ENSG00000182718	ANXA2	Hsa15	58426643	120055	estExt_fgenesh2_pg.C_380096	BflBf_V2_27	1632320	
3417	ENSG00000128915	NARG2	Hsa15	58499100	92061	fgenesh2_pg.scaffold_217000051	Bflscaffold_217	969087	
4726	ENSG00000069667	RORA	Hsa15	58576755	174225	gw.311.78.1	BflBf_V2_153	627125	
2734	ENSG00000198535	NP_997205.1	Hsa15	60146506	109545	fgenesh2_pg.scaffold_694000001	BflBf_V2_323	1450	
2393	ENSG00000171914	TLN2	Hsa15	60726802	129467	estExt_fgenesh2_pg.C_3680027	Bflscaffold_368	623076	
917	ENSG00000174498	PUNC	Hsa15	63407842	216524	e_gw.66.57.1	BflBf_V2_216	1379900	
917	ENSG00000103742	NP_066013.1	Hsa15	63460878	216524	e_gw.66.57.1	BflBf_V2_216	1379900	
5895	ENSG00000184254	ALDH1A3	Hsa15	99237580	113974	estExt_fgenesh2_pm.C_310003	BflBf_V2_204	475020	
2362	ENSG00000167083	GNGT2	Hsa17	44638596	251807	e_gw.595.39.1	Bflscaffold_595	232299	
716	ENSG00000041353	RAB27B	Hsa18	50646706	281741	estExt_gwp.C_2370096	BflBf_V2_106	137537	
1139	ENSG00000091157	WDR7	Hsa18	52469614	131766	estExt_fgenesh2_pg.C_5820012	Bflscaffold_582	246963	
3399	ENSG00000141650	284288	Hsa18	53656706	273508	estExt_GenewiseH_1.C_6810009	BflBf_V2_321	187556	
2362	ENSG00000176533	GNG7	Hsa19	2462218	251807	e_gw.595.39.1	Bflscaffold_595	232299	
1027	ENSG00000167654	ATCAY	Hsa19	3831672	239803	e_gw.373.4.1	BflBf_V2_88	1754013	
2491	ENSG00000142347	MYO1F	Hsa19	8491689	219333	e_gw.78.36.1	BflBf_V2_32	3765987	
6857	ENSG00000130477	UNC13A	Hsa19	17573167	102486	fgenesh2_pg.scaffold_410000031	BflBf_V2_32	3039460	
2362	ENSG00000167414	GNG8	Hsa19	51829173	251807	e_gw.595.39.1	Bflscaffold_595	232299	
1610	ENSG00000155368	DBI	Hsa2	119841055	98944	fgenesh2_pg.scaffold_332000020	BflBf_V2_118	211011	
1857	ENSG00000124091	Q9HCV8_HUMAN	Hsa20	54499955	138210	gw.347.7.1	BflBf_V2_161	1280693	
3399	ENSG00000114391	RPL24	Hsa3	102882626	273508	estExt_GenewiseH_1.C_6810009	BflBf_V2_321	187556	
864	ENSG00000138767	CNOT6L	Hsa4	78857199	130644	estExt_fgenesh2_pg.C_4510011	BflBf_V2_108	39666	
494	ENSG00000138772	ANXA3	Hsa4	79694613	120055	estExt_fgenesh2_pg.C_380096	BflBf_V2_27	1632320	
494	ENSG00000164111	ANXA5	Hsa4	122808598	120055	estExt_fgenesh2_pg.C_380096	BflBf_V2_27	1632320	
5994	ENSG00000145386	CCNA2	Hsa4	122958002	69715	fgenesh2_pg.scaffold_24000220	BflBf_V2_150	3731640	
5984	ENSG00000145425	RPS3A	Hsa4	152240204	69722	fgenesh2_pg.scaffold_24000227	BflBf_V2_150	3873865	
5984	ENSG00000178429	XR_017186.1	Hsa4	153691862	69722	fgenesh2_pg.scaffold_24000227	BflBf_V2_150	3873865	
5994	ENSG00000134057	CCNB1	Hsa5	68498593	69715	fgenesh2_pg.scaffold_24000220	BflBf_V2_150	3731640	
1857	ENSG00000176928	GCNT4	Hsa5	74360257	138210	gw.347.7.1	BflBf_V2_161	1280693	
864	ENSG00000113300	CNOT6	Hsa5	179854023	130644	estExt_fgenesh2_pg.C_4510011	BflBf_V2_108	39666	
1857	ENSG00000111846	GCNT2	Hsa6	10636575	138210	gw.347.7.1	BflBf_V2_161	1280693	
1857	ENSG00000205318	Q5T4J0_HUMAN	Hsa6	10742144	138210	gw.347.7.1	BflBf_V2_161	1280693	
3399	ENSG00000181524	Q5T8W0_HUMAN	Hsa6	43032061	273508	estExt_GenewiseH_1.C_6810009	BflBf_V2_321	187556	
5139	ENSG00000146701	MDH2	Hsa7	75515328	114460	estExt_fgenesh2_pm.C_770009	BflBf_V2_162	1612550	
2362	ENSG00000127928	GNGT1	Hsa7	93373756	251807	e_gw.595.39.1	Bflscaffold_595	232299	
2362	ENSG00000127920	GNG11	Hsa7	93388974	251807	e_gw.595.39.1	Bflscaffold_595	232299	
4828	ENSG00000165269	AQP7	Hsa9	33374948	288680	estExt_gwp.C_6430015	Bflscaffold_643	275173	
4828	ENSG00000165272	AQP3	Hsa9	33431152	288680	estExt_gwp.C_6430015	Bflscaffold_643	275173	
6857	ENSG00000198722	UNC13B	Hsa9	35151999	102486	fgenesh2_pg.scaffold_410000031	BflBf_V2_32	3039460	
2393	ENSG00000137076	TLN1	Hsa9	35687336	129467	estExt_fgenesh2_pg.C_3680027	Bflscaffold_368	623076	
5895	ENSG00000165092	ALDH1A1	Hsa9	74705408	113974	estExt_fgenesh2_pm.C_310003	BflBf_V2_204	475020	
4726	ENSG00000198963	RORB	Hsa9	76302072	174225	gw.311.78.1	BflBf_V2_153	627125	
1857	ENSG00000187210	GCNT1	Hsa9	78263966	138210	gw.347.7.1	BflBf_V2_161	1280693	

Genomic distribution of the paralog groups of the surrounding 10-Mb window including the ALDH1A2, using the Ciona intestinalis genomic database as the outgroup for the best reciprocal BLAST hit analysis								
Genomic regiona analyzed: human chromosome 15 (51.000 KB - 61.000 KB)								
Group ID	Query Gene ID	Query Ext ID	Query Chromosome	Query Start Base	Outg Gene ID	Outg Ext ID	Outg Chromosome	Outg Start Base
4661	ENSG00000143412	ANXA9	Hsa1	149221178	ENSCING00000004236		Cin9q	4106459
2422	ENSG00000163141	BNIPL	Hsa1	149275670	ENSCING00000016431		Cinscaffold_55	1071429
4502	ENSG00000143595	AQP10	Hsa1	152560180	ENSCING00000009588		Cin9q	2974363
4661	ENSG00000150165	ANXA8L1	Hsa10	46577989	ENSCING00000004236		Cin9q	4106459
4661	ENSG00000186807	ANXA8L2	Hsa10	47216942	ENSCING00000004236		Cin9q	3805745
4661	ENSG00000165390	ANXA8	Hsa10	47875259	ENSCING00000004236		Cin9q	3910900
4661	ENSG00000138279	ANXA7	Hsa10	74805209	ENSCING00000004236		Cin9q	155680
4661	ENSG00000122359	ANXA11	Hsa10	81900625	ENSCING00000004236		Cin9q	2688529
1033	ENSG00000134333	LDHA	Hsa11	18372683	ENSCING00000003426		Cin14q	294557
1033	ENSG00000166796	LDHC	Hsa11	18390429	ENSCING00000003426		Cin14q	294557
1033	ENSG00000166800	LDHAL6A	Hsa11	18434007	ENSCING00000003426		Cin14q	294557
1033	ENSG00000111716	LDHB	Hsa12	21679543	ENSCING00000003426		Cin14q	294557
1033	ENSG00000178001		Hsa12	61683600	ENSCING00000003426		Cin14q	294557
4888	ENSG00000111275	ALDH2	Hsa12	110688729	ENSCING00000007853		Cin7q	3337864
2520	ENSG00000133101	CCNA1	Hsa13	35904495	ENSCING00000008478		Cin2q	1882180
4276	ENSG00000080824	HSP90AA1	Hsa14	101617139	ENSCING00000012576		Cin3q	4106459
2514	ENSG00000205871	648659	Hsa15	41195189	ENSCING00000005199		Cin2q	3910900
3348	ENSG00000166415	WDR72	Hsa15	51594673	ENSCING00000007297		Cin8q	4259777
1174	ENSG00000137766	UNC13C	Hsa15	52314521	ENSCING00000002173		Cin3p	1882180
2247	ENSG00000137876	C15orf15	Hsa15	53260813	ENSCING00000005865		Cin14p	967701
841	ENSG00000069974	RAB27A	Hsa15	53283094	ENSCING00000007626		Cin4q	155680
2023	ENSG00000069943	PIGB	Hsa15	53398741	ENSCING00000008140		Cin12p	3197110
3709	ENSG00000128845	CCPG1	Hsa15	53434741	ENSCING00000002889	NP_001027805	Cin9p	562345
962	ENSG00000205548		Hsa15	53488038	ENSCING00000017702		Cinscaffold_382	155680
2271	ENSG00000166466	440280	Hsa15	54085203	ENSCING00000006911		Cinscaffold_199	51330
576	ENSG00000151575	TEX9	Hsa15	54444936	ENSCING00000017439		Cinscaffold_966	155680
2201	ENSG00000138587	MNS1	Hsa15	54508299	ENSCING00000014568		Cin14p	2166750
4342	ENSG00000140262	TCF12	Hsa15	54998125	ENSCING00000009523	Q4H3N7_CIOIN	Cin3q	3805745
4888	ENSG00000128918	ALDH1A2	Hsa15	56032920	ENSCING00000007853		Cin7q	155680
4502	ENSG00000103569	AQP9	Hsa15	56217771	ENSCING00000009588		Cin9q	2974363
3211	ENSG00000166035	LIPC	Hsa15	56511467	ENSCING00000006474		Cin8q	2974363
4211	ENSG00000137845	ADAM10	Hsa15	56675802	ENSCING00000009495		Cin3q	155680
4276	ENSG00000205527	HSP90AB4P	Hsa15	56770594	ENSCING00000012576		Cin3q	3197110
3371	ENSG00000137776	SLTM	Hsa15	56958537	ENSCING00000003000		Cin8q	1071429
2520	ENSG00000157456	CCNB2	Hsa15	57184612	ENSCING00000008478		Cin2q	270342
7	ENSG00000157483	MYO1E	Hsa15	57215461	ENSCING00000003718		Cinscaffold_139	155680
1033	ENSG00000171989	LDHAL6B	Hsa15	57286314	ENSCING00000003426		Cin14q	3337864
3089	ENSG00000157470	FAM81A	Hsa15	57517664	ENSCING00000004867		Cinscaffold_36	4106459
3225	ENSG00000140297	GCNT3	Hsa15	57691415	ENSCING00000013219		Cin8q	4970219
2422	ENSG00000140299	BNIP2	Hsa15	57742356	ENSCING00000016431		Cinscaffold_55	3337864
2514	ENSG00000187899		Hsa15	57847843	ENSCING00000005199		Cin2q	772640
4661	ENSG00000182718	ANXA2	Hsa15	58426643	ENSCING00000004236		Cin9q	2688529
1252	ENSG00000069667	RORA	Hsa15	58576755	ENSCING00000002152	Q4H2V2_CIOIN	Cin3p	4259777
434	ENSG00000129003	VPS13C	Hsa15	59931884	ENSCING00000001843		Cin1p	2688529
4207	ENSG00000171914	TLN2	Hsa15	60726802	ENSCING00000009522		Cin3q	270342
4888	ENSG00000184254	ALDH1A3	Hsa15	99237580	ENSCING00000007853		Cin7q	1346821
3211	ENSG00000101670	LIPG	Hsa18	45342425	ENSCING00000006474		Cin8q	155680
841	ENSG00000041353	RAB27B	Hsa18	50646706	ENSCING00000007626		Cin4q	155680
4342	ENSG00000196628	TCF4	Hsa18	51046093	ENSCING00000009523	Q4H3N7_CIOIN	Cin3q	155680
3348	ENSG00000091157	WDR7	Hsa18	52469614	ENSCING00000007297		Cin8q	3197110
2247	ENSG00000141650	284288	Hsa18	53656706	ENSCING00000005865		Cin14p	3900975
4342	ENSG00000071564	TCF3	Hsa19	1561964	ENSCING00000009523	Q4H3N7_CIOIN	Cin3q	1882180
2422	ENSG00000167654	ATCAY	Hsa19	3831672	ENSCING00000016431		Cinscaffold_55	2688529
7	ENSG00000142347	MYO1F	Hsa19	8491689	ENSCING00000003718		Cinscaffold_139	562345
1174	ENSG00000130477	UNC13A	Hsa19	17573167	ENSCING00000002173		Cin3p	3805745
4661	ENSG00000196975	ANXA4	Hsa2	69801427	ENSCING00000004236		Cin9q	155680
3225	ENSG00000124091	Q9HCV8_HUMAN	Hsa20	54499955	ENSCING00000013219		Cin8q	1044744
2247	ENSG00000114391	RPL24	Hsa3	102882626	ENSCING00000005865		Cin14p	9120
2271	ENSG00000138767	CNOT6L	Hsa4	78857199	ENSCING00000006911		Cinscaffold_199	9587
4661	ENSG00000138772	ANXA3	Hsa4	79694613	ENSCING00000004236		Cin9q	1457745
4276	ENSG00000183199	Q58FF7_HUMAN	Hsa4	89032019	ENSCING00000012576		Cin3q	51330
4661	ENSG00000164111	ANXA5	Hsa4	122808598	ENSCING00000004236		Cin9q	1044744
2520	ENSG00000145386	CCNA2	Hsa4	122958002	ENSCING00000008478		Cin2q	294557
2514	ENSG00000145425	RPS3A	Hsa4	152240204	ENSCING00000005199		Cin2q	3805745
2514	ENSG00000178429	XR_017186.1	Hsa4	153691882	ENSCING00000005199		Cin2q	7421351
4661	ENSG00000109511	ANXA10	Hsa4	169250310	ENSCING00000004236		Cin9q	3337864
2520	ENSG00000134057	CCNB1	Hsa5	68498593	ENSCING00000008478		Cin2q	107149
3225	ENSG00000176928	GCNT4	Hsa5	74360257	ENSCING00000013219		Cin8q	3337864
3089	ENSG00000153347	FAM81B	Hsa5	94754247	ENSCING00000004867		Cinscaffold_36	562345
2271	ENSG00000113300	CNOT6	Hsa5	179854023	ENSCING00000006911		Cinscaffold_199	2546667
3225	ENSG00000111846	GCNT2	Hsa6	10636575	ENSCING00000013219		Cin8q	3910900
3225	ENSG00000205318	Q5T4J0_HUMAN	Hsa6	10742144	ENSCING00000013219		Cin8q	2166750
4276	ENSG00000096384	HSP90AB1	Hsa6	44322802	ENSCING00000012576		Cin3q	155680
3211	ENSG00000175445	LPL	Hsa8	19841232	ENSCING00000006474		Cin8q	1346821
4661	ENSG00000104537	ANXA13	Hsa8	124762216	ENSCING00000004236		Cin9q	2688529
4502	ENSG00000165269	AQP7	Hsa9	33374948	ENSCING00000009588		Cin9q	155680
4502	ENSG00000165272	AQP3	Hsa9	33431152	ENSCING00000009588		Cin9q	1882180
**4661**	**ENSG00000183059**	**ANXA2P2**	**Hsa9**	**33614223**	**ENSCING00000004236**		**Cin9q**	**2688529**
1174	ENSG00000198722	UNC13B	Hsa9	35151999	ENSCING00000002173		Cin3p	2974363
4207	ENSG00000137076	TLN1	Hsa9	35687336	ENSCING00000009522		Cin3q	1457745
4888	ENSG00000137124	ALDH1B1	Hsa9	38382661	ENSCING00000007853		Cin7q	4106459
4502	ENSG00000181997		Hsa9	42848068	ENSCING00000009588		Cin9q	4106459

4502	ENSG00000176115		Hsa9	66770881	ENSCING00000009588		Cin9q	107149
4502	ENSG00000186466	AQP7P2	Hsa9	66961803	ENSCING00000009588		Cin9q	51330
4888	ENSG00000165092	ALDH1A1	Hsa9	74705408	ENSCING00000007853		Cin7q	4970219
4661	ENSG00000135046	ANXA1	Hsa9	74956493	ENSCING00000004236		Cin9q	2688529
1252	ENSG00000198963	RORB	Hsa9	76302072	ENSCING00000002152	Q4H2V2_CIOIN	Cin3p	1457745
3225	ENSG00000187210	GCNT1	Hsa9	78263966	ENSCING00000013219		Cin8q	1071429
434	ENSG00000197969	VPS13A	Hsa9	78982181	ENSCING00000001843		Cin1p	155680

Genomic distribution of the paralog groups of the surrounding 10-Mb window including the ALDH1A3, using the Branchiostoma floridae genomic database as the outgroup for the best reciprocal BLAST hit analysis								
Genomic regiona analyzed: human chromosome 15 (90.000 KB - 100.000 KB)								
Group ID	Query Gene ID	Query Ext ID	Query Chromosome	Query Start Base	Outg Gene ID	Outg Ext ID	Outg Chromosome	Outg Start Base
562	ENSG00000168509	HFE2	Hsa1	144124628	82627	fgenesh2_pg.scaffold_113000060	BflBf_V2_119	1262645
5173	ENSG00000117289	TXNIP	Hsa1	144149826	118530	estExt_fgenesh2_pg.C_150153	BflBf_V2_65	4485499
6237	ENSG00000143418	LASS2	Hsa1	149204286	280654	estExt_gwp.C_860189	BflBf_V2_207	2065554
4777	ENSG00000027644	INSRR	Hsa1	155076479	128184	estExt_fgenesh2_pg.C_2960033	BflBf_V2_380	100742
1997	ENSG00000084453	SLCO1A2	Hsa12	21313094	126199	estExt_fgenesh2_pg.C_2070022	Bflscaffold_207	500002
6237	ENSG00000139624	LASS5	Hsa12	48809849	280654	estExt_gwp.C_860189	BflBf_V2_207	2065554
5895	ENSG00000128918	ALDH1A2	Hsa15	56032920	113974	estExt_fgenesh2_pm.C_310003	BflBf_V2_204	475020
1997	ENSG00000176463	SLCO3A1	Hsa15	90197950	126199	estExt_fgenesh2_pg.C_2070022	Bflscaffold_207	500002
4783	ENSG00000140557	ST8SIA2	Hsa15	90738144	247570	e_gw.494.65.1	BflBf_V2_32	7508176
2464	ENSG00000185442	NP_997329.1	Hsa15	90961685	122141	estExt_fgenesh2_pg.C_780166	BflBf_V2_32	5026527
1767	ENSG00000173575	CHD2	Hsa15	91244423	266878	estExt_GenewiseH_1.C_2530071	Bflscaffold_253	1057823
562	ENSG00000182175	RGMA	Hsa15	91387651	82627	fgenesh2_pg.scaffold_113000060	BflBf_V2_119	1262645
563	ENSG00000140563	MCTP2	Hsa15	92575955	82577	fgenesh2_pg.scaffold_113000010	BflBf_V2_119	107322
5173	ENSG00000140450	ARRDC4	Hsa15	96304947	118530	estExt_fgenesh2_pg.C_150153	BflBf_V2_65	4485499
4777	ENSG00000140443	IGF1R	Hsa15	97010302	128184	estExt_fgenesh2_pg.C_2960033	BflBf_V2_380	100742
5071	ENSG00000183571	145814	Hsa15	97328859	124319	estExt_fgenesh2_pg.C_1450040	BflBf_V2_123	701790
366	ENSG00000103852	TTC23	Hsa15	97494052	68857	fgenesh2_pg.scaffold_20000205	BflBf_V2_261	1506896
650	ENSG00000168904	LRRC28	Hsa15	97609175	59583	fgenesh2_pm.scaffold_146000015	BflBf_V2_32	6601306
5052	ENSG00000183060	LYSMD4	Hsa15	98073429	85789	fgenesh2_pg.scaffold_145000047	BflBf_V2_123	678098
6761	ENSG00000140470	ADAMTS17	Hsa15	98331993	104449	fgenesh2_pg.scaffold_463000032	Bflscaffold_463	278638
6237	ENSG00000154227	LASS3	Hsa15	98758124	280654	estExt_gwp.C_860189	BflBf_V2_207	2065554
6762	ENSG00000140471	LINS1	Hsa15	98926957	104462	fgenesh2_pg.scaffold_463000045	BflBf_V2_185	43996
686	ENSG00000183475	ASB7	Hsa15	98960337	85901	fgenesh2_pg.scaffold_146000049	BflBf_V2_32	6510385
5895	ENSG00000184254	ALDH1A3	Hsa15	99237580	113974	estExt_fgenesh2_pm.C_310003	BflBf_V2_204	475020
5058	ENSG00000131873	CHSY1	Hsa15	99533456	124328	estExt_fgenesh2_pg.C_1450053	BflBf_V2_123	588134
6679	ENSG00000131871	SELS_HUMAN	Hsa15	99628737	272122	estExt_GenewiseH_1.C_5160031	Bflscaffold_516	371510
3291	ENSG00000131876	SNRPA1	Hsa15	99639240	115963	estExt_fgenesh2_pm.C_3710008	BflBf_V2_69	1237349
5044	ENSG00000140479	PCSK6	Hsa15	99661657	124316	estExt_fgenesh2_pg.C_1450035	BflBf_V2_123	788680
5060	ENSG00000184277	TM2D3	Hsa15	99999580	85791	fgenesh2_pg.scaffold_145000049	BflBf_V2_123	651902
2464	ENSG00000118050	C19orf24	Hsa19	1226520	122141	estExt_fgenesh2_pg.C_780166	BflBf_V2_32	5026527
4777	ENSG00000171105	INSR	Hsa19	7067049	128184	estExt_fgenesh2_pg.C_2960033	BflBf_V2_380	100742
5173	ENSG00000105643	ARRDC2	Hsa19	17972944	118530	estExt_fgenesh2_pg.C_150153	BflBf_V2_65	4485499
5071	ENSG00000130517	PGPEP1	Hsa19	18312450	124319	estExt_fgenesh2_pg.C_1450040	BflBf_V2_123	701790
3291	ENSG00000144158	Q9NU36_HUMAN	Hsa2	114131955	115963	estExt_fgenesh2_pm.C_3710008	BflBf_V2_69	1237349
1997	ENSG00000174640	SLCO2A1	Hsa3	135134236	126199	estExt_fgenesh2_pg.C_2070022	Bflscaffold_207	500002
5052	ENSG00000176018	LYSMD3	Hsa5	89847203	85789	fgenesh2_pg.scaffold_145000047	BflBf_V2_123	678098
5173	ENSG00000113369	ARRDC3	Hsa5	90700299	118530	estExt_fgenesh2_pg.C_150153	BflBf_V2_65	4485499
563	ENSG00000175471	MCTP1	Hsa5	94068601	82577	fgenesh2_pg.scaffold_113000010	BflBf_V2_119	107322
562	ENSG00000174136	RGMB	Hsa5	98132900	82627	fgenesh2_pg.scaffold_113000060	BflBf_V2_119	1262645
1767	ENSG00000153922	CHD1	Hsa5	98218819	266878	estExt_GenewiseH_1.C_2530071	Bflscaffold_253	1057823
4783	ENSG00000113532	ST8SIA4	Hsa5	100170803	247570	e_gw.494.65.1	BflBf_V2_32	7508176
6761	ENSG00000145808	ADAMTS19	Hsa5	128824002	104449	fgenesh2_pg.scaffold_463000032	Bflscaffold_463	278638
5058	ENSG00000198108	CHSS3_HUMAN	Hsa5	129268422	124328	estExt_fgenesh2_pg.C_1450053	BflBf_V2_123	588134
5895	ENSG00000165092	ALDH1A1	Hsa9	74705408	113974	estExt_fgenesh2_pm.C_310003	BflBf_V2_204	475020

Genomic distribution of the paralog groups of the surrounding 10-Mb window including the ALDH1A3, using the Ciona intestinalis genomic database as the outgroup for the best reciprocal BLAST hit analysis

Genomic regiona analyzed: human chromosome 15 (90.000 KB - 100.000 KB)

Group ID	Query Gene ID	Query Ext ID	Query Chromosome	Query Start Base	Outg Gene ID	Outg Ext ID	Outg Chromosome	Outg Start Base
3075	ENSG00000168509	HFE2	Hsa1	144124628	ENSCING00000004900		Cinscaffold_36	161251
1234	ENSG00000143418	LASS2	Hsa1	149204286	ENSCING00000007135	Q4H395_CIOIN	Cin3p	1259787
1308	ENSG00000116604	MEF2D	Hsa1	154700143	ENSCING00000000628	Q4H375_CIOIN	Cinscaffold_187	72757
2606	ENSG00000148488	ST8SIA6	Hsa10	17400700	ENSCING00000008556	NP_001027703	Cin2q	4459681
3488	ENSG00000137491	SLCO2B1	Hsa11	74539809	ENSCING00000001637		Cin8q	7321733
2606	ENSG00000111728	ST8SIA1	Hsa12	22245204	ENSCING00000008556	NP_001027703	Cin2q	4459681
1234	ENSG00000139624	LASS5	Hsa12	48809849	ENSCING00000007135	Q4H395_CIOIN	Cin3p	1259787
4888	ENSG00000111275	ALDH2	Hsa12	110688729	ENSCING00000007853		Cin7q	3337864
4888	ENSG00000128918	ALDH1A2	Hsa15	56032920	ENSCING00000007853		Cin7q	3337864
2317	ENSG00000140564	FURIN	Hsa15	89212889	ENSCING00000005033		Cinscaffold_152	114187
3488	ENSG00000176463	SLCO3A1	Hsa15	90197950	ENSCING00000001637		Cin8q	7321733
2606	ENSG00000140557	ST8SIA2	Hsa15	90738144	ENSCING00000008556	NP_001027703	Cin2q	4459681
4723	ENSG00000173575	CHD2	Hsa15	91244423	ENSCING00000007700		Cin7q	5425296
3075	ENSG00000182175	RGMA	Hsa15	91387651	ENSCING00000004900		Cinscaffold_36	161251
1941	ENSG00000140563	MCTP2	Hsa15	92575955	ENSCING00000003454		Cinscaffold_95	57765
3512	ENSG00000185551	NR2F2	Hsa15	94674950	ENSCING00000002023	Q4H3S1_CIOIN	Cin8q	7452513
1964	ENSG00000140450	ARRDC4	Hsa15	96304947	ENSCING00000015335		Cinscaffold_63	177428
1308	ENSG00000068305	MEF2A	Hsa15	97923712	ENSCING00000000628	Q4H375_CIOIN	Cinscaffold_187	72757
1234	ENSG00000154227	LASS3	Hsa15	98758124	ENSCING00000007135	Q4H395_CIOIN	Cin3p	1259787
4888	ENSG00000184254	ALDH1A3	Hsa15	99237580	ENSCING00000007853		Cin7q	3337864
837	ENSG00000131873	CHSY1	Hsa15	99533456	ENSCING00000009006		Cin4q	373051
608	ENSG00000131876	SNRPA1	Hsa15	99639240	ENSCING00000017376		Cinscaffold_334	37777
2317	ENSG00000140479	PCSK6	Hsa15	99661657	ENSCING00000005033		Cinscaffold_152	114187
2606	ENSG00000157350	ST3GAL2	Hsa16	68972810	ENSCING00000008556	NP_001027703	Cin2q	4459681
2606	ENSG00000177511	ST8SIA3	Hsa18	53169042	ENSCING00000008556	NP_001027703	Cin2q	4459681
2317	ENSG00000115257	PCSK4	Hsa19	1432427	ENSCING00000005033		Cinscaffold_152	114187
1234	ENSG00000090661	LASS4	Hsa19	8180253	ENSCING00000007135	Q4H395_CIOIN	Cin3p	1259787
3512	ENSG00000160113	NR2F6	Hsa19	17203694	ENSCING00000002023	Q4H3S1_CIOIN	Cin8q	7452513
1964	ENSG00000105643	ARRDC2	Hsa19	17972944	ENSCING00000015335		Cinscaffold_63	177428
1308	ENSG00000064489	MEF2B	Hsa19	19117379	ENSCING00000000628	Q4H375_CIOIN	Cinscaffold_187	72757
608	ENSG00000144158	Q9NU36_HUMAN	Hsa2	114131955	ENSCING00000017376		Cinscaffold_334	37777
2606	ENSG00000204180		Hsa20	33572230	ENSCING00000008556	NP_001027703	Cin2q	4459681
2606	ENSG00000064225	ST3GAL6	Hsa3	99934029	ENSCING00000008556	NP_001027703	Cin2q	4459681
1308	ENSG00000081189	MEF2C	Hsa5	88051922	ENSCING00000000628	Q4H375_CIOIN	Cinscaffold_187	72757
1964	ENSG00000113369	ARRDC3	Hsa5	90700299	ENSCING00000015335		Cinscaffold_63	177428
3512	ENSG00000175745	NR2F1	Hsa5	92944799	ENSCING00000002023	Q4H3S1_CIOIN	Cin8q	7452513
1941	ENSG00000175471	MCTP1	Hsa5	94068601	ENSCING00000003454		Cinscaffold_95	57765
2317	ENSG00000175426	PCSK1	Hsa5	95751875	ENSCING00000005033		Cinscaffold_152	114187
4723	ENSG00000153922	CHD1	Hsa5	98218819	ENSCING00000007700		Cin7q	5425296
2606	ENSG00000113532	ST8SIA4	Hsa5	100170803	ENSCING00000008556	NP_001027703	Cin2q	4459681
3488	ENSG00000137571	SLCO5A1	Hsa8	70747129	ENSCING00000001637		Cin8q	7321733
2606	ENSG00000008513	ST3GAL1	Hsa8	134540327	ENSCING00000008556	NP_001027703	Cin2q	4459681
4888	ENSG00000137124	ALDH1B1	Hsa9	38382661	ENSCING00000007853		Cin7q	3337864
4888	ENSG00000165092	ALDH1A1	Hsa9	74705408	ENSCING00000007853		Cin7q	3337864
2317	ENSG00000099139	PCSK5	Hsa9	77695406	ENSCING00000005033		Cinscaffold_152	114187
2606	ENSG00000136840	ST6GALNAC4	Hsa9	129709986	ENSCING00000008556	NP_001027703	Cin2q	4459681

Genomic distribution of the paralog groups of the surrounding 10-Mb window including the ALDH1B1, using the Brachiostoma floridae genomic database as the outgroup for the best reciprocal BLAST hit analysis

Genomic regiona analyzed: human chromosome 9 (33.000 KB - 43.000 KB)

Group ID	Query Gene ID	Query Ext ID	Query Chromosome	Query Start Base	Outg Gene ID	Outg Ext ID	Outg Chromosome	Outg Start Base
5998	ENSG00000176256	HMGB4	Hsa1	34098663	57556	fgenesh2_pm.scaffold_24000033	BflBf_V2_150	3712238
3199	ENSG00000117411	B4GALT2	Hsa1	44217453	211142	e_gw.44.77.1	Bflscaffold_44	522961
6843	ENSG00000152763	WDR78	Hsa1	67051156	242526	e_gw.410.43.1	BflBf_V2_32	3129559
305	ENSG00000162616	DNAJB4	Hsa1	78243126	224427	e_gw.101.51.1	BflBf_V2_127	1737375
6912	ENSG00000117519	CNN3	Hsa1	95135095	61874	fgenesh2_pm.scaffold_435000001	BflBf_V2_127	1574721
1513	ENSG00000169418	NPR1	Hsa1	151917737	241945	e_gw.403.62.1	BflBf_V2_282	456341
1567	ENSG00000143549	TPM3	Hsa1	152395457	59343	fgenesh2_pm.scaffold_126000010	BflBf_V2_65	710427
7235	ENSG00000143569	UBAP2L	Hsa1	152459279	125234	estExt_fgenesh2_pg.C_1750064	BflBf_V2_195	547032
4828	ENSG00000143595	AQP10	Hsa1	152560180	288680	estExt_gwp.C_6430015	Bflscaffold_643	275173
6389	ENSG00000160712	IL6R	Hsa1	152644293	119272	estExt_fgenesh2_pg.C_260094	BflBf_V2_135	2917812
3199	ENSG00000158850	B4GALT3	Hsa1	159407724	211142	e_gw.44.77.1	Bflscaffold_44	522961
3360	ENSG00000122359	ANXA11	Hsa10	81900625	289104	estExt_gwp.C_7090021	Bflscaffold_709	229249
485	ENSG00000171862	PTEN	Hsa10	89612850	209333	e_gw.38.168.1	BflBf_V2_27	2380306
3497	ENSG00000075891	PAX2	Hsa10	102495322	56669	fgenesh2_kg.scaffold_131000004	BflBf_V2_2	1255234
6912	ENSG00000149591	TAGLN	Hsa11	116575250	61874	fgenesh2_pm.scaffold_435000001	BflBf_V2_127	1574721
3189	ENSG00000111275	ALDH2	Hsa12	110688729	106815	fgenesh2_pg.scaffold_550000018	BflBf_V2_19	1936884
5998	ENSG00000189403	HMGB1	Hsa13	29930884	57556	fgenesh2_pm.scaffold_24000033	BflBf_V2_150	3712238
5998	ENSG00000180189		Hsa14	57821354	57556	fgenesh2_pm.scaffold_24000033	BflBf_V2_150	3712238
6857	ENSG00000137766	UNC13C	Hsa15	52314521	102486	fgenesh2_pg.scaffold_410000031	BflBf_V2_32	3039460
4828	ENSG00000103569	AQP9	Hsa15	56217771	288680	estExt_gwp.C_6430015	Bflscaffold_643	275173
2393	ENSG00000171914	TLN2	Hsa15	60726802	129467	estExt_fgenesh2_pg.C_3680027	Bflscaffold_368	623076
1567	ENSG00000140416	TPM1	Hsa15	61121891	59343	fgenesh2_pm.scaffold_126000010	BflBf_V2_65	710427
921	ENSG00000140403	DNAJA4	Hsa15	76343551	264103	estExt_GenewiseH_1.C_660100	BflBf_V2_216	987498
4080	ENSG00000129925	TMEM8	Hsa16	361861	125509	estExt_fgenesh2_pg.C_1850093	Bflscaffold_185	1380730
2570	ENSG00000152910	CNTNAP4	Hsa16	74901295	282496	estExt_gwp.C_2600008	Bflscaffold_260	92841
5889	ENSG00000197566	ZNF624	Hsa17	16464785	71028	fgenesh2_pg.scaffold_31000075	BflBf_V2_204	1465380
5998	ENSG00000182181	441795	Hsa17	38053701	57556	fgenesh2_pm.scaffold_24000033	BflBf_V2_150	3712238
2570	ENSG00000108797	CNTNAP1	Hsa17	38088158	282496	estExt_gwp.C_2600008	Bflscaffold_260	92841
2095	ENSG00000167434	CA4	Hsa17	55582079	127510	estExt_fgenesh2_pg.C_2610037	BflBf_V2_129	872504
5326	ENSG00000141437	MCART2	Hsa18	27593729	113471	estExt_fgenesh2_pm.C_10013	BflBf_V2_258	1157345
1019	ENSG00000099804	CDC34	Hsa19	482733	91986	fgenesh2_pg.scaffold_216000039	Bflscaffold_216	903467
6912	ENSG00000064666	CNN2	Hsa19	977298	61874	fgenesh2_pm.scaffold_435000001	BflBf_V2_127	1574721
6912	ENSG00000130176	CNN1	Hsa19	11510579	61874	fgenesh2_pm.scaffold_435000001	BflBf_V2_127	1574721
305	ENSG00000132002	DNAJB1	Hsa19	14486582	224427	e_gw.101.51.1	BflBf_V2_127	1737375
6857	ENSG00000130477	UNC13A	Hsa19	17573167	102486	fgenesh2_pg.scaffold_410000031	BflBf_V2_32	3039460
5889	ENSG00000198521	ZNF43	Hsa19	21779594	71028	fgenesh2_pg.scaffold_31000075	BflBf_V2_204	1465380
5889	ENSG00000160321	ZNF208	Hsa19	21945325	71028	fgenesh2_pg.scaffold_31000075	BflBf_V2_204	1465380
5889	ENSG00000196109	646825	Hsa19	22278397	71028	fgenesh2_pg.scaffold_31000075	BflBf_V2_204	1465380
5889	ENSG00000167232	ZNF91	Hsa19	23333883	71028	fgenesh2_pg.scaffold_31000075	BflBf_V2_204	1465380
5357	ENSG00000174562	KLK15	Hsa19	56020357	88359	fgenesh2_pg.scaffold_173000024	BflBf_V2_242	511066
5357	ENSG00000129455	KLK8	Hsa19	56191082	88359	fgenesh2_pg.scaffold_173000024	BflBf_V2_242	511066
5357	ENSG00000167757	KLK11	Hsa19	56217301	88359	fgenesh2_pg.scaffold_173000024	BflBf_V2_242	511066
5357	ENSG00000186474	KLK12	Hsa19	56224160	88359	fgenesh2_pg.scaffold_173000024	BflBf_V2_242	511066
5357	ENSG00000167759	KLK13	Hsa19	56251275	88359	fgenesh2_pg.scaffold_173000024	BflBf_V2_242	511066
5357	ENSG00000129437	KLK14	Hsa19	56272386	88359	fgenesh2_pg.scaffold_173000024	BflBf_V2_242	511066
5889	ENSG00000197608	XR_015252.1	Hsa19	57260172	71028	fgenesh2_pg.scaffold_31000075	BflBf_V2_204	1465380
5889	ENSG00000196267	Q6ZNA1_HUMAN	Hsa19	57350074	71028	fgenesh2_pg.scaffold_31000075	BflBf_V2_204	1465380
6912	ENSG00000204718		Hsa2	94766764	61874	fgenesh2_pm.scaffold_435000001	BflBf_V2_127	1574721
3497	ENSG00000125618	PAX8	Hsa2	113690046	56669	fgenesh2_kg.scaffold_131000004	BflBf_V2_2	1255234
2570	ENSG00000155052	CNTNAP5	Hsa2	124499334	282496	estExt_gwp.C_2600008	Bflscaffold_260	92841
5998	ENSG00000181242	Q75MM1_HUMAN	Hsa2	171166409	57556	fgenesh2_pm.scaffold_24000033	BflBf_V2_150	3712238
5998	ENSG00000124097	HMG1L1	Hsa20	55496392	57556	fgenesh2_pm.scaffold_24000033	BflBf_V2_150	3712238
5998	ENSG00000100118	HMG1L10	Hsa22	25286492	57556	fgenesh2_pm.scaffold_24000033	BflBf_V2_150	3712238
5998	ENSG00000132967	Q5T7C0_HUMAN	Hsa3	22398314	57556	fgenesh2_pm.scaffold_24000033	BflBf_V2_150	3712238
6912	ENSG00000144834	TAGLN3	Hsa3	113200276	61874	fgenesh2_pm.scaffold_435000001	BflBf_V2_127	1574721
3199	ENSG00000121578	B4GALT4	Hsa3	120413286	211142	e_gw.44.77.1	Bflscaffold_44	522961
4855	ENSG00000163453	IGFBP7	Hsa4	57591696	148425	gw.967.8.1	Bflscaffold_967	14013
5204	ENSG00000145331	RG9MTD2	Hsa4	100686910	123423	estExt_fgenesh2_pg.C_1200013	Bflscaffold_120	176458
5998	ENSG00000164104	HMGB2	Hsa4	174489434	57556	fgenesh2_pm.scaffold_24000033	BflBf_V2_150	3712238
6389	ENSG00000113494	PRLR	Hsa5	35084621	119272	estExt_fgenesh2_pg.C_260094	BflBf_V2_135	2917812
911	ENSG00000169567	HINT1	Hsa5	130522883	216507	e_gw.66.117.1	BflBf_V2_216	640977
5989	ENSG00000175416	CLTB	Hsa5	175752062	119200	estExt_fgenesh2_pg.C_240224	BflBf_V2_150	3878773
5998	ENSG00000182711	729595	Hsa5	179053804	57556	fgenesh2_pm.scaffold_24000033	BflBf_V2_150	3712238
5998	ENSG00000184579		Hsa7	26988911	57556	fgenesh2_pm.scaffold_24000033	BflBf_V2_150	3712238
5357	ENSG00000173636	PRSS1	Hsa7	142136904	88359	fgenesh2_pg.scaffold_173000024	BflBf_V2_242	511066
5357	ENSG00000204983	NR_001296.2	Hsa7	142158416	88359	fgenesh2_pg.scaffold_173000024	BflBf_V2_242	511066
911	ENSG00000173568	730647	Hsa7	142719896	216507	e_gw.66.117.1	BflBf_V2_216	640977
2570	ENSG00000174469	CNTNAP2	Hsa7	145444902	282496	estExt_gwp.C_2600008	Bflscaffold_260	92841
3792	ENSG00000104756	KCTD9	Hsa8	25341283	245477	e_gw.455.17.1	Bflscaffold_455	441881
3792	ENSG00000187074		Hsa8	31197233	245477	e_gw.455.17.1	Bflscaffold_455	441881
921	ENSG00000086061	DNAJA1	Hsa9	33015209	264103	estExt_GenewiseH_1.C_660100	BflBf_V2_216	987498
3232	ENSG00000122692	SMU1	Hsa9	33031762	289144	estExt_gwp.C_7150002	Bflscaffold_715	27666
3199	ENSG00000086062	B4GALT1	Hsa9	33100635	211142	e_gw.44.77.1	Bflscaffold_44	522961
1284	ENSG00000107262	BAG1	Hsa9	33244163	94947	fgenesh2_pg.scaffold_259000079	BflBf_V2_23	967094
6437	ENSG00000086065	CHMP5	Hsa9	33254167	277348	estExt_gwp.C_340058	BflBf_V2_98	1949197
1806	ENSG00000086102	NFX1	Hsa9	33280509	183675	gw.95.108.1	Bflscaffold_95	305089
4828	ENSG00000165269	AQP7	Hsa9	33374948	288680	estExt_gwp.C_6430015	Bflscaffold_643	275173
4828	ENSG00000165272	AQP3	Hsa9	33431152	288680	estExt_gwp.C_6430015	Bflscaffold_643	275173
1449	ENSG00000165271	NOL6	Hsa9	33451354	279825	estExt_gwp.C_710055	BflBf_V2_185	216378
3360	ENSG00000183059	ANXA2P2	Hsa9	33614223	289104	estExt_gwp.C_7090021	Bflscaffold_709	229249
485	ENSG00000147974	PTENP1	Hsa9	33665438	209333	e_gw.38.168.1	BflBf_V2_27	2380306
5357	ENSG00000010438	PRSS3	Hsa9	33740515	88359	fgenesh2_pg.scaffold_173000024	BflBf_V2_242	511066
1019	ENSG00000107341	UBE2R2	Hsa9	33807182	91986	fgenesh2_pg.scaffold_216000039	Bflscaffold_216	903467
7235	ENSG00000137073	UBAP2	Hsa9	33911691	125234	estExt_fgenesh2_pg.C_1750064	BflBf_V2_195	547032
5880	ENSG00000165006	UBAP1	Hsa9	34169003	107050	fgenesh2_pg.scaffold_560000005	Bflscaffold_560	86904

4500	ENSG00000186638	C9orf48	Hsa9	34242379	266132	estExt_GenewiseH_1.C_1060023	BflBf_V2_113	1187076
5878	ENSG00000164978	NUDT2	Hsa9	34319504	287930	estExt_gwp.C_5600005	Bflscaffold_560	63460
1261	ENSG00000164976	KIAA1161	Hsa9	34356668	92215	fgenesh2_pg.scaffold_219000073	BflBf_V2_69	192878
6843	ENSG00000122735	DNAI1	Hsa9	34448811	242526	e_gw.410.43.1	BflBf_V2_32	3129559
6389	ENSG00000122756	CNTFR	Hsa9	34541430	119272	estExt_fgenesh2_pg.C_260094	BflBf_V2_135	2917812
4400	ENSG00000137100	DCTN3	Hsa9	34603548	114103	estExt_fgenesh2_pm.C_420006	BflBf_V2_6	808578
1034	ENSG00000147955	OPRS1	Hsa9	34624717	124168	estExt_fgenesh2_pg.C_1400072	BflBf_V2_1	119934
1023	ENSG00000137070	GALT	Hsa9	34636635	126461	estExt_fgenesh2_pg.C_2160036	Bflscaffold_216	895721
4319	ENSG00000122733	KIAA1045	Hsa9	34948192	109300	fgenesh2_pg.scaffold_678000012	Bflscaffold_678	243069
305	ENSG00000137094	DNAJB5	Hsa9	34979742	224427	e_gw.101.51.1	BflBf_V2_127	1737375
7060	ENSG00000165280	VCP	Hsa9	35046061	267157	estExt_GenewiseH_1.C_2650020	Bflscaffold_265	90054
5453	ENSG00000165282	PIGO	Hsa9	35078685	82003	fgenesh2_pg.scaffold_107000058	Bflscaffold_107	1252908
662	ENSG00000165283	STOML2	Hsa9	35089593	85895	fgenesh2_pg.scaffold_146000043	BflBf_V2_32	6475704
6857	ENSG00000198722	UNC13B	Hsa9	35151999	102486	fgenesh2_pg.scaffold_410000031	BflBf_V2_32	3039460
2446	ENSG00000107140	TESK1	Hsa9	35595281	126370	estExt_fgenesh2_pg.C_2120066	BflBf_V2_61	1304665
2095	ENSG00000107159	CA9	Hsa9	35663853	127510	estExt_fgenesh2_pg.C_2610037	BflBf_V2_129	872504
1567	ENSG00000198467	TPM2	Hsa9	35671989	59343	fgenesh2_pm.scaffold_126000010	BflBf_V2_65	710427
2393	ENSG00000137076	TLN1	Hsa9	35687336	129467	estExt_fgenesh2_pg.C_3680027	Bflscaffold_368	623076
5045	ENSG00000070610	GBA2	Hsa9	35726863	124315	estExt_fgenesh2_pg.C_1450034	BflBf_V2_123	834549
1013	ENSG00000107185	KIAA0258	Hsa9	35739315	115368	estExt_fgenesh2_pm.C_2160008	Bflscaffold_216	976825
1513	ENSG00000159899	NPR2	Hsa9	35782151	241945	e_gw.403.62.1	BflBf_V2_282	456341
911	ENSG00000137133	HINT2	Hsa9	35802957	216507	e_gw.66.117.1	BflBf_V2_216	640977
4080	ENSG00000137103	C9orf127	Hsa9	35804448	125509	estExt_fgenesh2_pg.C_1850093	Bflscaffold_185	1380730
3307	ENSG00000122707	RECK	Hsa9	36026430	215934	e_gw.64.62.1	BflBf_V2_160	1322338
3053	ENSG00000122694	C9orf19	Hsa9	36126695	239943	e_gw.376.63.1	BflBf_V2_222	588958
5989	ENSG00000122705	CLTA	Hsa9	36180928	119200	estExt_fgenesh2_pg.C_240224	BflBf_V2_150	3878773
891	ENSG00000159921	GNE	Hsa9	36204430	124939	estExt_fgenesh2_pg.C_1640017	BflBf_V2_262	566532
5998	ENSG00000173961		Hsa9	36293539	57556	fgenesh2_pm.scaffold_24000033	BflBf_V2_150	3712238
3507	ENSG00000165304	MELK	Hsa9	36562873	123883	estExt_fgenesh2_pg.C_1310039	BflBf_V2_2	1347618
3497	ENSG00000196092	PAX5	Hsa9	36823272	56669	fgenesh2_kg.scaffold_131000004	BflBf_V2_2	1255234
4493	ENSG00000137106	GRHPR	Hsa9	37412663	104160	fgenesh2_pg.scaffold_454000010	BflBf_V2_2	391503
3792	ENSG00000187988		Hsa9	37467146	245477	e_gw.455.17.1	Bflscaffold_455	441881
6707	ENSG00000147912	FBXO10	Hsa9	37500893	101540	fgenesh2_pg.scaffold_384000038	Bflscaffold_384	664945
5204	ENSG00000165275	RG9MTD3	Hsa9	37743802	123423	estExt_fgenesh2_pg.C_1200013	Bflscaffold_120	176458
544	ENSG00000107371	EXOSC3	Hsa9	37770308	277941	estExt_gwp.C_410281	Bflscaffold_41	2794120
5326	ENSG00000122696	MCART1	Hsa9	37875368	113471	estExt_fgenesh2_pm.C_10013	BflBf_V2_258	1157345
3189	ENSG00000137124	ALDH1B1	Hsa9	38382661	106815	fgenesh2_pg.scaffold_550000018	BflBf_V2_19	1936884
4855	ENSG00000137142	IGFBPL1	Hsa9	38398991	148425	gw.967.8.1	Bflscaffold_967	14013
2570	ENSG00000106714	CNTNAP3	Hsa9	39062764	282496	estExt_gwp.C_2600008	Bflscaffold_260	92841
5889	ENSG00000196409	ZNF658B	Hsa9	39433814	71028	fgenesh2_pg.scaffold_31000075	BflBf_V2_204	1465380
5889	ENSG00000198566	ZNF658	Hsa9	40750700	71028	fgenesh2_pg.scaffold_31000075	BflBf_V2_204	1465380
5889	ENSG00000198416	NP_001027468	Hsa9	41578834	71028	fgenesh2_pg.scaffold_31000075	BflBf_V2_204	1465380
625	ENSG00000204827	Q6ZQT2_HUMAN	Hsa9	42787521	241679	e_gw.400.53.1	BflBf_V2_287	579876
7080	ENSG00000181997		Hsa9	42848068	92631	fgenesh2_pg.scaffold_225000050	BflBf_V2_93	2616274
6912	ENSG00000204826	XR_018140.1	Hsa9	42999838	61874	fgenesh2_pm.scaffold_435000001	BflBf_V2_127	1574721
2570	ENSG00000154529	CNTNAP3B	Hsa9	43624898	282496	estExt_gwp.C_2600008	Bflscaffold_260	92841
7080	ENSG00000176115		Hsa9	66770881	92631	fgenesh2_pg.scaffold_225000050	BflBf_V2_93	2616274
7080	ENSG00000186466	AQP7P2	Hsa9	66961803	92631	fgenesh2_pg.scaffold_225000050	BflBf_V2_93	2616274
6912	ENSG00000204782	XR_016647.1	Hsa9	68788495	61874	fgenesh2_pm.scaffold_435000001	BflBf_V2_127	1574721
625	ENSG00000204781	Q6ZQT2_HUMAN	Hsa9	69387663	241679	e_gw.400.53.1	BflBf_V2_287	579876
625	ENSG00000204777	Q6ZQT2_HUMAN	Hsa9	69588393	241679	e_gw.400.53.1	BflBf_V2_287	579876
5326	ENSG00000176274	MCART6	HsaX	103230554	113471	estExt_fgenesh2_pm.C_10013	BflBf_V2_258	1157345
5998	ENSG00000175117	203510	HsaX	111819805	57556	fgenesh2_pm.scaffold_24000033	BflBf_V2_150	3712238
5998	ENSG00000029993	HMGB3	HsaX	149902417	57556	fgenesh2_pm.scaffold_24000033	BflBf_V2_150	3712238

Genomic distribution of the paralog groups of the surrounding 10-Mb window including the ALDH1B1, using the Ciona intestinalis genomic database as the outgroup for the best reciprocal BLAST hit analysis									
Genomic regiona analyzed: human chromosome 9 (33.000 KB - 43.000 KB)									
Group ID	Query Gene ID	Query Ext ID	Query Chromosome	Query Start Base	Outg Gene ID	Outg Ext ID	Outg Chromosome	Outg Start Base	
73	ENSG00000131686	CA6	Hsa1	8928509	ENSCING00000014543		Cin13q	1604122	
1006	ENSG00000176256	HMGB4	Hsa1	34098663	ENSCING00000004075	Q4H3E0_CIOIN	Cin14q	2070911	
4639	ENSG00000117411	B4GALT2	Hsa1	44217453	ENSCING00000008394		Cin9q	2051709	
3091	ENSG00000162616	DNAJB4	Hsa1	78243126	ENSCING00000006225		Cinscaffold_36	194004	
3982	ENSG00000117519	CNN3	Hsa1	95135095	ENSCING00000007496		Cin5q	430113	
73	ENSG00000118298	CA14	Hsa1	148496793	ENSCING00000014543		Cin13q	1604122	
4661	ENSG00000143412	ANXA9	Hsa1	149221178	ENSCING00000004236		Cin9q	155680	
2339	ENSG00000169418	NPR1	Hsa1	151917737	ENSCING00000001143		Cinscaffold_156	158889	
1013	ENSG00000143549	TPM3	Hsa1	152395457	ENSCING00000002349	TPM1_CIOIN	Cin14q	3018755	
648	ENSG00000143569	UBAP2L	Hsa1	152459279	ENSCING00000008788		Cin4q	5022821	
4502	ENSG00000143595	AQP10	Hsa1	152560180	ENSCING00000009588		Cin9q	2688529	
4639	ENSG00000158850	B4GALT3	Hsa1	159407724	ENSCING00000008394		Cin9q	2051709	
4661	ENSG00000150165	ANXA8L1	Hsa10	46577989	ENSCING00000004236		Cin9q	155680	
4661	ENSG00000186807	ANXA8L2	Hsa10	47216942	ENSCING00000004236		Cin9q	155680	
4661	ENSG00000165390	ANXA8	Hsa10	47875259	ENSCING00000004236		Cin9q	155680	
4661	ENSG00000138279	ANXA7	Hsa10	74805209	ENSCING00000004236		Cin9q	155680	
4661	ENSG00000122359	ANXA11	Hsa10	81900625	ENSCING00000004236		Cin9q	155680	
2187	ENSG00000171862	PTEN	Hsa10	89612850	ENSCING00000002379		Cin14p	552004	
3104	ENSG00000107821	KAZALD1	Hsa10	102810989	ENSCING00000008203		Cinscaffold_36	940796	
1393	ENSG00000110076	NRXN2	Hsa11	64130222	ENSCING00000005088		Cinscaffold_285	22098	
4888	ENSG00000111275	ALDH2	Hsa12	110688729	ENSCING00000007853		Cin7q	3337864	
1006	ENSG00000189403	HMGB1	Hsa13	29930884	ENSCING00000004075	Q4H3E0_CIOIN	Cin14q	2070911	
1006	ENSG00000180189		Hsa14	57821354	ENSCING00000004075	Q4H3E0_CIOIN	Cin14q	2070911	
1393	ENSG00000021645	NRXN3	Hsa14	77779190	ENSCING00000005088		Cinscaffold_285	22098	
3963	ENSG00000171763	SPATA5L1	Hsa15	43481871	ENSCING00000007091		Cin5q	3451073	
1174	ENSG00000137766	UNC13C	Hsa15	52314521	ENSCING00000002173		Cin3p	1457745	
4888	ENSG00000128918	ALDH1A2	Hsa15	56032920	ENSCING00000007853		Cin7q	3337864	
4502	ENSG00000103569	AQP9	Hsa15	56217771	ENSCING00000009588		Cin9q	2688529	
4661	ENSG00000182718	ANXA2	Hsa15	58426643	ENSCING00000004236		Cin9q	155680	
4207	ENSG00000171914	TLN2	Hsa15	60726802	ENSCING00000009522		Cin3q	1044744	
1013	ENSG00000140416	TPM1	Hsa15	61121891	ENSCING00000002349	TPM1_CIOIN	Cin14q	3018755	
73	ENSG00000074410	CA12	Hsa15	61402784	ENSCING00000014543		Cin13q	1604122	
2950	ENSG00000178761	C15orf17	Hsa15	72982013	ENSCING00000000935		Cin12q	4270353	
4300	ENSG00000140403	DNAJA4	Hsa15	76343551	ENSCING00000008988		Cin3q	4862633	
4888	ENSG00000184254	ALDH1A3	Hsa15	99237580	ENSCING00000007853		Cin7q	3337864	
4300	ENSG00000069345	DNAJA2	Hsa16	45547796	ENSCING00000008988		Cin3q	4862633	
1013	ENSG00000187536		Hsa16	51246717	ENSCING00000002349	TPM1_CIOIN	Cin14q	3018755	
2339	ENSG00000132518	GUCY2D	Hsa17	7846713	ENSCING00000001143		Cinscaffold_156	158889	
1006	ENSG00000182181	441795	Hsa17	38053701	ENSCING00000004075	Q4H3E0_CIOIN	Cin14q	2070911	
73	ENSG00000167434	CA4	Hsa17	55582079	ENSCING00000014543		Cin13q	1604122	
4639	ENSG00000118276	B4GALT6	Hsa18	27457131	ENSCING00000008394		Cin9q	2051709	
493	ENSG00000141437	MCART2	Hsa18	27593729	ENSCING00000006011		Cin1p	2135424	
4313	ENSG00000099804	CDC34	Hsa19	482733	ENSCING00000008583		Cin3q	3271890	
3982	ENSG00000064666	CNN2	Hsa19	977298	ENSCING00000007496		Cin5q	430113	
3982	ENSG00000130176	CNN1	Hsa19	11510579	ENSCING00000007496		Cin5q	430113	
3091	ENSG00000132002	DNAJB1	Hsa19	14486582	ENSCING00000006225		Cinscaffold_36	194004	
1013	ENSG00000167460	TPM4	Hsa19	16039348	ENSCING00000002349	TPM1_CIOIN	Cin14q	3018755	
1174	ENSG00000130477	UNC13A	Hsa19	17573167	ENSCING00000002173		Cin3p	1457745	
1952	ENSG00000160321	ZNF208	Hsa19	21945325	ENSCING00000003072		Cinscaffold_63	72814	
1952	ENSG00000196109	646825	Hsa19	22278397	ENSCING00000003072		Cinscaffold_63	72814	
1952	ENSG00000167232	ZNF91	Hsa19	23333883	ENSCING00000003072		Cinscaffold_63	72814	
1952	ENSG00000128000	ZNF780B	Hsa19	45231537	ENSCING00000003072		Cinscaffold_63	72814	
1934	ENSG00000167748	KLK1	Hsa19	56014216	ENSCING00000003513		Cinscaffold_95	202695	
1934	ENSG00000167751	KLK2	Hsa19	56068501	ENSCING00000003513		Cinscaffold_95	202695	
1934	ENSG00000167755	KLK6	Hsa19	56153700	ENSCING00000003513		Cinscaffold_95	202695	
1934	ENSG00000169035	KLK7	Hsa19	56171541	ENSCING00000003513		Cinscaffold_95	202695	
1934	ENSG00000167757	KLK11	Hsa19	56217301	ENSCING00000003513		Cinscaffold_95	202695	
1952	ENSG00000197608	XR_015252.1	Hsa19	57260172	ENSCING00000003072		Cinscaffold_63	72814	
1952	ENSG00000196267	Q6ZNA1_HUMAN	Hsa19	57350074	ENSCING00000003072		Cinscaffold_63	72814	
1952	ENSG00000198482	NP_001034975	Hsa19	57722717	ENSCING00000003072		Cinscaffold_63	72814	
1952	ENSG00000170949	ZNF160	Hsa19	58261004	ENSCING00000003072		Cinscaffold_63	72814	
1952	ENSG00000196931	Q96IR2_HUMAN	Hsa19	58546740	ENSCING00000003072		Cinscaffold_63	72814	
4160	ENSG00000138081	FBXO11	Hsa2	47887565	ENSCING00000004333		Cin3q	5546719	
1393	ENSG00000205000		Hsa2	50546085	ENSCING00000005088		Cinscaffold_285	22098	
4661	ENSG00000196975	ANXA4	Hsa2	69801427	ENSCING00000004236		Cin9q	155680	
3982	ENSG00000204718		Hsa2	94766764	ENSCING00000007496		Cin5q	430113	
1006	ENSG00000181242	Q75MM1_HUMAN	Hsa2	171166409	ENSCING00000004075	Q4H3E0_CIOIN	Cin14q	2070911	
4639	ENSG00000158470	B4GALT5	Hsa20	47682889	ENSCING00000008394		Cin9q	2051709	
1006	ENSG00000124097	HMG1L1	Hsa20	55496392	ENSCING00000004075	Q4H3E0_CIOIN	Cin14q	2070911	
1006	ENSG00000100118	HMG1L10	Hsa22	25286492	ENSCING00000004075	Q4H3E0_CIOIN	Cin14q	2070911	
1006	ENSG00000132967	Q5T7C0_HUMAN	Hsa3	22398314	ENSCING00000004075	Q4H3E0_CIOIN	Cin14q	2070911	
4639	ENSG00000121578	B4GALT4	Hsa3	120413286	ENSCING00000008394		Cin9q	2051709	
3104	ENSG00000163453	IGFBP7	Hsa4	57591696	ENSCING00000008203		Cinscaffold_36	940796	
4661	ENSG00000138772	ANXA3	Hsa4	79694613	ENSCING00000004236		Cin9q	155680	
4661	ENSG00000164111	ANXA5	Hsa4	122808598	ENSCING00000004236		Cin9q	155680	
4661	ENSG00000109511	ANXA10	Hsa4	169250310	ENSCING00000004236		Cin9q	155680	
1006	ENSG00000164104	HMGB2	Hsa4	174489434	ENSCING00000004075	Q4H3E0_CIOIN	Cin14q	2070911	
2578	ENSG00000169567	HINT1	Hsa5	130522883	ENSCING00000011420		Cin2q	5080923	
4730	ENSG00000175416	CLTB	Hsa5	175752062	ENSCING00000003978		Cin7q	683121	
1006	ENSG00000182711	729595	Hsa5	179053804	ENSCING00000004075	Q4H3E0_CIOIN	Cin14q	2070911	
1952	ENSG00000196670	ZFP62	Hsa5	180208335	ENSCING00000003072		Cinscaffold_63	72814	
1006	ENSG00000184579		Hsa7	26988911	ENSCING00000004075	Q4H3E0_CIOIN	Cin14q	2070911	

1952	ENSG00000196247	ZNF588	Hsa7	63763946	ENSCING00000003072		Cinscaffold_63	72814
1934	ENSG00000173636	PRSS1	Hsa7	142136904	ENSCING00000003513		Cinscaffold_95	202695
1934	ENSG00000204983	NR_001296.2	Hsa7	142158416	ENSCING00000003513		Cinscaffold_95	202695
1934	ENSG00000204982	PRSS2	Hsa7	142167698	ENSCING00000003513		Cinscaffold_95	202695
1934	ENSG00000204981		Hsa7	142170799	ENSCING00000003513		Cinscaffold_95	202695
2578	ENSG00000173568	730647	Hsa7	142719896	ENSCING00000011420		Cin2q	5080923
1393	ENSG00000174469	CNTNAP2	Hsa7	145444902	ENSCING00000005088		Cinscaffold_285	22098
4661	ENSG00000104537	ANXA13	Hsa8	124762216	ENSCING00000004236		Cin9q	155680
4300	ENSG00000086061	DNAJA1	Hsa9	33015209	ENSCING00000008988		Cin3q	4862633
3743	ENSG00000122692	SMU1	Hsa9	33031762	ENSCING00000001827		Cin9p	1968564
4639	ENSG00000086062	B4GALT1	Hsa9	33100635	ENSCING00000008394		Cin9q	2051709
4268	ENSG00000107262	BAG1	Hsa9	33244163	ENSCING00000012405		Cin3q	5537053
474	ENSG00000086065	CHMP5	Hsa9	33254167	ENSCING00000004764		Cin1p	2324922
1852	ENSG00000086102	NFX1	Hsa9	33280509	ENSCING00000004746	Q4H346_CIOIN	Cin1q	3931781
4502	ENSG00000165269	AQP7	Hsa9	33374948	ENSCING00000009588		Cin9q	2688529
4502	ENSG00000165272	AQP3	Hsa9	33431152	ENSCING00000009588		Cin9q	2688529
3151	ENSG00000165271	NOL6	Hsa9	33451354	ENSCING00000000461		Cinscaffold_26	861853
4661	ENSG00000183059	ANXA2P2	Hsa9	33614223	ENSCING00000004236		Cin9q	155680
2187	ENSG00000147974	PTENP1	Hsa9	33665438	ENSCING00000002379		Cin14p	552004
1934	ENSG00000010438	PRSS3	Hsa9	33740515	ENSCING00000003513		Cinscaffold_95	202695
4313	ENSG00000107341	UBE2R2	Hsa9	33807182	ENSCING00000008583		Cin3q	3271890
648	ENSG00000137073	UBAP2	Hsa9	33911691	ENSCING00000008788		Cin4q	5022821
4686	ENSG00000165006	UBAP1	Hsa9	34169003	ENSCING00000002034		Cinscaffold_153	66380
2950	ENSG00000164970	C9orf25	Hsa9	34388182	ENSCING00000000935		Cin12q	4270353
642	ENSG00000122735	DNAI1	Hsa9	34448811	ENSCING00000008832		Cin4q	4647678
4083	ENSG00000137100	DCTN3	Hsa9	34603548	ENSCING00000009222		Cin5q	5907120
1806	ENSG00000147955	OPRS1	Hsa9	34624717	ENSCING00000007963		Cin1q	2296594
29	ENSG00000137070	GALT	Hsa9	34636635	ENSCING00000016647		Cinscaffold_1244	5198
3091	ENSG00000137094	DNAJB5	Hsa9	34979742	ENSCING00000006225		Cinscaffold_36	194004
3963	ENSG00000165280	VCP	Hsa9	35046061	ENSCING00000007091		Cin5q	3451073
4000	ENSG00000165283	STOML2	Hsa9	35089593	ENSCING00000009357		Cin5q	3116795
1174	ENSG00000198722	UNC13B	Hsa9	35151999	ENSCING00000002173		Cin3p	1457745
73	ENSG00000107159	CA9	Hsa9	35663853	ENSCING00000014543		Cin13q	1604122
1013	ENSG00000198467	TPM2	Hsa9	35671989	ENSCING00000002349	TPM1_CIOIN	Cin14q	3018755
4207	ENSG00000137076	TLN1	Hsa9	35687336	ENSCING00000009522		Cin3q	1044744
1921	ENSG00000070610	GBA2	Hsa9	35726863	ENSCING00000003478		Cinscaffold_95	124220
714	ENSG00000107185	KIAA0258	Hsa9	35739315	ENSCING00000007998		Cin4q	1126155
2339	ENSG00000159899	NPR2	Hsa9	35782151	ENSCING00000001143		Cinscaffold_156	158889
2578	ENSG00000137133	HINT2	Hsa9	35802957	ENSCING00000011420		Cin2q	5080923
4434	ENSG00000122707	RECK	Hsa9	36026430	ENSCING00000002924		Cinscaffold_121	236164
1118	ENSG00000122694	C9orf19	Hsa9	36126695	ENSCING00000000989		Cin14q	2018430
4730	ENSG00000122705	CLTA	Hsa9	36180928	ENSCING00000003978		Cin7q	683121
1006	ENSG00000173961		Hsa9	36293539	ENSCING00000004075	Q4H3E0_CIOIN	Cin14q	2070911
3004	ENSG00000165304	MELK	Hsa9	36562873	ENSCING00000003875		Cin12q	2449813
2900	ENSG00000137106	GRHPR	Hsa9	37412663	ENSCING00000014190		Cin12q	1804388
4160	ENSG00000147912	FBXO10	Hsa9	37500893	ENSCING00000004333		Cin3q	5546719
208	ENSG00000107371	EXOSC3	Hsa9	37770308	ENSCING00000004719		Cinscaffold_89	28344
2679	ENSG00000122741	WDR32	Hsa9	37790864	ENSCING00000009813		Cin2q	4742834
493	ENSG00000122696	MCART1	Hsa9	37875368	ENSCING00000006011		Cin1p	2135424
4888	ENSG00000137124	ALDH1B1	Hsa9	38382661	ENSCING00000007853		Cin7q	3337864
3104	ENSG00000137142	IGFBPL1	Hsa9	38398991	ENSCING00000008203		Cinscaffold_36	940796
1393	ENSG00000106714	CNTNAP3	Hsa9	39062764	ENSCING00000005088		Cinscaffold_285	22098
1952	ENSG00000196409	ZNF658B	Hsa9	39433814	ENSCING00000003072		Cinscaffold_63	72814
1952	ENSG00000198566	ZNF658	Hsa9	40750700	ENSCING00000003072		Cinscaffold_63	72814
1952	ENSG00000198416	NP_001027468	Hsa9	41578834	ENSCING00000003072		Cinscaffold_63	72814
4502	ENSG00000181997		Hsa9	42848068	ENSCING00000009588		Cin9q	2688529
3982	ENSG00000204826	XR_018140.1	Hsa9	42999838	ENSCING00000007496		Cin5q	430113
1393	ENSG00000154529	CNTNAP3B	Hsa9	43624898	ENSCING00000005088		Cinscaffold_285	22098
4502	ENSG00000176115		Hsa9	66770881	ENSCING00000009588		Cin9q	2688529
4502	ENSG00000186466	AQP7P2	Hsa9	66961803	ENSCING00000009588		Cin9q	2688529
3982	ENSG00000204782	XR_016647.1	Hsa9	68788495	ENSCING00000007496		Cin5q	430113
4888	ENSG00000165092	ALDH1A1	Hsa9	74705408	ENSCING00000007853		Cin7q	3337864
4661	ENSG00000135046	ANXA1	Hsa9	74956493	ENSCING00000004236		Cin9q	155680
1952	ENSG00000147118	ZNF182	HsaX	47719194	ENSCING00000003072		Cinscaffold_63	72814
493	ENSG00000176274	MCART6	HsaX	103230554	ENSCING00000006011		Cin1p	2135424
2339	ENSG00000101890	GUCY2F	HsaX	108502791	ENSCING00000001143		Cinscaffold_156	158889
1006	ENSG00000175117	203510	HsaX	111819805	ENSCING00000004075	Q4H3E0_CIOIN	Cin14q	2070911
1006	ENSG00000029993	HMGB3	HsaX	149902417	ENSCING00000004075	Q4H3E0_CIOIN	Cin14q	2070911

Genomic distribution of the paralog groups of the surrounding 10-Mb window in the ALDH1A3-ogm GN, using the Brachiostoma floridae genomic database as the outgroup for the best reciprocal BLAST hit analysis

Genomic regiona analyzed: human chromosome 5 90000 KB - 100000 KB)

Group ID	Query Gene ID	Query Ext ID	Query Chromosome	Query Start Base	Outg Gene ID	Outg Ext ID	Outg Chromosome	Outg Start Base
562	ENSG00000182175	RGMA	Hsa15	91387651	82627	fgenesh2_pg.scaffold_113000060	BflBf_V2_119	1262645
562	ENSG00000174136	RGMB	Hsa5	98132900	82627	fgenesh2_pg.scaffold_113000060	BflBf_V2_119	1262645
562	ENSG00000168509	HFE2	Hsa1	144124628	82627	fgenesh2_pg.scaffold_113000060	BflBf_V2_119	1262645
563	ENSG00000175471	MCTP1	Hsa5	94068601	82577	fgenesh2_pg.scaffold_113000010	BflBf_V2_119	107322
563	ENSG00000140563	MCTP2	Hsa15	92575955	82577	fgenesh2_pg.scaffold_113000010	BflBf_V2_119	107322
764	ENSG00000058729	RIOK2	Hsa5	96522327	235502	e_gw.324.72.1	BflBf_V2_169	449678
1187	ENSG00000178015	GPR150	Hsa5	94981538	65210	fgenesh2_pg.scaffold_6000205	BflBf_V2_159	1009353
1435	ENSG00000164291	ARSK	Hsa5	94916608	77511	fgenesh2_pg.scaffold_71000048	BflBf_V2_185	233847
1562	ENSG00000173221	GLRX	Hsa5	95175309	243036	e_gw.421.61.1	Bflscaffold_421	619649
1562	ENSG00000118990		Hsa5	161110059	243036	e_gw.421.61.1	Bflscaffold_421	619649
1565	ENSG00000198677	KIAA0372	Hsa5	94825355	102906	fgenesh2_pg.scaffold_421000023	Bflscaffold_421	357825
1767	ENSG00000153922	CHD1	Hsa5	98218819	266878	estExt_GenewiseH_1.C_2530071	Bflscaffold_253	1057823
1767	ENSG00000173575	CHD2	Hsa15	91244423	266878	estExt_GenewiseH_1.C_2530071	Bflscaffold_253	1057823
1788	ENSG00000164292	RHOBTB3	Hsa5	95092635	124528	estExt_fgenesh2_pg.C_1490092	BflBf_V2_226	2009424
2856	ENSG00000175426	PCSK1	Hsa5	95751875	105744	fgenesh2_pg.scaffold_505000017	BflBf_V2_296	229274
2856	ENSG00000140564	FURIN	Hsa15	89212889	105744	fgenesh2_pg.scaffold_505000017	BflBf_V2_296	229274
2856	ENSG00000115257	PCSK4	Hsa19	1432427	105744	fgenesh2_pg.scaffold_505000017	BflBf_V2_296	229274
3096	ENSG00000166825	ANPEP	Hsa15	88129131	118386	estExt_fgenesh2_pg.C_130007	Bflscaffold_13	179644
3096	ENSG00000072657	TRHDE	Hsa12	70952730	118386	estExt_fgenesh2_pg.C_130007	Bflscaffold_13	179644
3096	ENSG00000138792	ENPEP	Hsa4	111616697	118386	estExt_fgenesh2_pg.C_130007	Bflscaffold_13	179644
3096	ENSG00000164307	ARTS1_HUMAN	Hsa5	96122277	118386	estExt_fgenesh2_pg.C_130007	Bflscaffold_13	179644
3096	ENSG00000164308	NP_071745.1	Hsa5	96237474	118386	estExt_fgenesh2_pg.C_130007	Bflscaffold_13	179644
3096	ENSG00000172901	LAEVR_HUMAN	Hsa5	115326214	118386	estExt_fgenesh2_pg.C_130007	Bflscaffold_13	179644
3096	ENSG00000113441	LNPEP	Hsa5	96296924	118386	estExt_fgenesh2_pg.C_130007	Bflscaffold_13	179644
4142	ENSG00000175449	RFESD	Hsa5	95008239	107504	fgenesh2_pg.scaffold_579000026	Bflscaffold_579	352170
4182	ENSG00000131467	PSME3	Hsa17	38238949	98409	fgenesh2_pg.scaffold_321000027	Bflscaffold_321	472931
4182	ENSG00000092010	PSME1	Hsa14	23661207	98409	fgenesh2_pg.scaffold_321000027	Bflscaffold_321	472931
4182	ENSG00000100911	PSME2	Hsa14	23682449	98409	fgenesh2_pg.scaffold_321000027	Bflscaffold_321	472931
4182	ENSG00000174144		Hsa5	97574862	98409	fgenesh2_pg.scaffold_321000027	Bflscaffold_321	472931
5173	ENSG00000113369	ARRDC3	Hsa5	90700299	118530	estExt_fgenesh2_pg.C_150153	BflBf_V2_65	4485499
5173	ENSG00000105643	ARRDC2	Hsa19	17972944	118530	estExt_fgenesh2_pg.C_150153	BflBf_V2_65	4485499
5173	ENSG00000140450	ARRDC4	Hsa15	96304947	118530	estExt_fgenesh2_pg.C_150153	BflBf_V2_65	4485499
5173	ENSG00000117289	TXNIP	Hsa1	144149826	118530	estExt_fgenesh2_pg.C_150153	BflBf_V2_65	4485499
5451	ENSG00000153347	FAM81B	Hsa5	94754247	82002	fgenesh2_pg.scaffold_107000057	Bflscaffold_107	1243595
6141	ENSG00000152022	LIX1L	Hsa1	144188442	282166	estExt_gwp.C_2500055	BflBf_V2_88	2240381
6141	ENSG00000145721	LIX1	Hsa5	96453330	282166	estExt_gwp.C_2500055	BflBf_V2_88	2240381
6321	ENSG00000105656	ELL	Hsa19	18414475	95758	fgenesh2_pg.scaffold_273000010	BflBf_V2_124	132598
6321	ENSG00000118985	ELL2	Hsa5	95246558	95758	fgenesh2_pg.scaffold_273000010	BflBf_V2_124	132598

Genomic distribution of the paralog groups of the surrounding 10-Mb window including the ALDH1A3-ogm, using the Ciona intestinalis genomic database as the outgroup for the best reciprocal BLAST hit analysis								
Genomic regiona analyzed: human chromosome 5 90000 KB - 100000 KB)								
Group ID	Query Gene ID	Query Ext ID	Query Chromosome	Query Start Base	Outg Gene ID	Outg Ext ID	Outg Chromosome	Outg Start Base
1941	ENSG00000175471	MCTP1	Hsa5	94068601	ENSCING00000003454		Cinscaffold_95	57765
1941	ENSG00000140563	MCTP2	Hsa15	92575955	ENSCING00000003454		Cinscaffold_95	57765
1964	ENSG00000113369	ARRDC3	Hsa5	90700299	ENSCING00000015335		Cinscaffold_63	177428
1964	ENSG00000105643	ARRDC2	Hsa19	17972944	ENSCING00000015335		Cinscaffold_63	177428
1964	ENSG00000140450	ARRDC4	Hsa15	96304947	ENSCING00000015335		Cinscaffold_63	177428
2226	ENSG00000152022	LIX1L	Hsa1	144188442	ENSCING00000014662		Cin14p	1565662
2226	ENSG00000145721	LIX1	Hsa5	96453330	ENSCING00000014662		Cin14p	1565662
2317	ENSG00000140479	PCSK6	Hsa15	99661657	ENSCING00000005033		Cinscaffold_152	114187
2317	ENSG00000099139	PCSK5	Hsa9	77695406	ENSCING00000005033		Cinscaffold_152	114187
2317	ENSG00000140564	FURIN	Hsa15	89212889	ENSCING00000005033		Cinscaffold_152	114187
2317	ENSG00000115257	PCSK4	Hsa19	1432427	ENSCING00000005033		Cinscaffold_152	114187
2317	ENSG00000175426	PCSK1	Hsa5	95751875	ENSCING00000005033		Cinscaffold_152	114187
2976	ENSG00000164307	ARTS1_HUMAN	Hsa5	96122277	ENSCING00000004229		Cin12q	3076100
2976	ENSG00000113441	LNPEP	Hsa5	96296924	ENSCING00000004229		Cin12q	3076100
2976	ENSG00000164308	NP_071745.1	Hsa5	96237474	ENSCING00000004229		Cin12q	3076100
3089	ENSG00000157470	FAM81A	Hsa15	57517664	ENSCING00000004867		Cinscaffold_36	107149
3089	ENSG00000153347	FAM81B	Hsa5	94754247	ENSCING00000004867		Cinscaffold_36	107149
3149	ENSG00000072422	RHOBTB1	Hsa10	62299202	ENSCING00000000475		Cinscaffold_26	835545
3149	ENSG00000008853	RHOBTB2	Hsa8	22900875	ENSCING00000000475		Cinscaffold_26	835545
3149	ENSG00000164292	RHOBTB3	Hsa5	95092635	ENSCING00000000475		Cinscaffold_26	835545
3369	ENSG00000092010	PSME1	Hsa14	23661207	ENSCING00000007472		Cin8q	5094361
3369	ENSG00000100911	PSME2	Hsa14	23682449	ENSCING00000007472		Cin8q	5094361
3369	ENSG00000174144		Hsa5	97574862	ENSCING00000007472		Cin8q	5094361
3512	ENSG00000185551	NR2F2	Hsa15	94674950	ENSCING00000002023	Q4H3S1_CIOIN	Cin8q	7452513
3512	ENSG00000175745	NR2F1	Hsa5	92944799	ENSCING00000002023	Q4H3S1_CIOIN	Cin8q	7452513
3512	ENSG00000160113	NR2F6	Hsa19	17203694	ENSCING00000002023	Q4H3S1_CIOIN	Cin8q	7452513
3640	ENSG00000173221	GLRX	Hsa5	95175309	ENSCING00000016950		Cinscaffold_1545	164
3640	ENSG00000118990		Hsa5	161110059	ENSCING00000016950		Cinscaffold_1545	164
4723	ENSG00000173575	CHD2	Hsa15	91244423	ENSCING00000007700		Cin7q	5425296
4723	ENSG00000153922	CHD1	Hsa5	98218819	ENSCING00000007700		Cin7q	5425296
4880	ENSG00000058729	RIOK2	Hsa5	96522327	ENSCING00000002364		Cin7q	7066384

Genomic distribution of the paralog groups of the surrounding 10-Mb window in the ALDH1A-related GN in Hsa1, using the Brachiostoma floridae genomic database as the outgroup for the best reciprocal BLAST hit analysis

Genomic regiona analyzed: human chromosome 1 145000 KB - 155000 KB)

Group ID	Query Gene ID	Query Ext ID	Query Chromosome	Query Start Base	Outg Gene ID	Outg Ext ID	Outg Chromosome	Outg Start Base
22	ENSG00000184270	HIST2H2AB	Hsa1	148125698	200697	e_gw.5.720.1	Bflscaffold_5	3040596
22	ENSG00000184260	HIST2H2AC	Hsa1	148125149	200697	e_gw.5.720.1	Bflscaffold_5	3040596
22	ENSG00000180573	HIST1H2AC	Hsa6	26232352	200697	e_gw.5.720.1	Bflscaffold_5	3040596
22	ENSG00000164508	HIST1H2AA	Hsa6	25834339	200697	e_gw.5.720.1	Bflscaffold_5	3040596
22	ENSG00000183558	HIST2H2AA4	Hsa1	148080129	200697	e_gw.5.720.1	Bflscaffold_5	3040596
22	ENSG00000203812	H2A2A_HUMAN	Hsa1	148089252	200697	e_gw.5.720.1	Bflscaffold_5	3040596
22	ENSG00000182611	HIST1H2AJ	Hsa6	27890111	200697	e_gw.5.720.1	Bflscaffold_5	3040596
22	ENSG00000196787	H2A1_HUMAN	Hsa6	27208800	200697	e_gw.5.720.1	Bflscaffold_5	3040596
22	ENSG00000181218	HIST3H2A	Hsa1	226711303	200697	e_gw.5.720.1	Bflscaffold_5	3040596
22	ENSG00000184825	H2A1H_HUMAN	Hsa6	27222840	200697	e_gw.5.720.1	Bflscaffold_5	3040596
22	ENSG00000184348	HIST1H2AG	Hsa6	27913704	200697	e_gw.5.720.1	Bflscaffold_5	3040596
22	ENSG00000198374	HIST1H2AL	Hsa6	27941086	200697	e_gw.5.720.1	Bflscaffold_5	3040596
22	ENSG00000196866	HIST1H2AM	Hsa6	27968514	200697	e_gw.5.720.1	Bflscaffold_5	3040596
27	ENSG00000183598	HIST2H3D	Hsa1	148051450	64755	fgenesh2_pg.scaffold_5000171	Bflscaffold_5	2936273
27	ENSG00000203813	HIST2H3C	Hsa1	148077734	64755	fgenesh2_pg.scaffold_5000171	Bflscaffold_5	2936273
27	ENSG00000203811	HIST2H3A	Hsa1	148090805	64755	fgenesh2_pg.scaffold_5000171	Bflscaffold_5	2936273
27	ENSG00000196747	HIST1H2AI	Hsa6	27883949	64755	fgenesh2_pg.scaffold_5000171	Bflscaffold_5	2936273
27	ENSG00000197409	HIST1H3D	Hsa6	26305047	64755	fgenesh2_pg.scaffold_5000171	Bflscaffold_5	2936273
27	ENSG00000197153	HIST1H3J	Hsa6	27966139	64755	fgenesh2_pg.scaffold_5000171	Bflscaffold_5	2936273
27	ENSG00000182572	HIST1H3I	Hsa6	27947662	64755	fgenesh2_pg.scaffold_5000171	Bflscaffold_5	2936273
27	ENSG00000198366	H31_HUMAN	Hsa6	26128697	64755	fgenesh2_pg.scaffold_5000171	Bflscaffold_5	2936273
27	ENSG00000178458	HIST1H3A	Hsa6	26379181	64755	fgenesh2_pg.scaffold_5000171	Bflscaffold_5	2936273
27	ENSG00000196532	HIST1H3C	Hsa6	26153591	64755	fgenesh2_pg.scaffold_5000171	Bflscaffold_5	2936273
27	ENSG00000112727	HIST1H3F	Hsa6	26358402	64755	fgenesh2_pg.scaffold_5000171	Bflscaffold_5	2936273
27	ENSG00000124693	HIST1H3B	Hsa6	26139857	64755	fgenesh2_pg.scaffold_5000171	Bflscaffold_5	2936273
27	ENSG00000196966	HIST1H3E	Hsa6	26333362	64755	fgenesh2_pg.scaffold_5000171	Bflscaffold_5	2936273
57	ENSG00000143303	C1orf66	Hsa1	154964858	200329	e_gw.5.310.1	BflBf_V2_174	3796315
81	ENSG00000196374	HIST1H2BM	Hsa6	27890801	200319	e_gw.5.191.1	Bflscaffold_5	2938095
81	ENSG00000196226	HIST1H2BB	Hsa6	26151484	200319	e_gw.5.191.1	Bflscaffold_5	2938095
81	ENSG00000184678	HIST2H2BE	Hsa1	148122633	200319	e_gw.5.191.1	Bflscaffold_5	2938095
81	ENSG00000196331	HIST1H2BO	Hsa6	27969182	200319	e_gw.5.191.1	Bflscaffold_5	2938095
81	ENSG00000197697	HIST1H2BE	Hsa6	26292002	200319	e_gw.5.191.1	Bflscaffold_5	2938095
81	ENSG00000158373	HIST1H2BD	Hsa6	26266328	200319	e_gw.5.191.1	Bflscaffold_5	2938095
81	ENSG00000197846	HIST1H2BF	Hsa6	26307727	200319	e_gw.5.191.1	Bflscaffold_5	2938095
81	ENSG00000187990	HIST1H2BG	Hsa6	26324433	200319	e_gw.5.191.1	Bflscaffold_5	2938095
81	ENSG00000180596	HIST1H2BC	Hsa6	26222612	200319	e_gw.5.191.1	Bflscaffold_5	2938095
81	ENSG00000168242	HIST1H2BI	Hsa6	26381183	200319	e_gw.5.191.1	Bflscaffold_5	2938095
81	ENSG00000197459	HIST1H2BH	Hsa6	26359858	200319	e_gw.5.191.1	Bflscaffold_5	2938095
81	ENSG00000196501	HIST1H2BN	Hsa6	27913517	200319	e_gw.5.191.1	Bflscaffold_5	2938095
81	ENSG00000124635	HIST1H2BJ	Hsa6	27207560	200319	e_gw.5.191.1	Bflscaffold_5	2938095
81	ENSG00000197903	HIST1H2BK	Hsa6	27222129	200319	e_gw.5.191.1	Bflscaffold_5	2938095
81	ENSG00000203814	HIST2H2BF	Hsa1	148049907	200319	e_gw.5.191.1	Bflscaffold_5	2938095
136	ENSG00000163354	DCST2	Hsa1	153257620	101779	fgenesh2_pg.scaffold_391000033	BflBf_V2_292	524886
136	ENSG00000163357	DCST1	Hsa1	153272924	101779	fgenesh2_pg.scaffold_391000033	BflBf_V2_292	524886
141	ENSG00000180304	OAZ2	Hsa15	62766829	241182	e_gw.391.24.1	BflBf_V2_292	371290
141	ENSG00000143450	OAZ3	Hsa1	150002069	241182	e_gw.391.24.1	BflBf_V2_292	371290
356	ENSG00000131781	FMO5	Hsa1	145124462	113828	estExt_fgenesh2_pm.C_200029	BflBf_V2_261	1656776
356	ENSG00000007933	FMO3	Hsa1	169326642	113828	estExt_fgenesh2_pm.C_200029	BflBf_V2_261	1656776
356	ENSG00000010932	FMO1	Hsa1	169484262	113828	estExt_fgenesh2_pm.C_200029	BflBf_V2_261	1656776
356	ENSG00000143151	Q5VT49_HUMAN	Hsa1	164840618	113828	estExt_fgenesh2_pm.C_200029	BflBf_V2_261	1656776
383	ENSG00000143554	SLC27A3	Hsa1	152013454	68867	fgenesh2_pg.scaffold_20000215	BflBf_V2_261	1662722
526	ENSG00000137819	PAQR5	Hsa15	67439358	96405	fgenesh2_pg.scaffold_284000016	BflBf_V2_163	694828
526	ENSG00000160781	PAQR6	Hsa1	154479830	96405	fgenesh2_pg.scaffold_284000016	BflBf_V2_163	694828
526	ENSG00000188582	PAQR9	Hsa3	144163735	96405	fgenesh2_pg.scaffold_284000016	BflBf_V2_163	694828
555	ENSG00000187631	CKS1_HUMAN	Hsa5	61843827	59154	fgenesh2_pm.scaffold_113000001	BflBf_V2_119	32652
555	ENSG00000173207	CKS1B	Hsa1	153213753	59154	fgenesh2_pm.scaffold_113000001	BflBf_V2_119	32652
555	ENSG00000197474		Hsa10	30026870	59154	fgenesh2_pm.scaffold_113000001	BflBf_V2_119	32652
555	ENSG00000178556	646469	HsaX	30545444	59154	fgenesh2_pm.scaffold_113000001	BflBf_V2_119	32652
555	ENSG00000123975	CKS2	Hsa9	91115925	59154	fgenesh2_pm.scaffold_113000001	BflBf_V2_119	32652
556	ENSG00000183578	TNFAIP8L3	Hsa15	49136093	59157	fgenesh2_pm.scaffold_113000004	BflBf_V2_119	498136
556	ENSG00000145779	TNFAIP8	Hsa5	118756403	59157	fgenesh2_pm.scaffold_113000004	BflBf_V2_119	498136
556	ENSG00000185361	TNFAIP8L1	Hsa19	4590530	59157	fgenesh2_pm.scaffold_113000004	BflBf_V2_119	498136
556	ENSG00000163154	TNFAIP8L2	Hsa1	149395729	59157	fgenesh2_pm.scaffold_113000004	BflBf_V2_119	498136
558	ENSG00000160752	FDPS	Hsa1	153545267	123202	estExt_fgenesh2_pg.C_1130049	BflBf_V2_119	1094847
558	ENSG00000196898	XR_017751.1	Hsa7	76435514	123202	estExt_fgenesh2_pg.C_1130049	BflBf_V2_119	1094847
558	ENSG00000197470	Q499Z7_HUMAN	Hsa7	75937415	123202	estExt_fgenesh2_pg.C_1130049	BflBf_V2_119	1094847
651	ENSG00000143437	ARNT	Hsa1	149048810	124387	estExt_fgenesh2_pg.C_1460068	BflBf_V2_32	6880339
651	ENSG00000172379	ARNT2	Hsa15	78483747	124387	estExt_fgenesh2_pg.C_1460068	BflBf_V2_32	6880339
660	ENSG00000198715	C1orf85	Hsa1	154529103	85896	fgenesh2_pg.scaffold_146000044	BflBf_V2_32	6481418
666	ENSG00000143420	ENSA	Hsa1	148839951	124375	estExt_fgenesh2_pg.C_1460045	BflBf_V2_32	6487768
666	ENSG00000128989	ARP19_HUMAN	Hsa15	50626535	124375	estExt_fgenesh2_pg.C_1460045	BflBf_V2_32	6487768
666	ENSG00000204637	646227	Hsa5	180341879	124375	estExt_fgenesh2_pg.C_1460045	BflBf_V2_32	6487768
666	ENSG00000198779	643896	Hsa2	100492171	124375	estExt_fgenesh2_pg.C_1460045	BflBf_V2_32	6487768
666	ENSG00000177261		Hsa2	215270704	124375	estExt_fgenesh2_pg.C_1460045	BflBf_V2_32	6487768
673	ENSG00000081189	MEF2C	Hsa5	88051922	59582	fgenesh2_pm.scaffold_146000014	BflBf_V2_32	6536355
673	ENSG00000116604	MEF2D	Hsa1	154700143	59582	fgenesh2_pm.scaffold_146000014	BflBf_V2_32	6536355
673	ENSG00000064489	MEF2B	Hsa19	19117379	59582	fgenesh2_pm.scaffold_146000014	BflBf_V2_32	6536355
681	ENSG00000143515	ATP8B2	Hsa1	152564654	85893	fgenesh2_pg.scaffold_146000041	BflBf_V2_32	6435722
681	ENSG00000104043	ATP8B4	Hsa15	47937727	85893	fgenesh2_pg.scaffold_146000041	BflBf_V2_32	6435722
681	ENSG00000081923	ATP8B1	Hsa18	53466591	85893	fgenesh2_pg.scaffold_146000041	BflBf_V2_32	6435722
755	ENSG00000080709	KCNN2	Hsa5	113725565	235511	e_gw.324.15.1	BflBf_V2_169	739377
755	ENSG00000143603	KCNN3	Hsa1	152946526	235511	e_gw.324.15.1	BflBf_V2_169	739377
755	ENSG00000105642	KCNN1	Hsa19	17945647	235511	e_gw.324.15.1	BflBf_V2_169	739377
755	ENSG00000104783	KCNN4	Hsa19	48962578	235511	e_gw.324.15.1	BflBf_V2_169	739377
759	ENSG00000143374	TARSL1	Hsa1	148726514	268618	estExt_GenewiseH_1.C_3240030	BflBf_V2_169	472598

846	ENSG00000159409	TNRC4	Hsa1	149941454	114402	estExt_fgenesh2_pm.C_730008	BflBf_V2_243	973756
908	ENSG00000143621	ILF2	Hsa1	151900905	279457	estExt_gwp.C_660054	Bflscaffold_66	798032
919	ENSG00000143398	PIP5K1A	Hsa1	149437651	216408	e_gw.66.2.1	BflBf_V2_216	1914305
919	ENSG00000186111	PIP5K1C	Hsa19	3581182	216408	e_gw.66.2.1	BflBf_V2_216	1914305
919	ENSG00000107242	PIP5K1B	Hsa9	70510436	216408	e_gw.66.2.1	BflBf_V2_216	1914305
919	ENSG00000173780	Q8TBY6_HUMAN	Hsa6	7931335	216408	e_gw.66.2.1	BflBf_V2_216	1914305
926	ENSG00000167461	RAB8A	Hsa19	16083467	114333	estExt_fgenesh2_pm.C_660005	BflBf_V2_216	649805
926	ENSG00000166128	RAB8B	Hsa15	61268781	114333	estExt_fgenesh2_pm.C_660005	BflBf_V2_216	649805
926	ENSG00000143545	RAB13	Hsa1	152220751	114333	estExt_fgenesh2_pm.C_660005	BflBf_V2_216	649805
926	ENSG00000205319	649713	Hsa12	54660473	114333	estExt_fgenesh2_pm.C_660005	BflBf_V2_216	649805
928	ENSG00000160785	NP_055470.1	Hsa1	154430502	216361	e_gw.66.39.1	BflBf_V2_216	1298677
1026	ENSG00000143363	PRUNE	Hsa1	149247577	239767	e_gw.373.23.1	BflBf_V2_88	1731434
1026	ENSG00000156035	C9orf65	Hsa9	78593080	239767	e_gw.373.23.1	BflBf_V2_88	1731434
1027	ENSG00000167654	ATCAY	Hsa19	3831672	239803	e_gw.373.4.1	BflBf_V2_88	1754013
1027	ENSG00000140299	BNIP2	Hsa15	57742356	239803	e_gw.373.4.1	BflBf_V2_88	1754013
1027	ENSG00000163141	BNIPL	Hsa1	149275670	239803	e_gw.373.4.1	BflBf_V2_88	1754013
1028	ENSG00000140350	ANP32A	Hsa15	66845408	239771	e_gw.373.51.1	BflBf_V2_88	1797018
1028	ENSG00000143401	ANP32E	Hsa1	148457341	239771	e_gw.373.51.1	BflBf_V2_88	1797018
1028	ENSG00000184701	ANP32C	Hsa4	165337609	239771	e_gw.373.51.1	BflBf_V2_88	1797018
1028	ENSG00000139223	ANP32D	Hsa12	47152715	239771	e_gw.373.51.1	BflBf_V2_88	1797018
1234	ENSG00000160714	UBE2Q1	Hsa1	152787675	57036	fgenesh2_pm.scaffold_6000039	Bflscaffold_6	4887873
1234	ENSG00000140367	UBE2Q2	Hsa15	73922855	57036	fgenesh2_pm.scaffold_6000039	Bflscaffold_6	4887873
1276	ENSG00000117362	APH1A	Hsa1	148504428	123275	estExt_fgenesh2_pg.C_1150114	BflBf_V2_41	39989
1276	ENSG00000138613	APH1B	Hsa15	61356844	123275	estExt_fgenesh2_pg.C_1150114	BflBf_V2_41	39989
1322	ENSG00000104537	ANXA13	Hsa8	124762216	282940	estExt_gwp.C_2780035	Bflscaffold_278	411470
1322	ENSG00000196975	ANXA4	Hsa2	69801427	282940	estExt_gwp.C_2780035	Bflscaffold_278	411470
1322	ENSG00000135046	ANXA1	Hsa9	74956493	282940	estExt_gwp.C_2780035	Bflscaffold_278	411470
1322	ENSG00000109511	ANXA10	Hsa4	169250310	282940	estExt_gwp.C_2780035	Bflscaffold_278	411470
1322	ENSG00000143412	ANXA9	Hsa1	149221178	282940	estExt_gwp.C_2780035	Bflscaffold_278	411470
1411	ENSG00000140519	RHCG	Hsa15	87815646	248117	e_gw.507.23.1	Bflscaffold_507	338749
1411	ENSG00000132677	RHBG	Hsa1	154605627	248117	e_gw.507.23.1	Bflscaffold_507	338749
1445	ENSG00000113368	LMNB1	Hsa5	126140214	121709	estExt_fgenesh2_pg.C_710026	BflBf_V2_297	501066
1445	ENSG00000160789	LMNA	Hsa1	154318993	121709	estExt_fgenesh2_pg.C_710026	BflBf_V2_297	501066
1445	ENSG00000176619	LMNB2	Hsa19	2379165	121709	estExt_fgenesh2_pg.C_710026	BflBf_V2_297	501066
1451	ENSG00000185033	SEMA4B	Hsa15	88529156	279857	estExt_gwp.C_710214	BflBf_V2_185	1130267
1451	ENSG00000137872	SEMA6D	Hsa15	45797978	279857	estExt_gwp.C_710214	BflBf_V2_185	1130267
1451	ENSG00000196189	SEMA4A	Hsa1	154386434	279857	estExt_gwp.C_710214	BflBf_V2_185	1130267
1459	ENSG00000160716	CHRNB2	Hsa1	152806881	217686	e_gw.71.216.1	BflBf_V2_185	937155
1467	ENSG00000150712	MTMR12	Hsa5	32262872	217622	e_gw.71.177.1	Bflscaffold_71	659744
1467	ENSG00000014914	MTMR11	Hsa1	148167168	217622	e_gw.71.177.1	Bflscaffold_71	659744
1513	ENSG00000169418	NPR1	Hsa1	151917737	241945	e_gw.403.62.1	BflBf_V2_282	456341
1513	ENSG00000159899	NPR2	Hsa9	35782151	241945	e_gw.403.62.1	BflBf_V2_282	456341
1519	ENSG00000198952	SMG5	Hsa1	154485639	102221	fgenesh2_pg.scaffold_403000022	BflBf_V2_282	277111
1522	ENSG00000187225	LCE3C	Hsa1	150839822	241920	e_gw.403.27.1	BflBf_V2_282	225008
1557	ENSG00000085365	SCAMP1	Hsa5	77692110	136229	gw.421.6.1	Bflscaffold_421	202736
1557	ENSG00000140497	SCAMP2	Hsa15	72924249	136229	gw.421.6.1	Bflscaffold_421	202736
1557	ENSG00000116521	SCAMP3	Hsa1	153492394	136229	gw.421.6.1	Bflscaffold_421	202736
1557	ENSG00000198794	SCAMP5	Hsa15	73074958	136229	gw.421.6.1	Bflscaffold_421	202736
1560	ENSG00000136631	VPS45A	Hsa1	148305993	270701	estExt_GenewiseH_1.C_4210057	Bflscaffold_421	570162
1567	ENSG00000143549	TPM3	Hsa1	152395457	59343	fgenesh2_pm.scaffold_126000010	BflBf_V2_65	710427
1567	ENSG00000198467	TPM2	Hsa9	35671989	59343	fgenesh2_pm.scaffold_126000010	BflBf_V2_65	710427
1567	ENSG00000140416	TPM1	Hsa15	61121891	59343	fgenesh2_pm.scaffold_126000010	BflBf_V2_65	710427
1575	ENSG00000143575	HAX1	Hsa1	152511611	123687	estExt_fgenesh2_pg.C_1260016	Bflscaffold_126	168624
1581	ENSG00000178796	Q8N9Z1_HUMAN	Hsa1	149949533	123701	estExt_fgenesh2_pg.C_1260061	Bflscaffold_126	994679
1699	ENSG00000143452	HORMAD1	Hsa1	148937160	246028	e_gw.465.24.1	Bflscaffold_465	250712
1699	ENSG00000176635	HORMAD2	Hsa22	28806453	246028	e_gw.465.24.1	Bflscaffold_465	250712
1818	ENSG00000125459	MSTO1	Hsa1	153846631	288884	estExt_gwp.C_6750021	Bflscaffold_675	169062
2039	ENSG00000143436	MRPL9	Hsa1	149998743	231456	e_gw.282.10.1	BflBf_V2_23	3188722
2069	ENSG00000173171	MTX1	Hsa1	153445114	132083	estExt_fgenesh2_pg.C_6290010	Bflscaffold_629	114816
2071	ENSG00000169231	THBS3	Hsa1	153432003	273182	estExt_GenewiseH_1.C_6290011	Bflscaffold_629	80966
2071	ENSG00000113296	THBS4	Hsa5	79366859	273182	estExt_GenewiseH_1.C_6290011	Bflscaffold_629	80966
2071	ENSG00000105664	COMP	Hsa19	18754584	273182	estExt_GenewiseH_1.C_6290011	Bflscaffold_629	80966
2481	ENSG00000169241	RAG1AP1	Hsa1	153374444	280364	estExt_gwp.C_780238	BflBf_V2_32	4829021
2492	ENSG00000132872	SYT4	Hsa18	39101857	219414	e_gw.78.118.1	BflBf_V2_32	3927051
2492	ENSG00000132718	SYT11	Hsa1	154095924	219414	e_gw.78.118.1	BflBf_V2_32	3927051
2618	ENSG00000143570	SLC39A1	Hsa1	152198199	243746	e_gw.431.48.1	BflBf_V2_111	711914
2682	ENSG00000159445	THEM4	Hsa1	150112684	97343	fgenesh2_pg.scaffold_301000048	Bflscaffold_301	906358
2682	ENSG00000196407	THEM5	Hsa1	150084844	97343	fgenesh2_pg.scaffold_301000048	Bflscaffold_301	906358
2766	ENSG00000198558	HIST1H4L	Hsa6	27948956	61348	fgenesh2_pm.scaffold_348000005	Bflscaffold_348	830827
2766	ENSG00000198339	HIST1H4I	Hsa6	27214346	61348	fgenesh2_pm.scaffold_348000005	Bflscaffold_348	830827
2766	ENSG00000197238	HIST1H4J	Hsa6	27899863	61348	fgenesh2_pm.scaffold_348000005	Bflscaffold_348	830827
2766	ENSG00000182217	H4_HUMAN	Hsa1	148097376	61348	fgenesh2_pm.scaffold_348000005	Bflscaffold_348	830827
2766	ENSG00000183941	HIST2H4A	Hsa1	148070866	61348	fgenesh2_pm.scaffold_348000005	Bflscaffold_348	830827
2766	ENSG00000198518	HIST4H4	Hsa6	26312817	61348	fgenesh2_pm.scaffold_348000005	Bflscaffold_348	830827
2766	ENSG00000188987	HIST1H4D	Hsa6	26296972	61348	fgenesh2_pm.scaffold_348000005	Bflscaffold_348	830827
2766	ENSG00000196176	HIST1H4A	Hsa6	26129886	61348	fgenesh2_pm.scaffold_348000005	Bflscaffold_348	830827
2766	ENSG00000197061	HIST1H4C	Hsa6	26212105	61348	fgenesh2_pm.scaffold_348000005	Bflscaffold_348	830827
2766	ENSG00000158406	HIST1H4H	Hsa6	26389262	61348	fgenesh2_pm.scaffold_348000005	Bflscaffold_348	830827
2766	ENSG00000198327	HIST1H4F	Hsa6	26348540	61348	fgenesh2_pm.scaffold_348000005	Bflscaffold_348	830827
2766	ENSG00000124529	HIST1H4B	Hsa6	26135148	61348	fgenesh2_pm.scaffold_348000005	Bflscaffold_348	830827
2766	ENSG00000197837	H4_HUMAN	Hsa12	14814974	61348	fgenesh2_pm.scaffold_348000005	Bflscaffold_348	830827
2766	ENSG00000197914	HIST1H4K	Hsa6	27906973	61348	fgenesh2_pm.scaffold_348000005	Bflscaffold_348	830827
2766	ENSG00000124578	HIST1H4G	Hsa6	26354888	61348	fgenesh2_pm.scaffold_348000005	Bflscaffold_348	830827
3155	ENSG00000163207	IVL	Hsa1	151147645	104821	fgenesh2_pg.scaffold_476000025	BflBf_V2_18	3280932
3379	ENSG00000131791	PRKAB2	Hsa1	145093309	89923	fgenesh2_pg.scaffold_191000101	BflBf_V2_29	609098
3379	ENSG00000111725	PRKAB1	Hsa12	118590144	89923	fgenesh2_pg.scaffold_191000101	BflBf_V2_29	609098
3440	ENSG00000143314	MRPL24	Hsa1	154973718	286645	estExt_gwp.C_4500057	Bflscaffold_450	421305
3510	ENSG00000198618	PPIA	Hsa21	19151917	114823	estExt_fgenesh2_pm.C_1310004	BflBf_V2_2	831007
3510	ENSG00000196262	PPIA_HUMAN	Hsa7	44802804	114823	estExt_fgenesh2_pm.C_1310004	BflBf_V2_2	831007

3510	ENSG00000174715		Hsa5	81341171	114823	estExt_fgenesh2_pm.C_1310004	BflBf_V2_2	831007
3510	ENSG00000203826	728945	Hsa1	147072639	114823	estExt_fgenesh2_pm.C_1310004	BflBf_V2_2	831007
3510	ENSG00000203829	728945	Hsa1	146910635	114823	estExt_fgenesh2_pm.C_1310004	BflBf_V2_2	831007
3510	ENSG00000188875	728945	Hsa1	146568591	114823	estExt_fgenesh2_pm.C_1310004	BflBf_V2_2	831007
3510	ENSG00000203847	XR_016130.1	Hsa1	142555943	114823	estExt_fgenesh2_pm.C_1310004	BflBf_V2_2	831007
3510	ENSG00000198936	NP_839944.1	Hsa1	147819281	114823	estExt_fgenesh2_pm.C_1310004	BflBf_V2_2	831007
3510	ENSG00000198161	PPIAL4	Hsa1	146418535	114823	estExt_fgenesh2_pm.C_1310004	BflBf_V2_2	831007
3510	ENSG00000198360	NP_839944.1	Hsa1	143072096	114823	estExt_fgenesh2_pm.C_1310004	BflBf_V2_2	831007
3550	ENSG00000122012	SV2C	Hsa5	75463332	102736	fgenesh2_pg.scaffold_416000027	Bflscaffold_416	509520
3550	ENSG00000159164	SV2A	Hsa1	148141494	102736	fgenesh2_pg.scaffold_416000027	Bflscaffold_416	509520
3550	ENSG00000185518	SV2B	Hsa15	89570104	102736	fgenesh2_pg.scaffold_416000027	Bflscaffold_416	509520
3552	ENSG00000117360	PRPF3	Hsa1	148560549	286006	estExt_gwp.C_4160012	Bflscaffold_416	174271
3736	ENSG00000152214	RIT2	Hsa18	38577191	57645	fgenesh2_pm.scaffold_29000004	Bflscaffold_29	287886
3736	ENSG00000143622	RIT1	Hsa1	154134223	57645	fgenesh2_pm.scaffold_29000004	Bflscaffold_29	287886
3795	ENSG00000143416	SELENBP1	Hsa1	149603402	278700	estExt_gwp.C_530071	Bflscaffold_53	547217
3996	ENSG00000163462	TRIM46	Hsa1	153412978	98282	fgenesh2_pg.scaffold_319000001	Bflscaffold_319	11199
4014	ENSG00000116586	MAPIP_HUMAN	Hsa1	154291167	56889	fgenesh2_pm.scaffold_4000008	BflBf_V2_145	2395517
4078	ENSG00000159352	PSMD4	Hsa1	149493803	125492	estExt_fgenesh2_pg.C_1850058	BflBf_V2_39	2597590
4120	ENSG00000176444	CLK2	Hsa1	153499283	282533	estExt_gwp.C_2640004	Bflscaffold_264	86118
4120	ENSG00000178340	CLK2P	Hsa7	23591206	282533	estExt_gwp.C_2640004	Bflscaffold_264	86118
4120	ENSG00000179335	CLK3	Hsa15	72687766	282533	estExt_gwp.C_2640004	Bflscaffold_264	86118
4120	ENSG00000113240	CLK4	Hsa5	177962272	282533	estExt_gwp.C_2640004	Bflscaffold_264	86118
4120	ENSG00000013441	CLK1	Hsa2	201425978	282533	estExt_gwp.C_2640004	Bflscaffold_264	86118
4180	ENSG00000198962	CYCSP52	Hsa1	149142996	235255	e_gw.321.129.1	Bflscaffold_321	816289
4180	ENSG00000172115	CYCS	Hsa7	25124802	235255	e_gw.321.129.1	Bflscaffold_321	816289
4180	ENSG00000186709		Hsa16	25373161	235255	e_gw.321.129.1	Bflscaffold_321	816289
4180	ENSG00000184844		Hsa8	33946621	235255	e_gw.321.129.1	Bflscaffold_321	816289
4180	ENSG00000188512		Hsa13	23872841	235255	e_gw.321.129.1	Bflscaffold_321	816289
4180	ENSG00000174440		Hsa2	100353051	235255	e_gw.321.129.1	Bflscaffold_321	816289
4237	ENSG00000176624	RKHD2	Hsa18	46954920	126022	estExt_fgenesh2_pg.C_2010087	BflBf_V2_32	5164970
4237	ENSG00000183496	RKHD3	Hsa15	80121183	126022	estExt_fgenesh2_pg.C_2010087	BflBf_V2_32	5164970
4237	ENSG00000181588	RKHD1	Hsa19	1505672	126022	estExt_fgenesh2_pg.C_2010087	BflBf_V2_32	5164970
4237	ENSG00000203759	92312	Hsa1	154312989	126022	estExt_fgenesh2_pg.C_2010087	BflBf_V2_32	5164970
4238	ENSG00000143578	CREB3L4	Hsa1	152207003	126026	estExt_fgenesh2_pg.C_2010091	BflBf_V2_32	5125619
4240	ENSG00000143376	SNX27	Hsa1	149851182	90729	fgenesh2_pg.scaffold_201000034	BflBf_V2_32	5738922
4250	ENSG00000160783	PMF1	Hsa1	154449397	90699	fgenesh2_pg.scaffold_201000004	BflBf_V2_32	6101719
4326	ENSG00000163131	CTSS	Hsa1	148969175	284345	estExt_gwp.C_3340139	BflBf_V2_20	52815
4334	ENSG00000143387	CTSK	Hsa1	149035308	284308	estExt_gwp.C_3340074	BflBf_V2_20	240687
4336	ENSG00000113384	GOLPH3	Hsa5	32160567	231282	e_gw.280.7.1	BflBf_V2_159	580498
4336	ENSG00000143457	GOLPH3L	Hsa1	148885325	231282	e_gw.280.7.1	BflBf_V2_159	580498
4450	ENSG00000178096	BOLA1	Hsa1	148125110	203075	e_gw.9.112.1	BflBf_V2_167	1857803
4508	ENSG00000186269	LCE1F	Hsa1	151015472	181798	gw.106.139.1	Bflscaffold_106	1860123
4598	ENSG00000143553	SNAPAP	Hsa1	151897754	118250	estExt_fgenesh2_pg.C_110121	BflBf_V2_136	910843
4729	ENSG00000143409	FAM63A	Hsa1	149232306	234152	e_gw.311.43.1	BflBf_V2_153	64833
4770	ENSG00000163468	CCT3	Hsa1	154545376	283359	estExt_gwp.C_2960079	Bflscaffold_296	524769
4790	ENSG00000143543	JTB	Hsa1	152213369	247586	e_gw.494.69.1	BflBf_V2_32	7605128
4828	ENSG00000143595	AQP10	Hsa1	152560180	288680	estExt_gwp.C_6430015	Bflscaffold_643	275173
4828	ENSG00000103569	AQP9	Hsa15	56217771	288680	estExt_gwp.C_6430015	Bflscaffold_643	275173
4828	ENSG00000165272	AQP3	Hsa9	33431152	288680	estExt_gwp.C_6430015	Bflscaffold_643	275173
4828	ENSG00000165269	AQP7	Hsa9	33374948	288680	estExt_gwp.C_6430015	Bflscaffold_643	275173
4830	ENSG00000132676	DAP3	Hsa1	153925473	253250	e_gw.643.5.1	Bflscaffold_643	138929
4969	ENSG00000163344	PMVK	Hsa1	153163834	114776	estExt_fgenesh2_pm.C_1230015	BflBf_V2_32	2930833
5050	ENSG00000135018	UBQLN1	Hsa9	85464698	124348	estExt_fgenesh2_pg.C_1450100	BflBf_V2_123	112871
5050	ENSG00000160803	UBQLN4	Hsa1	154271716	124348	estExt_fgenesh2_pg.C_1450100	BflBf_V2_123	112871
5050	ENSG00000174995	650339	Hsa3	150186338	124348	estExt_fgenesh2_pg.C_1450100	BflBf_V2_123	112871
5050	ENSG00000188021	UBQLN2	HsaX	56606797	124348	estExt_fgenesh2_pg.C_1450100	BflBf_V2_123	112871
5050	ENSG00000175518	NP_659490.3	Hsa11	5492199	124348	estExt_fgenesh2_pg.C_1450100	BflBf_V2_123	112871
5070	ENSG00000177628	GBA	Hsa1	153470867	85797	fgenesh2_pg.scaffold_145000055	BflBf_V2_123	574172
5075	ENSG00000118298	CA14	Hsa1	148496793	129334	estExt_fgenesh2_pg.C_3590004	Bflscaffold_359	58664
5133	ENSG00000117262	GPR89A	Hsa1	144475768	114456	estExt_fgenesh2_pm.C_770004	BflBf_V2_162	1795560
5133	ENSG00000188092	GPR89_HUMAN	Hsa1	145867130	114456	estExt_fgenesh2_pm.C_770004	BflBf_V2_162	1795560
5133	ENSG00000196644	728932	Hsa1	144595234	114456	estExt_fgenesh2_pm.C_770004	BflBf_V2_162	1795560
5259	ENSG00000143379	SETDB1	Hsa1	149165512	125045	estExt_fgenesh2_pg.C_1670003	BflBf_V2_39	897945
5487	ENSG00000107362	C9orf77	Hsa9	73667188	112094	fgenesh2_pg.scaffold_1036000002	Bflscaffold_1036	21125
5487	ENSG00000182502	150207	Hsa22	20799236	112094	fgenesh2_pg.scaffold_1036000002	Bflscaffold_1036	21125
5487	ENSG00000136379	Q6PCB6_HUMAN	Hsa15	78774737	112094	fgenesh2_pg.scaffold_1036000002	Bflscaffold_1036	21125
5487	ENSG00000129968	FAM108A1	Hsa19	1827976	112094	fgenesh2_pg.scaffold_1036000002	Bflscaffold_1036	21125
5487	ENSG00000203704	648359	Hsa1	212845423	112094	fgenesh2_pg.scaffold_1036000002	Bflscaffold_1036	21125
5487	ENSG00000198658	Q5RGM9_HUMAN	Hsa1	144787932	112094	fgenesh2_pg.scaffold_1036000002	Bflscaffold_1036	21125
5487	ENSG00000203835	FAM108A2	Hsa1	146084969	112094	fgenesh2_pg.scaffold_1036000002	Bflscaffold_1036	21125
5487	ENSG00000182556		Hsa22	19352115	112094	fgenesh2_pg.scaffold_1036000002	Bflscaffold_1036	21125
5488	ENSG00000067225	PKM2	Hsa15	70278424	110402	fgenesh2_pg.scaffold_765000006	BflBf_V2_347	124708
5488	ENSG00000143627	PKLR	Hsa1	153526254	110402	fgenesh2_pg.scaffold_765000006	BflBf_V2_347	124708
5735	ENSG00000179085	DPM3	Hsa1	153378991	276321	estExt_gwp.C_100377	BflBf_V2_158	4565445
6134	ENSG00000185236	RAB11B	Hsa19	8361229	266781	estExt_GenewiseH_1.C_2500058	BflBf_V2_88	2250578
6134	ENSG00000103769	RAB11A	Hsa15	63948850	266781	estExt_GenewiseH_1.C_2500058	BflBf_V2_88	2250578
6134	ENSG00000132698	RAB25	Hsa1	154297575	266781	estExt_GenewiseH_1.C_2500058	BflBf_V2_88	2250578
6237	ENSG00000139624	LASS5	Hsa12	48809849	280654	estExt_gwp.C_860189	BflBf_V2_207	2065554
6237	ENSG00000143418	LASS2	Hsa1	149204286	280654	estExt_gwp.C_860189	BflBf_V2_207	2065554
6237	ENSG00000154227	LASS3	Hsa15	98758124	280654	estExt_gwp.C_860189	BflBf_V2_207	2065554
6293	ENSG00000143365	LRRN6D	Hsa1	150039364	132257	estExt_fgenesh2_pg.C_6630011	Bflscaffold_663	214264
6317	ENSG00000163113	OTUD7B	Hsa1	148178855	230625	e_gw.273.104.1	BflBf_V2_124	767237
6317	ENSG00000169918	OTUD7A	Hsa15	29562621	230625	e_gw.273.104.1	BflBf_V2_124	767237
6324	ENSG00000140575	IQGAP1	Hsa15	88732477	282799	estExt_gwp.C_2730082	Bflscaffold_273	895788
6324	ENSG00000145703	IQGAP2	Hsa5	75734905	282799	estExt_gwp.C_2730082	Bflscaffold_273	895788
6324	ENSG00000183856	IQGAP3	Hsa1	154761821	282799	estExt_gwp.C_2730082	Bflscaffold_273	895788
6348	ENSG00000143368	SF3B4	Hsa1	148161833	116781	estExt_fgenesh2_pm.C_9540001	Bflscaffold_954	6309
6349	ENSG00000163156	SCNM1	Hsa1	149405142	88281	fgenesh2_pg.scaffold_172000018	Bflscaffold_172	403867
6352	ENSG00000163155	LYSMD1	Hsa1	149398846	88280	fgenesh2_pg.scaffold_172000017	Bflscaffold_172	400196

6389	ENSG00000113494	PRLR	Hsa5	35084621	119272	estExt_fgenesh2_pg.C_260094	BflBf_V2_135	2917812
6389	ENSG00000122756	CNTFR	Hsa9	34541430	119272	estExt_fgenesh2_pg.C_260094	BflBf_V2_135	2917812
6389	ENSG00000160712	IL6R	Hsa1	152644293	119272	estExt_fgenesh2_pg.C_260094	BflBf_V2_135	2917812
6571	ENSG00000163159	VPS72	Hsa1	149415404	126813	estExt_fgenesh2_pg.C_2300054	Bflscaffold_230	1155109
6583	ENSG00000160688	FLAD1	Hsa1	153222441	255975	e_gw.767.10.1	BflBf_V2_124	930633
6590	ENSG00000160679	C1orf77	Hsa1	151873149	116232	estExt_fgenesh2_pm.C_4520005	BflBf_V2_88	1106158
6591	ENSG00000159377	PSMB4	Hsa1	149610623	286692	estExt_gwp.C_4520012	BflBf_V2_88	1045449
6593	ENSG00000163382	APOA1BP	Hsa1	154828178	104103	fgenesh2_pg.scaffold_452000028	BflBf_V2_88	1374222
6850	ENSG00000167491	GATAD2A	Hsa19	19237469	130063	estExt_fgenesh2_pg.C_4100036	BflBf_V2_32	2976163
6850	ENSG00000143614	GATAD2B	Hsa1	152043825	130063	estExt_fgenesh2_pg.C_4100036	BflBf_V2_32	2976163
7187	ENSG00000143624	INTS3	Hsa1	151967167	126263	estExt_fgenesh2_pg.C_2090059	BflBf_V2_40	3158614
7235	ENSG00000137073	UBAP2	Hsa9	33911691	125234	estExt_fgenesh2_pg.C_1750064	BflBf_V2_195	547032
7235	ENSG00000143569	UBAP2L	Hsa1	152459279	125234	estExt_fgenesh2_pg.C_1750064	BflBf_V2_195	547032
7302	ENSG00000143393	PIK4CB	Hsa1	149531037	69754	fgenesh2_pg.scaffold_25000022	BflBf_V2_43	330248

Genomic distribution of the paralog groups of the surrounding 10-Mb window including the ALDH1A-related GN in Hsa1, using the Ciona intestinalis genomic database as the outgroup for the best reciprocal BLAST hit analysis								
Genomic regiona analyzed: human chromosome 1 145000 KB - 155000 KB)								
Group ID	Query Gene ID	Query Ext ID	Query Chromosome	Query Start Base	Outg Gene ID	Outg Ext ID	Outg Chromosome	Outg Start Base
15	ENSG00000169231	THBS3	Hsa1	153432003	ENSCING00000008607		Cinscaffold_157	122215
15	ENSG00000113296	THBS4	Hsa5	79366859	ENSCING00000008607		Cinscaffold_157	122215
15	ENSG00000105664	COMP	Hsa19	18754584	ENSCING00000008607		Cinscaffold_157	122215
73	ENSG00000118298	CA14	Hsa1	148496793	ENSCING00000014543		Cin13q	1604122
73	ENSG00000131686	CA6	Hsa1	8928509	ENSCING00000014543		Cin13q	1604122
73	ENSG00000074410	CA12	Hsa15	61402784	ENSCING00000014543		Cin13q	1604122
73	ENSG00000107159	CA9	Hsa9	35663853	ENSCING00000014543		Cin13q	1604122
73	ENSG00000167434	CA4	Hsa17	55582079	ENSCING00000014543		Cin13q	1604122
120	ENSG00000123975	CKS2	Hsa9	91115925	ENSCING00000017633		Cinscaffold_114	362569
120	ENSG00000187631	CKS1_HUMAN	Hsa5	61843827	ENSCING00000017633		Cinscaffold_114	362569
120	ENSG00000173207	CKS1B	Hsa1	153213753	ENSCING00000017633		Cinscaffold_114	362569
120	ENSG00000197474		Hsa10	30026870	ENSCING00000017633		Cinscaffold_114	362569
120	ENSG00000178556	646469	HsaX	30545444	ENSCING00000017633		Cinscaffold_114	362569
144	ENSG00000160716	CHRNB2	Hsa1	152806881	ENSCING00000002138		Cinscaffold_65	615233
144	ENSG00000117971	CHRNB4	Hsa15	76703691	ENSCING00000002138		Cinscaffold_65	615233
144	ENSG00000135902	CHRND	Hsa2	233099166	ENSCING00000002138		Cinscaffold_65	615233
228	ENSG00000143314	MRPL24	Hsa1	154973718	ENSCING00000000305		Cinscaffold_789	14409
309	ENSG00000159409	TNRC4	Hsa1	149941454	ENSCING00000001999		Cin6q	127361
309	ENSG00000101489	BRUNOL4	Hsa18	33077828	ENSCING00000001999		Cin6q	127361
309	ENSG00000161082	BRUNOL5	Hsa19	3175701	ENSCING00000001999		Cin6q	127361
309	ENSG00000155636	DRBP1_HUMAN	Hsa2	178685520	ENSCING00000001999		Cin6q	127361
340	ENSG00000145779	TNFAIP8	Hsa5	118756403	ENSCING00000013803		Cin1p	2308769
340	ENSG00000183578	TNFAIP8L3	Hsa15	49136093	ENSCING00000013803		Cin1p	2308769
340	ENSG00000185361	TNFAIP8L1	Hsa19	4590530	ENSCING00000013803		Cin1p	2308769
340	ENSG00000163154	TNFAIP8L2	Hsa1	149395729	ENSCING00000013803		Cin1p	2308769
362	ENSG00000173171	MTX1	Hsa1	153445114	ENSCING00000013820		Cin1p	1547651
380	ENSG00000143368	SF3B4	Hsa1	148161833	ENSCING00000002337		Cin1p	3675069
409	ENSG00000113384	GOLPH3	Hsa5	32160567	ENSCING00000002602		Cin1p	5869264
409	ENSG00000143457	GOLPH3L	Hsa1	148885325	ENSCING00000002602		Cin1p	5869264
584	ENSG00000143621	ILF2	Hsa1	151900905	ENSCING00000003447	Q4H352_CIOIN	Cinscaffold_163	31796
648	ENSG00000143569	UBAP2L	Hsa1	152459279	ENSCING00000008788		Cin4q	5022821
648	ENSG00000137073	UBAP2	Hsa9	33911691	ENSCING00000008788		Cin4q	5022821
663	ENSG00000198952	SMG5	Hsa1	154485639	ENSCING00000008706		Cin4q	5389672
672	ENSG00000111725	PRKAB1	Hsa12	118590144	ENSCING00000004973		Cin4q	3487981
672	ENSG00000131791	PRKAB2	Hsa1	145093309	ENSCING00000004973		Cin4q	3487981
695	ENSG00000140367	UBE2Q2	Hsa15	73922855	ENSCING00000008673		Cin4q	5742993
695	ENSG00000160714	UBE2Q1	Hsa1	152787675	ENSCING00000008673		Cin4q	5742993
755	ENSG00000058453	CROCC	Hsa1	17049877	ENSCING00000008061		Cin4q	828851
755	ENSG00000126001	CEP250	Hsa20	33506564	ENSCING00000008061		Cin4q	828851
755	ENSG00000144674	GOLGA4	Hsa3	37259742	ENSCING00000008061		Cin4q	828851
755	ENSG00000159450	TCHH	Hsa1	150345417	ENSCING00000008061		Cin4q	828851
766	ENSG00000081923	ATP8B1	Hsa18	53466591	ENSCING00000006046		Cin4q	3332533
766	ENSG00000104043	ATP8B4	Hsa15	47937727	ENSCING00000006046		Cin4q	3332533
766	ENSG00000143515	ATP8B2	Hsa1	152564654	ENSCING00000006046		Cin4q	3332533
766	ENSG00000130270	ATP8B3	Hsa19	1733076	ENSCING00000006046		Cin4q	3332533
766	ENSG00000068650	ATP11A	Hsa13	112392644	ENSCING00000006046		Cin4q	3332533
766	ENSG00000101974	ATP11C	HsaX	138636171	ENSCING00000006046		Cin4q	3332533
780	ENSG00000135018	UBQLN1	Hsa9	85464698	ENSCING00000008783		Cin4q	5040218
780	ENSG00000188021	UBQLN2	HsaX	56606797	ENSCING00000008783		Cin4q	5040218
780	ENSG00000160803	UBQLN4	Hsa1	154271716	ENSCING00000008783		Cin4q	5040218
780	ENSG00000174995	650339	Hsa3	150186338	ENSCING00000008783		Cin4q	5040218
780	ENSG00000175520	UBQLN3	Hsa11	5485106	ENSCING00000008783		Cin4q	5040218
780	ENSG00000175518	NP_659490.3	Hsa11	5492199	ENSCING00000008783		Cin4q	5040218
826	ENSG00000174485	DENND4A	Hsa15	63740596	ENSCING00000005367		Cin4q	6019319
826	ENSG00000198837	DENND4B	Hsa1	152168601	ENSCING00000005367		Cin4q	6019319
826	ENSG00000137145	DENND4C	Hsa9	19286038	ENSCING00000005367		Cin4q	6019319
874	ENSG00000176444	CLK2	Hsa1	153499283	ENSCING00000007926		Cin4q	1312139
874	ENSG00000178340	CLK2P	Hsa7	23591206	ENSCING00000007926		Cin4q	1312139
874	ENSG00000179335	CLK3	Hsa15	72687766	ENSCING00000007926		Cin4q	1312139
874	ENSG00000013441	CLK1	Hsa2	201425978	ENSCING00000007926		Cin4q	1312139
874	ENSG00000113240	CLK4	Hsa5	177962272	ENSCING00000007926		Cin4q	1312139
887	ENSG00000143382	ADAMTSL4	Hsa1	148788508	ENSCING00000009176		Cin4q	6378025
887	ENSG00000197859	ADAMTSL2	Hsa9	135387107	ENSCING00000009176		Cin4q	6378025
937	ENSG00000164434	FABP7	Hsa6	123142319	ENSCING00000002061		Cinscaffold_140	237083
937	ENSG00000170323	FABP4	Hsa8	82553484	ENSCING00000002061		Cinscaffold_140	237083
937	ENSG00000147588	PMP2	Hsa8	82515121	ENSCING00000002061		Cinscaffold_140	237083
937	ENSG00000121769	FABP3	Hsa1	31610687	ENSCING00000002061		Cinscaffold_140	237083
937	ENSG00000164687	FABP5	Hsa8	82355326	ENSCING00000002061		Cinscaffold_140	237083
937	ENSG00000166899	FABPE_HUMAN	Hsa11	59305362	ENSCING00000002061		Cinscaffold_140	237083
937	ENSG00000118928	387934	Hsa13	72572717	ENSCING00000002061		Cinscaffold_140	237083
937	ENSG00000197416	650722	Hsa8	82599863	ENSCING00000002061		Cinscaffold_140	237083
937	ENSG00000205186	Q0Z7S8_HUMAN	Hsa8	82533173	ENSCING00000002061		Cinscaffold_140	237083
937	ENSG00000174104		Hsa13	41841395	ENSCING00000002061		Cinscaffold_140	237083
937	ENSG00000143320	CRABP2	Hsa1	154936022	ENSCING00000002061		Cinscaffold_140	237083
937	ENSG00000166426	CRABP1	Hsa15	76419744	ENSCING00000002061		Cinscaffold_140	237083
937	ENSG00000114115	RBP1	Hsa3	140718970	ENSCING00000002061		Cinscaffold_140	237083
937	ENSG00000114113	RBP2	Hsa3	140654417	ENSCING00000002061		Cinscaffold_140	237083
947	ENSG00000125459	MSTO1	Hsa1	153846631	ENSCING00000008620		Cinscaffold_62	67657
964	ENSG00000138594	TMOD3	Hsa15	49909155	ENSCING00000000042		Cinscaffold_59	491177
964	ENSG00000136842	TMOD1	Hsa9	99303742	ENSCING00000000042		Cinscaffold_59	491177
964	ENSG00000163157	TMOD4	Hsa1	149409087	ENSCING00000000042		Cinscaffold_59	491177
964	ENSG00000128872	TMOD2	Hsa15	49831102	ENSCING00000000042		Cinscaffold_59	491177
964	ENSG00000163380	LMOD3	Hsa3	69250392	ENSCING00000000042		Cinscaffold_59	491177

989	ENSG00000153147	SMARCA5	Hsa4	144654066	ENSCING00000004110	Q4H2Q8_CIOIN	Cinscaffold_106	208779
989	ENSG00000102038	SMARCA1	HsaX	128408163	ENSCING00000004110	Q4H2Q8_CIOIN	Cinscaffold_106	208779
989	ENSG00000131778	CHD1L	Hsa1	145180958	ENSCING00000004110	Q4H2Q8_CIOIN	Cinscaffold_106	208779
994	ENSG00000196189	SEMA4A	Hsa1	154386434	ENSCING00000014403		Cin14q	2146733
994	ENSG00000075213	SEMA3A	Hsa7	83428426	ENSCING00000014403		Cin14q	2146733
994	ENSG00000012171	SEMA3B	Hsa3	50280044	ENSCING00000014403		Cin14q	2146733
994	ENSG00000170381	SEMA3E	Hsa7	82831158	ENSCING00000014403		Cin14q	2146733
994	ENSG00000075223	SEMA3C	Hsa7	80209791	ENSCING00000014403		Cin14q	2146733
994	ENSG00000153993	SEMA3D	Hsa7	84462812	ENSCING00000014403		Cin14q	2146733
1013	ENSG00000143549	TPM3	Hsa1	152395457	ENSCING00000002349	TPM1_CIOIN	Cin14q	3018755
1013	ENSG00000198467	TPM2	Hsa9	35671989	ENSCING00000002349	TPM1_CIOIN	Cin14q	3018755
1013	ENSG00000140416	TPM1	Hsa15	61121891	ENSCING00000002349	TPM1_CIOIN	Cin14q	3018755
1013	ENSG00000167460	TPM4	Hsa19	16039348	ENSCING00000002349	TPM1_CIOIN	Cin14q	3018755
1013	ENSG00000187536		Hsa16	51246717	ENSCING00000002349	TPM1_CIOIN	Cin14q	3018755
1015	ENSG00000159164	SV2A	Hsa1	148141494	ENSCING00000000453		Cin14q	254515
1015	ENSG00000122012	SV2C	Hsa5	75463332	ENSCING00000000453		Cin14q	254515
1015	ENSG00000185518	SV2B	Hsa15	89570104	ENSCING00000000453		Cin14q	254515
1226	ENSG00000143365	LRRN6D	Hsa1	150039364	ENSCING00000002152	Q4H2V2_CIOIN	Cin3p	1346821
1234	ENSG00000143418	LASS2	Hsa1	149204286	ENSCING00000007135	Q4H395_CIOIN	Cin3p	1259787
1234	ENSG00000139624	LASS5	Hsa12	48809849	ENSCING00000007135	Q4H395_CIOIN	Cin3p	1259787
1234	ENSG00000090661	LASS4	Hsa19	8180253	ENSCING00000007135	Q4H395_CIOIN	Cin3p	1259787
1234	ENSG00000154227	LASS3	Hsa15	98758124	ENSCING00000007135	Q4H395_CIOIN	Cin3p	1259787
1254	ENSG00000117360	PRPF3	Hsa1	148560549	ENSCING00000003312		Cin3p	88209
1282	ENSG00000067715	SYT1	Hsa12	77782685	ENSCING00000006816		Cinscaffold_198	20608
1282	ENSG00000143858	SYT2	Hsa1	200826347	ENSCING00000006816		Cinscaffold_198	20608
1282	ENSG00000129990	SYT5	Hsa19	60376281	ENSCING00000006816		Cinscaffold_198	20608
1282	ENSG00000134207	SYT6	Hsa1	114433437	ENSCING00000006816		Cinscaffold_198	20608
1282	ENSG00000110975	SYT10	Hsa12	33419627	ENSCING00000006816		Cinscaffold_198	20608
1282	ENSG00000170743	SYT9	Hsa11	7229757	ENSCING00000006816		Cinscaffold_198	20608
1282	ENSG00000149043	SYT8	Hsa11	1808893	ENSCING00000006816		Cinscaffold_198	20608
1282	ENSG00000161681	SHANK1	Hsa19	55817048	ENSCING00000006816		Cinscaffold_198	20608
1282	ENSG00000132872	SYT4	Hsa18	39101857	ENSCING00000006816		Cinscaffold_198	20608
1282	ENSG00000103528	SYT17	Hsa16	19087139	ENSCING00000006816		Cinscaffold_198	20608
1282	ENSG00000132718	SYT11	Hsa1	154095924	ENSCING00000006816		Cinscaffold_198	20608
1282	ENSG00000204176	SYT15	Hsa10	46372768	ENSCING00000006816		Cinscaffold_198	20608
1282	ENSG00000173227	SYT12	Hsa11	66530825	ENSCING00000006816		Cinscaffold_198	20608
1282	ENSG00000019505	SYT13	Hsa11	45218428	ENSCING00000006816		Cinscaffold_198	20608
1308	ENSG00000068305	MEF2A	Hsa15	97923712	ENSCING00000000628	Q4H375_CIOIN	Cinscaffold_187	72757
1308	ENSG00000081189	MEF2C	Hsa5	88051922	ENSCING00000000628	Q4H375_CIOIN	Cinscaffold_187	72757
1308	ENSG00000116604	MEF2D	Hsa1	154700143	ENSCING00000000628	Q4H375_CIOIN	Cinscaffold_187	72757
1308	ENSG00000064489	MEF2B	Hsa19	19117379	ENSCING00000000628	Q4H375_CIOIN	Cinscaffold_187	72757
1397	ENSG00000163159	VPS72	Hsa1	149415404	ENSCING00000014430	Q4H2Q4_CIOIN	Cinscaffold_181	137598
1436	ENSG00000169241	RAG1AP1	Hsa1	153374444	ENSCING00000005468		Cin10q	2224293
1438	ENSG00000099822	HCN2	Hsa19	540893	ENSCING00000005528		Cin10q	1167144
1438	ENSG00000164588	HCN1	Hsa5	45297730	ENSCING00000005528		Cin10q	1167144
1438	ENSG00000143630	HCN3	Hsa1	153513998	ENSCING00000005528		Cin10q	1167144
1441	ENSG00000085365	SCAMP1	Hsa5	77692110	ENSCING00000010498		Cin10q	3036034
1441	ENSG00000140497	SCAMP2	Hsa15	72924249	ENSCING00000010498		Cin10q	3036034
1441	ENSG00000116521	SCAMP3	Hsa1	153492394	ENSCING00000010498		Cin10q	3036034
1441	ENSG00000198794	SCAMP5	Hsa15	73074958	ENSCING00000010498		Cin10q	3036034
1498	ENSG00000129946	SHC2	Hsa19	367593	ENSCING00000006400		Cin10q	2884248
1498	ENSG00000148082	SHC3	Hsa9	90817880	ENSCING00000006400		Cin10q	2884248
1498	ENSG00000160691	SHC1	Hsa1	153201398	ENSCING00000006400		Cin10q	2884248
1498	ENSG00000185634	SHC4	Hsa15	46903227	ENSCING00000006400		Cin10q	2884248
1535	ENSG00000160752	FDPS	Hsa1	153545267	ENSCING00000008276		Cin10q	240936
1535	ENSG00000196898	XR_017751.1	Hsa7	76435514	ENSCING00000008276		Cin10q	240936
1535	ENSG00000197470	Q499Z7_HUMAN	Hsa7	75937415	ENSCING00000008276		Cin10q	240936
1651	ENSG00000160785	NP_055470.1	Hsa1	154430502	ENSCING00000007741		Cin1q	7181839
1663	ENSG00000137819	PAQR5	Hsa15	67439358	ENSCING00000007567		Cin1q	4547726
1663	ENSG00000188582	PAQR9	Hsa3	144163735	ENSCING00000007567		Cin1q	4547726
1663	ENSG00000160781	PAQR6	Hsa1	154479830	ENSCING00000007567		Cin1q	4547726
1903	ENSG00000173080	RXFP4	Hsa1	154178125	ENSCING00000012511		Cin1q	4671863
2006	ENSG00000203833	NP_110423.3	Hsa1	146559962	ENSCING00000001568		Cin12p	843897
2075	ENSG00000113368	LMNB1	Hsa5	126140214	ENSCING00000000897	Q9GNN4_CIOIN	Cin12p	905718
2075	ENSG00000160789	LMNA	Hsa1	154318993	ENSCING00000000897	Q9GNN4_CIOIN	Cin12p	905718
2075	ENSG00000176619	LMNB2	Hsa19	2379165	ENSCING00000000897	Q9GNN4_CIOIN	Cin12p	905718
2105	ENSG00000163468	CCT3	Hsa1	154545376	ENSCING00000001433		Cinscaffold_67	525807
2157	ENSG00000117362	APH1A	Hsa1	148504428	ENSCING00000002845		Cin14p	2306106
2157	ENSG00000138613	APH1B	Hsa15	61356844	ENSCING00000002845		Cin14p	2306106
2184	ENSG00000160679	C1orf77	Hsa1	151873149	ENSCING00000014517		Cin14p	2948206
2273	ENSG00000198715	C1orf85	Hsa1	154529103	ENSCING00000001029		Cinscaffold_199	124849
2316	ENSG00000143416	SELENBP1	Hsa1	149603402	ENSCING00000004957		Cinscaffold_152	59555
2333	ENSG00000160688	FLAD1	Hsa1	153222441	ENSCING00000001147		Cinscaffold_156	171635
2339	ENSG00000169418	NPR1	Hsa1	151917737	ENSCING00000001143		Cinscaffold_156	158889
2339	ENSG00000159899	NPR2	Hsa9	35782151	ENSCING00000001143		Cinscaffold_156	158889
2339	ENSG00000101890	GUCY2F	HsaX	108502791	ENSCING00000001143		Cinscaffold_156	158889
2339	ENSG00000132518	GUCY2D	Hsa17	7846713	ENSCING00000001143		Cinscaffold_156	158889
2344	ENSG00000185130	HIST1H2BL	Hsa6	27883201	ENSCING00000017446		Cinscaffold_7047	1
2344	ENSG00000184678	HIST2H2BE	Hsa1	148122633	ENSCING00000017446		Cinscaffold_7047	1
2344	ENSG00000196331	HIST1H2BO	Hsa6	27969182	ENSCING00000017446		Cinscaffold_7047	1
2344	ENSG00000196374	HIST1H2BM	Hsa6	27890801	ENSCING00000017446		Cinscaffold_7047	1
2344	ENSG00000197846	HIST1H2BF	Hsa6	26307727	ENSCING00000017446		Cinscaffold_7047	1
2344	ENSG00000158373	HIST1H2BD	Hsa6	26266328	ENSCING00000017446		Cinscaffold_7047	1
2344	ENSG00000197697	HIST1H2BE	Hsa6	26292002	ENSCING00000017446		Cinscaffold_7047	1
2344	ENSG00000187990	HIST1H2BG	Hsa6	26324433	ENSCING00000017446		Cinscaffold_7047	1
2344	ENSG00000180596	HIST1H2BC	Hsa6	26222612	ENSCING00000017446		Cinscaffold_7047	1

2344	ENSG00000168242	HIST1H2BI	Hsa6	26381183	ENSCING00000017446		Cinscaffold_7047	1
2344	ENSG00000197459	HIST1H2BH	Hsa6	26359858	ENSCING00000017446		Cinscaffold_7047	1
2344	ENSG00000196226	HIST1H2BB	Hsa6	26151484	ENSCING00000017446		Cinscaffold_7047	1
2344	ENSG00000196501	HIST1H2BN	Hsa6	27913517	ENSCING00000017446		Cinscaffold_7047	1
2344	ENSG00000197435	HIST3H2BB	Hsa1	226712431	ENSCING00000017446		Cinscaffold_7047	1
2344	ENSG00000124635	HIST1H2BJ	Hsa6	27207560	ENSCING00000017446		Cinscaffold_7047	1
2394	ENSG00000132677	RHBG	Hsa1	154605627	ENSCING00000002595	Q6XV77_CIOIN	Cinscaffold_161	48615
2394	ENSG00000112077	RHAG	Hsa6	49680830	ENSCING00000002595	Q6XV77_CIOIN	Cinscaffold_161	48615
2420	ENSG00000143363	PRUNE	Hsa1	149247577	ENSCING00000003006		Cinscaffold_55	572929
2420	ENSG00000156035	C9orf65	Hsa9	78593080	ENSCING00000003006		Cinscaffold_55	572929
2422	ENSG00000167654	ATCAY	Hsa19	3831672	ENSCING00000016431		Cinscaffold_55	562345
2422	ENSG00000140299	BNIP2	Hsa15	57742356	ENSCING00000016431		Cinscaffold_55	562345
2422	ENSG00000163141	BNIPL	Hsa1	149275670	ENSCING00000016431		Cinscaffold_55	562345
2793	ENSG00000159352	PSMD4	Hsa1	149493803	ENSCING00000006425		Cin2q	4997000
2801	ENSG00000107362	C9orf77	Hsa9	73667188	ENSCING00000008539		Cin2q	4528155
2801	ENSG00000182556		Hsa22	19352115	ENSCING00000008539		Cin2q	4528155
2801	ENSG00000182502	150207	Hsa22	20799236	ENSCING00000008539		Cin2q	4528155
2801	ENSG00000136379	Q6PCB6_HUMAN	Hsa15	78774737	ENSCING00000008539		Cin2q	4528155
2801	ENSG00000129968	FAM108A1	Hsa19	1827976	ENSCING00000008539		Cin2q	4528155
2801	ENSG00000203835	FAM108A2	Hsa1	146084969	ENSCING00000008539		Cin2q	4528155
2801	ENSG00000198658	Q5RGM9_HUMAN	Hsa1	144787932	ENSCING00000008539		Cin2q	4528155
2801	ENSG00000203704	648359	Hsa1	212845423	ENSCING00000008539		Cin2q	4528155
2833	ENSG00000080561	MID2	HsaX	106955765	ENSCING00000008591		Cin2q	4267974
2833	ENSG00000101871	MID1	HsaX	10373596	ENSCING00000008591		Cin2q	4267974
2833	ENSG00000152503	TRIM36	Hsa5	114488377	ENSCING00000008591		Cin2q	4267974
2833	ENSG00000119283	TRIM67	Hsa1	229365297	ENSCING00000008591		Cin2q	4267974
2833	ENSG00000100505	TRIM9	Hsa14	50511738	ENSCING00000008591		Cin2q	4267974
2833	ENSG00000163462	TRIM46	Hsa1	153412978	ENSCING00000008591		Cin2q	4267974
2864	ENSG00000067225	PKM2	Hsa15	70278424	ENSCING00000004140		Cin2q	270569
2864	ENSG00000143627	PKLR	Hsa1	153526254	ENSCING00000004140		Cin2q	270569
2956	ENSG00000136943	CTSL2	Hsa9	98834761	ENSCING00000007675		Cin12q	989473
2956	ENSG00000163131	CTSS	Hsa1	148969175	ENSCING00000007675		Cin12q	989473
2986	ENSG00000143612	C1orf43	Hsa1	152445807	ENSCING00000014051		Cin12q	3880917
3115	ENSG00000136854	STXBP1	Hsa9	129414365	ENSCING00000002400		Cinscaffold_273	42490
3115	ENSG00000076944	STXBP2	Hsa19	7608010	ENSCING00000002400		Cinscaffold_273	42490
3115	ENSG00000116266	STXBP3	Hsa1	109090831	ENSCING00000002400		Cinscaffold_273	42490
3115	ENSG00000136631	VPS45A	Hsa1	148305993	ENSCING00000002400		Cinscaffold_273	42490
3189	ENSG00000197381	ADARB1	Hsa21	45318943	ENSCING00000001001		Cinscaffold_137	285311
3189	ENSG00000185736	ADARB2	Hsa10	1218073	ENSCING00000001001		Cinscaffold_137	285311
3189	ENSG00000160710	ADAR	Hsa1	152821161	ENSCING00000001001		Cinscaffold_137	285311
3189	ENSG00000164113	NP_640336.1	Hsa4	123519618	ENSCING00000001001		Cinscaffold_137	285311
3290	ENSG00000203782	LOR	Hsa1	151498800	ENSCING00000007371		Cin8q	4064453
3365	ENSG00000140284	SLC27A2	Hsa15	48261716	ENSCING00000004468		Cin8q	2484251
3365	ENSG00000113396	SLC27A6	Hsa5	128328720	ENSCING00000004468		Cin8q	2484251
3365	ENSG00000083807	SLC27A5	Hsa19	63701516	ENSCING00000004468		Cin8q	2484251
3365	ENSG00000143554	SLC27A3	Hsa1	152013454	ENSCING00000004468		Cin8q	2484251
3604	ENSG00000180573	HIST1H2AC	Hsa6	26232352	ENSCING00000010029		Cin10p	1603512
3604	ENSG00000111332	H2AFJ	Hsa12	14818672	ENSCING00000010029		Cin10p	1603512
3604	ENSG00000184270	HIST2H2AB	Hsa1	148125698	ENSCING00000010029		Cin10p	1603512
3604	ENSG00000184260	HIST2H2AC	Hsa1	148125149	ENSCING00000010029		Cin10p	1603512
3604	ENSG00000183558	HIST2H2AA4	Hsa1	148080129	ENSCING00000010029		Cin10p	1603512
3604	ENSG00000203812	H2A2A_HUMAN	Hsa1	148089252	ENSCING00000010029		Cin10p	1603512
3604	ENSG00000181218	HIST3H2A	Hsa1	226711303	ENSCING00000010029		Cin10p	1603512
3604	ENSG00000184348	HIST1H2AG	Hsa6	27913704	ENSCING00000010029		Cin10p	1603512
3604	ENSG00000184825	H2A1H_HUMAN	Hsa6	27222840	ENSCING00000010029		Cin10p	1603512
3604	ENSG00000196787	H2A1_HUMAN	Hsa6	27208800	ENSCING00000010029		Cin10p	1603512
3604	ENSG00000182611	HIST1H2AJ	Hsa6	27890111	ENSCING00000010029		Cin10p	1603512
3604	ENSG00000196866	HIST1H2AM	Hsa6	27968514	ENSCING00000010029		Cin10p	1603512
3604	ENSG00000198374	HIST1H2AL	Hsa6	27941086	ENSCING00000010029		Cin10p	1603512
3678	ENSG00000131781	FMO5	Hsa1	145124462	ENSCING00000005517		Cin9p	2534230
3678	ENSG00000094963	FMO2	Hsa1	169420971	ENSCING00000005517		Cin9p	2534230
3678	ENSG00000007933	FMO3	Hsa1	169326642	ENSCING00000005517		Cin9p	2534230
3678	ENSG00000117507	FMO6	Hsa1	169373503	ENSCING00000005517		Cin9p	2534230
3678	ENSG00000076258	FMO4	Hsa1	169549997	ENSCING00000005517		Cin9p	2534230
3678	ENSG00000010932	FMO1	Hsa1	169484262	ENSCING00000005517		Cin9p	2534230
3678	ENSG00000143151	Q5VT49_HUMAN	Hsa1	164840618	ENSCING00000005517		Cin9p	2534230
3685	ENSG00000132676	DAP3	Hsa1	153925473	ENSCING00000003252		Cin9p	2284265
3793	ENSG00000198618	PPIA	Hsa21	19151917	ENSCING00000014306		Cin9p	1132607
3793	ENSG00000196262	PPIA_HUMAN	Hsa7	44802804	ENSCING00000014306		Cin9p	1132607
3793	ENSG00000174715		Hsa5	81341171	ENSCING00000014306		Cin9p	1132607
3793	ENSG00000176992	390299	Hsa11	3870199	ENSCING00000014306		Cin9p	1132607
3793	ENSG00000197781	XR_018829.1	Hsa2	11409204	ENSCING00000014306		Cin9p	1132607
3793	ENSG00000168777	390006	Hsa10	116439892	ENSCING00000014306		Cin9p	1132607
3793	ENSG00000203847	XR_016130.1	Hsa1	142555943	ENSCING00000014306		Cin9p	1132607
3793	ENSG00000188905	342541	Hsa17	58902697	ENSCING00000014306		Cin9p	1132607
3793	ENSG00000198936	NP_839944.1	Hsa1	147819281	ENSCING00000014306		Cin9p	1132607
3793	ENSG00000198161	PPIAL4	Hsa1	146418535	ENSCING00000014306		Cin9p	1132607
3793	ENSG00000198360	NP_839944.1	Hsa1	143072096	ENSCING00000014306		Cin9p	1132607
3793	ENSG00000203826	728945	Hsa1	147072639	ENSCING00000014306		Cin9p	1132607
3793	ENSG00000203829	728945	Hsa1	146910635	ENSCING00000014306		Cin9p	1132607
3825	ENSG00000125249	RAP2A	Hsa13	96884475	ENSCING00000001232		Cinscaffold_92	56086
3825	ENSG00000123728	RAP2C	HsaX	131164734	ENSCING00000001232		Cinscaffold_92	56086
3825	ENSG00000181467	RAP2B	Hsa3	154362719	ENSCING00000001232		Cinscaffold_92	56086
3825	ENSG00000127314	RAP1B	Hsa12	67290919	ENSCING00000001232		Cinscaffold_92	56086
3825	ENSG00000116473	RAP1A	Hsa1	111886363	ENSCING00000001232		Cinscaffold_92	56086
3825	ENSG00000176276	643752	Hsa5	75501667	ENSCING00000001232		Cinscaffold_92	56086

3825	ENSG00000133703	KRAS	Hsa12	25249449	ENSCING00000001232		Cinscaffold_92	56086
3825	ENSG00000174775	HRAS	Hsa11	522243	ENSCING00000001232		Cinscaffold_92	56086
3825	ENSG00000143622	RIT1	Hsa1	154134223	ENSCING00000001232		Cinscaffold_92	56086
3825	ENSG00000152214	RIT2	Hsa18	38577191	ENSCING00000001232		Cinscaffold_92	56086
3825	ENSG00000126458	RRAS	Hsa19	54830364	ENSCING00000001232		Cinscaffold_92	56086
3837	ENSG00000084733	RAB10	Hsa2	26110483	ENSCING00000003959		Cinscaffold_92	184881
3837	ENSG00000166128	RAB8B	Hsa15	61268781	ENSCING00000003959		Cinscaffold_92	184881
3837	ENSG00000167461	RAB8A	Hsa19	16083467	ENSCING00000003959		Cinscaffold_92	184881
3837	ENSG00000143545	RAB13	Hsa1	152220751	ENSCING00000003959		Cinscaffold_92	184881
3837	ENSG00000205319	649713	Hsa12	54660473	ENSCING00000003959		Cinscaffold_92	184881
3837	ENSG00000138069	RAB1A	Hsa2	65167493	ENSCING00000003959		Cinscaffold_92	184881
3837	ENSG00000172794	RAB37	Hsa17	70178312	ENSCING00000003959		Cinscaffold_92	184881
3837	ENSG00000152932	RAB3C	Hsa5	57914671	ENSCING00000003959		Cinscaffold_92	184881
3837	ENSG00000167964	RAB26	Hsa16	2138646	ENSCING00000003959		Cinscaffold_92	184881
3837	ENSG00000105649	RAB3A	Hsa19	18168611	ENSCING00000003959		Cinscaffold_92	184881
3837	ENSG00000105514	RAB3D	Hsa19	11296094	ENSCING00000003959		Cinscaffold_92	184881
3837	ENSG00000169213	RAB3B	Hsa1	52146216	ENSCING00000003959		Cinscaffold_92	184881
4006	ENSG00000198726	648822	Hsa1	153387644	ENSCING00000009334		Cin5q	2734960
4018	ENSG00000143437	ARNT	Hsa1	149048810	ENSCING00000008102	Q4H3W4_CIOIN	Cin5q	4437703
4018	ENSG00000172379	ARNT2	Hsa15	78483747	ENSCING00000008102	Q4H3W4_CIOIN	Cin5q	4437703
4018	ENSG00000133794	ARNTL	Hsa11	13255921	ENSCING00000008102	Q4H3W4_CIOIN	Cin5q	4437703
4018	ENSG00000029153	ARNTL2	Hsa12	27377255	ENSCING00000008102	Q4H3W4_CIOIN	Cin5q	4437703
4093	ENSG00000198558	HIST1H4L	Hsa6	27948956	ENSCING00000015343		Cinscaffold_736	586
4093	ENSG00000198339	HIST1H4I	Hsa6	27214346	ENSCING00000015343		Cinscaffold_736	586
4093	ENSG00000197238	HIST1H4J	Hsa6	27899863	ENSCING00000015343		Cinscaffold_736	586
4093	ENSG00000182217	H4_HUMAN	Hsa1	148097376	ENSCING00000015343		Cinscaffold_736	586
4093	ENSG00000183941	HIST2H4A	Hsa1	148070866	ENSCING00000015343		Cinscaffold_736	586
4093	ENSG00000198518	HIST4H4	Hsa6	26312817	ENSCING00000015343		Cinscaffold_736	586
4093	ENSG00000188987	HIST1H4D	Hsa6	26296972	ENSCING00000015343		Cinscaffold_736	586
4093	ENSG00000196176	HIST1H4A	Hsa6	26129886	ENSCING00000015343		Cinscaffold_736	586
4093	ENSG00000197061	HIST1H4C	Hsa6	26212105	ENSCING00000015343		Cinscaffold_736	586
4093	ENSG00000158406	HIST1H4H	Hsa6	26389262	ENSCING00000015343		Cinscaffold_736	586
4093	ENSG00000198327	HIST1H4F	Hsa6	26348540	ENSCING00000015343		Cinscaffold_736	586
4093	ENSG00000124529	HIST1H4B	Hsa6	26135148	ENSCING00000015343		Cinscaffold_736	586
4093	ENSG00000197837	H4_HUMAN	Hsa12	14814974	ENSCING00000015343		Cinscaffold_736	586
4093	ENSG00000197914	HIST1H4K	Hsa6	27906973	ENSCING00000015343		Cinscaffold_736	586
4093	ENSG00000124578	HIST1H4G	Hsa6	26354888	ENSCING00000015343		Cinscaffold_736	586
4103	ENSG00000080709	KCNN2	Hsa5	113725565	ENSCING00000009747		Cinscaffold_44	615987
4103	ENSG00000143603	KCNN3	Hsa1	152946526	ENSCING00000009747		Cinscaffold_44	615987
4103	ENSG00000105642	KCNN1	Hsa19	17945647	ENSCING00000009747		Cinscaffold_44	615987
4103	ENSG00000104783	KCNN4	Hsa19	48962578	ENSCING00000009747		Cinscaffold_44	615987
4106	ENSG00000121743	GJA3	Hsa13	19614120	ENSCING00000014820		Cinscaffold_44	145508
4106	ENSG00000121634	GJA8	Hsa1	145841560	ENSCING00000014820		Cinscaffold_44	145508
4106	ENSG00000165474	GJB2	Hsa13	19659609	ENSCING00000014820		Cinscaffold_44	145508
4106	ENSG00000121742	GJB6	Hsa13	19694103	ENSCING00000014820		Cinscaffold_44	145508
4106	ENSG00000169562	GJB1	HsaX	70351769	ENSCING00000014820		Cinscaffold_44	145508
4106	ENSG00000143140	GJA5	Hsa1	145695517	ENSCING00000014820		Cinscaffold_44	145508
4106	ENSG00000152661	GJA1	Hsa6	121798487	ENSCING00000014820		Cinscaffold_44	145508
4106	ENSG00000183153	GJC1	Hsa17	35770761	ENSCING00000014820		Cinscaffold_44	145508
4106	ENSG00000176857		Hsa5	108414912	ENSCING00000014820		Cinscaffold_44	145508
4122	ENSG00000140575	IQGAP1	Hsa15	88732477	ENSCING00000000781	NP_001027628	Cinscaffold_44	711863
4122	ENSG00000183856	IQGAP3	Hsa1	154761821	ENSCING00000000781	NP_001027628	Cinscaffold_44	711863
4122	ENSG00000145703	IQGAP2	Hsa5	75734905	ENSCING00000000781	NP_001027628	Cinscaffold_44	711863
4169	ENSG00000172115	CYCS	Hsa7	25124802	ENSCING00000012495		Cin3q	4562442
4169	ENSG00000198962	CYCSP52	Hsa1	149142996	ENSCING00000012495		Cin3q	4562442
4169	ENSG00000184844		Hsa8	33946621	ENSCING00000012495		Cin3q	4562442
4169	ENSG00000186709		Hsa16	25373161	ENSCING00000012495		Cin3q	4562442
4169	ENSG00000188512		Hsa13	23872841	ENSCING00000012495		Cin3q	4562442
4169	ENSG00000174440		Hsa2	100353051	ENSCING00000012495		Cin3q	4562442
4242	ENSG00000140350	ANP32A	Hsa15	66845408	ENSCING00000012732		Cin3q	2052285
4242	ENSG00000136938	ANP32B	Hsa9	99785462	ENSCING00000012732		Cin3q	2052285
4242	ENSG00000143401	ANP32E	Hsa1	148457341	ENSCING00000012732		Cin3q	2052285
4242	ENSG00000184701	ANP32C	Hsa4	165337609	ENSCING00000012732		Cin3q	2052285
4242	ENSG00000139223	ANP32D	Hsa12	47152715	ENSCING00000012732		Cin3q	2052285
4328	ENSG00000107242	PIP5K1B	Hsa9	70510436	ENSCING00000009558		Cin3q	1417104
4328	ENSG00000186111	PIP5K1C	Hsa19	3581182	ENSCING00000009558		Cin3q	1417104
4328	ENSG00000143398	PIP5K1A	Hsa1	149437651	ENSCING00000009558		Cin3q	1417104
4328	ENSG00000173780	Q8TBY6_HUMAN	Hsa6	7931335	ENSCING00000009558		Cin3q	1417104
4366	ENSG00000185236	RAB11B	Hsa19	8361229	ENSCING00000009526		Cin3q	1093338
4366	ENSG00000103769	RAB11A	Hsa15	63948850	ENSCING00000009526		Cin3q	1093338
4366	ENSG00000132698	RAB25	Hsa1	154297575	ENSCING00000009526		Cin3q	1093338
4377	ENSG00000135047	CTSL	Hsa9	89530254	ENSCING00000012592		Cin3q	3649028
4377	ENSG00000143387	CTSK	Hsa1	149035308	ENSCING00000012592		Cin3q	3649028
4385	ENSG00000184349	EFNA5	Hsa5	106740489	ENSCING00000005716	Q4H3L9_CIOIN	Cin3q	1758928
4385	ENSG00000143590	EFNA3	Hsa1	153317972	ENSCING00000005716	Q4H3L9_CIOIN	Cin3q	1758928
4399	ENSG00000116586	MAPIP_HUMAN	Hsa1	154291167	ENSCING00000012787		Cin3q	1039442
4502	ENSG00000165272	AQP3	Hsa9	33431152	ENSCING00000009588		Cin9q	2688529
4502	ENSG00000143595	AQP10	Hsa1	152560180	ENSCING00000009588		Cin9q	2688529
4502	ENSG00000103569	AQP9	Hsa15	56217771	ENSCING00000009588		Cin9q	2688529
4502	ENSG00000165269	AQP7	Hsa9	33374948	ENSCING00000009588		Cin9q	2688529
4502	ENSG00000181997		Hsa9	42848068	ENSCING00000009588		Cin9q	2688529
4502	ENSG00000176115		Hsa9	66770881	ENSCING00000009588		Cin9q	2688529
4502	ENSG00000186466	AQP7P2	Hsa9	66961803	ENSCING00000009588		Cin9q	2688529
4548	ENSG00000143303	C1orf66	Hsa1	154964858	ENSCING00000012865		Cin9q	3825567
4661	ENSG00000138279	ANXA7	Hsa10	74805209	ENSCING00000004236		Cin9q	155680
4661	ENSG00000122359	ANXA11	Hsa10	81900625	ENSCING00000004236		Cin9q	155680

4661	ENSG00000196975	ANXA4	Hsa2	69801427	ENSCING00000004236		Cin9q	155680
4661	ENSG00000138772	ANXA3	Hsa4	79694613	ENSCING00000004236		Cin9q	155680
4661	ENSG00000164111	ANXA5	Hsa4	122808598	ENSCING00000004236		Cin9q	155680
4661	ENSG00000104537	ANXA13	Hsa8	124762216	ENSCING00000004236		Cin9q	155680
4661	ENSG00000165390	ANXA8	Hsa10	47875259	ENSCING00000004236		Cin9q	155680
4661	ENSG00000150165	ANXA8L1	Hsa10	46577989	ENSCING00000004236		Cin9q	155680
4661	ENSG00000186807	ANXA8L2	Hsa10	47216942	ENSCING00000004236		Cin9q	155680
4661	ENSG00000135046	ANXA1	Hsa9	74956493	ENSCING00000004236		Cin9q	155680
4661	ENSG00000182718	ANXA2	Hsa15	58426643	ENSCING00000004236		Cin9q	155680
4661	ENSG00000183059	ANXA2P2	Hsa9	33614223	ENSCING00000004236		Cin9q	155680
4661	ENSG00000109511	ANXA10	Hsa4	169250310	ENSCING00000004236		Cin9q	155680
4661	ENSG00000143412	ANXA9	Hsa1	149221178	ENSCING00000004236		Cin9q	155680
4690	ENSG00000143624	INTS3	Hsa1	151967167	ENSCING00000002537		Cinscaffold_153	162400
4709	ENSG00000183598	HIST2H3D	Hsa1	148051450	ENSCING00000002753	Q6TXQ3_CIOIN	Cinscaffold_47	727136
4709	ENSG00000203813	HIST2H3C	Hsa1	148077734	ENSCING00000002753	Q6TXQ3_CIOIN	Cinscaffold_47	727136
4709	ENSG00000203811	HIST2H3A	Hsa1	148090805	ENSCING00000002753	Q6TXQ3_CIOIN	Cinscaffold_47	727136
4709	ENSG00000196747	HIST1H2AI	Hsa6	27883949	ENSCING00000002753	Q6TXQ3_CIOIN	Cinscaffold_47	727136
4709	ENSG00000197409	HIST1H3D	Hsa6	26305047	ENSCING00000002753	Q6TXQ3_CIOIN	Cinscaffold_47	727136
4709	ENSG00000197153	HIST1H3J	Hsa6	27966139	ENSCING00000002753	Q6TXQ3_CIOIN	Cinscaffold_47	727136
4709	ENSG00000182572	HIST1H3I	Hsa6	27947662	ENSCING00000002753	Q6TXQ3_CIOIN	Cinscaffold_47	727136
4709	ENSG00000198366	H31_HUMAN	Hsa6	26128697	ENSCING00000002753	Q6TXQ3_CIOIN	Cinscaffold_47	727136
4709	ENSG00000178458	HIST1H3A	Hsa6	26379181	ENSCING00000002753	Q6TXQ3_CIOIN	Cinscaffold_47	727136
4709	ENSG00000196532	HIST1H3C	Hsa6	26153591	ENSCING00000002753	Q6TXQ3_CIOIN	Cinscaffold_47	727136
4709	ENSG00000112727	HIST1H3F	Hsa6	26358402	ENSCING00000002753	Q6TXQ3_CIOIN	Cinscaffold_47	727136
4709	ENSG00000124693	HIST1H3B	Hsa6	26139857	ENSCING00000002753	Q6TXQ3_CIOIN	Cinscaffold_47	727136
4709	ENSG00000196966	HIST1H3E	Hsa6	26333362	ENSCING00000002753	Q6TXQ3_CIOIN	Cinscaffold_47	727136
4709	ENSG00000132475	H3F3B	Hsa17	71284112	ENSCING00000002753	Q6TXQ3_CIOIN	Cinscaffold_47	727136
4709	ENSG00000163041	H3F3A	Hsa1	224316175	ENSCING00000002753	Q6TXQ3_CIOIN	Cinscaffold_47	727136
4767	ENSG00000143450	OAZ3	Hsa1	150002069	ENSCING00000011954		Cin7q	3488254
4908	ENSG00000159377	PSMB4	Hsa1	149610623	ENSCING00000002321		Cin7q	7005268
4908	ENSG00000126067	PSMB2	Hsa1	35808000	ENSCING00000002321		Cin7q	7005268

Genomic distribution of the paralog groups of the surrounding 10-Mb window in the ALDH1A-related GN in Hsa19, using the Brachiostoma floridae genomic database as the outgroup for the best reciprocal BLAST hit analysis								
Genomic regiona analyzed: human chromosome 19 0 - 20000 KB								
Group ID	Query Gene ID	Query Ext ID	Query Chromosome	Query Start Base	Outg Gene ID	Outg Ext ID	Outg Chromosome	Outg Start Base
20	ENSG00000104907	TRMT1	Hsa19	13076721	134752	gw.5.10.1	Bflscaffold_5	4697698
25	ENSG00000147403	RPL10	HsaX	153279765	275426	estExt_gwp.C_50583	BflBf_V2_174	4324218
25	ENSG00000178464	XR_017237.1	Hsa19	12615089	275426	estExt_gwp.C_50583	BflBf_V2_174	4324218
25	ENSG00000165496	RPL10L	Hsa14	46190045	275426	estExt_gwp.C_50583	BflBf_V2_174	4324218
25	ENSG00000174572	389342	Hsa5	167975935	275426	estExt_gwp.C_50583	BflBf_V2_174	4324218
111	ENSG00000130313	PGLS	Hsa19	17483432	115123	estExt_fgenesh2_pm.C_1770004	Bflscaffold_177	433760
112	ENSG00000171444	MCC	Hsa5	112385695	183875	gw.683.24.1	Bflscaffold_683	62288
112	ENSG00000130307	USHBP1	Hsa19	17221849	183875	gw.683.24.1	Bflscaffold_683	62288
114	ENSG00000105289	TJP3	Hsa19	3679374	109387	fgenesh2_pg.scaffold_683000010	Bflscaffold_683	112451
139	ENSG00000160111	CPAMD8	Hsa19	16864758	101769	fgenesh2_pg.scaffold_391000023	BflBf_V2_292	380994
140	ENSG00000148123	NP_060223.2	Hsa9	102830812	101756	fgenesh2_pg.scaffold_391000010	BflBf_V2_292	321824
140	ENSG00000117598	NP_001010861	Hsa1	99128391	101756	fgenesh2_pg.scaffold_391000010	BflBf_V2_292	321824
140	ENSG00000105520	NP_073574.1	Hsa19	11327107	101756	fgenesh2_pg.scaffold_391000010	BflBf_V2_292	321824
205	ENSG00000171942	OR10H2	Hsa19	15699854	111310	fgenesh2_pg.scaffold_873000002	Bflscaffold_873	12075
205	ENSG00000172519	OR10H5	Hsa19	15765859	111310	fgenesh2_pg.scaffold_873000002	Bflscaffold_873	12075
217	ENSG00000127526	SLC35E1	Hsa19	16522666	110100	fgenesh2_pg.scaffold_735000010	Bflscaffold_735	83148
241	ENSG00000165029	ABCA1	Hsa9	106583104	234874	e_gw.318.18.1	Bflscaffold_318	310870
241	ENSG00000198691	ABCA4	Hsa1	94230981	234874	e_gw.318.18.1	Bflscaffold_318	310870
241	ENSG00000064687	ABCA7	Hsa19	992361	234874	e_gw.318.18.1	Bflscaffold_318	310870
244	ENSG00000186526	CYP4F8	Hsa19	15587421	234892	e_gw.318.45.1	Bflscaffold_318	870946
244	ENSG00000142973	CYP4B1	Hsa1	47037305	234892	e_gw.318.45.1	Bflscaffold_318	870946
244	ENSG00000186377	CYP4X1	Hsa1	47261827	234892	e_gw.318.45.1	Bflscaffold_318	870946
261	ENSG00000099622	CIRBP	Hsa19	1220336	71461	fgenesh2_pg.scaffold_33000146	BflBf_V2_42	665459
261	ENSG00000102317	RBM3	HsaX	48317780	71461	fgenesh2_pg.scaffold_33000146	BflBf_V2_42	665459
278	ENSG00000083312	TNPO1	Hsa5	72148171	128305	estExt_fgenesh2_pg.C_3040012	BflBf_V2_22	666721
278	ENSG00000105576	TNPO2	Hsa19	12672967	128305	estExt_fgenesh2_pg.C_3040012	BflBf_V2_22	666721
287	ENSG00000104886	PLEKHJ1	Hsa19	2184155	233460	e_gw.304.44.1	BflBf_V2_22	299210
298	ENSG00000142949	PTPRF	Hsa1	43769134	281312	estExt_gwp.C_1010065	BflBf_V2_127	2071422
298	ENSG00000153707	PTPRD	Hsa9	8304246	281312	estExt_gwp.C_1010065	BflBf_V2_127	2071422
298	ENSG00000105426	PTPRS	Hsa19	5157379	281312	estExt_gwp.C_1010065	BflBf_V2_127	2071422
298	ENSG00000173482	PTPRM	Hsa18	7557817	281312	estExt_gwp.C_1010065	BflBf_V2_127	2071422
305	ENSG00000137094	DNAJB5	Hsa9	34979742	224427	e_gw.101.51.1	BflBf_V2_127	1737375
305	ENSG00000162616	DNAJB4	Hsa1	78243126	224427	e_gw.101.51.1	BflBf_V2_127	1737375
305	ENSG00000132002	DNAJB1	Hsa19	14486582	224427	e_gw.101.51.1	BflBf_V2_127	1737375
319	ENSG00000076984	O14733-2	Hsa19	7874728	99237	fgenesh2_pg.scaffold_337000027	BflBf_V2_240	1395825
352	ENSG00000065268	WDR18	Hsa19	935328	82729	fgenesh2_pg.scaffold_114000069	Bflscaffold_114	1059374
354	ENSG00000125651	GTF2F1	Hsa19	6235811	82771	fgenesh2_pg.scaffold_114000111	BflBf_V2_127	360713
359	ENSG00000178878	APOLD1	Hsa12	12770130	57422	fgenesh2_pm.scaffold_20000003	Bflscaffold_20	893796
359	ENSG00000105671	DDX49	Hsa19	18891494	57422	fgenesh2_pm.scaffold_20000003	Bflscaffold_20	893796
360	ENSG00000148339	SLC25A25	Hsa9	129870300	113822	estExt_fgenesh2_pm.C_200017	BflBf_V2_261	895367
360	ENSG00000085491	SLC25A24	Hsa1	108478965	113822	estExt_fgenesh2_pm.C_200017	BflBf_V2_261	895367
360	ENSG00000125648	SLC25A23	Hsa19	6389747	113822	estExt_fgenesh2_pm.C_200017	BflBf_V2_261	895367
360	ENSG00000181240	NP_775908.1	Hsa19	6377050	113822	estExt_fgenesh2_pm.C_200017	BflBf_V2_261	895367
388	ENSG00000104889	RNASEH2A	Hsa19	12778488	285333	estExt_gwp.C_3780020	BflBf_V2_202	157623
397	ENSG00000118473	SGIP1	Hsa1	66771654	105958	fgenesh2_pg.scaffold_513000012	BflBf_V2_99	958121
397	ENSG00000130475	FCHO1	Hsa19	17726920	105958	fgenesh2_pg.scaffold_513000012	BflBf_V2_99	958121
441	ENSG00000130811	EIF3S4	Hsa19	10086691	283255	estExt_gwp.C_2920029	BflBf_V2_176	184860
446	ENSG00000078579	FGF20	Hsa8	16894049	220214	e_gw.82.114.1	BflBf_V2_132	3395701
446	ENSG00000102678	FGF9	Hsa13	21143170	220214	e_gw.82.114.1	BflBf_V2_132	3395701
446	ENSG00000196468	FGF16	HsaX	76596303	220214	e_gw.82.114.1	BflBf_V2_132	3395701
446	ENSG00000102466	FGF14	Hsa13	101169308	220214	e_gw.82.114.1	BflBf_V2_132	3395701
446	ENSG00000070388	FGF22	Hsa19	590920	220214	e_gw.82.114.1	BflBf_V2_132	3395701
446	ENSG00000114279	FGF12	Hsa3	193342992	220214	e_gw.82.114.1	BflBf_V2_132	3395701
446	ENSG00000129682	FGF13	HsaX	137541401	220214	e_gw.82.114.1	BflBf_V2_132	3395701
446	ENSG00000161958	FGF11	Hsa17	7283413	220214	e_gw.82.114.1	BflBf_V2_132	3395701
481	ENSG00000170144	HNRPA3	Hsa2	177785774	237208	e_gw.343.49.1	Bflscaffold_343	704062
481	ENSG00000135486	HNRPA1	Hsa12	52960755	237208	e_gw.343.49.1	Bflscaffold_343	704062
481	ENSG00000139675	NP_001011725	Hsa13	52089606	237208	e_gw.343.49.1	Bflscaffold_343	704062
481	ENSG00000187999	651179	Hsa19	11637988	237208	e_gw.343.49.1	Bflscaffold_343	704062
481	ENSG00000197029	402562	Hsa7	84450808	237208	e_gw.343.49.1	Bflscaffold_343	704062
481	ENSG00000176757	648210	Hsa5	79690652	237208	e_gw.343.49.1	Bflscaffold_343	704062
481	ENSG00000196682	120364	Hsa11	110163965	237208	e_gw.343.49.1	Bflscaffold_343	704062
481	ENSG00000206228	651179	Hsa18	28246175	237208	e_gw.343.49.1	Bflscaffold_343	704062
481	ENSG00000205412	Q3MIB7_HUMAN	Hsa16	50237230	237208	e_gw.343.49.1	Bflscaffold_343	704062
481	ENSG00000122566	HNRPA2B1	Hsa7	26196459	237208	e_gw.343.49.1	Bflscaffold_343	704062
481	ENSG00000176825		Hsa3	75346391	237208	e_gw.343.49.1	Bflscaffold_343	704062
481	ENSG00000181836	Q5T6S7_HUMAN	Hsa13	65260065	237208	e_gw.343.49.1	Bflscaffold_343	704062
481	ENSG00000177733	HNRPA0	Hsa5	137116737	237208	e_gw.343.49.1	Bflscaffold_343	704062
481	ENSG00000177219	Q65ZQ3_HUMAN	Hsa10	43605032	237208	e_gw.343.49.1	Bflscaffold_343	704062
547	ENSG00000125652	ALKBH7	Hsa19	6323444	262579	estExt_GenewiseH_1.C_410293	Bflscaffold_41	2916967
556	ENSG00000183578	TNFAIP8L3	Hsa15	49136093	59157	fgenesh2_pm.scaffold_113000004	BflBf_V2_119	498136
556	ENSG00000145779	TNFAIP8	Hsa5	118756403	59157	fgenesh2_pm.scaffold_113000004	BflBf_V2_119	498136
556	ENSG00000185361	TNFAIP8L1	Hsa19	4590530	59157	fgenesh2_pm.scaffold_113000004	BflBf_V2_119	498136
556	ENSG00000163154	TNFAIP8L2	Hsa1	149395729	59157	fgenesh2_pm.scaffold_113000004	BflBf_V2_119	498136
561	ENSG00000141985	SH3GL1	Hsa19	4311370	82644	fgenesh2_pg.scaffold_113000077	Bflscaffold_113	1589706
561	ENSG00000107295	SH3GL2	Hsa9	17569121	82644	fgenesh2_pg.scaffold_113000077	Bflscaffold_113	1589706
561	ENSG00000097033	SH3GLB1	Hsa1	86942847	82644	fgenesh2_pg.scaffold_113000077	Bflscaffold_113	1589706
561	ENSG00000148341	SH3GLB2	Hsa9	130810134	82644	fgenesh2_pg.scaffold_113000077	Bflscaffold_113	1589706
564	ENSG00000181781	C19orf19	Hsa19	414360	123195	estExt_fgenesh2_pg.C_1130005	BflBf_V2_119	39161
564	ENSG00000182950	ODF3L1	Hsa15	73803374	123195	estExt_fgenesh2_pg.C_1130005	BflBf_V2_119	39161
667	ENSG00000105676	ARMC6	Hsa19	19005538	124365	estExt_fgenesh2_pg.C_1460027	BflBf_V2_32	6332512
670	ENSG00000104980	TIMM44	Hsa19	7897604	124412	estExt_fgenesh2_pg.C_1460106	Bflscaffold_146	1674778
673	ENSG00000081189	MEF2C	Hsa5	88051922	59582	fgenesh2_pm.scaffold_146000014	BflBf_V2_32	6536355
673	ENSG00000116604	MEF2D	Hsa1	154700143	59582	fgenesh2_pm.scaffold_146000014	BflBf_V2_32	6536355
673	ENSG00000064489	MEF2B	Hsa19	19117379	59582	fgenesh2_pm.scaffold_146000014	BflBf_V2_32	6536355

674	ENSG00000152413	HOMER1	Hsa5	78707505	85919	fgenesh2_pg.scaffold_146000067	BflBf_V2_32	6866652
674	ENSG00000103942	HOMER2	Hsa15	81314792	85919	fgenesh2_pg.scaffold_146000067	BflBf_V2_32	6866652
674	ENSG00000051128	HOMER3	Hsa19	18901047	85919	fgenesh2_pg.scaffold_146000067	BflBf_V2_32	6866652
755	ENSG00000080709	KCNN2	Hsa5	113725565	235511	e_gw.324.15.1	BflBf_V2_169	739377
755	ENSG00000143603	KCNN3	Hsa1	152946526	235511	e_gw.324.15.1	BflBf_V2_169	739377
755	ENSG00000105642	KCNN1	Hsa19	17945647	235511	e_gw.324.15.1	BflBf_V2_169	739377
755	ENSG00000104783	KCNN4	Hsa19	48962578	235511	e_gw.324.15.1	BflBf_V2_169	739377
760	ENSG00000007080	CCDC124	Hsa19	17908217	115788	estExt_fgenesh2_pm.C_3240002	BflBf_V2_169	582947
763	ENSG00000077009	ITGB1BP3	Hsa19	3884101	128651	estExt_fgenesh2_pg.C_3240012	BflBf_V2_169	576995
763	ENSG00000106733	C9orf95	Hsa9	76865309	128651	estExt_fgenesh2_pg.C_3240012	BflBf_V2_169	576995
821	ENSG00000198356	ASNA1	Hsa19	12709306	109002	fgenesh2_pg.scaffold_659000007	Bflscaffold_659	153686
822	ENSG00000152932	RAB3C	Hsa5	57914671	253798	e_gw.659.14.1	BflBf_V2_69	118476
822	ENSG00000105649	RAB3A	Hsa19	18168611	253798	e_gw.659.14.1	BflBf_V2_69	118476
822	ENSG00000169213	RAB3B	Hsa1	52146216	253798	e_gw.659.14.1	BflBf_V2_69	118476
822	ENSG00000105514	RAB3D	Hsa19	11296094	253798	e_gw.659.14.1	BflBf_V2_69	118476
822	ENSG00000152932	RAB3C	Hsa5	57914671	253798	e_gw.659.14.1	BflBf_V2_69	118476
822	ENSG00000105649	RAB3A	Hsa19	18168611	253798	e_gw.659.14.1	BflBf_V2_69	118476
822	ENSG00000169213	RAB3B	Hsa1	52146216	253798	e_gw.659.14.1	BflBf_V2_69	118476
822	ENSG00000105514	RAB3D	Hsa19	11296094	253798	e_gw.659.14.1	BflBf_V2_69	118476
887	ENSG00000105655	NP_057452.1	Hsa19	18406620	126153	estExt_fgenesh2_pg.C_2050079	BflBf_V2_36	4686787
905	ENSG00000205922	ONECUT3	Hsa19	1726154	163102	gw.66.125.1	BflBf_V2_216	331303
919	ENSG00000143398	PIP5K1A	Hsa1	149437651	216408	e_gw.66.2.1	BflBf_V2_216	1914305
919	ENSG00000186111	PIP5K1C	Hsa19	3581182	216408	e_gw.66.2.1	BflBf_V2_216	1914305
919	ENSG00000107242	PIP5K1B	Hsa9	70510436	216408	e_gw.66.2.1	BflBf_V2_216	1914305
919	ENSG00000173780	Q8TBY6_HUMAN	Hsa6	7931335	216408	e_gw.66.2.1	BflBf_V2_216	1914305
926	ENSG00000167461	RAB8A	Hsa19	16083467	114333	estExt_fgenesh2_pm.C_660005	BflBf_V2_216	649805
926	ENSG00000166128	RAB8B	Hsa15	61268781	114333	estExt_fgenesh2_pm.C_660005	BflBf_V2_216	649805
926	ENSG00000143545	RAB13	Hsa1	152220751	114333	estExt_fgenesh2_pm.C_660005	BflBf_V2_216	649805
926	ENSG00000205319	649713	Hsa12	54660473	114333	estExt_fgenesh2_pm.C_660005	BflBf_V2_216	649805
1019	ENSG00000107341	UBE2R2	Hsa9	33807182	91986	fgenesh2_pg.scaffold_216000039	Bflscaffold_216	903467
1019	ENSG00000099804	CDC34	Hsa19	482733	91986	fgenesh2_pg.scaffold_216000039	Bflscaffold_216	903467
1024	ENSG00000138615	CILP	Hsa15	63275398	239827	e_gw.373.1.1	Bflscaffold_373	131814
1024	ENSG00000160161	CILP2	Hsa19	19510057	239827	e_gw.373.1.1	Bflscaffold_373	131814
1027	ENSG00000167654	ATCAY	Hsa19	3831672	239803	e_gw.373.4.1	BflBf_V2_88	1754013
1027	ENSG00000140299	BNIP2	Hsa15	57742356	239803	e_gw.373.4.1	BflBf_V2_88	1754013
1027	ENSG00000163141	BNIPL	Hsa1	149275670	239803	e_gw.373.4.1	BflBf_V2_88	1754013
1105	ENSG00000080511	RDH8	Hsa19	9984925	211870	e_gw.46.199.1	Bflscaffold_46	81116
1138	ENSG00000104883	PEX11G	Hsa19	7447761	288202	estExt_gwp.C_5820021	Bflscaffold_582	320897
1191	ENSG00000129625	REEP5	Hsa5	112239983	57028	fgenesh2_pm.scaffold_6000031	BflBf_V2_159	1051270
1191	ENSG00000115255	REEP6	Hsa19	1442165	57028	fgenesh2_pm.scaffold_6000031	BflBf_V2_159	1051270
1253	ENSG00000107105	ELAVL2	Hsa9	23680102	92208	fgenesh2_pg.scaffold_219000066	BflBf_V2_69	362134
1253	ENSG00000066044	ELAVL1	Hsa19	7933159	92208	fgenesh2_pg.scaffold_219000066	BflBf_V2_69	362134
1253	ENSG00000162374	ELAVL4	Hsa1	50342169	92208	fgenesh2_pg.scaffold_219000066	BflBf_V2_69	362134
1253	ENSG00000196361	ELAVL3	Hsa19	11423143	92208	fgenesh2_pg.scaffold_219000066	BflBf_V2_69	362134
1253	ENSG00000107105	ELAVL2	Hsa9	23680102	92208	fgenesh2_pg.scaffold_219000066	BflBf_V2_69	362134
1253	ENSG00000066044	ELAVL1	Hsa19	7933159	92208	fgenesh2_pg.scaffold_219000066	BflBf_V2_69	362134
1253	ENSG00000162374	ELAVL4	Hsa1	50342169	92208	fgenesh2_pg.scaffold_219000066	BflBf_V2_69	362134
1253	ENSG00000196361	ELAVL3	Hsa19	11423143	92208	fgenesh2_pg.scaffold_219000066	BflBf_V2_69	362134
1254	ENSG00000099795	NDUFB7	Hsa19	14537892	115384	estExt_fgenesh2_pm.C_2190002	Bflscaffold_219	31944
1259	ENSG00000125656	CLPP	Hsa19	6312463	92198	fgenesh2_pg.scaffold_219000056	BflBf_V2_69	526275
1260	ENSG00000129347	NP_075384.2	Hsa19	10524761	92213	fgenesh2_pg.scaffold_219000071	BflBf_V2_69	200729
1264	ENSG00000167774	NDUFA7	Hsa19	8282234	126563	estExt_fgenesh2_pg.C_2190046	Bflscaffold_219	725895
1294	ENSG00000179913	B3GNT3	Hsa19	17766658	139225	gw.259.3.1	BflBf_V2_23	1547934
1321	ENSG00000064932	KIAA0963	Hsa19	1058641	153828	gw.278.27.1	BflBf_V2_18	3434394
1345	ENSG00000187650	NP_001017921	Hsa19	5855885	124114	estExt_fgenesh2_pg.C_1380077	Bflscaffold_138	1387009
1348	ENSG00000180448	HMHA1	Hsa19	1018174	124122	estExt_fgenesh2_pg.C_1380087	BflBf_V2_198	949850
1348	ENSG00000137962	ARHGAP29	Hsa1	94409895	124122	estExt_fgenesh2_pg.C_1380087	BflBf_V2_198	949850
1348	ENSG00000089639	GMIP	Hsa19	19601285	124122	estExt_fgenesh2_pg.C_1380087	BflBf_V2_198	949850
1348	ENSG00000180448	HMHA1	Hsa19	1018174	124122	estExt_fgenesh2_pg.C_1380087	BflBf_V2_198	949850
1348	ENSG00000137962	ARHGAP29	Hsa1	94409895	124122	estExt_fgenesh2_pg.C_1380087	BflBf_V2_198	949850
1348	ENSG00000089639	GMIP	Hsa19	19601285	124122	estExt_fgenesh2_pg.C_1380087	BflBf_V2_198	949850
1349	ENSG00000123154	MORG1_HUMAN	Hsa19	12641567	124121	estExt_fgenesh2_pg.C_1380086	BflBf_V2_198	943044
1354	ENSG00000104897	SF3A2	Hsa19	2187816	85094	fgenesh2_pg.scaffold_138000039	Bflscaffold_138	708678
1373	ENSG00000065243	PKN2	Hsa1	88922493	286497	estExt_gwp.C_4430017	Bflscaffold_443	217806
1373	ENSG00000123143	PKN1	Hsa19	14405135	286497	estExt_gwp.C_4430017	Bflscaffold_443	217806
1373	ENSG00000160447	PKN3	Hsa9	130504623	286497	estExt_gwp.C_4430017	Bflscaffold_443	217806
1375	ENSG00000167114	SLC27A4	Hsa9	130142661	103765	fgenesh2_pg.scaffold_443000016	Bflscaffold_443	358093
1375	ENSG00000130304	SLC27A1	Hsa19	17442350	103765	fgenesh2_pg.scaffold_443000016	Bflscaffold_443	358093
1442	ENSG00000173926	MARCH3	Hsa5	126233454	169531	gw.71.205.1	BflBf_V2_297	494000
1442	ENSG00000099785	MARCH2	Hsa19	8384187	169531	gw.71.205.1	BflBf_V2_297	494000
1445	ENSG00000113368	LMNB1	Hsa5	126140214	121709	estExt_fgenesh2_pg.C_710026	BflBf_V2_297	501066
1445	ENSG00000160789	LMNA	Hsa1	154318993	121709	estExt_fgenesh2_pg.C_710026	BflBf_V2_297	501066
1445	ENSG00000176619	LMNB2	Hsa19	2379165	121709	estExt_fgenesh2_pg.C_710026	BflBf_V2_297	501066
1457	ENSG00000105298	CS029_HUMAN	Hsa19	3561650	121714	estExt_fgenesh2_pg.C_710032	Bflscaffold_71	562372
1458	ENSG00000131788	PIAS3	Hsa1	144287346	194147	gw.71.240.1	Bflscaffold_71	959280
1458	ENSG00000078043	PIAS2	Hsa18	42646058	194147	gw.71.240.1	Bflscaffold_71	959280
1458	ENSG00000105229	PIAS4	Hsa19	3958748	194147	gw.71.240.1	Bflscaffold_71	959280
1458	ENSG00000033800	PIAS1	Hsa15	66165695	194147	gw.71.240.1	Bflscaffold_71	959280
1470	ENSG00000167468	GPX4	Hsa19	1054936	131657	estExt_fgenesh2_pg.C_5670025	Bflscaffold_567	355646
1501	ENSG00000082996	RNF13	Hsa3	151013194	145765	gw.392.25.1	Bflscaffold_392	650462
1501	ENSG00000108523	RNF167	Hsa17	4784048	145765	gw.392.25.1	Bflscaffold_392	650462
1501	ENSG00000105428	ZNRF4	Hsa19	5406442	145765	gw.392.25.1	Bflscaffold_392	650462
1514	ENSG00000065717	TLE2	Hsa19	2948637	102222	fgenesh2_pg.scaffold_403000023	BflBf_V2_282	356197
1514	ENSG00000106829	TLE4	Hsa9	81376508	102222	fgenesh2_pg.scaffold_403000023	BflBf_V2_282	356197
1514	ENSG00000140332	TLE3	Hsa15	68129473	102222	fgenesh2_pg.scaffold_403000023	BflBf_V2_282	356197
1514	ENSG00000196781	TLE1	Hsa9	83388418	102222	fgenesh2_pg.scaffold_403000023	BflBf_V2_282	356197
1583	ENSG00000141977	CIB3	Hsa19	16133179	83937	fgenesh2_pg.scaffold_126000006	Bflscaffold_126	48572
1583	ENSG00000136425	CIB2	Hsa15	76184046	83937	fgenesh2_pg.scaffold_126000006	Bflscaffold_126	48572
1584	ENSG00000125912	NCLN	Hsa19	3136875	123703	estExt_fgenesh2_pg.C_1260063	Bflscaffold_126	1018004

1597	ENSG00000167460	TPM4	Hsa19	16039348	90043	fgenesh2_pg.scaffold_193000048	BflBf_V2_223	286489
1597	ENSG00000187536		Hsa16	51246717	90043	fgenesh2_pg.scaffold_193000048	BflBf_V2_223	286489
1597	ENSG00000177152		Hsa7	116399455	90043	fgenesh2_pg.scaffold_193000048	BflBf_V2_223	286489
1598	ENSG00000148408	CACNA1B	Hsa9	139892062	90019	fgenesh2_pg.scaffold_193000024	Bflscaffold_193	730715
1598	ENSG00000141837	CACNA1A	Hsa19	13179088	90019	fgenesh2_pg.scaffold_193000024	Bflscaffold_193	730715
1598	ENSG00000198216	CACNA1E	Hsa1	179648918	90019	fgenesh2_pg.scaffold_193000024	Bflscaffold_193	730715
1598	ENSG00000102001	CACNA1F	HsaX	48948467	90019	fgenesh2_pg.scaffold_193000024	Bflscaffold_193	730715
1602	ENSG00000183617	MRPL54	Hsa19	3713672	125728	estExt_fgenesh2_pg.C_1930051	BflBf_V2_223	252625
1612	ENSG00000141934	PPAP2C	Hsa19	232048	236130	e_gw.332.59.1	Bflscaffold_332	862261
1619	ENSG00000105607	GCDH	Hsa19	12862974	289893	estExt_gwp.C_8960001	Bflscaffold_896	731
1626	ENSG00000117500	TMED5	Hsa1	93387887	125388	estExt_fgenesh2_pg.C_1810009	BflBf_V2_264	268427
1626	ENSG00000099203	TMED1	Hsa19	10804115	125388	estExt_fgenesh2_pg.C_1810009	BflBf_V2_264	268427
1764	ENSG00000167670	CHAF1A	Hsa19	4353661	94449	fgenesh2_pg.scaffold_253000033	BflBf_V2_119	1542345
1768	ENSG00000011132	APBA3	Hsa19	3701771	266870	estExt_GenewiseH_1.C_2530045	BflBf_V2_119	1410621
1829	ENSG00000154582	TCEB1	Hsa8	75019928	131718	estExt_fgenesh2_pg.C_5760018	Bflscaffold_576	309778
1829	ENSG00000123257	649647	Hsa10	10256199	131718	estExt_fgenesh2_pg.C_5760018	Bflscaffold_576	309778
1829	ENSG00000105694	O75863_HUMAN	Hsa19	2489108	131718	estExt_fgenesh2_pg.C_5760018	Bflscaffold_576	309778
1901	ENSG00000087258	GNAO1	Hsa16	54783389	251150	e_gw.577.15.1	Bflscaffold_577	228590
1901	ENSG00000156049	GNA14	Hsa9	79227815	251150	e_gw.577.15.1	Bflscaffold_577	228590
1901	ENSG00000060558	GNA15	Hsa19	3087230	251150	e_gw.577.15.1	Bflscaffold_577	228590
1940	ENSG00000141994	DUS3L	Hsa19	5736155	90627	fgenesh2_pg.scaffold_200000010	Bflscaffold_200	205628
2016	ENSG00000188039	NP_001007526	Hsa19	16703001	124973	estExt_fgenesh2_pg.C_1650057	BflBf_V2_186	2378674
2071	ENSG00000169231	THBS3	Hsa1	153432003	273182	estExt_GenewiseH_1.C_6290011	Bflscaffold_629	80966
2071	ENSG00000113296	THBS4	Hsa5	79366859	273182	estExt_GenewiseH_1.C_6290011	Bflscaffold_629	80966
2071	ENSG00000105664	COMP	Hsa19	18754584	273182	estExt_GenewiseH_1.C_6290011	Bflscaffold_629	80966
2215	ENSG00000185630	PBX1	Hsa1	162795496	275000	estExt_gwp.C_30002	Bflscaffold_3	30202
2215	ENSG00000167081	PBX3	Hsa9	127548372	275000	estExt_gwp.C_30002	Bflscaffold_3	30202
2215	ENSG00000204304	PBX2	Hsa6	32260496	275000	estExt_gwp.C_30002	Bflscaffold_3	30202
2215	ENSG00000206247	PBX2_HUMAN	Hsac6_COX	32248249	275000	estExt_gwp.C_30002	Bflscaffold_3	30202
2215	ENSG00000206315	PBX2_HUMAN	Hsac6_QBL	32252994	275000	estExt_gwp.C_30002	Bflscaffold_3	30202
2215	ENSG00000181814	Q6NW39_HUMAN	Hsa3	144377817	275000	estExt_gwp.C_30002	Bflscaffold_3	30202
2215	ENSG00000105717	PBX4	Hsa19	19533524	275000	estExt_gwp.C_30002	Bflscaffold_3	30202
2246	ENSG00000130309	GLT25D1	Hsa19	17527511	199663	e_gw.3.147.1	Bflscaffold_3	1099099
2246	ENSG00000198756	GLT25D2	Hsa1	182171594	199663	e_gw.3.147.1	Bflscaffold_3	1099099
2246	ENSG00000167123	CEECAM1	Hsa9	130213866	199663	e_gw.3.147.1	Bflscaffold_3	1099099
2274	ENSG00000105248	CCDC94	Hsa19	4198087	64047	fgenesh2_pg.scaffold_3000077	BflBf_V2_41	4342218
2293	ENSG00000150471	LPHN3	Hsa4	62045434	124770	estExt_fgenesh2_pg.C_1560062	Bflscaffold_156	685225
2293	ENSG00000117114	LPHN2	Hsa1	81544439	124770	estExt_fgenesh2_pg.C_1560062	Bflscaffold_156	685225
2293	ENSG00000072071	LPHN1	Hsa19	14119547	124770	estExt_fgenesh2_pg.C_1560062	Bflscaffold_156	685225
2316	ENSG00000147852	VLDLR	Hsa9	2525657	227282	e_gw.244.94.1	BflBf_V2_113	317048
2316	ENSG00000130164	LDLR	Hsa19	11061155	227282	e_gw.244.94.1	BflBf_V2_113	317048
2316	ENSG00000157193	LRP8	Hsa1	53483800	227282	e_gw.244.94.1	BflBf_V2_113	317048
2362	ENSG00000176533	GNG7	Hsa19	2462218	251807	e_gw.595.39.1	Bflscaffold_595	232299
2362	ENSG00000172380	GNG12	Hsa1	67939737	251807	e_gw.595.39.1	Bflscaffold_595	232299
2362	ENSG00000186469	GNG2	Hsa14	51383702	251807	e_gw.595.39.1	Bflscaffold_595	232299
2362	ENSG00000168243	GNG4	Hsa1	233780755	251807	e_gw.595.39.1	Bflscaffold_595	232299
2362	ENSG00000167414	GNG8	Hsa19	51829173	251807	e_gw.595.39.1	Bflscaffold_595	232299
2362	ENSG00000162188	GNG3	Hsa11	62231706	251807	e_gw.595.39.1	Bflscaffold_595	232299
2362	ENSG00000182625		Hsa15	56765902	251807	e_gw.595.39.1	Bflscaffold_595	232299
2362	ENSG00000127920	GNG11	Hsa7	93388974	251807	e_gw.595.39.1	Bflscaffold_595	232299
2362	ENSG00000167083	GNGT2	Hsa17	44638596	251807	e_gw.595.39.1	Bflscaffold_595	232299
2362	ENSG00000127928	GNGT1	Hsa7	93373756	251807	e_gw.595.39.1	Bflscaffold_595	232299
2425	ENSG00000105641	SLC5A5	Hsa19	17843782	213839	e_gw.55.23.1	BflBf_V2_246	823255
2434	ENSG00000105072	C19orf44	Hsa19	16468224	126338	estExt_fgenesh2_pg.C_2120007	BflBf_V2_61	531284
2435	ENSG00000142453	CARM1	Hsa19	10843253	126366	estExt_fgenesh2_pg.C_2120060	BflBf_V2_61	1251783
2438	ENSG00000090674	MCOLN1	Hsa19	7493514	91623	fgenesh2_pg.scaffold_212000002	BflBf_V2_61	480361
2438	ENSG00000153898	MCOLN2	Hsa1	85163855	91623	fgenesh2_pg.scaffold_212000002	BflBf_V2_61	480361
2438	ENSG00000055732	MCOLN3	Hsa1	85256353	91623	fgenesh2_pg.scaffold_212000002	BflBf_V2_61	480361
2440	ENSG00000127452	FBXL12	Hsa19	9781948	126380	estExt_fgenesh2_pg.C_2120092	BflBf_V2_61	1778238
2441	ENSG00000050628	PTGER3	Hsa1	71090624	126346	estExt_fgenesh2_pg.C_2120024	BflBf_V2_61	705854
2441	ENSG00000160013	PTGIR	Hsa19	51815566	126346	estExt_fgenesh2_pg.C_2120024	BflBf_V2_61	705854
2441	ENSG00000168229	PTGDR	Hsa14	51804181	126346	estExt_fgenesh2_pg.C_2120024	BflBf_V2_61	705854
2441	ENSG00000006638	TBXA2R	Hsa19	3545778	126346	estExt_fgenesh2_pg.C_2120024	BflBf_V2_61	705854
2441	ENSG00000122420	PTGFR	Hsa1	78542156	126346	estExt_fgenesh2_pg.C_2120024	BflBf_V2_61	705854
2441	ENSG00000160951	PTGER1	Hsa19	14444279	126346	estExt_fgenesh2_pg.C_2120024	BflBf_V2_61	705854
2441	ENSG00000050628	PTGER3	Hsa1	71090624	126346	estExt_fgenesh2_pg.C_2120024	BflBf_V2_61	705854
2441	ENSG00000160013	PTGIR	Hsa19	51815566	126346	estExt_fgenesh2_pg.C_2120024	BflBf_V2_61	705854
2441	ENSG00000168229	PTGDR	Hsa14	51804181	126346	estExt_fgenesh2_pg.C_2120024	BflBf_V2_61	705854
2441	ENSG00000006638	TBXA2R	Hsa19	3545778	126346	estExt_fgenesh2_pg.C_2120024	BflBf_V2_61	705854
2441	ENSG00000122420	PTGFR	Hsa1	78542156	126346	estExt_fgenesh2_pg.C_2120024	BflBf_V2_61	705854
2441	ENSG00000160951	PTGER1	Hsa19	14444279	126346	estExt_fgenesh2_pg.C_2120024	BflBf_V2_61	705854
2443	ENSG00000130175	PRKCSH	Hsa19	11407269	126344	estExt_fgenesh2_pg.C_2120014	BflBf_V2_61	627684
2444	ENSG00000145675	PIK3R1	Hsa5	67547360	91712	fgenesh2_pg.scaffold_212000091	BflBf_V2_61	1726745
2444	ENSG00000105647	PIK3R2	Hsa19	18125016	91712	fgenesh2_pg.scaffold_212000091	BflBf_V2_61	1726745
2445	ENSG00000086015	MAST2	Hsa1	46041872	126376	estExt_fgenesh2_pg.C_2120083	BflBf_V2_61	1620481
2445	ENSG00000105613	MAST1	Hsa19	12810348	126376	estExt_fgenesh2_pg.C_2120083	BflBf_V2_61	1620481
2450	ENSG00000179218	CALR	Hsa19	12910392	115348	estExt_fgenesh2_pm.C_2120001	BflBf_V2_61	538731
2450	ENSG00000141979	CALR3	Hsa19	16450888	115348	estExt_fgenesh2_pm.C_2120001	BflBf_V2_61	538731
2450	ENSG00000179218	CALR	Hsa19	12910392	115348	estExt_fgenesh2_pm.C_2120001	BflBf_V2_61	538731
2450	ENSG00000141979	CALR3	Hsa19	16450888	115348	estExt_fgenesh2_pm.C_2120001	BflBf_V2_61	538731
2452	ENSG00000123159	GIPC1	Hsa19	14449572	91652	fgenesh2_pg.scaffold_212000031	BflBf_V2_61	813202
2452	ENSG00000179855	GIPC3	Hsa19	3536569	91652	fgenesh2_pg.scaffold_212000031	BflBf_V2_61	813202
2452	ENSG00000137960	GIPC2	Hsa1	78284174	91652	fgenesh2_pg.scaffold_212000031	BflBf_V2_61	813202
2452	ENSG00000123159	GIPC1	Hsa19	14449572	91652	fgenesh2_pg.scaffold_212000031	BflBf_V2_61	813202
2452	ENSG00000179855	GIPC3	Hsa19	3536569	91652	fgenesh2_pg.scaffold_212000031	BflBf_V2_61	813202
2452	ENSG00000137960	GIPC2	Hsa1	78284174	91652	fgenesh2_pg.scaffold_212000031	BflBf_V2_61	813202
2464	ENSG00000185442	NP_997329.1	Hsa15	90961685	122141	estExt_fgenesh2_pg.C_780166	BflBf_V2_32	5026527
2464	ENSG00000118050	C19orf24	Hsa19	1226520	122141	estExt_fgenesh2_pg.C_780166	BflBf_V2_32	5026527
2465	ENSG00000177076	ASAH3L	Hsa9	19398925	280354	estExt_gwp.C_780194	BflBf_V2_32	4637900

2465	ENSG00000167769	ASAH3	Hsa19	6257725	280354	estExt_gwp.C_780194	BflBf_V2_32	4637900
2465	ENSG00000180105		Hsa2	206983053	280354	estExt_gwp.C_780194	BflBf_V2_32	4637900
2468	ENSG00000127220	ABHD8	Hsa19	17263941	219317	e_gw.78.234.1	BflBf_V2_32	4552229
2479	ENSG00000173976	RAXL1	Hsa19	3448813	219419	e_gw.78.228.1	BflBf_V2_32	4049754
2488	ENSG00000184162	NP_795361.1	Hsa19	19173734	219478	e_gw.78.76.1	BflBf_V2_32	4986934
2491	ENSG00000157483	MYO1E	Hsa15	57215461	219333	e_gw.78.36.1	BflBf_V2_32	3765987
2491	ENSG00000142347	MYO1F	Hsa19	8491689	219333	e_gw.78.36.1	BflBf_V2_32	3765987
2495	ENSG00000074842	CS010_HUMAN	Hsa19	4608566	122127	estExt_fgenesh2_pg.C_780139	Bflscaffold_78	1940458
2502	ENSG00000164331	ANKRA2	Hsa5	72883919	280360	estExt_gwp.C_780217	Bflscaffold_78	1974091
2502	ENSG00000064490	RFXANK	Hsa19	19164008	280360	estExt_gwp.C_780217	Bflscaffold_78	1974091
2538	ENSG00000056097	ZFR	Hsa5	32390214	133155	estExt_fgenesh2_pg.C_9550005	BflBf_V2_409	37049
2538	ENSG00000105278	KIAA1086	Hsa19	3755022	133155	estExt_fgenesh2_pg.C_9550005	BflBf_V2_409	37049
2540	ENSG00000145780	FEM1C	Hsa5	114884507	274612	estExt_GenewiseH_1.C_9550005	BflBf_V2_409	61715
2540	ENSG00000141965	FEM1A	Hsa19	4742770	274612	estExt_GenewiseH_1.C_9550005	BflBf_V2_409	61715
2540	ENSG00000169018	FEM1B	Hsa15	66357810	274612	estExt_GenewiseH_1.C_9550005	BflBf_V2_409	61715
2604	ENSG00000131351	NP_219485.1	Hsa19	17021573	69118	fgenesh2_pg.scaffold_22000056	BflBf_V2_174	565091
2730	ENSG00000125735	TNFSF14	Hsa19	6615568	100450	fgenesh2_pg.scaffold_360000041	Bflscaffold_360	555950
2758	ENSG00000130255	RPL36	Hsa19	5641272	132286	estExt_fgenesh2_pg.C_6700004	Bflscaffold_670	99558
2758	ENSG00000186502	347292	Hsa9	110428934	132286	estExt_fgenesh2_pg.C_6700004	Bflscaffold_670	99558
2758	ENSG00000130225	Q8WX03_HUMAN	HsaX	114339251	132286	estExt_fgenesh2_pg.C_6700004	Bflscaffold_670	99558
2758	ENSG00000169253	643205	Hsa4	77176927	132286	estExt_fgenesh2_pg.C_6700004	Bflscaffold_670	99558
2760	ENSG00000117569	PTBP2	Hsa1	96959927	237696	e_gw.348.59.1	BflBf_V2_278	510700
2760	ENSG00000011304	PTBP1	Hsa19	748411	237696	e_gw.348.59.1	BflBf_V2_278	510700
2760	ENSG00000119314	ROD1	Hsa9	114020536	237696	e_gw.348.59.1	BflBf_V2_278	510700
2770	ENSG00000129353	SLC44A2	Hsa19	10574186	164301	gw.333.77.1	Bflscaffold_333	214276
2770	ENSG00000206269	CTL4_HUMAN	Hsac6_COX	31965993	164301	gw.333.77.1	Bflscaffold_333	214276
2770	ENSG00000206378	CTL4_HUMAN	Hsac6_QBL	31964443	164301	gw.333.77.1	Bflscaffold_333	214276
2770	ENSG00000204385	SLC44A4	Hsa6	31938946	164301	gw.333.77.1	Bflscaffold_333	214276
2770	ENSG00000137968	SLC44A5	Hsa1	75440404	164301	gw.333.77.1	Bflscaffold_333	214276
2776	ENSG00000160113	NR2F6	Hsa19	17203694	236186	e_gw.333.48.1	BflBf_V2_34	1973527
2856	ENSG00000175426	PCSK1	Hsa5	95751875	105744	fgenesh2_pg.scaffold_505000017	BflBf_V2_296	229274
2856	ENSG00000140564	FURIN	Hsa15	89212889	105744	fgenesh2_pg.scaffold_505000017	BflBf_V2_296	229274
2856	ENSG00000115257	PCSK4	Hsa19	1432427	105744	fgenesh2_pg.scaffold_505000017	BflBf_V2_296	229274
2862	ENSG00000105669	COPE	Hsa19	18871323	287380	estExt_gwp.C_5050035	BflBf_V2_296	276318
2919	ENSG00000105364	MRPL4	Hsa19	10223640	124913	estExt_fgenesh2_pg.C_1620010	BflBf_V2_247	627080
2941	ENSG00000008382	NP_116257.2	Hsa19	4294551	221394	e_gw.88.5.1	BflBf_V2_152	485895
2960	ENSG00000123144	C19orf43	Hsa19	12702487	102279	fgenesh2_pg.scaffold_404000038	Bflscaffold_404	540067
2964	ENSG00000161847	RAVER1	Hsa19	10287891	241985	e_gw.404.38.1	BflBf_V2_99	490311
3021	ENSG00000105726	ATP13A1	Hsa19	19617009	124469	estExt_fgenesh2_pg.C_1480029	Bflscaffold_148	568185
3026	ENSG00000088247	KHSRP	Hsa19	6364457	124476	estExt_fgenesh2_pg.C_1480047	BflBf_V2_192	884855
3030	ENSG00000135823	STX6	Hsa1	179179798	116588	estExt_fgenesh2_pm.C_6570002	Bflscaffold_657	139014
3030	ENSG00000104915	STX10	Hsa19	13115903	116588	estExt_fgenesh2_pm.C_6570002	Bflscaffold_657	139014
3097	ENSG00000130332	LSM7	Hsa19	2272522	118403	estExt_fgenesh2_pg.C_130080	BflBf_V2_197	1919966
3100	ENSG00000106701	FSD1L	Hsa9	107250147	67111	fgenesh2_pg.scaffold_13000023	BflBf_V2_197	351889
3100	ENSG00000105255	FSD1	Hsa19	4255668	67111	fgenesh2_pg.scaffold_13000023	BflBf_V2_197	351889
3102	ENSG00000164180	TMEM161B	Hsa5	87526781	67147	fgenesh2_pg.scaffold_13000059	BflBf_V2_197	1313438
3102	ENSG00000064545	TMEM161A	Hsa19	19091430	67147	fgenesh2_pg.scaffold_13000059	BflBf_V2_197	1313438
3173	ENSG00000105088	OLFM2	Hsa19	9825438	226575	e_gw.238.47.1	BflBf_V2_34	3207763
3173	ENSG00000118733	OLFM3	Hsa1	102040718	226575	e_gw.238.47.1	BflBf_V2_34	3207763
3173	ENSG00000183801	OLFML1	Hsa11	7463289	226575	e_gw.238.47.1	BflBf_V2_34	3207763
3202	ENSG00000186204	CYP4F12	Hsa19	15645340	211211	e_gw.44.1.1	Bflscaffold_44	154158
3202	ENSG00000171903	CYP4F11	Hsa19	15884181	211211	e_gw.44.1.1	Bflscaffold_44	154158
3202	ENSG00000186529	CYP4F3	Hsa19	15613196	211211	e_gw.44.1.1	Bflscaffold_44	154158
3202	ENSG00000171954	NP_775754.1	Hsa19	15497144	211211	e_gw.44.1.1	Bflscaffold_44	154158
3231	ENSG00000175898	EDG5	Hsa19	10195520	109885	fgenesh2_pg.scaffold_715000019	Bflscaffold_715	194777
3235	ENSG00000032444	PNPLA6	Hsa19	7505075	81516	fgenesh2_pg.scaffold_103000011	BflBf_V2_240	101133
3235	ENSG00000130653	PNPLA7	Hsa9	139474225	81516	fgenesh2_pg.scaffold_103000011	BflBf_V2_240	101133
3295	ENSG00000174886	NDUFA11	Hsa19	5842287	129508	estExt_fgenesh2_pg.C_3710021	BflBf_V2_69	1070854
3308	ENSG00000011009	LYPLA2	Hsa1	23990047	137204	gw.541.4.1	Bflscaffold_541	149994
3308	ENSG00000120992	LYPLA1	Hsa8	55121492	137204	gw.541.4.1	Bflscaffold_541	149994
3308	ENSG00000206204	NR_001444.2	Hsac6_COX	33402009	137204	gw.541.4.1	Bflscaffold_541	149994
3308	ENSG00000204198	NR_001444.2	Hsa6	33441303	137204	gw.541.4.1	Bflscaffold_541	149994
3308	ENSG00000206276		Hsac6_QBL	33405022	137204	gw.541.4.1	Bflscaffold_541	149994
3308	ENSG00000205677	388499	Hsa19	7850330	137204	gw.541.4.1	Bflscaffold_541	149994
3364	ENSG00000147872	ADFP	Hsa9	19105760	237458	e_gw.346.53.1	Bflscaffold_346	360484
3364	ENSG00000105355	M6PRBP1	Hsa19	4789346	237458	e_gw.346.53.1	Bflscaffold_346	360484
3365	ENSG00000115268	RPS15	Hsa19	1389363	99641	fgenesh2_pg.scaffold_346000008	BflBf_V2_180	145557
3365	ENSG00000138396	401019	Hsa2	172082026	99641	fgenesh2_pg.scaffold_346000008	BflBf_V2_180	145557
3365	ENSG00000162872	440733	Hsa1	232559073	99641	fgenesh2_pg.scaffold_346000008	BflBf_V2_180	145557
3368	ENSG00000115286	NDUFS7	Hsa19	1334883	269169	estExt_GenewiseH_1.C_3460062	Bflscaffold_346	590407
3373	ENSG00000173365	OR6M1	Hsa11	123181326	89840	fgenesh2_pg.scaffold_191000018	Bflscaffold_191	249673
3373	ENSG00000176231	OR10H4	Hsa19	15920818	89840	fgenesh2_pg.scaffold_191000018	Bflscaffold_191	249673
3373	ENSG00000171936	OR10H3	Hsa19	15713203	89840	fgenesh2_pg.scaffold_191000018	Bflscaffold_191	249673
3401	ENSG00000123131	PRDX4	HsaX	23592300	288924	estExt_gwp.C_6810012	BflBf_V2_321	233951
3401	ENSG00000167815	PRDX2	Hsa19	12768635	288924	estExt_gwp.C_6810012	BflBf_V2_321	233951
3433	ENSG00000188229	TUBB2C	Hsa9	139255532	96664	fgenesh2_pg.scaffold_288000035	Bflscaffold_288	715205
3433	ENSG00000137285	TUBB2B	Hsa6	3169516	96664	fgenesh2_pg.scaffold_288000035	Bflscaffold_288	715205
3433	ENSG00000196230	TUBB	Hsa6	30796101	96664	fgenesh2_pg.scaffold_288000035	Bflscaffold_288	715205
3433	ENSG00000183311	TBB2_HUMAN	Hsac6_QBL	30820521	96664	fgenesh2_pg.scaffold_288000035	Bflscaffold_288	715205
3433	ENSG00000137379	TBB2_HUMAN	Hsac6_COX	30825252	96664	fgenesh2_pg.scaffold_288000035	Bflscaffold_288	715205
3433	ENSG00000104833	TUBB4	Hsa19	6445331	96664	fgenesh2_pg.scaffold_288000035	Bflscaffold_288	715205
3433	ENSG00000176014	TUBB6	Hsa18	12298215	96664	fgenesh2_pg.scaffold_288000035	Bflscaffold_288	715205
3433	ENSG00000173876	NP_817124.1	Hsa10	82832	96664	fgenesh2_pg.scaffold_288000035	Bflscaffold_288	715205
3433	ENSG00000173213		Hsa18	37358	96664	fgenesh2_pg.scaffold_288000035	Bflscaffold_288	715205
3433	ENSG00000159247	643224	Hsa9	140189314	96664	fgenesh2_pg.scaffold_288000035	Bflscaffold_288	715205
3433	ENSG00000127589	TUBB4Q	Hsa4	191140672	96664	fgenesh2_pg.scaffold_288000035	Bflscaffold_288	715205
3435	ENSG00000110436	SLC1A2	Hsa11	35229329	283182	estExt_gwp.C_2880022	BflBf_V2_33	1005760
3435	ENSG00000105143	SLC1A6	Hsa19	14921991	283182	estExt_gwp.C_2880022	BflBf_V2_33	1005760
3443	ENSG00000105705	SF4	Hsa19	19247827	104023	fgenesh2_pg.scaffold_450000023	Bflscaffold_450	339494

3480	ENSG00000105549	THEG	Hsa19	313059	120588	estExt_fgenesh2_pg.C_470079	BflBf_V2_38	1489935
3489	ENSG00000105011	ASF1B	Hsa19	14091322	129202	estExt_fgenesh2_pg.C_3500053	Bflscaffold_350	778445
3489	ENSG00000111875	ASF1A	Hsa6	119256901	129202	estExt_fgenesh2_pg.C_3500053	Bflscaffold_350	778445
3514	ENSG00000113448	PDE4D	Hsa5	58305622	134158	gw.798.1.1	Bflscaffold_798	7151
3514	ENSG00000184588	PDE4B	Hsa1	66030785	134158	gw.798.1.1	Bflscaffold_798	7151
3514	ENSG00000105650	PDE4C	Hsa19	18182010	134158	gw.798.1.1	Bflscaffold_798	7151
3555	ENSG00000142002	DPP9	Hsa19	4626237	102713	fgenesh2_pg.scaffold_416000004	Bflscaffold_416	129235
3592	ENSG00000072954	TMEM38A	Hsa19	16632938	280800	estExt_gwp.C_900011	Bflscaffold_90	80268
3665	ENSG00000182004	SNRPE	Hsa1	202097366	268096	estExt_GenewiseH_1.C_3020024	BflBf_V2_243	445446
3665	ENSG00000182367	SNRPEL1	Hsa9	6738546	268096	estExt_GenewiseH_1.C_3020024	BflBf_V2_243	445446
3665	ENSG00000182225	651086	Hsa19	5527671	268096	estExt_GenewiseH_1.C_3020024	BflBf_V2_243	445446
3670	ENSG00000129355	CDKN2D	Hsa19	10538139	112465	fgenesh2_pg.scaffold_1304000001	Bflscaffold_1304	5728
3670	ENSG00000123080	CDKN2C	Hsa1	51199005	112465	fgenesh2_pg.scaffold_1304000001	Bflscaffold_1304	5728
3670	ENSG00000147889	CDKN2A	Hsa9	21957751	112465	fgenesh2_pg.scaffold_1304000001	Bflscaffold_1304	5728
3670	ENSG00000147883	CDKN2B	Hsa9	21992902	112465	fgenesh2_pg.scaffold_1304000001	Bflscaffold_1304	5728
3701	ENSG00000182490	TSSK2	Hsa22	17497793	58823	fgenesh2_pm.scaffold_89000002	BflBf_V2_147	3795667
3701	ENSG00000162526	TSSK3	Hsa1	32600385	58823	fgenesh2_pm.scaffold_89000002	BflBf_V2_147	3795667
3701	ENSG00000178093	TSSK6	Hsa19	19484230	58823	fgenesh2_pm.scaffold_89000002	BflBf_V2_147	3795667
3714	ENSG00000105519	CAPS	Hsa19	5862386	284500	estExt_gwp.C_3420021	Bflscaffold_342	193636
3719	ENSG00000156052	GNAQ	Hsa9	79520823	269618	estExt_GenewiseH_1.C_3630023	Bflscaffold_363	576110
3719	ENSG00000088256	GNA11	Hsa19	3045408	269618	estExt_GenewiseH_1.C_3630023	Bflscaffold_363	576110
3726	ENSG00000161888	SPBC24	Hsa19	11118859	119496	estExt_fgenesh2_pg.C_290143	BflBf_V2_94	3220925
3806	ENSG00000139219	COL2A1	Hsa12	46653018	74779	fgenesh2_pg.scaffold_53000100	BflBf_V2_9	2461621
3806	ENSG00000108821	COL1A1	Hsa17	45616456	74779	fgenesh2_pg.scaffold_53000100	BflBf_V2_9	2461621
3806	ENSG00000204262	COL5A2	Hsa2	189605486	74779	fgenesh2_pg.scaffold_53000100	BflBf_V2_9	2461621
3806	ENSG00000164692	COL1A2	Hsa7	93861809	74779	fgenesh2_pg.scaffold_53000100	BflBf_V2_9	2461621
3806	ENSG00000168542	COL3A1	Hsa2	189547344	74779	fgenesh2_pg.scaffold_53000100	BflBf_V2_9	2461621
3806	ENSG00000130635	COL5A1	Hsa9	136673473	74779	fgenesh2_pg.scaffold_53000100	BflBf_V2_9	2461621
3806	ENSG00000060718	COL11A1	Hsa1	103114611	74779	fgenesh2_pg.scaffold_53000100	BflBf_V2_9	2461621
3806	ENSG00000204248	COL11A2	Hsa6	33238447	74779	fgenesh2_pg.scaffold_53000100	BflBf_V2_9	2461621
3806	ENSG00000112112	Q6LCP7_HUMAN	Hsac6_COX	33199176	74779	fgenesh2_pg.scaffold_53000100	BflBf_V2_9	2461621
3806	ENSG00000080573	COL5A3	Hsa19	9931237	74779	fgenesh2_pg.scaffold_53000100	BflBf_V2_9	2461621
3806	ENSG00000187498	COL4A1	Hsa13	109599311	74779	fgenesh2_pg.scaffold_53000100	BflBf_V2_9	2461621
3806	ENSG00000188153	COL4A5	HsaX	107569810	74779	fgenesh2_pg.scaffold_53000100	BflBf_V2_9	2461621
3806	ENSG00000169436	COL22A1	Hsa8	139669661	74779	fgenesh2_pg.scaffold_53000100	BflBf_V2_9	2461621
3806	ENSG00000081052	COL4A4	Hsa2	227578177	74779	fgenesh2_pg.scaffold_53000100	BflBf_V2_9	2461621
3809	ENSG00000105518	NP_940938.1	Hsa19	11314457	213322	e_gw.53.321.1	BflBf_V2_9	1202243
3890	ENSG00000127527	EPS15L1	Hsa19	16333408	194060	gw.287.136.1	BflBf_V2_39	4771285
3967	ENSG00000079999	KEAP1	Hsa19	10457802	99607	fgenesh2_pg.scaffold_345000037	BflBf_V2_135	419993
3968	ENSG00000165023	DIRAS2	Hsa9	92411934	237370	e_gw.345.34.1	BflBf_V2_135	549882
3968	ENSG00000176490	DIRAS1	Hsa19	2665566	237370	e_gw.345.34.1	BflBf_V2_135	549882
3968	ENSG00000162595	DIRAS3	Hsa1	68284233	237370	e_gw.345.34.1	BflBf_V2_135	549882
3972	ENSG00000105085	CRSP7	Hsa19	16546718	129066	estExt_fgenesh2_pg.C_3450034	BflBf_V2_135	384950
3997	ENSG00000095059	DHPS	Hsa19	12647565	234951	e_gw.319.25.1	BflBf_V2_163	975807
4024	ENSG00000130159	ECSIT	Hsa19	11477744	259872	estExt_GenewiseH_1.C_40423	Bflscaffold_4	5104483
4115	ENSG00000170989	EDG1	Hsa1	101475032	286966	estExt_gwp.C_4700033	Bflscaffold_470	459997
4115	ENSG00000186354	C9orf47	Hsa9	90795598	286966	estExt_gwp.C_4700033	Bflscaffold_470	459997
4115	ENSG00000198121	EDG2	Hsa9	112675364	286966	estExt_gwp.C_4700033	Bflscaffold_470	459997
4115	ENSG00000171517	EDG7	Hsa1	85049873	286966	estExt_gwp.C_4700033	Bflscaffold_470	459997
4115	ENSG00000064547	EDG4	Hsa19	19595478	286966	estExt_gwp.C_4700033	Bflscaffold_470	459997
4115	ENSG00000181773	GPR3	Hsa1	27591735	286966	estExt_gwp.C_4700033	Bflscaffold_470	459997
4115	ENSG00000188822	CNR2	Hsa1	24069603	286966	estExt_gwp.C_4700033	Bflscaffold_470	459997
4115	ENSG00000125910	EDG6	Hsa19	3129766	286966	estExt_gwp.C_4700033	Bflscaffold_470	459997
4115	ENSG00000132975	GPR12	Hsa13	26230447	286966	estExt_gwp.C_4700033	Bflscaffold_470	459997
4115	ENSG00000170989	EDG1	Hsa1	101475032	286966	estExt_gwp.C_4700033	Bflscaffold_470	459997
4115	ENSG00000186354	C9orf47	Hsa9	90795598	286966	estExt_gwp.C_4700033	Bflscaffold_470	459997
4115	ENSG00000198121	EDG2	Hsa9	112675364	286966	estExt_gwp.C_4700033	Bflscaffold_470	459997
4115	ENSG00000171517	EDG7	Hsa1	85049873	286966	estExt_gwp.C_4700033	Bflscaffold_470	459997
4115	ENSG00000064547	EDG4	Hsa19	19595478	286966	estExt_gwp.C_4700033	Bflscaffold_470	459997
4115	ENSG00000181773	GPR3	Hsa1	27591735	286966	estExt_gwp.C_4700033	Bflscaffold_470	459997
4115	ENSG00000188822	CNR2	Hsa1	24069603	286966	estExt_gwp.C_4700033	Bflscaffold_470	459997
4115	ENSG00000125910	EDG6	Hsa19	3129766	286966	estExt_gwp.C_4700033	Bflscaffold_470	459997
4115	ENSG00000132975	GPR12	Hsa13	26230447	286966	estExt_gwp.C_4700033	Bflscaffold_470	459997
4119	ENSG00000188181	126037	Hsa19	14042210	229529	e_gw.264.89.1	Bflscaffold_264	150111
4144	ENSG00000132024	CC2D1A	Hsa19	13878014	251291	e_gw.579.19.1	Bflscaffold_579	318712
4144	ENSG00000154222	CC2D1B	Hsa1	52588855	251291	e_gw.579.19.1	Bflscaffold_579	318712
4235	ENSG00000077463	SIRT6	Hsa19	4125106	125988	estExt_fgenesh2_pg.C_2010030	BflBf_V2_32	5777123
4237	ENSG00000176624	RKHD2	Hsa18	46954920	126022	estExt_fgenesh2_pg.C_2010087	BflBf_V2_32	5164970
4237	ENSG00000183496	RKHD3	Hsa15	80121183	126022	estExt_fgenesh2_pg.C_2010087	BflBf_V2_32	5164970
4237	ENSG00000181588	RKHD1	Hsa19	1505672	126022	estExt_fgenesh2_pg.C_2010087	BflBf_V2_32	5164970
4237	ENSG00000203759	92312	Hsa1	154312989	126022	estExt_fgenesh2_pg.C_2010087	BflBf_V2_32	5164970
4241	ENSG00000130288	NDUFA13	Hsa19	19487639	115274	estExt_fgenesh2_pm.C_2010007	BflBf_V2_32	5506011
4254	ENSG00000151292	CSNK1G3	Hsa5	122909227	125993	estExt_fgenesh2_pg.C_2010038	BflBf_V2_32	5671151
4254	ENSG00000169118	CSNK1G1	Hsa15	62244771	125993	estExt_fgenesh2_pg.C_2010038	BflBf_V2_32	5671151
4254	ENSG00000133275	CSNK1G2	Hsa19	1892188	125993	estExt_fgenesh2_pg.C_2010038	BflBf_V2_32	5671151
4255	ENSG00000198723	C19orf45	Hsa19	7468460	125998	estExt_fgenesh2_pg.C_2010046	BflBf_V2_32	5501942
4259	ENSG00000105639	JAK3	Hsa19	17788324	90761	fgenesh2_pg.scaffold_201000066	Bflscaffold_201	889217
4259	ENSG00000096968	JAK2	Hsa9	4975245	90761	fgenesh2_pg.scaffold_201000066	Bflscaffold_201	889217
4259	ENSG00000105397	TYK2	Hsa19	10322205	90761	fgenesh2_pg.scaffold_201000066	Bflscaffold_201	889217
4259	ENSG00000162434	JAK1	Hsa1	65071500	90761	fgenesh2_pg.scaffold_201000066	Bflscaffold_201	889217
4259	ENSG00000105639	JAK3	Hsa19	17788324	90761	fgenesh2_pg.scaffold_201000066	Bflscaffold_201	889217
4259	ENSG00000096968	JAK2	Hsa9	4975245	90761	fgenesh2_pg.scaffold_201000066	Bflscaffold_201	889217
4259	ENSG00000105397	TYK2	Hsa19	10322205	90761	fgenesh2_pg.scaffold_201000066	Bflscaffold_201	889217
4259	ENSG00000162434	JAK1	Hsa1	65071500	90761	fgenesh2_pg.scaffold_201000066	Bflscaffold_201	889217
4260	ENSG00000053501	USE1_HUMAN	Hsa19	17187168	125999	estExt_fgenesh2_pg.C_2010047	BflBf_V2_32	5497771
4305	ENSG00000125703	ATG4C	Hsa1	63022394	286359	estExt_gwp.C_4360047	Bflscaffold_436	519909
4305	ENSG00000130734	ATG4D	Hsa19	10515593	286359	estExt_gwp.C_4360047	Bflscaffold_436	519909
4307	ENSG00000141873	SLC39A3	Hsa19	2683525	244130	e_gw.436.23.1	Bflscaffold_436	649920
4308	ENSG00000105648	IFI30	Hsa19	18145579	270946	estExt_GenewiseH_1.C_4360014	Bflscaffold_436	157714

4340	ENSG00000132000	PODNL1	Hsa19	13904167	127889	estExt_fgenesh2_pg.C_2800043	BflBf_V2_159	659082
4352	ENSG00000142459	EVI5L	Hsa19	7801243	88764	fgenesh2_pg.scaffold_178000014	Bflscaffold_178	530515
4352	ENSG00000067208	EVI5	Hsa1	92746841	88764	fgenesh2_pg.scaffold_178000014	Bflscaffold_178	530515
4358	ENSG00000120162	MOBKL2B	Hsa9	27315207	210581	e_gw.42.268.1	BflBf_V2_6	1804109
4358	ENSG00000172081	MOBKL2A	Hsa19	2022037	210581	e_gw.42.268.1	BflBf_V2_6	1804109
4358	ENSG00000142961	MOBKL2C	Hsa1	46845978	210581	e_gw.42.268.1	BflBf_V2_6	1804109
4417	ENSG00000106976	DNM1	Hsa9	130005479	121263	estExt_fgenesh2_pg.C_620139	BflBf_V2_166	2599558
4417	ENSG00000079805	DNM2	Hsa19	10673106	121263	estExt_fgenesh2_pg.C_620139	BflBf_V2_166	2599558
4417	ENSG00000197959	DNM3	Hsa1	170077261	121263	estExt_fgenesh2_pg.C_620139	BflBf_V2_166	2599558
4458	ENSG00000179115	FARSLA	Hsa19	12894294	203076	e_gw.9.50.1	BflBf_V2_167	3860142
4471	ENSG00000085872	CHERP	Hsa19	16489710	117969	estExt_fgenesh2_pg.C_90008	BflBf_V2_167	5006217
4499	ENSG00000099875	MKNK2	Hsa19	1988481	266139	estExt_GenewiseH_1.C_1060041	Bflscaffold_106	696732
4499	ENSG00000079277	MKNK1	Hsa1	46795677	266139	estExt_GenewiseH_1.C_1060041	Bflscaffold_106	696732
4503	ENSG00000116209	TMEM59	Hsa1	54269935	81894	fgenesh2_pg.scaffold_106000072	BflBf_V2_113	1835121
4503	ENSG00000105696	BSMAP_HUMAN	Hsa19	18584691	81894	fgenesh2_pg.scaffold_106000072	BflBf_V2_113	1835121
4505	ENSG00000168172	HOOK3	Hsa8	42871190	281537	estExt_gwp.C_1060100	BflBf_V2_113	1874414
4505	ENSG00000134709	HOOK1	Hsa1	60053121	281537	estExt_gwp.C_1060100	BflBf_V2_113	1874414
4505	ENSG00000095066	HOOK2	Hsa19	12734817	281537	estExt_gwp.C_1060100	BflBf_V2_113	1874414
4513	ENSG00000179271	GADD45GIP1	Hsa19	12925972	281553	estExt_gwp.C_1060138	BflBf_V2_113	2121290
4514	ENSG00000099797	GPSN2	Hsa19	14501382	123019	estExt_fgenesh2_pg.C_1060085	BflBf_V2_113	2018603
4514	ENSG00000205678	NP_001010874	Hsa4	64828385	123019	estExt_fgenesh2_pg.C_1060085	BflBf_V2_113	2018603
4571	ENSG00000143344	RGL1	Hsa1	181871843	118269	estExt_fgenesh2_pg.C_110221	BflBf_V2_136	2098351
4571	ENSG00000205517	RGL3	Hsa19	11356232	118269	estExt_fgenesh2_pg.C_110221	BflBf_V2_136	2098351
4571	ENSG00000206210	RGL2_HUMAN	Hsac6_COX	33328122	118269	estExt_fgenesh2_pg.C_110221	BflBf_V2_136	2098351
4571	ENSG00000206282	RGL2_HUMAN	Hsac6_QBL	33331137	118269	estExt_fgenesh2_pg.C_110221	BflBf_V2_136	2098351
4571	ENSG00000204218	RGL2	Hsa6	33367409	118269	estExt_fgenesh2_pg.C_110221	BflBf_V2_136	2098351
4573	ENSG00000198003	NP_659482.2	Hsa19	11392273	276428	estExt_gwp.C_110599	BflBf_V2_136	2138054
4597	ENSG00000206445	UAP56_HUMAN	Hsac6_QBL	31631137	118235	estExt_fgenesh2_pg.C_110084	BflBf_V2_136	82007
4597	ENSG00000206334	UAP56_HUMAN	Hsac6_COX	31632712	118235	estExt_fgenesh2_pg.C_110084	BflBf_V2_136	82007
4597	ENSG00000198563	BAT1	Hsa6	31605975	118235	estExt_fgenesh2_pg.C_110084	BflBf_V2_136	82007
4597	ENSG00000123136	DDX39	Hsa19	14380632	118235	estExt_fgenesh2_pg.C_110084	BflBf_V2_136	82007
4606	ENSG00000099624	ATP5D	Hsa19	1192749	261515	estExt_GenewiseH_1.C_280119	Bflscaffold_28	1230756
4608	ENSG00000058799	YIPF1	Hsa1	54089980	70406	fgenesh2_pg.scaffold_28000010	BflBf_V2_22	897213
4608	ENSG00000130733	YIPF2	Hsa19	10894446	70406	fgenesh2_pg.scaffold_28000010	BflBf_V2_22	897213
4610	ENSG00000065000	AP3D1	Hsa19	2051993	276908	estExt_gwp.C_280211	BflBf_V2_99	118369
4611	ENSG00000065989	PDE4A	Hsa19	10388449	261528	estExt_GenewiseH_1.C_280159	BflBf_V2_22	2126217
4611	ENSG00000205268	PDE7A	Hsa8	66793867	261528	estExt_GenewiseH_1.C_280159	BflBf_V2_22	2126217
4611	ENSG00000171408	PDE7B	Hsa6	136214527	261528	estExt_GenewiseH_1.C_280159	BflBf_V2_22	2126217
4612	ENSG00000142444	C19orf52	Hsa19	10900466	119395	estExt_fgenesh2_pg.C_280009	BflBf_V2_22	894917
4621	ENSG00000104957	CCDC130	Hsa19	13703574	119420	estExt_fgenesh2_pg.C_280086	Bflscaffold_28	2406231
4622	ENSG00000127226	Q567V2_HUMAN	Hsa19	18165092	206123	e_gw.28.113.1	BflBf_V2_22	1149698
4622	ENSG00000156968	MPV17L	Hsa16	15397163	206123	e_gw.28.113.1	BflBf_V2_22	1149698
4632	ENSG00000105325	FZR1	Hsa19	3457368	261554	estExt_GenewiseH_1.C_280251	Bflscaffold_28	3040390
4633	ENSG00000167470	MIDN	Hsa19	1199552	70434	fgenesh2_pg.scaffold_28000038	BflBf_V2_22	1600042
4636	ENSG00000186115	CYP4F2	Hsa19	15849834	205999	e_gw.28.111.1	BflBf_V2_22	1573667
4636	ENSG00000154198	Q8N1L4_HUMAN	Hsa1	47081354	205999	e_gw.28.111.1	BflBf_V2_22	1573667
4638	ENSG00000099800	TIMM13	Hsa19	2376630	60179	fgenesh2_pm.scaffold_198000005	BflBf_V2_237	385437
4698	ENSG00000167475	SCAMP4	Hsa19	1856373	289594	estExt_gwp.C_8050034	Bflscaffold_805	143953
4723	ENSG00000181029	TRAPPC5	Hsa19	7651761	234234	e_gw.311.93.1	BflBf_V2_153	536071
4727	ENSG00000169375	SIN3A	Hsa15	73450330	97881	fgenesh2_pg.scaffold_311000026	BflBf_V2_153	315236
4727	ENSG00000127511	SIN3B	Hsa19	16801211	97881	fgenesh2_pg.scaffold_311000026	BflBf_V2_153	315236
4777	ENSG00000171105	INSR	Hsa19	7067049	128184	estExt_fgenesh2_pg.C_2960033	BflBf_V2_380	100742
4777	ENSG00000140443	IGF1R	Hsa15	97010302	128184	estExt_fgenesh2_pg.C_2960033	BflBf_V2_380	100742
4777	ENSG00000027644	INSRR	Hsa1	155076479	128184	estExt_fgenesh2_pg.C_2960033	BflBf_V2_380	100742
4788	ENSG00000099821	POLRMT	Hsa19	568228	287284	estExt_gwp.C_4940035	BflBf_V2_32	7455654
4871	ENSG00000130520	LSM4	Hsa19	18278720	281075	estExt_gwp.C_960049	BflBf_V2_211	389554
4871	ENSG00000111987	LSM2_HUMAN	Hsac6_COX	31899794	281075	estExt_gwp.C_960049	BflBf_V2_211	389554
4871	ENSG00000172850	LSM2_HUMAN	Hsac6_QBL	31898277	281075	estExt_gwp.C_960049	BflBf_V2_211	389554
4871	ENSG00000204392	LSM2	Hsa6	31873152	281075	estExt_gwp.C_960049	BflBf_V2_211	389554
5043	ENSG00000169032	MAP2K1	Hsa15	64466674	124326	estExt_fgenesh2_pg.C_1450051	BflBf_V2_123	635598
5043	ENSG00000126934	MAP2K2	Hsa19	4041331	124326	estExt_fgenesh2_pg.C_1450051	BflBf_V2_123	635598
5046	ENSG00000105701	FKBP8	Hsa19	18503568	85785	fgenesh2_pg.scaffold_145000043	BflBf_V2_123	685094
5056	ENSG00000105662	CRTC1_HUMAN	Hsa19	18655488	85835	fgenesh2_pg.scaffold_145000093	BflBf_V2_123	210729
5063	ENSG00000161091	C19orf28	Hsa19	3495198	85802	fgenesh2_pg.scaffold_145000060	Bflscaffold_145	1077121
5065	ENSG00000074855	TMEM16H	Hsa19	17295034	85779	fgenesh2_pg.scaffold_145000037	BflBf_V2_123	731934
5067	ENSG00000121848	ZNF364	Hsa1	144322393	124329	estExt_fgenesh2_pg.C_1450057	BflBf_V2_123	551276
5067	ENSG00000070423	RNF126	Hsa19	598530	124329	estExt_fgenesh2_pg.C_1450057	BflBf_V2_123	551276
5071	ENSG00000130517	PGPEP1	Hsa19	18312450	124319	estExt_fgenesh2_pg.C_1450040	BflBf_V2_123	701790
5071	ENSG00000183571	145814	Hsa15	97328859	124319	estExt_fgenesh2_pg.C_1450040	BflBf_V2_123	701790
5074	ENSG00000127445	PIN1	Hsa19	9806999	114914	estExt_fgenesh2_pm.C_1450001	BflBf_V2_123	1054987
5143	ENSG00000172148	OR7A2_HUMAN	Hsa19	14835598	88114	fgenesh2_pg.scaffold_169000063	BflBf_V2_205	1393258
5143	ENSG00000188000	OR7D2	Hsa19	9157458	88114	fgenesh2_pg.scaffold_169000063	BflBf_V2_205	1393258
5143	ENSG00000174667	OR7D4	Hsa19	9185575	88114	fgenesh2_pg.scaffold_169000063	BflBf_V2_205	1393258
5143	ENSG00000170923	OR7G2	Hsa19	9073945	88114	fgenesh2_pg.scaffold_169000063	BflBf_V2_205	1393258
5143	ENSG00000185385	OR7A17	Hsa19	14852238	88114	fgenesh2_pg.scaffold_169000063	BflBf_V2_205	1393258
5143	ENSG00000171496	OR1L8	Hsa9	124369648	88114	fgenesh2_pg.scaffold_169000063	BflBf_V2_205	1393258
5143	ENSG00000127529	OR7C2	Hsa19	14913175	88114	fgenesh2_pg.scaffold_169000063	BflBf_V2_205	1393258
5143	ENSG00000172148	OR7A2_HUMAN	Hsa19	14835598	88114	fgenesh2_pg.scaffold_169000063	BflBf_V2_205	1393258
5143	ENSG00000188000	OR7D2	Hsa19	9157458	88114	fgenesh2_pg.scaffold_169000063	BflBf_V2_205	1393258
5143	ENSG00000174667	OR7D4	Hsa19	9185575	88114	fgenesh2_pg.scaffold_169000063	BflBf_V2_205	1393258
5143	ENSG00000170923	OR7G2	Hsa19	9073945	88114	fgenesh2_pg.scaffold_169000063	BflBf_V2_205	1393258
5143	ENSG00000185385	OR7A17	Hsa19	14852238	88114	fgenesh2_pg.scaffold_169000063	BflBf_V2_205	1393258
5143	ENSG00000171496	OR1L8	Hsa9	124369648	88114	fgenesh2_pg.scaffold_169000063	BflBf_V2_205	1393258
5143	ENSG00000127529	OR7C2	Hsa19	14913175	88114	fgenesh2_pg.scaffold_169000063	BflBf_V2_205	1393258
5166	ENSG00000196365	PRSS15	Hsa19	5642845	67659	fgenesh2_pg.scaffold_15000151	BflBf_V2_65	4444538
5173	ENSG00000113369	ARRDC3	Hsa5	90700299	118530	estExt_fgenesh2_pg.C_150153	BflBf_V2_65	4485499
5173	ENSG00000105643	ARRDC2	Hsa19	17972944	118530	estExt_fgenesh2_pg.C_150153	BflBf_V2_65	4485499
5173	ENSG00000140450	ARRDC4	Hsa15	96304947	118530	estExt_fgenesh2_pg.C_150153	BflBf_V2_65	4485499
5173	ENSG00000117289	TXNIP	Hsa1	144149826	118530	estExt_fgenesh2_pg.C_150153	BflBf_V2_65	4485499

5181	ENSG00000172009	THOP1	Hsa19	2736506	123354	estExt_fgenesh2_pg.C_1180037	BflBf_V2_19	963901
5181	ENSG00000123213	NLN	Hsa5	65053779	123354	estExt_fgenesh2_pg.C_1180037	BflBf_V2_19	963901
5235	ENSG00000118046	STK11	Hsa19	1156798	290116	estExt_gwp.C_10040001	Bflscaffold_1004	14091
5263	ENSG00000037757	NP_001026897	Hsa19	13736360	253882	e_gw.662.13.1	Bflscaffold_662	244056
5274	ENSG00000035664	DAPK2	Hsa15	61986288	232191	e_gw.290.8.1	BflBf_V2_170	74743
5274	ENSG00000167657	DAPK3	Hsa19	3909454	232191	e_gw.290.8.1	BflBf_V2_170	74743
5305	ENSG00000076826	KIAA1543	Hsa19	7566788	63318	fgenesh2_pg.scaffold_1000124	BflBf_V2_258	39640
5305	ENSG00000118200	CAMSAP1L1	Hsa1	198975309	63318	fgenesh2_pg.scaffold_1000124	BflBf_V2_258	39640
5305	ENSG00000130559	CAMSAP1	Hsa9	137842976	63318	fgenesh2_pg.scaffold_1000124	BflBf_V2_258	39640
5324	ENSG00000175221	THRAP5	Hsa19	818961	198705	e_gw.1.659.1	BflBf_V2_107	1789539
5332	ENSG00000197070	ARRDC1	Hsa9	139619917	199194	e_gw.1.402.1	BflBf_V2_107	1243450
5332	ENSG00000205784	645432	Hsa19	4842058	199194	e_gw.1.402.1	BflBf_V2_107	1243450
5365	ENSG00000104774	MAN2B1	Hsa19	12618322	287967	estExt_gwp.C_5620020	BflBf_V2_3	139492
5386	ENSG00000130810	PPAN	Hsa19	10077965	288223	estExt_gwp.C_5870009	Bflscaffold_587	24516
5459	ENSG00000167658	EEF2	Hsa19	3927055	281567	estExt_gwp.C_1070078	Bflscaffold_107	1110372
5487	ENSG00000107362	C9orf77	Hsa9	73667188	112094	fgenesh2_pg.scaffold_1036000002	Bflscaffold_1036	21125
5487	ENSG00000182502	150207	Hsa22	20799236	112094	fgenesh2_pg.scaffold_1036000002	Bflscaffold_1036	21125
5487	ENSG00000136379	Q6PCB6_HUMAN	Hsa15	78774737	112094	fgenesh2_pg.scaffold_1036000002	Bflscaffold_1036	21125
5487	ENSG00000129968	FAM108A1	Hsa19	1827976	112094	fgenesh2_pg.scaffold_1036000002	Bflscaffold_1036	21125
5487	ENSG00000203704	648359	Hsa1	212845423	112094	fgenesh2_pg.scaffold_1036000002	Bflscaffold_1036	21125
5487	ENSG00000198658	Q5RGM9_HUMAN	Hsa1	144787932	112094	fgenesh2_pg.scaffold_1036000002	Bflscaffold_1036	21125
5487	ENSG00000203835	FAM108A2	Hsa1	146084969	112094	fgenesh2_pg.scaffold_1036000002	Bflscaffold_1036	21125
5487	ENSG00000182556		Hsa22	19352115	112094	fgenesh2_pg.scaffold_1036000002	Bflscaffold_1036	21125
5574	ENSG00000160994	CCDC105	Hsa19	14982539	131270	estExt_fgenesh2_pg.C_5150007	Bflscaffold_515	143238
5795	ENSG00000039987	VMD2L1	Hsa19	12724407	275663	estExt_gwp.C_70098	BflBf_V2_196	254626
5795	ENSG00000127325	VMD2L3	Hsa12	68323601	275663	estExt_gwp.C_70098	BflBf_V2_196	254626
5795	ENSG00000142959	VMD2L2	Hsa1	45021844	275663	estExt_gwp.C_70098	BflBf_V2_196	254626
5795	ENSG00000167995	VMD2	Hsa11	61474408	275663	estExt_gwp.C_70098	BflBf_V2_196	254626
5796	ENSG00000167487	KLHL26	Hsa19	18608838	202115	e_gw.7.71.1	BflBf_V2_196	1307834
5796	ENSG00000186231	KIAA1900	Hsa6	97479326	202115	e_gw.7.71.1	BflBf_V2_196	1307834
5839	ENSG00000160293	VAV2	Hsa9	135616837	86548	fgenesh2_pg.scaffold_152000095	Bflscaffold_152	1413606
5839	ENSG00000134215	VAV3	Hsa1	107915305	86548	fgenesh2_pg.scaffold_152000095	Bflscaffold_152	1413606
5839	ENSG00000141968	VAV1	Hsa19	6723722	86548	fgenesh2_pg.scaffold_152000095	Bflscaffold_152	1413606
5861	ENSG00000072958	AP1M1	Hsa19	16169731	270921	estExt_GenewiseH_1.C_4340018	Bflscaffold_434	274739
5861	ENSG00000129354	AP1M2	Hsa19	10544348	270921	estExt_GenewiseH_1.C_4340018	Bflscaffold_434	274739
5861	ENSG00000072958	AP1M1	Hsa19	16169731	270921	estExt_GenewiseH_1.C_4340018	Bflscaffold_434	274739
5861	ENSG00000129354	AP1M2	Hsa19	10544348	270921	estExt_GenewiseH_1.C_4340018	Bflscaffold_434	274739
5864	ENSG00000080503	SMARCA2	Hsa9	2005342	286329	estExt_gwp.C_4340027	Bflscaffold_434	422464
5864	ENSG00000127616	SMARCA4	Hsa19	10932606	286329	estExt_gwp.C_4340027	Bflscaffold_434	422464
5866	ENSG00000130165	ELOF1	Hsa19	11524861	270931	estExt_GenewiseH_1.C_4340032	Bflscaffold_434	502684
5902	ENSG00000105058	FAM32A	Hsa19	16157254	206874	e_gw.31.155.1	BflBf_V2_204	376946
5961	ENSG00000178951	ZBTB7A	Hsa19	3996217	132130	estExt_fgenesh2_pg.C_6390002	Bflscaffold_639	8560
5977	ENSG00000124406	ATP8A1	Hsa4	42105147	69708	fgenesh2_pg.scaffold_24000213	BflBf_V2_150	3626547
5977	ENSG00000132932	ATP8A2	Hsa13	24844209	69708	fgenesh2_pg.scaffold_24000213	BflBf_V2_150	3626547
5977	ENSG00000130270	ATP8B3	Hsa19	1733076	69708	fgenesh2_pg.scaffold_24000213	BflBf_V2_150	3626547
5979	ENSG00000130816	DNMT1	Hsa19	10105023	204680	e_gw.24.105.1	BflBf_V2_150	3120128
6134	ENSG00000185236	RAB11B	Hsa19	8361229	266781	estExt_GenewiseH_1.C_2500058	BflBf_V2_88	2250578
6134	ENSG00000103769	RAB11A	Hsa15	63948850	266781	estExt_GenewiseH_1.C_2500058	BflBf_V2_88	2250578
6134	ENSG00000132698	RAB25	Hsa1	154297575	266781	estExt_GenewiseH_1.C_2500058	BflBf_V2_88	2250578
6136	ENSG00000181035	NP_848621.1	Hsa19	19035808	282150	estExt_gwp.C_2500027	BflBf_V2_88	2087644
6136	ENSG00000122912	SLC25A16	Hsa10	69912104	282150	estExt_gwp.C_2500027	BflBf_V2_88	2087644
6214	ENSG00000116014	KISS1R	Hsa19	868358	137213	gw.611.4.1	Bflscaffold_611	52841
6263	ENSG00000105141	CASP14	Hsa19	15024015	92562	fgenesh2_pg.scaffold_224000042	Bflscaffold_224	1042193
6277	ENSG00000076924	XAB2	Hsa19	7590417	109697	fgenesh2_pg.scaffold_705000001	Bflscaffold_705	11928
6285	ENSG00000105401	CDC37	Hsa19	10362809	115687	estExt_fgenesh2_pm.C_2910001	BflBf_V2_172	200268
6285	ENSG00000106993	CDC37L1	Hsa9	4669559	115687	estExt_fgenesh2_pm.C_2910001	BflBf_V2_172	200268
6297	ENSG00000105612	DNASE2	Hsa19	12847023	229767	e_gw.266.270.1	Bflscaffold_266	1017430
6297	ENSG00000137976	DNASE2B	Hsa1	84636803	229767	e_gw.266.270.1	Bflscaffold_266	1017430
6311	ENSG00000130311	C19orf58	Hsa19	17281350	230591	e_gw.273.31.1	BflBf_V2_124	491784
6312	ENSG00000156642	NPTN	Hsa15	71639418	127723	estExt_fgenesh2_pg.C_2730014	BflBf_V2_124	209588
6312	ENSG00000172270	BSG	Hsa19	462896	127723	estExt_fgenesh2_pg.C_2730014	BflBf_V2_124	209588
6314	ENSG00000138829	FBN2	Hsa5	127621500	127734	estExt_fgenesh2_pg.C_2730038	BflBf_V2_124	669691
6314	ENSG00000166147	FBN1	Hsa15	46489479	127734	estExt_fgenesh2_pg.C_2730038	BflBf_V2_124	669691
6314	ENSG00000142449	FBN3	Hsa19	8036287	127734	estExt_fgenesh2_pg.C_2730038	BflBf_V2_124	669691
6321	ENSG00000105656	ELL	Hsa19	18414475	95758	fgenesh2_pg.scaffold_273000010	BflBf_V2_124	132598
6321	ENSG00000118985	ELL2	Hsa5	95246558	95758	fgenesh2_pg.scaffold_273000010	BflBf_V2_124	132598
6377	ENSG00000072062	PRKACA	Hsa19	14063509	119299	estExt_fgenesh2_pg.C_260217	Bflscaffold_26	3501662
6377	ENSG00000165059	PRKACG	Hsa9	70817241	119299	estExt_fgenesh2_pg.C_260217	Bflscaffold_26	3501662
6377	ENSG00000183943	PRKX	HsaX	3532411	119299	estExt_fgenesh2_pg.C_260217	Bflscaffold_26	3501662
6391	ENSG00000034063	UHRF1	Hsa19	4860513	205176	e_gw.26.91.1	BflBf_V2_135	2787356
6391	ENSG00000147854	UHRF2	Hsa9	6403151	205176	e_gw.26.91.1	BflBf_V2_135	2787356
6400	ENSG00000049192	ADAMTS6	Hsa5	64480322	205307	e_gw.26.85.1	Bflscaffold_26	145
6400	ENSG00000142303	ADAMTS10	Hsa19	8551126	205307	e_gw.26.85.1	Bflscaffold_26	145
6401	ENSG00000105583	C19orf56	Hsa19	12639885	276672	estExt_gwp.C_260088	BflBf_V2_215	1274731
6404	ENSG00000182087	C19orf6	Hsa19	960650	119274	estExt_fgenesh2_pg.C_260097	BflBf_V2_135	2896292
6450	ENSG00000161807	OR7G1	Hsa19	9086274	85038	fgenesh2_pg.scaffold_137000080	Bflscaffold_137	1434351
6450	ENSG00000187847	OR7E24	Hsa19	9222720	85038	fgenesh2_pg.scaffold_137000080	Bflscaffold_137	1434351
6450	ENSG00000127515	OR7A10	Hsa19	14812760	85038	fgenesh2_pg.scaffold_137000080	Bflscaffold_137	1434351
6450	ENSG00000127530	OR7C1	Hsa19	14770986	85038	fgenesh2_pg.scaffold_137000080	Bflscaffold_137	1434351
6450	ENSG00000171501	OR1N2	Hsa9	124355270	85038	fgenesh2_pg.scaffold_137000080	Bflscaffold_137	1434351
6450	ENSG00000184166	OR1D2	Hsa17	2942102	85038	fgenesh2_pg.scaffold_137000080	Bflscaffold_137	1434351
6450	ENSG00000161807	OR7G1	Hsa19	9086274	85038	fgenesh2_pg.scaffold_137000080	Bflscaffold_137	1434351
6450	ENSG00000187847	OR7E24	Hsa19	9222720	85038	fgenesh2_pg.scaffold_137000080	Bflscaffold_137	1434351
6450	ENSG00000127515	OR7A10	Hsa19	14812760	85038	fgenesh2_pg.scaffold_137000080	Bflscaffold_137	1434351
6450	ENSG00000127530	OR7C1	Hsa19	14770986	85038	fgenesh2_pg.scaffold_137000080	Bflscaffold_137	1434351
6450	ENSG00000171501	OR1N2	Hsa9	124355270	85038	fgenesh2_pg.scaffold_137000080	Bflscaffold_137	1434351
6450	ENSG00000184166	OR1D2	Hsa17	2942102	85038	fgenesh2_pg.scaffold_137000080	Bflscaffold_137	1434351
6455	ENSG00000118432	CNR1	Hsa6	88906302	124065	estExt_fgenesh2_pg.C_1370042	BflBf_V2_205	594796
6455	ENSG00000180739	EDG8	Hsa19	10484623	124065	estExt_fgenesh2_pg.C_1370042	BflBf_V2_205	594796

6508	ENSG00000105640	RPL18A	Hsa19	17831731	287192	estExt_gwp.C_4860008	Bflscaffold_486	15773
6508	ENSG00000188096	285053	Hsa2	47771359	287192	estExt_gwp.C_4860008	Bflscaffold_486	15773
6508	ENSG00000186374	127545	Hsa1	33218133	287192	estExt_gwp.C_4860008	Bflscaffold_486	15773
6536	ENSG00000105135	ILVBL	Hsa19	15086789	279682	estExt_gwp.C_680267	BflBf_V2_28	452
6574	ENSG00000140382	HMG20A	Hsa15	75537798	126796	estExt_fgenesh2_pg.C_2300011	BflBf_V2_88	181891
6574	ENSG00000064961	HMG20B	Hsa19	3523955	126796	estExt_fgenesh2_pg.C_2300011	BflBf_V2_88	181891
6579	ENSG00000133243	BTBD2	Hsa19	1937487	62836	fgenesh2_pm.scaffold_767000001	BflBf_V2_124	1041176
6579	ENSG00000064726	BTBD1	Hsa15	81476189	62836	fgenesh2_pm.scaffold_767000001	BflBf_V2_124	1041176
6634	ENSG00000161082	BRUNOL5	Hsa19	3175701	222115	e_gw.91.28.1	Bflscaffold_91	2224591
6634	ENSG00000101489	BRUNOL4	Hsa18	33077828	222115	e_gw.91.28.1	Bflscaffold_91	2224591
6634	ENSG00000048740	CUGBP2	Hsa10	11246999	222115	e_gw.91.28.1	Bflscaffold_91	2224591
6634	ENSG00000149187	CUGBP1	Hsa11	47446522	222115	e_gw.91.28.1	Bflscaffold_91	2224591
6684	ENSG00000172292	LASS6	Hsa2	169021081	246779	e_gw.479.83.1	Bflscaffold_479	149212
6684	ENSG00000090661	LASS4	Hsa19	8180253	246779	e_gw.479.83.1	Bflscaffold_479	149212
6732	ENSG00000130005	GAMT	Hsa19	1348089	80266	fgenesh2_pg.scaffold_92000023	Bflscaffold_92	454695
6848	ENSG00000129933	NP_056144.2	Hsa19	19292644	61744	fgenesh2_pm.scaffold_410000009	BflBf_V2_32	2994116
6850	ENSG00000167491	GATAD2A	Hsa19	19237469	130063	estExt_fgenesh2_pg.C_4100036	BflBf_V2_32	2976163
6850	ENSG00000143614	GATAD2B	Hsa1	152043825	130063	estExt_fgenesh2_pg.C_4100036	BflBf_V2_32	2976163
6857	ENSG00000198722	UNC13B	Hsa9	35151999	102486	fgenesh2_pg.scaffold_410000031	BflBf_V2_32	3039460
6857	ENSG00000137766	UNC13C	Hsa15	52314521	102486	fgenesh2_pg.scaffold_410000031	BflBf_V2_32	3039460
6857	ENSG00000130477	UNC13A	Hsa19	17573167	102486	fgenesh2_pg.scaffold_410000031	BflBf_V2_32	3039460
6887	ENSG00000167733	HSD11B1L	Hsa19	5632035	84745	fgenesh2_pg.scaffold_134000039	BflBf_V2_109	1421796
6907	ENSG00000177606	JUN	Hsa1	59019048	130399	estExt_fgenesh2_pg.C_4350042	BflBf_V2_127	1298867
6907	ENSG00000130522	JUND	Hsa19	18252251	130399	estExt_fgenesh2_pg.C_4350042	BflBf_V2_127	1298867
6907	ENSG00000171223	JUNB	Hsa19	12763286	130399	estExt_fgenesh2_pg.C_4350042	BflBf_V2_127	1298867
6907	ENSG00000177606	JUN	Hsa1	59019048	130399	estExt_fgenesh2_pg.C_4350042	BflBf_V2_127	1298867
6907	ENSG00000130522	JUND	Hsa19	18252251	130399	estExt_fgenesh2_pg.C_4350042	BflBf_V2_127	1298867
6907	ENSG00000171223	JUNB	Hsa19	12763286	130399	estExt_fgenesh2_pg.C_4350042	BflBf_V2_127	1298867
6908	ENSG00000129932	DOHH	Hsa19	3441824	286334	estExt_gwp.C_4350130	Bflscaffold_435	311746
6910	ENSG00000167807	NP_001026904	Hsa19	10281740	103413	fgenesh2_pg.scaffold_435000009	BflBf_V2_127	1544176
6912	ENSG00000117519	CNN3	Hsa1	95135095	61874	fgenesh2_pm.scaffold_435000001	BflBf_V2_127	1574721
6912	ENSG00000130176	CNN1	Hsa19	11510579	61874	fgenesh2_pm.scaffold_435000001	BflBf_V2_127	1574721
6912	ENSG00000064666	CNN2	Hsa19	977298	61874	fgenesh2_pm.scaffold_435000001	BflBf_V2_127	1574721
6912	ENSG00000144834	TAGLN3	Hsa3	113200276	61874	fgenesh2_pm.scaffold_435000001	BflBf_V2_127	1574721
6912	ENSG00000149591	TAGLN	Hsa11	116575250	61874	fgenesh2_pm.scaffold_435000001	BflBf_V2_127	1574721
6912	ENSG00000204826	XR_018140.1	Hsa9	42999838	61874	fgenesh2_pm.scaffold_435000001	BflBf_V2_127	1574721
6912	ENSG00000204782	XR_016647.1	Hsa9	68788495	61874	fgenesh2_pm.scaffold_435000001	BflBf_V2_127	1574721
6912	ENSG00000204718		Hsa2	94766764	61874	fgenesh2_pm.scaffold_435000001	BflBf_V2_127	1574721
6912	ENSG00000117519	CNN3	Hsa1	95135095	61874	fgenesh2_pm.scaffold_435000001	BflBf_V2_127	1574721
6912	ENSG00000130176	CNN1	Hsa19	11510579	61874	fgenesh2_pm.scaffold_435000001	BflBf_V2_127	1574721
6912	ENSG00000064666	CNN2	Hsa19	977298	61874	fgenesh2_pm.scaffold_435000001	BflBf_V2_127	1574721
6912	ENSG00000144834	TAGLN3	Hsa3	113200276	61874	fgenesh2_pm.scaffold_435000001	BflBf_V2_127	1574721
6912	ENSG00000149591	TAGLN	Hsa11	116575250	61874	fgenesh2_pm.scaffold_435000001	BflBf_V2_127	1574721
6912	ENSG00000204826	XR_018140.1	Hsa9	42999838	61874	fgenesh2_pm.scaffold_435000001	BflBf_V2_127	1574721
6912	ENSG00000204782	XR_016647.1	Hsa9	68788495	61874	fgenesh2_pm.scaffold_435000001	BflBf_V2_127	1574721
6912	ENSG00000204718		Hsa2	94766764	61874	fgenesh2_pm.scaffold_435000001	BflBf_V2_127	1574721
6916	ENSG00000173928	C19orf39	Hsa19	11346383	132821	estExt_fgenesh2_pg.C_7970006	BflBf_V2_256	132406
7012	ENSG00000130377	ACSBG2	Hsa19	6086669	254146	e_gw.673.11.1	Bflscaffold_673	214284
7203	ENSG00000099817	POLR2E	Hsa19	1039166	278645	estExt_gwp.C_520097	BflBf_V2_245	1103312
7206	ENSG00000130383	FUT5	Hsa19	5817612	213035	e_gw.52.103.1	BflBf_V2_245	2141177
7206	ENSG00000156413	FUT6	Hsa19	5781770	213035	e_gw.52.103.1	BflBf_V2_245	2141177
7206	ENSG00000180549	FUT7	Hsa9	139044447	213035	e_gw.52.103.1	BflBf_V2_245	2141177
7206	ENSG00000171124	FUT3	Hsa19	5793902	213035	e_gw.52.103.1	BflBf_V2_245	2141177
7206	ENSG00000172461	FUT9	Hsa6	96570581	213035	e_gw.52.103.1	BflBf_V2_245	2141177
7213	ENSG00000184344	GDF3	Hsa12	7733649	278638	estExt_gwp.C_520059	BflBf_V2_245	1612305
7213	ENSG00000130283	GDF1	Hsa19	18840354	278638	estExt_gwp.C_520059	BflBf_V2_245	1612305
7247	ENSG00000174482	LRRN6C	Hsa9	27938076	88487	fgenesh2_pg.scaffold_175000006	BflBf_V2_195	1437660
7247	ENSG00000183014	645191	Hsa19	2240997	88487	fgenesh2_pg.scaffold_175000006	BflBf_V2_195	1437660
7247	ENSG00000169783	LRRN6A	Hsa15	75693441	88487	fgenesh2_pg.scaffold_175000006	BflBf_V2_195	1437660
7248	ENSG00000130299	GTPBP3	Hsa19	17309395	125232	estExt_fgenesh2_pg.C_1750061	BflBf_V2_195	639654
7346	ENSG00000198258	UBL5	Hsa19	9799568	116425	estExt_fgenesh2_pm.C_5530009	Bflscaffold_553	405221
7354	ENSG00000167671	UBXD1	Hsa19	4396261	137183	gw.553.5.1	Bflscaffold_553	73574
7371	ENSG00000080298	RFX3	Hsa9	3208297	130868	estExt_fgenesh2_pg.C_4710022	Bflscaffold_471	219240
7371	ENSG00000087903	RFX2	Hsa19	5944175	130868	estExt_fgenesh2_pg.C_4710022	Bflscaffold_471	219240
7371	ENSG00000132005	RFX1	Hsa19	13933353	130868	estExt_fgenesh2_pg.C_4710022	Bflscaffold_471	219240
7371	ENSG00000080298	RFX3	Hsa9	3208297	130868	estExt_fgenesh2_pg.C_4710022	Bflscaffold_471	219240
7371	ENSG00000087903	RFX2	Hsa19	5944175	130868	estExt_fgenesh2_pg.C_4710022	Bflscaffold_471	219240
7371	ENSG00000132005	RFX1	Hsa19	13933353	130868	estExt_fgenesh2_pg.C_4710022	Bflscaffold_471	219240
7388	ENSG00000198242	RPL23A	Hsa17	24071127	121811	estExt_fgenesh2_pg.C_740009	Bflscaffold_74	144952
7388	ENSG00000129973	XR_018134.1	Hsa3	162629609	121811	estExt_fgenesh2_pg.C_740009	Bflscaffold_74	144952
7388	ENSG00000186587	401904	Hsa19	15583467	121811	estExt_fgenesh2_pg.C_740009	Bflscaffold_74	144952
7388	ENSG00000185822	391282	Hsa21	39421366	121811	estExt_fgenesh2_pg.C_740009	Bflscaffold_74	144952
7388	ENSG00000186698	130773	Hsa2	64427904	121811	estExt_fgenesh2_pg.C_740009	Bflscaffold_74	144952
7388	ENSG00000206147	NP_001039013	Hsa16	376764	121811	estExt_fgenesh2_pg.C_740009	Bflscaffold_74	144952
7388	ENSG00000118975	341511	Hsa12	2761331	121811	estExt_fgenesh2_pg.C_740009	Bflscaffold_74	144952
7388	ENSG00000183102		Hsa3	75756529	121811	estExt_fgenesh2_pg.C_740009	Bflscaffold_74	144952
7388	ENSG00000186239	XR_017413.1	Hsa14	34024936	121811	estExt_fgenesh2_pg.C_740009	Bflscaffold_74	144952
7388	ENSG00000175487	NP_076428.2	Hsa19	63778624	121811	estExt_fgenesh2_pg.C_740009	Bflscaffold_74	144952
7388	ENSG00000176062	650472	Hsa1	247197526	121811	estExt_fgenesh2_pg.C_740009	Bflscaffold_74	144952
7388	ENSG00000184319	NP_976047.1	Hsa22	49569156	121811	estExt_fgenesh2_pg.C_740009	Bflscaffold_74	144952
7388	ENSG00000197413	653341	Hsa21	46935223	121811	estExt_fgenesh2_pg.C_740009	Bflscaffold_74	144952
7388	ENSG00000196573	653341	Hsa1	218155	121811	estExt_fgenesh2_pg.C_740009	Bflscaffold_74	144952
7389	ENSG00000141933	TPGS1_HUMAN	Hsa19	458507	121821	estExt_fgenesh2_pg.C_740022	BflBf_V2_78	800259

Genomic distribution of the paralog groups of the surrounding 10-Mb window including the ALDH1A-related GN in Hsa19, using the Ciona intestinalis genomic database as the outgroup for the best reciprocal BLAST hit analysis								
Genomic regiona analyzed: human chromosome 19 0 - 20000 KB								
Group ID	Query Gene ID	Query Ext ID	Query Chromosome	Query Start Base	Outg Gene ID	Outg Ext ID	Outg Chromosome	Outg Start Base
7	ENSG00000142347	MYO1F	Hsa19	8491689	ENSCING00000003718		Cinscaffold_139	270342
7	ENSG00000157483	MYO1E	Hsa15	57215461	ENSCING00000003718		Cinscaffold_139	270342
15	ENSG00000169231	THBS3	Hsa1	153432003	ENSCING00000008607		Cinscaffold_157	122215
15	ENSG00000113296	THBS4	Hsa5	79366859	ENSCING00000008607		Cinscaffold_157	122215
15	ENSG00000105664	COMP	Hsa19	18754584	ENSCING00000008607		Cinscaffold_157	122215
90	ENSG00000148408	CACNA1B	Hsa9	139892062	ENSCING00000002428		Cin13q	868869
90	ENSG00000141837	CACNA1A	Hsa19	13179088	ENSCING00000002428		Cin13q	868869
90	ENSG00000198216	CACNA1E	Hsa1	179648918	ENSCING00000002428		Cin13q	868869
94	ENSG00000137942	FNBP1L	Hsa1	93686246	ENSCING00000009110		Cin13q	2325867
94	ENSG00000125733	TRIP10	Hsa19	6690721	ENSCING00000009110		Cin13q	2325867
94	ENSG00000187239	FNBP1	Hsa9	131689287	ENSCING00000009110		Cin13q	2325867
103	ENSG00000185630	PBX1	Hsa1	162795496	ENSCING00000009075	Q4H2Y7_CIOIN	Cin13q	2032218
103	ENSG00000167081	PBX3	Hsa9	127548372	ENSCING00000009075	Q4H2Y7_CIOIN	Cin13q	2032218
103	ENSG00000206315	PBX2_HUMAN	Hsac6_QBL	32252994	ENSCING00000009075	Q4H2Y7_CIOIN	Cin13q	2032218
103	ENSG00000206247	PBX2_HUMAN	Hsac6_COX	32248249	ENSCING00000009075	Q4H2Y7_CIOIN	Cin13q	2032218
103	ENSG00000204304	PBX2	Hsa6	32260496	ENSCING00000009075	Q4H2Y7_CIOIN	Cin13q	2032218
103	ENSG00000181814	Q6NW39_HUMAN	Hsa3	144377817	ENSCING00000009075	Q4H2Y7_CIOIN	Cin13q	2032218
103	ENSG00000105717	PBX4	Hsa19	19533524	ENSCING00000009075	Q4H2Y7_CIOIN	Cin13q	2032218
110	ENSG00000130635	COL5A1	Hsa9	136673473	ENSCING00000001528		Cin13q	2437012
110	ENSG00000060718	COL11A1	Hsa1	103114611	ENSCING00000001528		Cin13q	2437012
110	ENSG00000204248	COL11A2	Hsa6	33238447	ENSCING00000001528		Cin13q	2437012
110	ENSG00000112112	Q6LCP7_HUMAN	Hsac6_COX	33199176	ENSCING00000001528		Cin13q	2437012
110	ENSG00000080573	COL5A3	Hsa19	9931237	ENSCING00000001528		Cin13q	2437012
110	ENSG00000196739	COL27A1	Hsa9	115957661	ENSCING00000001528		Cin13q	2437012
110	ENSG00000171502	COL24A1	Hsa1	85967504	ENSCING00000001528		Cin13q	2437012
118	ENSG00000106976	DNM1	Hsa9	130005479	ENSCING00000002282		Cin13q	242760
118	ENSG00000197959	DNM3	Hsa1	170077261	ENSCING00000002282		Cin13q	242760
118	ENSG00000079805	DNM2	Hsa19	10673106	ENSCING00000002282		Cin13q	242760
139	ENSG00000115268	RPS15	Hsa19	1389363	ENSCING00000005083		Cinscaffold_2408	762
139	ENSG00000138396	401019	Hsa2	172082026	ENSCING00000005083		Cinscaffold_2408	762
139	ENSG00000162872	440733	Hsa1	232559073	ENSCING00000005083		Cinscaffold_2408	762
142	ENSG00000099821	POLRMT	Hsa19	568228	ENSCING00000002136		Cinscaffold_65	609615
170	ENSG00000164180	TMEM161B	Hsa5	87526781	ENSCING00000002135		Cinscaffold_65	607113
170	ENSG00000064545	TMEM161A	Hsa19	19091430	ENSCING00000002135		Cinscaffold_65	607113
263	ENSG00000104774	MAN2B1	Hsa19	12618322	ENSCING00000001976		Cin6q	153992
263	ENSG00000013288	MAN2B2	Hsa4	6627803	ENSCING00000001976		Cin6q	153992
285	ENSG00000105648	IFI30	Hsa19	18145579	ENSCING00000003991		Cin6q	1820265
309	ENSG00000159409	TNRC4	Hsa1	149941454	ENSCING00000001999		Cin6q	127361
309	ENSG00000101489	BRUNOL4	Hsa18	33077828	ENSCING00000001999		Cin6q	127361
309	ENSG00000161082	BRUNOL5	Hsa19	3175701	ENSCING00000001999		Cin6q	127361
309	ENSG00000155636	DRBP1_HUMAN	Hsa2	178685520	ENSCING00000001999		Cin6q	127361
340	ENSG00000145779	TNFAIP8	Hsa5	118756403	ENSCING00000013803		Cin1p	2308769
340	ENSG00000183578	TNFAIP8L3	Hsa15	49136093	ENSCING00000013803		Cin1p	2308769
340	ENSG00000185361	TNFAIP8L1	Hsa19	4590530	ENSCING00000013803		Cin1p	2308769
340	ENSG00000163154	TNFAIP8L2	Hsa1	149395729	ENSCING00000013803		Cin1p	2308769
384	ENSG00000154582	TCEB1	Hsa8	75019928	ENSCING00000002160		Cin1p	613447
384	ENSG00000123257	649647	Hsa10	10256199	ENSCING00000002160		Cin1p	613447
384	ENSG00000105694	O75863_HUMAN	Hsa19	2489108	ENSCING00000002160		Cin1p	613447
387	ENSG00000120992	LYPLA1	Hsa8	55121492	ENSCING00000002678		Cin1p	1764744
387	ENSG00000011009	LYPLA2	Hsa1	23990047	ENSCING00000002678		Cin1p	1764744
387	ENSG00000206204	NR_001444.2	Hsac6_COX	33402009	ENSCING00000002678		Cin1p	1764744
387	ENSG00000204198	NR_001444.2	Hsa6	33441303	ENSCING00000002678		Cin1p	1764744
387	ENSG00000206276		Hsac6_QBL	33405022	ENSCING00000002678		Cin1p	1764744
387	ENSG00000205677	388499	Hsa19	7850330	ENSCING00000002678		Cin1p	1764744
387	ENSG00000143353	LYPLAL1	Hsa1	217413809	ENSCING00000002678		Cin1p	1764744
399	ENSG00000096968	JAK2	Hsa9	4975245	ENSCING00000005294	Q4H3A3_CIOIN	Cin1p	4333821
399	ENSG00000105639	JAK3	Hsa19	17788324	ENSCING00000005294	Q4H3A3_CIOIN	Cin1p	4333821
399	ENSG00000105397	TYK2	Hsa19	10322205	ENSCING00000005294	Q4H3A3_CIOIN	Cin1p	4333821
399	ENSG00000162434	JAK1	Hsa1	65071500	ENSCING00000005294	Q4H3A3_CIOIN	Cin1p	4333821
399	ENSG00000096968	JAK2	Hsa9	4975245	ENSCING00000005294	Q4H3A3_CIOIN	Cin1p	4333821
399	ENSG00000105639	JAK3	Hsa19	17788324	ENSCING00000005294	Q4H3A3_CIOIN	Cin1p	4333821
399	ENSG00000105397	TYK2	Hsa19	10322205	ENSCING00000005294	Q4H3A3_CIOIN	Cin1p	4333821
399	ENSG00000162434	JAK1	Hsa1	65071500	ENSCING00000005294	Q4H3A3_CIOIN	Cin1p	4333821
456	ENSG00000103942	HOMER2	Hsa15	81314792	ENSCING00000013854		Cin1p	746934
456	ENSG00000152413	HOMER1	Hsa5	78707505	ENSCING00000013854		Cin1p	746934
456	ENSG00000051128	HOMER3	Hsa19	18901047	ENSCING00000013854		Cin1p	746934
458	ENSG00000185043	CIB1	Hsa15	88574481	ENSCING00000013867		Cin1p	356052
458	ENSG00000136425	CIB2	Hsa15	76184046	ENSCING00000013867		Cin1p	356052
458	ENSG00000141977	CIB3	Hsa19	16133179	ENSCING00000013867		Cin1p	356052
458	ENSG00000157884	NP_001025052	Hsa2	26657577	ENSCING00000013867		Cin1p	356052
491	ENSG00000162701	DENND1B	Hsa1	195745397	ENSCING00000003894		Cin1p	3861579
491	ENSG00000205744	DENND1C	Hsa19	6418219	ENSCING00000003894		Cin1p	3861579
491	ENSG00000162777	DENND2D	Hsa1	111531319	ENSCING00000003894		Cin1p	3861579
518	ENSG00000115286	NDUFS7	Hsa19	1334883	ENSCING00000002646		Cin1p	1722648
606	ENSG00000157216	SSBP3	Hsa1	54464783	ENSCING00000000979		Cinscaffold_205	93703
606	ENSG00000145687	SSBP2	Hsa5	80744596	ENSCING00000000979		Cinscaffold_205	93703
606	ENSG00000130511	SSBP4	Hsa19	18391136	ENSCING00000000979		Cinscaffold_205	93703
609	ENSG00000099795	NDUFB7	Hsa19	14537892	ENSCING00000016442		Cinscaffold_2455	5426
622	ENSG00000196365	PRSS15	Hsa19	5642845	ENSCING00000009166		Cin4q	6332869
639	ENSG00000185761	ADAMTSL5	Hsa19	1456023	ENSCING00000013617		Cin4q	1429918
673	ENSG00000183186	KIAA1957	Hsa19	356508	ENSCING00000008873		Cin4q	4126021
685	ENSG00000105669	COPE	Hsa19	18871323	ENSCING00000005338		Cin4q	6150375

722	ENSG00000133275	CSNK1G2	Hsa19	1892188	ENSCING00000008975		Cin4q	517019
722	ENSG00000151292	CSNK1G3	Hsa5	122909227	ENSCING00000008975		Cin4q	517019
722	ENSG00000169118	CSNK1G1	Hsa15	62244771	ENSCING00000008975		Cin4q	517019
766	ENSG00000081923	ATP8B1	Hsa18	53466591	ENSCING00000006046		Cin4q	3332533
766	ENSG00000104043	ATP8B4	Hsa15	47937727	ENSCING00000006046		Cin4q	3332533
766	ENSG00000143515	ATP8B2	Hsa1	152564654	ENSCING00000006046		Cin4q	3332533
766	ENSG00000130270	ATP8B3	Hsa19	1733076	ENSCING00000006046		Cin4q	3332533
766	ENSG00000068650	ATP11A	Hsa13	112392644	ENSCING00000006046		Cin4q	3332533
766	ENSG00000101974	ATP11C	HsaX	138636171	ENSCING00000006046		Cin4q	3332533
795	ENSG00000125912	NCLN	Hsa19	3136875	ENSCING00000008655		Cin4q	5799624
803	ENSG00000141985	SH3GL1	Hsa19	4311370	ENSCING00000003595		Cin4q	3104132
803	ENSG00000107295	SH3GL2	Hsa9	17569121	ENSCING00000003595		Cin4q	3104132
803	ENSG00000140600	SH3GL3	Hsa15	81907287	ENSCING00000003595		Cin4q	3104132
821	ENSG00000106829	TLE4	Hsa9	81376508	ENSCING00000008703	Q4H3E9_CIOIN	Cin4q	5403681
821	ENSG00000140332	TLE3	Hsa15	68129473	ENSCING00000008703	Q4H3E9_CIOIN	Cin4q	5403681
821	ENSG00000196781	TLE1	Hsa9	83388418	ENSCING00000008703	Q4H3E9_CIOIN	Cin4q	5403681
821	ENSG00000065717	TLE2	Hsa19	2948637	ENSCING00000008703	Q4H3E9_CIOIN	Cin4q	5403681
829	ENSG00000123154	MORG1_HUMAN	Hsa19	12641567	ENSCING00000003306		Cin4q	2926590
835	ENSG00000171843	MLLT3	Hsa9	20331663	ENSCING00000006124		Cin4q	3364689
835	ENSG00000130382	MLLT1	Hsa19	6163966	ENSCING00000006124		Cin4q	3364689
859	ENSG00000005206	PSL1_HUMAN	Hsa19	2279680	ENSCING00000008845		Cin4q	4423776
859	ENSG00000138600	PSL2_HUMAN	Hsa15	48787057	ENSCING00000008845		Cin4q	4423776
861	ENSG00000105676	ARMC6	Hsa19	19005538	ENSCING00000001897		Cin4q	2534035
864	ENSG00000105671	DDX49	Hsa19	18891494	ENSCING00000008831		Cin4q	4653690
907	ENSG00000133243	BTBD2	Hsa19	1937487	ENSCING00000013464		Cin4q	4710911
907	ENSG00000064726	BTBD1	Hsa15	81476189	ENSCING00000013464		Cin4q	4710911
909	ENSG00000196084	UBA52	Hsa19	18543614	ENSCING00000005008		Cin4q	3541406
1007	ENSG00000105248	CCDC94	Hsa19	4198087	ENSCING00000014445		Cin14q	970333
1013	ENSG00000143549	TPM3	Hsa1	152395457	ENSCING00000002349	TPM1_CIOIN	Cin14q	3018755
1013	ENSG00000198467	TPM2	Hsa9	35671989	ENSCING00000002349	TPM1_CIOIN	Cin14q	3018755
1013	ENSG00000140416	TPM1	Hsa15	61121891	ENSCING00000002349	TPM1_CIOIN	Cin14q	3018755
1013	ENSG00000167460	TPM4	Hsa19	16039348	ENSCING00000002349	TPM1_CIOIN	Cin14q	3018755
1013	ENSG00000187536		Hsa16	51246717	ENSCING00000002349	TPM1_CIOIN	Cin14q	3018755
1153	ENSG00000156052	GNAQ	Hsa9	79520823	ENSCING00000003915	Q8WSR9_CIOIN	Cin3p	2005162
1153	ENSG00000088256	GNA11	Hsa19	3045408	ENSCING00000003915	Q8WSR9_CIOIN	Cin3p	2005162
1153	ENSG00000156049	GNA14	Hsa9	79227815	ENSCING00000003915	Q8WSR9_CIOIN	Cin3p	2005162
1153	ENSG00000060558	GNA15	Hsa19	3087230	ENSCING00000003915	Q8WSR9_CIOIN	Cin3p	2005162
1164	ENSG00000131788	PIAS3	Hsa1	144287346	ENSCING00000011724	Q4H2Y4_CIOIN	Cin3p	2565718
1164	ENSG00000078043	PIAS2	Hsa18	42646058	ENSCING00000011724	Q4H2Y4_CIOIN	Cin3p	2565718
1164	ENSG00000033800	PIAS1	Hsa15	66165695	ENSCING00000011724	Q4H2Y4_CIOIN	Cin3p	2565718
1164	ENSG00000105229	PIAS4	Hsa19	3958748	ENSCING00000011724	Q4H2Y4_CIOIN	Cin3p	2565718
1174	ENSG00000198722	UNC13B	Hsa9	35151999	ENSCING00000002173		Cin3p	1457745
1174	ENSG00000137766	UNC13C	Hsa15	52314521	ENSCING00000002173		Cin3p	1457745
1174	ENSG00000130477	UNC13A	Hsa19	17573167	ENSCING00000002173		Cin3p	1457745
1189	ENSG00000166573	GALR1	Hsa18	73090721	ENSCING00000002192		Cin3p	1546478
1189	ENSG00000182687	GALR2	Hsa17	71582479	ENSCING00000002192		Cin3p	1546478
1189	ENSG00000128310	GALR3	Hsa22	36549335	ENSCING00000002192		Cin3p	1546478
1189	ENSG00000116014	KISS1R	Hsa19	868358	ENSCING00000002192		Cin3p	1546478
1189	ENSG00000134817	AGTRL1	Hsa11	56757630	ENSCING00000002192		Cin3p	1546478
1189	ENSG00000160791	CCR5	Hsa3	46386637	ENSCING00000002192		Cin3p	1546478
1191	ENSG00000172031	ABHD7	Hsa1	92268127	ENSCING00000000384		Cin3p	2924132
1191	ENSG00000105131	ABHD9	Hsa19	15198731	ENSCING00000000384		Cin3p	2924132
1212	ENSG00000105701	FKBP8	Hsa19	18503568	ENSCING00000011704		Cin3p	3031145
1234	ENSG00000143418	LASS2	Hsa1	149204286	ENSCING00000007135	Q4H395_CIOIN	Cin3p	1259787
1234	ENSG00000139624	LASS5	Hsa12	48809849	ENSCING00000007135	Q4H395_CIOIN	Cin3p	1259787
1234	ENSG00000090661	LASS4	Hsa19	8180253	ENSCING00000007135	Q4H395_CIOIN	Cin3p	1259787
1234	ENSG00000154227	LASS3	Hsa15	98758124	ENSCING00000007135	Q4H395_CIOIN	Cin3p	1259787
1280	ENSG00000105549	THEG	Hsa19	313059	ENSCING00000006809		Cinscaffold_198	79114
1283	ENSG00000127325	VMD2L3	Hsa12	68323601	ENSCING00000006827		Cinscaffold_198	4784
1283	ENSG00000167995	VMD2	Hsa11	61474408	ENSCING00000006827		Cinscaffold_198	4784
1283	ENSG00000039987	VMD2L1	Hsa19	12724407	ENSCING00000006827		Cinscaffold_198	4784
1283	ENSG00000142959	VMD2L2	Hsa1	45021844	ENSCING00000006827		Cinscaffold_198	4784
1303	ENSG00000037757	NP_001026897	Hsa19	13736360	ENSCING00000000162		Cinscaffold_805	5024
1308	ENSG00000068305	MEF2A	Hsa15	97923712	ENSCING00000000628	Q4H375_CIOIN	Cinscaffold_187	72757
1308	ENSG00000081189	MEF2C	Hsa5	88051922	ENSCING00000000628	Q4H375_CIOIN	Cinscaffold_187	72757
1308	ENSG00000116604	MEF2D	Hsa1	154700143	ENSCING00000000628	Q4H375_CIOIN	Cinscaffold_187	72757
1308	ENSG00000064489	MEF2B	Hsa19	19117379	ENSCING00000000628	Q4H375_CIOIN	Cinscaffold_187	72757
1322	ENSG00000205784	645432	Hsa19	4842058	ENSCING00000015247		Cinscaffold_71	365335
1358	ENSG00000074603	DPP8	Hsa15	63526028	ENSCING00000005875		Cinscaffold_96	316278
1358	ENSG00000142002	DPP9	Hsa19	4626237	ENSCING00000005875		Cinscaffold_96	316278
1438	ENSG00000099822	HCN2	Hsa19	540893	ENSCING00000005528		Cin10q	1167144
1438	ENSG00000164588	HCN1	Hsa5	45297730	ENSCING00000005528		Cin10q	1167144
1438	ENSG00000143630	HCN3	Hsa1	153513998	ENSCING00000005528		Cin10q	1167144
1491	ENSG00000104760	FGL1	Hsa8	17766169	ENSCING00000005465		Cin10q	2189665
1491	ENSG00000091879	ANGPT2	Hsa8	6344580	ENSCING00000005465		Cin10q	2189665
1491	ENSG00000187151	ANGPTL5	Hsa11	101266616	ENSCING00000005465		Cin10q	2189665
1491	ENSG00000167772	ANGPTL4	Hsa19	8335011	ENSCING00000005465		Cin10q	2189665
1498	ENSG00000129946	SHC2	Hsa19	367593	ENSCING00000006400		Cin10q	2884248
1498	ENSG00000148082	SHC3	Hsa9	90817880	ENSCING00000006400		Cin10q	2884248
1498	ENSG00000160691	SHC1	Hsa1	153201398	ENSCING00000006400		Cin10q	2884248
1498	ENSG00000185634	SHC4	Hsa15	46903227	ENSCING00000006400		Cin10q	2884248
1533	ENSG00000156413	FUT6	Hsa19	5781770	ENSCING00000005523		Cin10q	1133998
1608	ENSG00000125651	GTF2F1	Hsa19	6235811	ENSCING00000001776		Cin1q	6796943
1633	ENSG00000106804	C5	Hsa9	122754437	ENSCING00000001054		Cin1q	5196768
1633	ENSG00000206264	NP_001002029	Hsac6_COX	32084607	ENSCING00000001054		Cin1q	5196768
1633	ENSG00000204342	C4B	Hsa6	32057780	ENSCING00000001054		Cin1q	5196768

1633	ENSG00000125730	C3	Hsa19	6628878	ENSCING00000001054		Cin1q	5196768
1633	ENSG00000204319	C4A	Hsa6	32090517	ENSCING00000001054		Cin1q	5196768
1660	ENSG00000198356	ASNA1	Hsa19	12709306	ENSCING00000012910		Cin1q	206196
1676	ENSG00000206445	UAP56_HUMAN	Hsac6_QBL	31631137	ENSCING00000005175		Cin1q	736973
1676	ENSG00000206334	UAP56_HUMAN	Hsac6_COX	31632712	ENSCING00000005175		Cin1q	736973
1676	ENSG00000198563	BAT1	Hsa6	31605975	ENSCING00000005175		Cin1q	736973
1676	ENSG00000123136	DDX39	Hsa19	14380632	ENSCING00000005175		Cin1q	736973
1704	ENSG00000095932	O75264_HUMAN	Hsa19	3425750	ENSCING00000001771		Cin1q	6807768
1742	ENSG00000136404	TM6SF1	Hsa15	81567328	ENSCING00000006087		Cin1q	5593767
1742	ENSG00000187664	TM6SF2	Hsa19	19226557	ENSCING00000006087		Cin1q	5593767
1795	ENSG00000011304	PTBP1	Hsa19	748411	ENSCING00000007912		Cin1q	2024764
1795	ENSG00000117569	PTBP2	Hsa1	96959927	ENSCING00000007912		Cin1q	2024764
1795	ENSG00000119314	ROD1	Hsa9	114020536	ENSCING00000007912		Cin1q	2024764
1795	ENSG00000143889	HNRPLL	Hsa2	38642624	ENSCING00000007912		Cin1q	2024764
1795	ENSG00000104824	HNRPL	Hsa19	44018869	ENSCING00000007912		Cin1q	2024764
1803	ENSG00000170144	HNRPA3	Hsa2	177785774	ENSCING00000004062		Cin1q	4720975
1803	ENSG00000176825		Hsa3	75346391	ENSCING00000004062		Cin1q	4720975
1803	ENSG00000135486	HNRPA1	Hsa12	52960755	ENSCING00000004062		Cin1q	4720975
1803	ENSG00000122566	HNRPA2B1	Hsa7	26196459	ENSCING00000004062		Cin1q	4720975
1803	ENSG00000139675	NP_001011725	Hsa13	52089606	ENSCING00000004062		Cin1q	4720975
1803	ENSG00000197029	402562	Hsa7	84450808	ENSCING00000004062		Cin1q	4720975
1803	ENSG00000187999	651179	Hsa19	11637988	ENSCING00000004062		Cin1q	4720975
1803	ENSG00000196682	120364	Hsa11	110163965	ENSCING00000004062		Cin1q	4720975
1803	ENSG00000177733	HNRPA0	Hsa5	137116737	ENSCING00000004062		Cin1q	4720975
1803	ENSG00000177219	Q65ZQ3_HUMAN	Hsa10	43605032	ENSCING00000004062		Cin1q	4720975
1803	ENSG00000176757	648210	Hsa5	79690652	ENSCING00000004062		Cin1q	4720975
1803	ENSG00000181836	Q5T6S7_HUMAN	Hsa13	65260065	ENSCING00000004062		Cin1q	4720975
1845	ENSG00000184162	NP_795361.1	Hsa19	19173734	ENSCING00000009832		Cin1q	1425230
1867	ENSG00000170340	B3GNT2	Hsa2	62276766	ENSCING00000005729		Cin1q	5407909
1867	ENSG00000172318	B3GALT1	Hsa2	168383428	ENSCING00000005729		Cin1q	5407909
1867	ENSG00000156966	B3GNT7	Hsa2	231970675	ENSCING00000005729		Cin1q	5407909
1867	ENSG00000179913	B3GNT3	Hsa19	17766658	ENSCING00000005729		Cin1q	5407909
1867	ENSG00000176383	B3GNT4	Hsa12	121254043	ENSCING00000005729		Cin1q	5407909
1867	ENSG00000176597	B3GNT5	Hsa3	184453739	ENSCING00000005729		Cin1q	5407909
1867	ENSG00000198488	B3GNT6	Hsa11	76423108	ENSCING00000005729		Cin1q	5407909
1867	ENSG00000162630	B3GALT2	Hsa1	191414798	ENSCING00000005729		Cin1q	5407909
1867	ENSG00000183778	B3GALT5	Hsa21	39850239	ENSCING00000005729		Cin1q	5407909
1867	ENSG00000177191	B3GNT8	Hsa19	46623105	ENSCING00000005729		Cin1q	5407909
1867	ENSG00000169255	B3GALNT1	Hsa3	162284365	ENSCING00000005729		Cin1q	5407909
1867	ENSG00000206285	B3GT4_HUMAN	Hsac6_QBL	33316612	ENSCING00000005729		Cin1q	5407909
1867	ENSG00000204222	B3GALT4	Hsa6	33352951	ENSCING00000005729		Cin1q	5407909
1872	ENSG00000118432	CNR1	Hsa6	88906302	ENSCING00000007801	NP_001027653	Cin1q	1607564
1872	ENSG00000198121	EDG2	Hsa9	112675364	ENSCING00000007801	NP_001027653	Cin1q	1607564
1872	ENSG00000171517	EDG7	Hsa1	85049873	ENSCING00000007801	NP_001027653	Cin1q	1607564
1872	ENSG00000186354	C9orf47	Hsa9	90795598	ENSCING00000007801	NP_001027653	Cin1q	1607564
1872	ENSG00000170989	EDG1	Hsa1	101475032	ENSCING00000007801	NP_001027653	Cin1q	1607564
1872	ENSG00000188822	CNR2	Hsa1	24069603	ENSCING00000007801	NP_001027653	Cin1q	1607564
1872	ENSG00000125910	EDG6	Hsa19	3129766	ENSCING00000007801	NP_001027653	Cin1q	1607564
1888	ENSG00000082996	RNF13	Hsa3	151013194	ENSCING00000012489		Cin1q	4930166
1888	ENSG00000108523	RNF167	Hsa17	4784048	ENSCING00000012489		Cin1q	4930166
1888	ENSG00000105428	ZNRF4	Hsa19	5406442	ENSCING00000012489		Cin1q	4930166
1931	ENSG00000103653	CSK	Hsa15	72861489	ENSCING00000003438		Cinscaffold_95	33992
1931	ENSG00000007264	MATK	Hsa19	3728968	ENSCING00000003438		Cinscaffold_95	33992
1932	ENSG00000105135	ILVBL	Hsa19	15086789	ENSCING00000000404		Cinscaffold_95	405159
1940	ENSG00000198242	RPL23A	Hsa17	24071127	ENSCING00000010522		Cinscaffold_95	450869
1940	ENSG00000129973	XR_018134.1	Hsa3	162629609	ENSCING00000010522		Cinscaffold_95	450869
1940	ENSG00000186587	401904	Hsa19	15583467	ENSCING00000010522		Cinscaffold_95	450869
1940	ENSG00000185822	391282	Hsa21	39421366	ENSCING00000010522		Cinscaffold_95	450869
1940	ENSG00000186698	130773	Hsa2	64427904	ENSCING00000010522		Cinscaffold_95	450869
1940	ENSG00000118975	341511	Hsa12	2761331	ENSCING00000010522		Cinscaffold_95	450869
1940	ENSG00000206147	NP_001039013	Hsa16	376764	ENSCING00000010522		Cinscaffold_95	450869
1940	ENSG00000183102		Hsa3	75756529	ENSCING00000010522		Cinscaffold_95	450869
1940	ENSG00000186239	XR_017413.1	Hsa14	34024936	ENSCING00000010522		Cinscaffold_95	450869
1940	ENSG00000175487	NP_076428.2	Hsa19	63778624	ENSCING00000010522		Cinscaffold_95	450869
1940	ENSG00000176062	650472	Hsa1	247197526	ENSCING00000010522		Cinscaffold_95	450869
1940	ENSG00000184319	NP_976047.1	Hsa22	49569156	ENSCING00000010522		Cinscaffold_95	450869
1940	ENSG00000197413	653341	Hsa21	46935223	ENSCING00000010522		Cinscaffold_95	450869
1940	ENSG00000196573	653341	Hsa1	218155	ENSCING00000010522		Cinscaffold_95	450869
1940	ENSG00000184423		Hsa3	1612458	ENSCING00000010522		Cinscaffold_95	450869
1950	ENSG00000137714	FDX1	Hsa11	109805804	ENSCING00000007094		Cinscaffold_63	596197
1950	ENSG00000181741		Hsa20	32527497	ENSCING00000007094		Cinscaffold_63	596197
1950	ENSG00000167807	NP_001026904	Hsa19	10281740	ENSCING00000007094		Cinscaffold_63	596197
1964	ENSG00000113369	ARRDC3	Hsa5	90700299	ENSCING00000015335		Cinscaffold_63	177428
1964	ENSG00000105643	ARRDC2	Hsa19	17972944	ENSCING00000015335		Cinscaffold_63	177428
1964	ENSG00000140450	ARRDC4	Hsa15	96304947	ENSCING00000015335		Cinscaffold_63	177428
2029	ENSG00000129625	REEP5	Hsa5	112239983	ENSCING00000000719		Cin12p	1644701
2029	ENSG00000115255	REEP6	Hsa19	1442165	ENSCING00000000719		Cin12p	1644701
2029	ENSG00000132563	REEP2	Hsa5	137802636	ENSCING00000000719		Cin12p	1644701
2029	ENSG00000068615	REEP1	Hsa2	86294633	ENSCING00000000719		Cin12p	1644701
2029	ENSG00000168476	REEP4	Hsa8	22051478	ENSCING00000000719		Cin12p	1644701
2029	ENSG00000165476	REEP3	Hsa10	64951179	ENSCING00000000719		Cin12p	1644701
2075	ENSG00000113368	LMNB1	Hsa5	126140214	ENSCING00000000897	Q9GNN4_CIOIN	Cin12p	905718
2075	ENSG00000160789	LMNA	Hsa1	154318993	ENSCING00000000897	Q9GNN4_CIOIN	Cin12p	905718
2075	ENSG00000176619	LMNB2	Hsa19	2379165	ENSCING00000000897	Q9GNN4_CIOIN	Cin12p	905718
2116	ENSG00000176624	RKHD2	Hsa18	46954920	ENSCING00000002603		Cinscaffold_818	6844
2116	ENSG00000183496	RKHD3	Hsa15	80121183	ENSCING00000002603		Cinscaffold_818	6844

2116	ENSG00000181588	RKHD1	Hsa19	1505672	ENSCING00000002603		Cinscaffold_818	6844
2131	ENSG00000130304	SLC27A1	Hsa19	17442350	ENSCING00000005316		Cinscaffold_165	179693
2131	ENSG00000167114	SLC27A4	Hsa9	130142661	ENSCING00000005316		Cinscaffold_165	179693
2147	ENSG00000105255	FSD1	Hsa19	4255668	ENSCING00000000895		Cinscaffold_1914	25
2147	ENSG00000106701	FSD1L	Hsa9	107250147	ENSCING00000000895		Cinscaffold_1914	25
2257	ENSG00000130005	GAMT	Hsa19	1348089	ENSCING00000014902		Cin14p	597785
2261	ENSG00000077463	SIRT6	Hsa19	4125106	ENSCING00000001266		Cin14p	312558
2317	ENSG00000140479	PCSK6	Hsa15	99661657	ENSCING00000005033		Cinscaffold_152	114187
2317	ENSG00000099139	PCSK5	Hsa9	77695406	ENSCING00000005033		Cinscaffold_152	114187
2317	ENSG00000140564	FURIN	Hsa15	89212889	ENSCING00000005033		Cinscaffold_152	114187
2317	ENSG00000115257	PCSK4	Hsa19	1432427	ENSCING00000005033		Cinscaffold_152	114187
2317	ENSG00000175426	PCSK1	Hsa5	95751875	ENSCING00000005033		Cinscaffold_152	114187
2354	ENSG00000104907	TRMT1	Hsa19	13076721	ENSCING00000002884		Cinscaffold_119	120698
2416	ENSG00000104177	MYEF2	Hsa15	46219719	ENSCING00000003046		Cinscaffold_55	638793
2416	ENSG00000099783	HNRPM	Hsa19	8415651	ENSCING00000003046		Cinscaffold_55	638793
2422	ENSG00000167654	ATCAY	Hsa19	3831672	ENSCING00000016431		Cinscaffold_55	562345
2422	ENSG00000140299	BNIP2	Hsa15	57742356	ENSCING00000016431		Cinscaffold_55	562345
2422	ENSG00000163141	BNIPL	Hsa1	149275670	ENSCING00000016431		Cinscaffold_55	562345
2428	ENSG00000188229	TUBB2C	Hsa9	139255532	ENSCING00000007589		Cinscaffold_78	297537
2428	ENSG00000196230	TUBB	Hsa6	30796101	ENSCING00000007589		Cinscaffold_78	297537
2428	ENSG00000183311	TBB2_HUMAN	Hsac6_QBL	30820521	ENSCING00000007589		Cinscaffold_78	297537
2428	ENSG00000137379	TBB2_HUMAN	Hsac6_COX	30825252	ENSCING00000007589		Cinscaffold_78	297537
2428	ENSG00000104833	TUBB4	Hsa19	6445331	ENSCING00000007589		Cinscaffold_78	297537
2428	ENSG00000198211	MC1R	Hsa16	88513168	ENSCING00000007589		Cinscaffold_78	297537
2428	ENSG00000176014	TUBB6	Hsa18	12298215	ENSCING00000007589		Cinscaffold_78	297537
2428	ENSG00000173876	NP_817124.1	Hsa10	82832	ENSCING00000007589		Cinscaffold_78	297537
2428	ENSG00000173213		Hsa18	37358	ENSCING00000007589		Cinscaffold_78	297537
2428	ENSG00000159247	643224	Hsa9	140189314	ENSCING00000007589		Cinscaffold_78	297537
2428	ENSG00000127589	TUBB4Q	Hsa4	191140672	ENSCING00000007589		Cinscaffold_78	297537
2428	ENSG00000101162	TUBB1	Hsa20	57027704	ENSCING00000007589		Cinscaffold_78	297537
2540	ENSG00000188181	126037	Hsa19	14042210	ENSCING00000003028		Cin2q	6458688
2583	ENSG00000065268	WDR18	Hsa19	935328	ENSCING00000011321		Cin2q	7890410
2688	ENSG00000105641	SLC5A5	Hsa19	17843782	ENSCING00000006397		Cin2q	5045647
2719	ENSG00000032444	PNPLA6	Hsa19	7505075	ENSCING00000006539		Cin2q	1107736
2719	ENSG00000130653	PNPLA7	Hsa9	139474225	ENSCING00000006539		Cin2q	1107736
2722	ENSG00000064547	EDG4	Hsa19	19595478	ENSCING00000005899		Cin2q	7366401
2723	ENSG00000053501	USE1_HUMAN	Hsa19	17187168	ENSCING00000011584		Cin2q	1566723
2738	ENSG00000107099	DOCK8	Hsa9	261625	ENSCING00000007422		Cin2q	1346893
2738	ENSG00000116641	DOCK7	Hsa1	62692987	ENSCING00000007422		Cin2q	1346893
2738	ENSG00000130158	DOCK6	Hsa19	11170973	ENSCING00000007422		Cin2q	1346893
2739	ENSG00000095209	TMEM38B	Hsa9	107496646	ENSCING00000006561		Cin2q	1174696
2739	ENSG00000072954	TMEM38A	Hsa19	16632938	ENSCING00000006561		Cin2q	1174696
2741	ENSG00000099797	GPSN2	Hsa19	14501382	ENSCING00000004183		Cin2q	381799
2741	ENSG00000205678	NP_001010874	Hsa4	64828385	ENSCING00000004183		Cin2q	381799
2801	ENSG00000107362	C9orf77	Hsa9	73667188	ENSCING00000008539		Cin2q	4528155
2801	ENSG00000182556		Hsa22	19352115	ENSCING00000008539		Cin2q	4528155
2801	ENSG00000182502	150207	Hsa22	20799236	ENSCING00000008539		Cin2q	4528155
2801	ENSG00000136379	Q6PCB6_HUMAN	Hsa15	78774737	ENSCING00000008539		Cin2q	4528155
2801	ENSG00000129968	FAM108A1	Hsa19	1827976	ENSCING00000008539		Cin2q	4528155
2801	ENSG00000203835	FAM108A2	Hsa1	146084969	ENSCING00000008539		Cin2q	4528155
2801	ENSG00000198658	Q5RGM9_HUMAN	Hsa1	144787932	ENSCING00000008539		Cin2q	4528155
2801	ENSG00000203704	648359	Hsa1	212845423	ENSCING00000008539		Cin2q	4528155
2804	ENSG00000169375	SIN3A	Hsa15	73450330	ENSCING00000009117		Cin2q	6323167
2804	ENSG00000127511	SIN3B	Hsa19	16801211	ENSCING00000009117		Cin2q	6323167
2825	ENSG00000130313	PGLS	Hsa19	17483432	ENSCING00000007444		Cin2q	1581340
2836	ENSG00000141994	DUS3L	Hsa19	5736155	ENSCING00000004940		Cin2q	8034946
2850	ENSG00000130299	GTPBP3	Hsa19	17309395	ENSCING00000005867		Cin2q	3147390
2926	ENSG00000147403	RPL10	HsaX	153279765	ENSCING00000003982		Cin12q	3512089
2926	ENSG00000178464	XR_017237.1	Hsa19	12615089	ENSCING00000003982		Cin12q	3512089
2926	ENSG00000165496	RPL10L	Hsa14	46190045	ENSCING00000003982		Cin12q	3512089
2926	ENSG00000174572	389342	Hsa5	167975935	ENSCING00000003982		Cin12q	3512089
3008	ENSG00000151726	ACSL1	Hsa4	185913744	ENSCING00000002865		Cin12q	4221590
3008	ENSG00000164398	ACSL6	Hsa5	131170735	ENSCING00000002865		Cin12q	4221590
3008	ENSG00000197142	ACSL5	Hsa10	114123766	ENSCING00000002865		Cin12q	4221590
3008	ENSG00000123983	ACSL3	Hsa2	223433976	ENSCING00000002865		Cin12q	4221590
3008	ENSG00000068366	ACSL4	HsaX	108771220	ENSCING00000002865		Cin12q	4221590
3008	ENSG00000103740	ACSBG1	Hsa15	76246865	ENSCING00000002865		Cin12q	4221590
3008	ENSG00000130377	ACSBG2	Hsa19	6086669	ENSCING00000002865		Cin12q	4221590
3016	ENSG00000122912	SLC25A16	Hsa10	69912104	ENSCING00000001635		Cin12q	276532
3016	ENSG00000181035	NP_848621.1	Hsa19	19035808	ENSCING00000001635		Cin12q	276532
3016	ENSG00000077713	NP_660348.1	HsaX	118397679	ENSCING00000001635		Cin12q	276532
3091	ENSG00000137094	DNAJB5	Hsa9	34979742	ENSCING00000006225		Cinscaffold_36	194004
3091	ENSG00000162616	DNAJB4	Hsa1	78243126	ENSCING00000006225		Cinscaffold_36	194004
3091	ENSG00000132002	DNAJB1	Hsa19	14486582	ENSCING00000006225		Cinscaffold_36	194004
3105	ENSG00000104889	RNASEH2A	Hsa19	12778488	ENSCING00000006190		Cinscaffold_36	308369
3113	ENSG00000179115	FARSLA	Hsa19	12894294	ENSCING00000002567		Cinscaffold_36	900472
3115	ENSG00000136854	STXBP1	Hsa9	129414365	ENSCING00000002400		Cinscaffold_273	42490
3115	ENSG00000076944	STXBP2	Hsa19	7608010	ENSCING00000002400		Cinscaffold_273	42490
3115	ENSG00000116266	STXBP3	Hsa1	109090831	ENSCING00000002400		Cinscaffold_273	42490
3115	ENSG00000136631	VPS45A	Hsa1	148305993	ENSCING00000002400		Cinscaffold_273	42490
3116	ENSG00000135823	STX6	Hsa1	179179798	ENSCING00000002425		Cinscaffold_273	69723
3116	ENSG00000104915	STX10	Hsa19	13115903	ENSCING00000002425		Cinscaffold_273	69723
3116	ENSG00000170310	STX8	Hsa17	9094514	ENSCING00000002425		Cinscaffold_273	69723
3125	ENSG00000115204	MPV17	Hsa2	27385864	ENSCING00000015049		Cinscaffold_26	987673
3125	ENSG00000176876	PXMP2	Hsa12	131774265	ENSCING00000015049		Cinscaffold_26	987673
3125	ENSG00000127226	Q567V2_HUMAN	Hsa19	18165092	ENSCING00000015049		Cinscaffold_26	987673

3130	ENSG00000130816	DNMT1	Hsa19	10105023	ENSCING00000005243		Cinscaffold_26	1053528
3183	ENSG00000180549	FUT7	Hsa9	139044447	ENSCING00000001142		Cinscaffold_137	74872
3183	ENSG00000171124	FUT3	Hsa19	5793902	ENSCING00000001142		Cinscaffold_137	74872
3183	ENSG00000130383	FUT5	Hsa19	5817612	ENSCING00000001142		Cinscaffold_137	74872
3183	ENSG00000172461	FUT9	Hsa6	96570581	ENSCING00000001142		Cinscaffold_137	74872
3199	ENSG00000055732	MCOLN3	Hsa1	85256353	ENSCING00000006356		Cin8q	3601902
3199	ENSG00000153898	MCOLN2	Hsa1	85163855	ENSCING00000006356		Cin8q	3601902
3199	ENSG00000090674	MCOLN1	Hsa19	7493514	ENSCING00000006356		Cin8q	3601902
3214	ENSG00000162415	ZSWIM5	Hsa1	45254658	ENSCING00000002202		Cin8q	2696870
3214	ENSG00000130449	ZSWIM6	Hsa5	60852928	ENSCING00000002202		Cin8q	2696870
3214	ENSG00000132003	ZSWIM4	Hsa19	13767274	ENSCING00000002202		Cin8q	2696870
3218	ENSG00000071626	DAZAP1	Hsa19	1358584	ENSCING00000003362	NP_001027721	Cin8q	2936623
3218	ENSG00000153944	MSI2	Hsa17	52688930	ENSCING00000003362	NP_001027721	Cin8q	2936623
3218	ENSG00000135097	MSI1	Hsa12	119263516	ENSCING00000003362	NP_001027721	Cin8q	2936623
3227	ENSG00000130175	PRKCSH	Hsa19	11407269	ENSCING00000007367		Cin8q	4741895
3230	ENSG00000172081	MOBKL2A	Hsa19	2022037	ENSCING00000001982		Cin8q	6624685
3230	ENSG00000120162	MOBKL2B	Hsa9	27315207	ENSCING00000001982		Cin8q	6624685
3230	ENSG00000142961	MOBKL2C	Hsa1	46845978	ENSCING00000001982		Cin8q	6624685
3262	ENSG00000058799	YIPF1	Hsa1	54089980	ENSCING00000007210		Cin8q	4546523
3262	ENSG00000130733	YIPF2	Hsa19	10894446	ENSCING00000007210		Cin8q	4546523
3277	ENSG00000105607	GCDH	Hsa19	12862974	ENSCING00000006483		Cin8q	3928919
3280	ENSG00000105640	RPL18A	Hsa19	17831731	ENSCING00000013142		Cin8q	5731696
3280	ENSG00000188096	285053	Hsa2	47771359	ENSCING00000013142		Cin8q	5731696
3280	ENSG00000186374	127545	Hsa1	33218133	ENSCING00000013142		Cin8q	5731696
3282	ENSG00000129932	DOHH	Hsa19	3441824	ENSCING00000008362		Cin8q	6294491
3285	ENSG00000130734	ATG4D	Hsa19	10515593	ENSCING00000003328		Cin8q	3126805
3285	ENSG00000125703	ATG4C	Hsa1	63022394	ENSCING00000003328		Cin8q	3126805
3312	ENSG00000089639	GMIP	Hsa19	19601285	ENSCING00000013155		Cin8q	5512474
3312	ENSG00000180448	HMHA1	Hsa19	1018174	ENSCING00000013155		Cin8q	5512474
3312	ENSG00000137962	ARHGAP29	Hsa1	94409895	ENSCING00000013155		Cin8q	5512474
3312	ENSG00000089639	GMIP	Hsa19	19601285	ENSCING00000013155		Cin8q	5512474
3312	ENSG00000180448	HMHA1	Hsa19	1018174	ENSCING00000013155		Cin8q	5512474
3312	ENSG00000137962	ARHGAP29	Hsa1	94409895	ENSCING00000013155		Cin8q	5512474
3325	ENSG00000117450	PRDX1	Hsa1	45749295	ENSCING00000003002	NP_001027810	Cin8q	7427039
3325	ENSG00000167815	PRDX2	Hsa19	12768635	ENSCING00000003002	NP_001027810	Cin8q	7427039
3325	ENSG00000123131	PRDX4	HsaX	23592300	ENSCING00000003002	NP_001027810	Cin8q	7427039
3325	ENSG00000165672	PRDX3	Hsa10	120917205	ENSCING00000003002	NP_001027810	Cin8q	7427039
3330	ENSG00000150471	LPHN3	Hsa4	62045434	ENSCING00000002093		Cin8q	2383308
3330	ENSG00000072071	LPHN1	Hsa19	14119547	ENSCING00000002093		Cin8q	2383308
3331	ENSG00000105325	FZR1	Hsa19	3457368	ENSCING00000001048		Cin8q	44579
3345	ENSG00000085872	CHERP	Hsa19	16489710	ENSCING00000004814		Cin8q	642623
3350	ENSG00000105072	C19orf44	Hsa19	16468224	ENSCING00000002200		Cin8q	2718102
3355	ENSG00000072958	AP1M1	Hsa19	16169731	ENSCING00000006771		Cin8q	2047477
3355	ENSG00000129354	AP1M2	Hsa19	10544348	ENSCING00000006771		Cin8q	2047477
3355	ENSG00000185009	AP3M1	Hsa10	75551523	ENSCING00000006771		Cin8q	2047477
3355	ENSG00000070718	AP3M2	Hsa8	42129748	ENSCING00000006771		Cin8q	2047477
3355	ENSG00000072958	AP1M1	Hsa19	16169731	ENSCING00000006771		Cin8q	2047477
3355	ENSG00000129354	AP1M2	Hsa19	10544348	ENSCING00000006771		Cin8q	2047477
3355	ENSG00000185009	AP3M1	Hsa10	75551523	ENSCING00000006771		Cin8q	2047477
3355	ENSG00000070718	AP3M2	Hsa8	42129748	ENSCING00000006771		Cin8q	2047477
3362	ENSG00000129347	NP_075384.2	Hsa19	10524761	ENSCING00000004801		Cin8q	733559
3377	ENSG00000174348	PODN	Hsa1	53300442	ENSCING00000004487		Cin8q	2467710
3377	ENSG00000132000	PODNL1	Hsa19	13904167	ENSCING00000004487		Cin8q	2467710
3377	ENSG00000172061	LRRC15	Hsa3	195557274	ENSCING00000004487		Cin8q	2467710
3377	ENSG00000188783	PRELP	Hsa1	201711506	ENSCING00000004487		Cin8q	2467710
3377	ENSG00000125848	FLRT3	Hsa20	14251634	ENSCING00000004487		Cin8q	2467710
3383	ENSG00000118046	STK11	Hsa19	1156798	ENSCING00000006359		Cin8q	3609834
3391	ENSG00000105401	CDC37	Hsa19	10362809	ENSCING00000006469		Cin8q	3881188
3391	ENSG00000106993	CDC37L1	Hsa9	4669559	ENSCING00000006469		Cin8q	3881188
3404	ENSG00000107077	JMJD2C	Hsa9	6747656	ENSCING00000001998		Cin8q	6474743
3404	ENSG00000066135	JMJD2A	Hsa1	43888416	ENSCING00000001998		Cin8q	6474743
3404	ENSG00000127663	JMJD2B	Hsa19	4920132	ENSCING00000001998		Cin8q	6474743
3409	ENSG00000104980	TIMM44	Hsa19	7897604	ENSCING00000002188		Cin8q	6731033
3411	ENSG00000079277	MKNK1	Hsa1	46795677	ENSCING00000002198		Cin8q	2731330
3411	ENSG00000099875	MKNK2	Hsa19	1988481	ENSCING00000002198		Cin8q	2731330
3420	ENSG00000160013	PTGIR	Hsa19	51815566	ENSCING00000013274		Cin8q	2694171
3420	ENSG00000171522	PTGER4	Hsa5	40715789	ENSCING00000013274		Cin8q	2694171
3420	ENSG00000125384	PTGER2	Hsa14	51850863	ENSCING00000013274		Cin8q	2694171
3420	ENSG00000050628	PTGER3	Hsa1	71090624	ENSCING00000013274		Cin8q	2694171
3420	ENSG00000168229	PTGDR	Hsa14	51804181	ENSCING00000013274		Cin8q	2694171
3420	ENSG00000122420	PTGFR	Hsa1	78542156	ENSCING00000013274		Cin8q	2694171
3420	ENSG00000006638	TBXA2R	Hsa19	3545778	ENSCING00000013274		Cin8q	2694171
3420	ENSG00000160951	PTGER1	Hsa19	14444279	ENSCING00000013274		Cin8q	2694171
3420	ENSG00000160013	PTGIR	Hsa19	51815566	ENSCING00000013274		Cin8q	2694171
3420	ENSG00000171522	PTGER4	Hsa5	40715789	ENSCING00000013274		Cin8q	2694171
3420	ENSG00000125384	PTGER2	Hsa14	51850863	ENSCING00000013274		Cin8q	2694171
3420	ENSG00000050628	PTGER3	Hsa1	71090624	ENSCING00000013274		Cin8q	2694171
3420	ENSG00000168229	PTGDR	Hsa14	51804181	ENSCING00000013274		Cin8q	2694171
3420	ENSG00000122420	PTGFR	Hsa1	78542156	ENSCING00000013274		Cin8q	2694171
3420	ENSG00000006638	TBXA2R	Hsa19	3545778	ENSCING00000013274		Cin8q	2694171
3420	ENSG00000160951	PTGER1	Hsa19	14444279	ENSCING00000013274		Cin8q	2694171
3426	ENSG00000105583	C19orf56	Hsa19	12639885	ENSCING00000007518		Cin8q	5326872
3427	ENSG00000104957	CCDC130	Hsa19	13703574	ENSCING00000006870		Cin8q	5404317
3428	ENSG00000167774	NDUFA7	Hsa19	8282234	ENSCING00000013360		Cin8q	327555
3435	ENSG00000102575	ACP5	Hsa19	11546506	ENSCING00000007515		Cin8q	5263945
3442	ENSG00000187650	NP_001017921	Hsa19	5855885	ENSCING00000005679		Cin8q	5500176

3445	ENSG00000179262	RAD23A	Hsa19	12917654	ENSCING00000008350		Cin8q	274721
3464	ENSG00000049192	ADAMTS6	Hsa5	64480322	ENSCING00000007274		Cin8q	4313195
3464	ENSG00000142303	ADAMTS10	Hsa19	8551126	ENSCING00000007274		Cin8q	4313195
3464	ENSG00000145808	ADAMTS19	Hsa5	128824002	ENSCING00000007274		Cin8q	4313195
3464	ENSG00000156140	ADAMTS3	Hsa4	73365551	ENSCING00000007274		Cin8q	4313195
3464	ENSG00000087116	ADAMTS2	Hsa5	178473474	ENSCING00000007274		Cin8q	4313195
3464	ENSG00000138316	ADAMTS14	Hsa10	72102565	ENSCING00000007274		Cin8q	4313195
3483	ENSG00000130810	PPAN	Hsa19	10077965	ENSCING00000005420		Cin8q	3411466
3487	ENSG00000104897	SF3A2	Hsa19	2187816	ENSCING00000003319		Cin8q	3122070
3500	ENSG00000145675	PIK3R1	Hsa5	67547360	ENSCING00000002107		Cin8q	2439772
3500	ENSG00000117461	PIK3R3	Hsa1	46278399	ENSCING00000002107		Cin8q	2439772
3500	ENSG00000105647	PIK3R2	Hsa19	18125016	ENSCING00000002107		Cin8q	2439772
3503	ENSG00000157107	FCHO2	Hsa5	72287650	ENSCING00000006462		Cin8q	3856684
3503	ENSG00000118473	SGIP1	Hsa1	66771654	ENSCING00000006462		Cin8q	3856684
3503	ENSG00000130475	FCHO1	Hsa19	17726920	ENSCING00000006462		Cin8q	3856684
3509	ENSG00000079999	KEAP1	Hsa19	10457802	ENSCING00000008356		Cin8q	6208323
3512	ENSG00000185551	NR2F2	Hsa15	94674950	ENSCING00000002023	Q4H3S1_CIOIN	Cin8q	7452513
3512	ENSG00000175745	NR2F1	Hsa5	92944799	ENSCING00000002023	Q4H3S1_CIOIN	Cin8q	7452513
3512	ENSG00000160113	NR2F6	Hsa19	17203694	ENSCING00000002023	Q4H3S1_CIOIN	Cin8q	7452513
3524	ENSG00000072062	PRKACA	Hsa19	14063509	ENSCING00000006522		Cin8q	3989626
3524	ENSG00000142875	PRKACB	Hsa1	84316329	ENSCING00000006522		Cin8q	3989626
3524	ENSG00000165059	PRKACG	Hsa9	70817241	ENSCING00000006522		Cin8q	3989626
3524	ENSG00000183943	PRKX	HsaX	3532411	ENSCING00000006522		Cin8q	3989626
3524	ENSG00000099725	PRKY	HsaY	7202013	ENSCING00000006522		Cin8q	3989626
3528	ENSG00000165023	DIRAS2	Hsa9	92411934	ENSCING00000007348		Cin8q	4143153
3528	ENSG00000176490	DIRAS1	Hsa19	2665566	ENSCING00000007348		Cin8q	4143153
3528	ENSG00000162595	DIRAS3	Hsa1	68284233	ENSCING00000007348		Cin8q	4143153
3528	ENSG00000100302	RASD2	Hsa22	34267296	ENSCING00000007348		Cin8q	4143153
3535	ENSG00000130520	LSM4	Hsa19	18278720	ENSCING00000007519		Cin8q	5328210
3535	ENSG00000100028	SNRPD3	Hsa22	23281618	ENSCING00000007519		Cin8q	5328210
3535	ENSG00000167088	SNRPD1	Hsa18	17446235	ENSCING00000007519		Cin8q	5328210
3535	ENSG00000204392	LSM2	Hsa6	31873152	ENSCING00000007519		Cin8q	5328210
3535	ENSG00000172850	LSM2_HUMAN	Hsac6_QBL	31898277	ENSCING00000007519		Cin8q	5328210
3535	ENSG00000111987	LSM2_HUMAN	Hsac6_COX	31899794	ENSCING00000007519		Cin8q	5328210
3535	ENSG00000181817	LSM10	Hsa1	36631618	ENSCING00000007519		Cin8q	5328210
3540	ENSG00000167470	MIDN	Hsa19	1199552	ENSCING00000013146		Cin8q	5686319
3544	ENSG00000125656	CLPP	Hsa19	6312463	ENSCING00000004492		Cin8q	2464117
3551	ENSG00000172009	THOP1	Hsa19	2736506	ENSCING00000008373		Cin8q	346275
3551	ENSG00000123213	NLN	Hsa5	65053779	ENSCING00000008373		Cin8q	346275
3618	ENSG00000145780	FEM1C	Hsa5	114884507	ENSCING00000006394		Cin10p	598456
3618	ENSG00000141965	FEM1A	Hsa19	4742770	ENSCING00000006394		Cin10p	598456
3627	ENSG00000007080	CCDC124	Hsa19	17908217	ENSCING00000001917		Cinscaffold_176	46632
3670	ENSG00000129353	SLC44A2	Hsa19	10574186	ENSCING00000005630		Cin9p	2821383
3670	ENSG00000204385	SLC44A4	Hsa6	31938946	ENSCING00000005630		Cin9p	2821383
3670	ENSG00000206269	CTL4_HUMAN	Hsac6_COX	31965993	ENSCING00000005630		Cin9p	2821383
3670	ENSG00000206378	CTL4_HUMAN	Hsac6_QBL	31964443	ENSCING00000005630		Cin9p	2821383
3670	ENSG00000137968	SLC44A5	Hsa1	75440404	ENSCING00000005630		Cin9p	2821383
3673	ENSG00000105726	ATP13A1	Hsa19	19617009	ENSCING00000005525		Cin9p	2579957
3674	ENSG00000127220	ABHD8	Hsa19	17263941	ENSCING00000006639		Cin9p	1583596
3677	ENSG00000080511	RDH8	Hsa19	9984925	ENSCING00000005577		Cin9p	2783378
3697	ENSG00000160293	VAV2	Hsa9	135616837	ENSCING00000008711		Cin9p	1304396
3697	ENSG00000141968	VAV1	Hsa19	6723722	ENSCING00000008711		Cin9p	1304396
3697	ENSG00000134215	VAV3	Hsa1	107915305	ENSCING00000008711		Cin9p	1304396
3698	ENSG00000076984	O14733-2	Hsa19	7874728	ENSCING00000002925	Q4H379_CIOIN	Cin9p	3790241
3704	ENSG00000171236	LRG1	Hsa19	4479439	ENSCING00000014175		Cin9p	2518529
3734	ENSG00000011454	RABGAP1	Hsa9	124743109	ENSCING00000003244		Cin9p	2257255
3734	ENSG00000152061	RABGAP1L	Hsa1	172395171	ENSCING00000003244		Cin9p	2257255
3734	ENSG00000142459	EVI5L	Hsa19	7801243	ENSCING00000003244		Cin9p	2257255
3734	ENSG00000067208	EVI5	Hsa1	92746841	ENSCING00000003244		Cin9p	2257255
3750	ENSG00000160117	ANKRD41	Hsa19	17253681	ENSCING00000005552		Cin9p	2692072
3762	ENSG00000095059	DHPS	Hsa19	12647565	ENSCING00000003390		Cin9p	3229827
3768	ENSG00000196814	FAM125B	Hsa9	128128949	ENSCING00000005543		Cin9p	2651268
3768	ENSG00000141971	FAM125A	Hsa19	17391853	ENSCING00000005543		Cin9p	2651268
3784	ENSG00000148339	SLC25A25	Hsa9	129870300	ENSCING00000003221		Cin9p	2183483
3784	ENSG00000085491	SLC25A24	Hsa1	108478965	ENSCING00000003221		Cin9p	2183483
3784	ENSG00000125648	SLC25A23	Hsa19	6389747	ENSCING00000003221		Cin9p	2183483
3784	ENSG00000181240	NP_775908.1	Hsa19	6377050	ENSCING00000003221		Cin9p	2183483
3795	ENSG00000008382	NP_116257.2	Hsa19	4294551	ENSCING00000006883		Cin9p	562297
3837	ENSG00000084733	RAB10	Hsa2	26110483	ENSCING00000003959		Cinscaffold_92	184881
3837	ENSG00000166128	RAB8B	Hsa15	61268781	ENSCING00000003959		Cinscaffold_92	184881
3837	ENSG00000167461	RAB8A	Hsa19	16083467	ENSCING00000003959		Cinscaffold_92	184881
3837	ENSG00000143545	RAB13	Hsa1	152220751	ENSCING00000003959		Cinscaffold_92	184881
3837	ENSG00000205319	649713	Hsa12	54660473	ENSCING00000003959		Cinscaffold_92	184881
3837	ENSG00000138069	RAB1A	Hsa2	65167493	ENSCING00000003959		Cinscaffold_92	184881
3837	ENSG00000172794	RAB37	Hsa17	70178312	ENSCING00000003959		Cinscaffold_92	184881
3837	ENSG00000152932	RAB3C	Hsa5	57914671	ENSCING00000003959		Cinscaffold_92	184881
3837	ENSG00000167964	RAB26	Hsa16	2138646	ENSCING00000003959		Cinscaffold_92	184881
3837	ENSG00000105649	RAB3A	Hsa19	18168611	ENSCING00000003959		Cinscaffold_92	184881
3837	ENSG00000105514	RAB3D	Hsa19	11296094	ENSCING00000003959		Cinscaffold_92	184881
3837	ENSG00000169213	RAB3B	Hsa1	52146216	ENSCING00000003959		Cinscaffold_92	184881
3837	ENSG00000084733	RAB10	Hsa2	26110483	ENSCING00000003959		Cinscaffold_92	184881
3837	ENSG00000166128	RAB8B	Hsa15	61268781	ENSCING00000003959		Cinscaffold_92	184881
3837	ENSG00000167461	RAB8A	Hsa19	16083467	ENSCING00000003959		Cinscaffold_92	184881
3837	ENSG00000143545	RAB13	Hsa1	152220751	ENSCING00000003959		Cinscaffold_92	184881
3837	ENSG00000205319	649713	Hsa12	54660473	ENSCING00000003959		Cinscaffold_92	184881
3837	ENSG00000138069	RAB1A	Hsa2	65167493	ENSCING00000003959		Cinscaffold_92	184881

3837	ENSG00000172794	RAB37	Hsa17	70178312	ENSCING00000003959		Cinscaffold_92	184881
3837	ENSG00000152932	RAB3C	Hsa5	57914671	ENSCING00000003959		Cinscaffold_92	184881
3837	ENSG00000167964	RAB26	Hsa16	2138646	ENSCING00000003959		Cinscaffold_92	184881
3837	ENSG00000105649	RAB3A	Hsa19	18168611	ENSCING00000003959		Cinscaffold_92	184881
3837	ENSG00000105514	RAB3D	Hsa19	11296094	ENSCING00000003959		Cinscaffold_92	184881
3837	ENSG00000169213	RAB3B	Hsa1	52146216	ENSCING00000003959		Cinscaffold_92	184881
3840	ENSG00000177076	ASAH3L	Hsa9	19398925	ENSCING00000000068		Cinscaffold_83	462706
3840	ENSG00000180105		Hsa2	206983053	ENSCING00000000068		Cinscaffold_83	462706
3840	ENSG00000167769	ASAH3	Hsa19	6257725	ENSCING00000000068		Cinscaffold_83	462706
3840	ENSG00000078124	PHCA	Hsa11	76249601	ENSCING00000000068		Cinscaffold_83	462706
3860	ENSG00000065000	AP3D1	Hsa19	2051993	ENSCING00000002744		Cin5q	2168993
3864	ENSG00000130522	JUND	Hsa19	18252251	ENSCING00000009285		Cin5q	3989566
3864	ENSG00000171223	JUNB	Hsa19	12763286	ENSCING00000009285		Cin5q	3989566
3864	ENSG00000130522	JUND	Hsa19	18252251	ENSCING00000009285		Cin5q	3989566
3864	ENSG00000171223	JUNB	Hsa19	12763286	ENSCING00000009285		Cin5q	3989566
3870	ENSG00000080298	RFX3	Hsa9	3208297	ENSCING00000009186	Q4H2V6_CIOIN	Cin5q	5499071
3870	ENSG00000132005	RFX1	Hsa19	13933353	ENSCING00000009186	Q4H2V6_CIOIN	Cin5q	5499071
3870	ENSG00000087903	RFX2	Hsa19	5944175	ENSCING00000009186	Q4H2V6_CIOIN	Cin5q	5499071
3870	ENSG00000080298	RFX3	Hsa9	3208297	ENSCING00000009186	Q4H2V6_CIOIN	Cin5q	5499071
3870	ENSG00000132005	RFX1	Hsa19	13933353	ENSCING00000009186	Q4H2V6_CIOIN	Cin5q	5499071
3870	ENSG00000087903	RFX2	Hsa19	5944175	ENSCING00000009186	Q4H2V6_CIOIN	Cin5q	5499071
3884	ENSG00000099624	ATP5D	Hsa19	1192749	ENSCING00000012171		Cin5q	4365869
3889	ENSG00000105011	ASF1B	Hsa19	14091322	ENSCING00000000181		Cin5q	1813452
3889	ENSG00000111875	ASF1A	Hsa6	119256901	ENSCING00000000181		Cin5q	1813452
3908	ENSG00000080503	SMARCA2	Hsa9	2005342	ENSCING00000009221		Cin5q	5889795
3908	ENSG00000127616	SMARCA4	Hsa19	10932606	ENSCING00000009221		Cin5q	5889795
3921	ENSG00000179218	CALR	Hsa19	12910392	ENSCING00000008071		Cin5q	4241606
3921	ENSG00000141979	CALR3	Hsa19	16450888	ENSCING00000008071		Cin5q	4241606
3921	ENSG00000179218	CALR	Hsa19	12910392	ENSCING00000008071		Cin5q	4241606
3921	ENSG00000141979	CALR3	Hsa19	16450888	ENSCING00000008071		Cin5q	4241606
3925	ENSG00000162599	NFIA	Hsa1	61103519	ENSCING00000002528	Q4H353_CIOIN	Cin5q	2570575
3925	ENSG00000008441	NFIX	Hsa19	12967584	ENSCING00000002528	Q4H353_CIOIN	Cin5q	2570575
3925	ENSG00000147862	NFIB	Hsa9	14071847	ENSCING00000002528	Q4H353_CIOIN	Cin5q	2570575
3925	ENSG00000141905	NFIC	Hsa19	3310616	ENSCING00000002528	Q4H353_CIOIN	Cin5q	2570575
3925	ENSG00000162599	NFIA	Hsa1	61103519	ENSCING00000002528	Q4H353_CIOIN	Cin5q	2570575
3925	ENSG00000008441	NFIX	Hsa19	12967584	ENSCING00000002528	Q4H353_CIOIN	Cin5q	2570575
3925	ENSG00000147862	NFIB	Hsa9	14071847	ENSCING00000002528	Q4H353_CIOIN	Cin5q	2570575
3925	ENSG00000141905	NFIC	Hsa19	3310616	ENSCING00000002528	Q4H353_CIOIN	Cin5q	2570575
3928	ENSG00000181029	TRAPPC5	Hsa19	7651761	ENSCING00000008070		Cin5q	4234554
3934	ENSG00000106688	SLC1A1	Hsa9	4480444	ENSCING00000007497		Cin5q	420206
3934	ENSG00000079215	SLC1A3	Hsa5	36642446	ENSCING00000007497		Cin5q	420206
3934	ENSG00000105143	SLC1A6	Hsa19	14921991	ENSCING00000007497		Cin5q	420206
3934	ENSG00000110436	SLC1A2	Hsa11	35229329	ENSCING00000007497		Cin5q	420206
3934	ENSG00000162383	SLC1A7	Hsa1	53325443	ENSCING00000007497		Cin5q	420206
3934	ENSG00000105281	SLC1A5	Hsa19	51969982	ENSCING00000007497		Cin5q	420206
3939	ENSG00000127527	EPS15L1	Hsa19	16333408	ENSCING00000009283		Cin5q	4024555
3948	ENSG00000031823	RANBP3	Hsa19	5868120	ENSCING00000007536		Cin5q	314935
3961	ENSG00000127526	SLC35E1	Hsa19	16522666	ENSCING00000007485		Cin5q	503311
3961	ENSG00000189339	SLC35E2	Hsa1	1582802	ENSCING00000007485		Cin5q	503311
3969	ENSG00000105518	NP_940938.1	Hsa19	11314457	ENSCING00000012290		Cin5q	1046465
3973	ENSG00000198642	KLHL9	Hsa9	21319670	ENSCING00000002534		Cin5q	2583806
3973	ENSG00000003096	KLHL13	HsaX	116915804	ENSCING00000002534		Cin5q	2583806
3973	ENSG00000167487	KLHL26	Hsa19	18608838	ENSCING00000002534		Cin5q	2583806
3973	ENSG00000135686	NP_079007.2	Hsa16	83239632	ENSCING00000002534		Cin5q	2583806
3973	ENSG00000124743	KBTBD1	Hsa6	53624355	ENSCING00000002534		Cin5q	2583806
3973	ENSG00000197705	KLHL14	Hsa18	28506632	ENSCING00000002534		Cin5q	2583806
3973	ENSG00000186231	KIAA1900	Hsa6	97479326	ENSCING00000002534		Cin5q	2583806
3973	ENSG00000185214	KLHL22	Hsa22	19125806	ENSCING00000002534		Cin5q	2583806
3973	ENSG00000174010	KLHL15	HsaX	23911758	ENSCING00000002534		Cin5q	2583806
3977	ENSG00000086015	MAST2	Hsa1	46041872	ENSCING00000009247		Cin5q	6169430
3977	ENSG00000105613	MAST1	Hsa19	12810348	ENSCING00000009247		Cin5q	6169430
3977	ENSG00000099308	MAST3	Hsa19	18069605	ENSCING00000009247		Cin5q	6169430
3977	ENSG00000086015	MAST2	Hsa1	46041872	ENSCING00000009247		Cin5q	6169430
3977	ENSG00000105613	MAST1	Hsa19	12810348	ENSCING00000009247		Cin5q	6169430
3977	ENSG00000099308	MAST3	Hsa19	18069605	ENSCING00000009247		Cin5q	6169430
3982	ENSG00000117519	CNN3	Hsa1	95135095	ENSCING00000007496		Cin5q	430113
3982	ENSG00000130176	CNN1	Hsa19	11510579	ENSCING00000007496		Cin5q	430113
3982	ENSG00000064666	CNN2	Hsa19	977298	ENSCING00000007496		Cin5q	430113
3982	ENSG00000204782	XR_016647.1	Hsa9	68788495	ENSCING00000007496		Cin5q	430113
3982	ENSG00000204826	XR_018140.1	Hsa9	42999838	ENSCING00000007496		Cin5q	430113
3982	ENSG00000204718		Hsa2	94766764	ENSCING00000007496		Cin5q	430113
3982	ENSG00000117519	CNN3	Hsa1	95135095	ENSCING00000007496		Cin5q	430113
3982	ENSG00000130176	CNN1	Hsa19	11510579	ENSCING00000007496		Cin5q	430113
3982	ENSG00000064666	CNN2	Hsa19	977298	ENSCING00000007496		Cin5q	430113
3982	ENSG00000204782	XR_016647.1	Hsa9	68788495	ENSCING00000007496		Cin5q	430113
3982	ENSG00000204826	XR_018140.1	Hsa9	42999838	ENSCING00000007496		Cin5q	430113
3982	ENSG00000204718		Hsa2	94766764	ENSCING00000007496		Cin5q	430113
3983	ENSG00000197860	SGTB	Hsa5	64997511	ENSCING00000001481		Cin5q	1580114
3983	ENSG00000104969	SGTA	Hsa19	2705712	ENSCING00000001481		Cin5q	1580114
4002	ENSG00000184588	PDE4B	Hsa1	66030785	ENSCING00000007423		Cin5q	588343
4002	ENSG00000113448	PDE4D	Hsa5	58305622	ENSCING00000007423		Cin5q	588343
4002	ENSG00000105650	PDE4C	Hsa19	18182010	ENSCING00000007423		Cin5q	588343
4002	ENSG00000065989	PDE4A	Hsa19	10388449	ENSCING00000007423		Cin5q	588343
4002	ENSG00000171408	PDE7B	Hsa6	136214527	ENSCING00000007423		Cin5q	588343
4002	ENSG00000205268	PDE7A	Hsa8	66793867	ENSCING00000007423		Cin5q	588343
4002	ENSG00000184588	PDE4B	Hsa1	66030785	ENSCING00000007423		Cin5q	588343

4002	ENSG00000113448	PDE4D	Hsa5	58305622	ENSCING00000007423		Cin5q	588343
4002	ENSG00000105650	PDE4C	Hsa19	18182010	ENSCING00000007423		Cin5q	588343
4002	ENSG00000065989	PDE4A	Hsa19	10388449	ENSCING00000007423		Cin5q	588343
4002	ENSG00000171408	PDE7B	Hsa6	136214527	ENSCING00000007423		Cin5q	588343
4002	ENSG00000205268	PDE7A	Hsa8	66793867	ENSCING00000007423		Cin5q	588343
4005	ENSG00000127452	FBXL12	Hsa19	9781948	ENSCING00000009233		Cin5q	6005633
4009	ENSG00000179271	GADD45GIP1	Hsa19	12925972	ENSCING00000012179		Cin5q	4200898
4010	ENSG00000181781	C19orf19	Hsa19	414360	ENSCING00000006307		Cin5q	1423057
4010	ENSG00000182950	ODF3L1	Hsa15	73803374	ENSCING00000006307		Cin5q	1423057
4021	ENSG00000083312	TNPO1	Hsa5	72148171	ENSCING00000008100		Cin5q	4421746
4021	ENSG00000105576	TNPO2	Hsa19	12672967	ENSCING00000008100		Cin5q	4421746
4026	ENSG00000123159	GIPC1	Hsa19	14449572	ENSCING00000009648		Cin5q	825978
4026	ENSG00000137960	GIPC2	Hsa1	78284174	ENSCING00000009648		Cin5q	825978
4026	ENSG00000179855	GIPC3	Hsa19	3536569	ENSCING00000009648		Cin5q	825978
4026	ENSG00000123159	GIPC1	Hsa19	14449572	ENSCING00000009648		Cin5q	825978
4026	ENSG00000137960	GIPC2	Hsa1	78284174	ENSCING00000009648		Cin5q	825978
4026	ENSG00000179855	GIPC3	Hsa19	3536569	ENSCING00000009648		Cin5q	825978
4029	ENSG00000130165	ELOF1	Hsa19	11524861	ENSCING00000000493		Cin5q	2328108
4031	ENSG00000125652	ALKBH7	Hsa19	6323444	ENSCING00000003788		Cin5q	1293227
4047	ENSG00000105426	PTPRS	Hsa19	5157379	ENSCING00000007442		Cin5q	543234
4047	ENSG00000142949	PTPRF	Hsa1	43769134	ENSCING00000007442		Cin5q	543234
4047	ENSG00000153707	PTPRD	Hsa9	8304246	ENSCING00000007442		Cin5q	543234
4059	ENSG00000099331	MYO9B	Hsa19	17073466	ENSCING00000009225		Cin5q	5921554
4059	ENSG00000066933	MYO9A	Hsa15	69905975	ENSCING00000009225		Cin5q	5921554
4075	ENSG00000142453	CARM1	Hsa19	10843253	ENSCING00000008138		Cin5q	4904612
4086	ENSG00000167658	EEF2	Hsa19	3927055	ENSCING00000009356		Cin5q	2519682
4103	ENSG00000080709	KCNN2	Hsa5	113725565	ENSCING00000009747		Cinscaffold_44	615987
4103	ENSG00000143603	KCNN3	Hsa1	152946526	ENSCING00000009747		Cinscaffold_44	615987
4103	ENSG00000105642	KCNN1	Hsa19	17945647	ENSCING00000009747		Cinscaffold_44	615987
4103	ENSG00000104783	KCNN4	Hsa19	48962578	ENSCING00000009747		Cinscaffold_44	615987
4115	ENSG00000169032	MAP2K1	Hsa15	64466674	ENSCING00000004105	Q4H383_CIOIN	Cinscaffold_44	304913
4115	ENSG00000126934	MAP2K2	Hsa19	4041331	ENSCING00000004105	Q4H383_CIOIN	Cinscaffold_44	304913
4115	ENSG00000137764	MAP2K5	Hsa15	65622075	ENSCING00000004105	Q4H383_CIOIN	Cinscaffold_44	304913
4234	ENSG00000182490	TSSK2	Hsa22	17497793	ENSCING00000012478		Cin3q	4772341
4234	ENSG00000162526	TSSK3	Hsa1	32600385	ENSCING00000012478		Cin3q	4772341
4234	ENSG00000178093	TSSK6	Hsa19	19484230	ENSCING00000012478		Cin3q	4772341
4234	ENSG00000139908	TSSK4	Hsa14	23744766	ENSCING00000012478		Cin3q	4772341
4241	ENSG00000138829	FBN2	Hsa5	127621500	ENSCING00000008571		Cin3q	3293995
4241	ENSG00000166147	FBN1	Hsa15	46489479	ENSCING00000008571		Cin3q	3293995
4241	ENSG00000142449	FBN3	Hsa19	8036287	ENSCING00000008571		Cin3q	3293995
4262	ENSG00000104883	PEX11G	Hsa19	7447761	ENSCING00000012654		Cin3q	2883803
4262	ENSG00000131779	PEX11B	Hsa1	144227609	ENSCING00000012654		Cin3q	2883803
4277	ENSG00000152611	CAPSL	Hsa5	35940228	ENSCING00000009506		Cin3q	878550
4277	ENSG00000105519	CAPS	Hsa19	5862386	ENSCING00000009506		Cin3q	878550
4313	ENSG00000107341	UBE2R2	Hsa9	33807182	ENSCING00000008583		Cin3q	3271890
4313	ENSG00000099804	CDC34	Hsa19	482733	ENSCING00000008583		Cin3q	3271890
4328	ENSG00000107242	PIP5K1B	Hsa9	70510436	ENSCING00000009558		Cin3q	1417104
4328	ENSG00000186111	PIP5K1C	Hsa19	3581182	ENSCING00000009558		Cin3q	1417104
4328	ENSG00000143398	PIP5K1A	Hsa1	149437651	ENSCING00000009558		Cin3q	1417104
4328	ENSG00000173780	Q8TBY6_HUMAN	Hsa6	7931335	ENSCING00000009558		Cin3q	1417104
4334	ENSG00000099622	CIRBP	Hsa19	1220336	ENSCING00000012693		Cin3q	2540881
4340	ENSG00000198258	UBL5	Hsa19	9799568	ENSCING00000012811		Cin3q	748429
4342	ENSG00000140262	TCF12	Hsa15	54998125	ENSCING00000009523	Q4H3N7_CIOIN	Cin3q	1071429
4342	ENSG00000196628	TCF4	Hsa18	51046093	ENSCING00000009523	Q4H3N7_CIOIN	Cin3q	1071429
4342	ENSG00000071564	TCF3	Hsa19	1561964	ENSCING00000009523	Q4H3N7_CIOIN	Cin3q	1071429
4366	ENSG00000185236	RAB11B	Hsa19	8361229	ENSCING00000009526		Cin3q	1093338
4366	ENSG00000103769	RAB11A	Hsa15	63948850	ENSCING00000009526		Cin3q	1093338
4366	ENSG00000132698	RAB25	Hsa1	154297575	ENSCING00000009526		Cin3q	1093338
4500	ENSG00000125731	SH2D3A	Hsa19	6703211	ENSCING00000006956		Cin9q	1147693
4513	ENSG00000117500	TMED5	Hsa1	93387887	ENSCING00000012949		Cin9q	2758543
4513	ENSG00000099203	TMED1	Hsa19	10804115	ENSCING00000012949		Cin9q	2758543
4577	ENSG00000171903	CYP4F11	Hsa19	15884181	ENSCING00000012966		Cin9q	2546363
4577	ENSG00000186115	CYP4F2	Hsa19	15849834	ENSCING00000012966		Cin9q	2546363
4577	ENSG00000186529	CYP4F3	Hsa19	15613196	ENSCING00000012966		Cin9q	2546363
4577	ENSG00000186204	CYP4F12	Hsa19	15645340	ENSCING00000012966		Cin9q	2546363
4577	ENSG00000186526	CYP4F8	Hsa19	15587421	ENSCING00000012966		Cin9q	2546363
4577	ENSG00000187048	CYP4A11	Hsa1	47167436	ENSCING00000012966		Cin9q	2546363
4577	ENSG00000162365	CYP4A22	Hsa1	47375433	ENSCING00000012966		Cin9q	2546363
4592	ENSG00000198003	NP_659482.2	Hsa19	11392273	ENSCING00000012955		Cin9q	2716183
4605	ENSG00000171954	NP_775754.1	Hsa19	15497144	ENSCING00000012965		Cin9q	2552893
4605	ENSG00000186160	CYP4Z1	Hsa1	47305634	ENSCING00000012965		Cin9q	2552893
4605	ENSG00000154198	Q8N1L4_HUMAN	Hsa1	47081354	ENSCING00000012965		Cin9q	2552893
4618	ENSG00000134250	NOTCH2	Hsa1	120255699	ENSCING00000003521		Cin9q	2333255
4618	ENSG00000148400	NOTCH1	Hsa9	138508717	ENSCING00000003521		Cin9q	2333255
4618	ENSG00000074181	NOTCH3	Hsa19	15131445	ENSCING00000003521		Cin9q	2333255
4618	ENSG00000204301	GPSM3	Hsa6	32266521	ENSCING00000003521		Cin9q	2333255
4618	ENSG00000112049	Q99466-2	Hsac6_COX	32258349	ENSCING00000003521		Cin9q	2333255
4629	ENSG00000117594	HSD11B1	Hsa1	207926133	ENSCING00000006578		Cin9q	3540834
4629	ENSG00000167733	HSD11B1L	Hsa19	5632035	ENSCING00000006578		Cin9q	3540834
4663	ENSG00000162613	FUBP1	Hsa1	78184755	ENSCING00000009628	Q4H3G6_CIOIN	Cin9q	3200056
4663	ENSG00000088247	KHSRP	Hsa19	6364457	ENSCING00000009628	Q4H3G6_CIOIN	Cin9q	3200056
4663	ENSG00000107164	FUBP3	Hsa9	132444799	ENSCING00000009628	Q4H3G6_CIOIN	Cin9q	3200056
4729	ENSG00000130255	RPL36	Hsa19	5641272	ENSCING00000012007		Cin7q	2184201
4729	ENSG00000186502	347292	Hsa9	110428934	ENSCING00000012007		Cin7q	2184201
4729	ENSG00000130225	Q8WX03_HUMAN	HsaX	114339251	ENSCING00000012007		Cin7q	2184201
4729	ENSG00000169253	643205	Hsa4	77176927	ENSCING00000012007		Cin7q	2184201

4753	ENSG00000180739	EDG8	Hsa19	10484623	ENSCING00000007164	Q69HR2_CIOIN	Cin7q	5133694
4755	ENSG00000076924	XAB2	Hsa19	7590417	ENSCING00000006985		Cin7q	3675996
