# Supplementary material for: Consequences of Lineage-Specific Gene Loss on Functional Evolution of Surviving Paralogs: ALDH1A and Retinoic Acid Signaling in Vertebrate Genomes
Source: PLoS Genet. 2009 May 29;5(5):e1000496. doi: 10.1371/journal.pgen.1000496 (PMC2682703; doi:10.1371/journal.pgen.1000496)
Supplement: Table S2 — Supplementary information for dot-plot on Figure 3A. (0.05 MB PDF) [file pgen.1000496.s004.pdf]

Table S2. Supplementary information for dot-plot on Figure 3A

Genomic distribution of the Mouse orthologos of the Human 10-Mb GN surrounding the human ALDH1A1.								
Genomic region analyzed: human chromosome 9 (69.000 KB - 79.000 KB)								
Group ID	Query Gene ID	Query Ext ID	Query Chromosome	Query Start Base	Outg Gene ID	Outg Ext ID	Outg Chromosome	Outg Start Base
11796	ENSG00000072062	PRKACA	Hsa19	14063509	ENSMUSG00000005469	Prkaca	Mmu8	86863093
11796	ENSG00000165059	PRKACG	Hsa9	70817241	ENSMUSG00000005469	Prkaca	Mmu8	86863093
14275	ENSG00000156017	C9orf41	Hsa9	76785754	ENSMUSG00000024726	2410127L17Ri	Mmu19	18737845
14279	ENSG00000107362	C9orf77	Hsa9	73667188	ENSMUSG00000047368	5730446C15Ri	Mmu19	21720406
14341	ENSG00000106733	C9orf95	Hsa9	76865309	ENSMUSG00000037847	BC016495	Mmu19	18703133
14347	ENSG00000135063	C9orf61	Hsa9	71130168	ENSMUSG00000071604	Gm967	Mmu19	24040525
14369	ENSG00000187210	GCNT1	Hsa9	78263966	ENSMUSG00000038843	Gcnt1	Mmu19	17393162
14370	ENSG00000165092	ALDH1A1	Hsa9	74705408	ENSMUSG00000053279	Aldh1a1	Mmu19	20669078
14375	ENSG00000198963	RORB	Hsa9	76302072	ENSMUSG00000036192	Rorb	Mmu19	19004903
14376	ENSG00000135045	C9orf40	Hsa9	76751317	ENSMUSG00000047044	D030056L22Ri	Mmu19	18780255
14378	ENSG00000083067	TRPM3	Hsa9	72339769	ENSMUSG00000052387	Trpm3	Mmu19	22206216
14379	ENSG00000107282	APBA1	Hsa9	71235022	ENSMUSG00000024897	Apba1	Mmu19	23825973
14381	ENSG00000119125	GDA	Hsa9	73954113	ENSMUSG00000058624	Gda	Mmu19	21370247
14382	ENSG00000099139	PCSK5	Hsa9	77695406	ENSMUSG00000024713	Pcsk5	Mmu19	17500412
14387	ENSG00000107372	ZFAND5	Hsa9	74159194	ENSMUSG00000024750	Za20d2	Mmu19	21339375
14393	ENSG00000198887	SMC5	Hsa9	72063757	ENSMUSG00000024943	Smc5l1	Mmu19	23275820
14397	ENSG00000165091	TMC1	Hsa9	74326537	ENSMUSG00000024749	Tmc1	Mmu19	20852006
14405	ENSG00000188647	PTAR1	Hsa9	71514261	ENSMUSG00000074925		Mmu19	23754554
14450	ENSG00000135002	RFK	Hsa9	78190253	ENSMUSG00000024712	Rfk	Mmu19	17461102
14457	ENSG00000119139	TJP2	Hsa9	70956382	ENSMUSG00000024812	Tjp2	Mmu19	24161608
14533	ENSG00000135046	ANXA1	Hsa9	74956493	ENSMUSG00000024659	Anxa1	Mmu19	20440453
14547	ENSG00000135048	TMEM2	Hsa9	73488102	ENSMUSG00000024754	Tmem2	Mmu19	21845439
14556	ENSG00000165060	FXN	Hsa9	70839995	ENSMUSG00000059363	Fxn	Mmu19	24328550
14563	ENSG00000187866	FAM122A	Hsa9	70584784	ENSMUSG00000074922	2900009I07Ri	Mmu19	24543599
14563	ENSG00000156500	FAM122C	HsaX	133768885	ENSMUSG00000074922	2900009I07Ri	Mmu19	24543599
14580	ENSG00000147996	CBWD1	Hsa9	111039	ENSMUSG00000024878	Cbwd1	Mmu19	24987487
14580	ENSG00000136682	CBWD2	Hsa2	113911853	ENSMUSG00000024878	Cbwd1	Mmu19	24987487
14580	ENSG00000172785	CBWD5	Hsa9	69671821	ENSMUSG00000024878	Cbwd1	Mmu19	24987487
14580	ENSG00000196873	CBWD6	Hsa9	70046217	ENSMUSG00000024878	Cbwd1	Mmu19	24987487
14580	ENSG00000204790	Q4V339_HUMAN	Hsa9	68494355	ENSMUSG00000024878	Cbwd1	Mmu19	24987487
14610	ENSG00000107242	PIP5K1B	Hsa9	70510436	ENSMUSG00000024867	Pip5k1a	Mmu19	24371129
14635	ENSG00000204711	C9orf135	Hsa9	71625529	ENSMUSG00000033053	1700028P14Ri	Mmu19	23625857
14647	ENSG00000154330	PGM5	Hsa9	70161635	ENSMUSG00000041731	Pgm5	Mmu19	24745358
14647	ENSG00000204794	Q5VTQ8_HUMAN	Hsa9	68155593	ENSMUSG00000041731	Pgm5	Mmu19	24745358
14656	ENSG00000134996	OSTF1	Hsa9	76893210	ENSMUSG00000024725	Ostf1	Mmu19	18648289
14664	ENSG00000170122	FOXD4L1	Hsa2	113972712	ENSMUSG00000051490	Foxd4	Mmu19	24966597
14664	ENSG00000187559	FOXD4L3	Hsa9	70107603	ENSMUSG00000051490	Foxd4	Mmu19	24966597
14664	ENSG00000204793	Q4V336_HUMAN	Hsa9	68489300	ENSMUSG00000051490	Foxd4	Mmu19	24966597
14664	ENSG00000204779	XR_016691.1	Hsa9	69465527	ENSMUSG00000051490	Foxd4	Mmu19	24966597
14664	ENSG00000184659	FOXD4L4	Hsa9	69666443	ENSMUSG00000051490	Foxd4	Mmu19	24966597
14664	ENSG00000204828	FX4L4_HUMAN	Hsa9	42707230	ENSMUSG00000051490	Foxd4	Mmu19	24966597
14664	ENSG00000184492	FOXD4	Hsa9	105991	ENSMUSG00000051490	Foxd4	Mmu19	24966597
14673	ENSG00000204612	FOXB2	Hsa9	78824391	ENSMUSG00000056829	Foxb2	Mmu19	16939373
14704	ENSG00000197969	VPS13A	Hsa9	78982181	ENSMUSG00000046230	Vps13a	Mmu19	16683690
14715	ENSG00000155621	C9orf85	Hsa9	73716243	ENSMUSG00000035171	1110059E24Ri	Mmu19	21664410
14735	ENSG00000119121	TRPM6	Hsa9	76527231	ENSMUSG00000024727	Trpm6	Mmu19	18817002
14740	ENSG00000165072	MAMDC2	Hsa9	71848317	ENSMUSG00000033207	Mamdc2	Mmu19	23369706
14750	ENSG00000119138	KLF9	Hsa9	72189323	ENSMUSG00000033863	Klf9	Mmu19	23208323
14795	ENSG00000181778	C9orf71	Hsa9	70341316	ENSMUSG00000048572	E030010A14Ri	Mmu19	24741102
15046	ENSG00000160916	ATP5J2	Hsa7	98893721	ENSMUSG00000038690	Atp5j2	Mmu5	145437366
15046	ENSG00000171995	653324	Hsa12	6249586	ENSMUSG00000038690	Atp5j2	Mmu5	145437366
15046	ENSG00000198475		Hsa17	59918102	ENSMUSG00000038690	Atp5j2	Mmu5	145437366
15046	ENSG00000185796		Hsa6	109335505	ENSMUSG00000038690	Atp5j2	Mmu5	145437366
15046	ENSG00000186390	645225	Hsa9	78845137	ENSMUSG00000038690	Atp5j2	Mmu5	145437366

Genomic distribution of the Mouse orthologos of the Human 10-Mb GN surrounding the human ALDH1A2.								
Genomic region analyzed: human chromosome 15 (51.000 KB - 61.000 KB)								
Group ID	Query Gene ID	Query Ext ID	Query Chromosome	Query Start Base	Outg Gene ID	Outg Ext ID	Outg Chromosome	Outg Start Base
1760	ENSG00000134333	LDHA	Hsa11	18372683	ENSMUSG00000063229	Ldha	Mmu7	46713883
1760	ENSG00000166800	LDHAL6A	Hsa11	18434007	ENSMUSG00000063229	Ldha	Mmu7	46713883
1760	ENSG00000171989	LDHAL6B	Hsa15	57286314	ENSMUSG00000063229	Ldha	Mmu7	46713883
1760	ENSG00000178001		Hsa12	61683600	ENSMUSG00000063229	Ldha	Mmu7	46713883
3733	ENSG00000176244	ACBD7	Hsa10	15157481	ENSMUSG00000026644	9230116B18Ri	Mmu2	3257539
3733	ENSG00000140238	650875	Hsa15	54925830	ENSMUSG00000026644	9230116B18Ri	Mmu2	3257539
5567	ENSG00000137876	C15orf15	Hsa15	53260813	ENSMUSG00000053740	BC003885	Mmu18	14713131
5567	ENSG00000141650	284288	Hsa18	53656706	ENSMUSG00000053740	BC003885	Mmu18	14713131
6834	ENSG00000205581	HMGN1	Hsa21	39636111	ENSMUSG00000040681	Hmgn1	Mmu16	96225528
6834	ENSG00000198875	731104	Hsa2	97810143	ENSMUSG00000040681	Hmgn1	Mmu16	96225528
6834	ENSG00000157576	XR_018454.1	Hsa15	91055981	ENSMUSG00000040681	Hmgn1	Mmu16	96225528
6834	ENSG00000174558	728325	Hsa11	3966963	ENSMUSG00000040681	Hmgn1	Mmu16	96225528
6834	ENSG00000184409	728776	HsaX	153698568	ENSMUSG00000040681	Hmgn1	Mmu16	96225528
6834	ENSG00000172186	388476	Hsa18	53837019	ENSMUSG00000040681	Hmgn1	Mmu16	96225528
6834	ENSG00000182015		Hsa15	60441529	ENSMUSG00000040681	Hmgn1	Mmu16	96225528
6834	ENSG00000197366		Hsa10	124643960	ENSMUSG00000040681	Hmgn1	Mmu16	96225528
6834	ENSG00000182765	731576	Hsa18	56612821	ENSMUSG00000040681	Hmgn1	Mmu16	96225528
6834	ENSG00000183731		Hsa17	38550499	ENSMUSG00000040681	Hmgn1	Mmu16	96225528
8911	ENSG00000145425	RPS3A	Hsa4	152240204	ENSMUSG00000028081	Rps3a	Mmu3	86223873
8911	ENSG00000187899		Hsa15	57847843	ENSMUSG00000028081	Rps3a	Mmu3	86223873
8911	ENSG00000205871	648659	Hsa15	41195189	ENSMUSG00000028081	Rps3a	Mmu3	86223873
8911	ENSG00000178429	XR_017186.1	Hsa4	153691882	ENSMUSG00000028081	Rps3a	Mmu3	86223873
9067	ENSG00000166035	LIPC	Hsa15	56511467	ENSMUSG00000032207	Lipc	Mmu9	70597343
9080	ENSG00000128845	CCPG1	Hsa15	53434741	ENSMUSG00000034563	Ccpg1	Mmu9	72782710
9089	ENSG00000137776	SLTM	Hsa15	56958537	ENSMUSG00000032212	Sltm	Mmu9	70342023
9095	ENSG00000128923	FAM63B	Hsa15	56850806	ENSMUSG00000042444	B230380D07Ri	Mmu9	70403733

9113	ENSG00000069974	RAB27A	Hsa15	53283094	ENSMUSG00000032202	Rab27a	Mmu9	72841997
9146	ENSG00000171956	FOXB1	Hsa15	58084427	ENSMUSG00000059246	Foxb1	Mmu9	69556916
9163	ENSG00000103569	AQP9	Hsa15	56217771	ENSMUSG00000032204	Aqp9	Mmu9	70909221
9167	ENSG00000181827	RFXDC2	Hsa15	54166958	ENSMUSG00000037674	Rfxdc2	Mmu9	72330807
9174	ENSG00000140299	BNIP2	Hsa15	57742356	ENSMUSG00000011958	Bnip2	Mmu9	69788665
9261	ENSG00000140307	GTF2A2	Hsa15	57718358	ENSMUSG00000033543	Gtf2a2	Mmu9	69811714
9266	ENSG00000069943	PIGB	Hsa15	53398741	ENSMUSG00000032203	Pigb	Mmu9	72805613
9283	ENSG00000171016	PYGO1	Hsa15	53625513	ENSMUSG00000034910	Pygo1	Mmu9	72722780
9298	ENSG00000129003	VPS13C	Hsa15	59931884	ENSMUSG00000035284	Vps13c	Mmu9	67639576
9331	ENSG00000151575	TEX9	Hsa15	54444936	ENSMUSG00000032219	Tex9	Mmu9	72257146
9364	ENSG00000157450	RNF111	Hsa15	57067157	ENSMUSG00000032217	Rnf111	Mmu9	70224593
9368	ENSG00000138587	MNS1	Hsa15	54508299	ENSMUSG00000032221	Mns1	Mmu9	72237807
9372	ENSG00000166450	PRTG	Hsa15	53699353	ENSMUSG00000036030	Prtg	Mmu9	72606024
9399	ENSG00000128849	CGNL1	Hsa15	55455997	ENSMUSG00000032232	Cgnl1	Mmu9	71425069
9524	ENSG00000140297	GCNT3	Hsa15	57691415	ENSMUSG00000032226	Gcnt3	Mmu9	69832709
9565	ENSG00000140262	TCF12	Hsa15	54998125	ENSMUSG00000032228	Tcf12	Mmu9	71644313
9622	ENSG00000128918	ALDH1A2	Hsa15	56032920	ENSMUSG00000013584	Aldh1a2	Mmu9	71014382
9623	ENSG00000137845	ADAM10	Hsa15	56675802	ENSMUSG00000054693	Adam10	Mmu9	70478203
9651	ENSG00000157470	FAM81A	Hsa15	57517664	ENSMUSG00000032224	6430514L14Ri	Mmu9	69889931
9651	ENSG00000153347	FAM81B	Hsa5	94754247	ENSMUSG00000032224	6430514L14Ri	Mmu9	69889931
9734	ENSG00000178620	GRINL1B	Hsa4	68586857	ENSMUSG00000032199	Grinl1a	Mmu9	71277019
9734	ENSG00000137878	GRINL1A	Hsa15	55671406	ENSMUSG00000032199	Grinl1a	Mmu9	71277019
9793	ENSG00000128915	NARG2	Hsa15	58499100	ENSMUSG00000032235	Narg2	Mmu9	69197162
9796	ENSG00000137871	SUHW4	Hsa15	54709671	ENSMUSG00000038535	Suhw4	Mmu9	72073547
9853	ENSG00000069869	NEDD4	Hsa15	53906423	ENSMUSG00000032216	Nedd4	Mmu9	72461124
9864	ENSG00000157456	CCNB2	Hsa15	57184612	ENSMUSG00000032218	Ccnb2	Mmu9	70206853
9867	ENSG00000069667	RORA	Hsa15	58576755	ENSMUSG00000032238	Rora	Mmu9	68452971
9884	ENSG00000166415	WDR72	Hsa15	51594673	ENSMUSG00000044976	Wdr72	Mmu9	73907488
9910	ENSG00000198535	NP_997205.1	Hsa15	60146506	ENSMUSG00000055125	3300001A09Ri	Mmu9	67558602
9910	ENSG00000205502	NP_001007596	Hsa15	60243294	ENSMUSG00000055125	3300001A09Ri	Mmu9	67558602
9925	ENSG00000157483	MYO1E	Hsa15	57215461	ENSMUSG00000032220	Myo1e	Mmu9	70086076
9927	ENSG00000182718	ANXA2	Hsa15	58426643	ENSMUSG00000032231	Anxa2	Mmu9	69252847
9927	ENSG00000183059	ANXA2P2	Hsa9	33614223	ENSMUSG00000032231	Anxa2	Mmu9	69252847
12608	ENSG00000182625		Hsa15	56765902	ENSMUSG00000038607	Gng10	Mmu4	59129210
12776	ENSG00000137076	TLN1	Hsa9	35687336	ENSMUSG00000028465	Tln1	Mmu4	43552618
12776	ENSG00000171914	TLN2	Hsa15	60726802	ENSMUSG00000028465	Tln1	Mmu4	43552618
13384	ENSG00000198722	UNC13B	Hsa9	35151999	ENSMUSG00000028456	Unc13b	Mmu4	43108981
13384	ENSG00000137766	UNC13C	Hsa15	52314521	ENSMUSG00000028456	Unc13b	Mmu4	43108981
14799	ENSG00000166466	440280	Hsa15	54085203	ENSMUSG00000034724	Cnot6l	Mmu5	96315891
14799	ENSG00000138767	CNOT6L	Hsa4	78857199	ENSMUSG00000034724	Cnot6l	Mmu5	96315891

Genomic distribution of the Mouse orthologos of the Human 10-Mb GN surrounding the human ALDH1A3.								
Genomic region analyzed: human chromosome 15 (90.000 KB - 100.000 KB)								
Group ID	Query Gene ID	Query Ext ID	Query Chromosome	Query Start Base	Outg Gene ID	Outg Ext ID	Outg Chromosome	Outg Start Base
1301	ENSG00000131873	CHSY1	Hsa15	99533456	ENSMUSG00000032640	Chsy1	Mmu7	65988465
1301	ENSG00000198108	CHSS3_HUMAN	Hsa5	129268422	ENSMUSG00000032640	Chsy1	Mmu7	65988465
1318	ENSG00000140450	ARRDC4	Hsa15	96304947	ENSMUSG00000042659	Arrdc4	Mmu7	68612993
1328	ENSG00000183571	145814	Hsa15	97328859	ENSMUSG00000030553	C330024D12Ri	Mmu7	68110123
1377	ENSG00000176463	SLCO3A1	Hsa15	90197950	ENSMUSG00000025790	Slco3a1	Mmu7	74148931
1497	ENSG00000168904	LRRC28	Hsa15	97609175	ENSMUSG00000030556	Lrrc28	Mmu7	67386924
1522	ENSG00000140471	LINS1	Hsa15	98926957	ENSMUSG00000053091	Lins2	Mmu7	66568908
1530	ENSG00000183060	LYSMD4	Hsa15	98073429	ENSMUSG00000043831	Lysmd4	Mmu7	67101558
1546	ENSG00000140557	ST8SIA2	Hsa15	90738144	ENSMUSG00000025789	St8sia2	Mmu7	73812635
1643	ENSG00000103852	TTC23	Hsa15	97494052	ENSMUSG00000030555	Ttc23	Mmu7	67520954
1690	ENSG00000140443	IGF1R	Hsa15	97010302	ENSMUSG00000005533	Igf1r	Mmu7	67826372
1696	ENSG00000173575	CHD2	Hsa15	91244423	ENSMUSG00000025788	Chd2	Mmu7	73325212
1735	ENSG00000182175	RGMA	Hsa15	91387651	ENSMUSG00000070509	Rgma	Mmu7	73249020
1752	ENSG00000184254	ALDH1A3	Hsa15	99237580	ENSMUSG00000015134	Aldh1a3	Mmu7	66269840
1766	ENSG00000154237	LRRK1	Hsa15	99277073	ENSMUSG00000015133	C230002E15Ri	Mmu7	66137693
1774	ENSG00000131871	SELS_HUMAN	Hsa15	99628737	ENSMUSG00000075701	H47	Mmu7	65958664
1882	ENSG00000183475	ASB7	Hsa15	98960337	ENSMUSG00000030509	Asb7	Mmu7	66523534
1933	ENSG00000154227	LASS3	Hsa15	98758124	ENSMUSG00000030510	XP_620510.2	Mmu7	66643126
2037	ENSG00000182253	DMN	Hsa15	97462771	ENSMUSG00000030554	Dmn	Mmu7	67606115
2069	ENSG00000185551	NR2F2	Hsa15	94674950	ENSMUSG00000030551	Nr2f2	Mmu7	70226952
2150	ENSG00000131876	SNRPA1	Hsa15	99639240	ENSMUSG00000030512	Snrpa1	Mmu7	65939512
2150	ENSG00000144158	Q9NU36_HUMAN	Hsa2	114131955	ENSMUSG00000030512	Snrpa1	Mmu7	65939512
2163	ENSG00000140479	PCSK6	Hsa15	99661657	ENSMUSG00000030513	Pcsk6	Mmu7	65741163
2286	ENSG00000140563	MCTP2	Hsa15	92575955	ENSMUSG00000032776	Gm489	Mmu7	71951343
2292	ENSG00000185087	NP_872368.2	Hsa15	96800383	ENSMUSG00000074071	NP_001013833	Mmu7	68174341
2364	ENSG00000068305	MEF2A	Hsa15	97923712	ENSMUSG00000030557	Mef2a	Mmu7	67113818
5328	ENSG00000174132	TMEM157	Hsa5	99898908	ENSMUSG00000051185	2310044D20Ri	Mmu1	97144034
5328	ENSG00000185442	NP_997329.1	Hsa15	90961685	ENSMUSG00000051185	2310044D20Ri	Mmu1	97144034
6834	ENSG00000205581	HMGN1	Hsa21	39636111	ENSMUSG00000040681	Hmgn1	Mmu16	96225528
6834	ENSG00000198875	731104	Hsa2	97810143	ENSMUSG00000040681	Hmgn1	Mmu16	96225528
6834	ENSG00000157576	XR_018454.1	Hsa15	91055981	ENSMUSG00000040681	Hmgn1	Mmu16	96225528
6834	ENSG00000174558	728325	Hsa11	3966963	ENSMUSG00000040681	Hmgn1	Mmu16	96225528
6834	ENSG00000184409	728776	HsaX	153698568	ENSMUSG00000040681	Hmgn1	Mmu16	96225528
6834	ENSG00000172186	388476	Hsa18	53837019	ENSMUSG00000040681	Hmgn1	Mmu16	96225528
6834	ENSG00000182015		Hsa15	60441529	ENSMUSG00000040681	Hmgn1	Mmu16	96225528
6834	ENSG00000197366		Hsa10	124643960	ENSMUSG00000040681	Hmgn1	Mmu16	96225528
6834	ENSG00000182765	731576	Hsa18	56612821	ENSMUSG00000040681	Hmgn1	Mmu16	96225528
6834	ENSG00000183731		Hsa17	38550499	ENSMUSG00000040681	Hmgn1	Mmu16	96225528

Genomic distribution of the Mouse orthologos of the Human 10-Mb GN related to the human ALDH1A3-ogm.								
Genomic regiona analyzed: human chromosome 5 (90000 KB - 100000 KB)								
Group ID	Query Gene ID	Query Ext ID	Query Chromosome	Query Start Base	Outg Gene ID	Outg Ext ID	Outg Chromosome	Outg Start Base
2641	ENSG00000145721	LIX1	Hsa5	96453330	ENSMUSG00000047786	Lix1	Mmu17	17107511
2731	ENSG00000113441	LNPEP	Hsa5	96296924	ENSMUSG00000023845	Lnpep	Mmu17	17232341
2838	ENSG00000058729	RIOK2	Hsa5	96522327	ENSMUSG00000023846	Riok2	Mmu17	17079190
3047	ENSG00000153922	CHD1	Hsa5	98218819	ENSMUSG00000023852	Chd1	Mmu17	15409664
3166	ENSG00000174136	RGMB	Hsa5	98132900	ENSMUSG00000048027	Rgmb	Mmu17	15509352
5328	ENSG00000174132	TMEM157	Hsa5	99898908	ENSMUSG00000051185	2310044D20Ri	Mmu1	97144034
5328	ENSG00000185442	NP_997329.1	Hsa15	90961685	ENSMUSG00000051185	2310044D20Ri	Mmu1	97144034
5925	ENSG00000175449	RFESD	Hsa5	95008239	ENSMUSG00000043190	AI256775	Mmu13	76467537
5972	ENSG00000173221	GLRX	Hsa5	95175309	ENSMUSG00000021591	Glrx	Mmu13	76305916
5972	ENSG00000118990		Hsa5	161110059	ENSMUSG00000021591	Glrx	Mmu13	76305916
6027	ENSG00000113369	ARRDC3	Hsa5	90700299	ENSMUSG00000074794	Arrdc3	Mmu13	81347107
6033	ENSG00000145757	SPATA9	Hsa5	95013641	ENSMUSG00000021590	Spata9	Mmu13	76433741
6056	ENSG00000118985	ELL2	Hsa5	95246558	ENSMUSG00000001542	Ell2	Mmu13	76173653
6064	ENSG00000164307	ARTS1_HUMAN	Hsa5	96122277	ENSMUSG00000021583	Arts1	Mmu13	75105875
6064	ENSG00000164308	NP_071745.1	Hsa5	96237474	ENSMUSG00000021583	Arts1	Mmu13	75105875
6074	ENSG00000175426	PCSK1	Hsa5	95751875	ENSMUSG00000021587	Pcsk1	Mmu13	75555989
6099	ENSG00000198677	KIAA0372	Hsa5	94825355	ENSMUSG00000033991	AK129128	Mmu13	76564804
6200	ENSG00000178015	GPR150	Hsa5	94981538	ENSMUSG00000045509	Gpr150	Mmu13	76521543
6218	ENSG00000113391	C5orf21	Hsa5	92979536	ENSMUSG00000064138	1110033M05Ri	Mmu13	78172266
6218	ENSG00000175841	131909	Hsa3	102723845	ENSMUSG00000064138	1110033M05Ri	Mmu13	78172266
6220	ENSG00000153113	CAST	Hsa5	96023533	ENSMUSG00000021585	Cast	Mmu13	75160288
6226	ENSG00000175471	MCTP1	Hsa5	94068601	ENSMUSG00000021596	Mctp1	Mmu13	77105255
6291	ENSG00000164292	RHOBTB3	Hsa5	95092635	ENSMUSG00000021589	Rhobtb3	Mmu13	76335549
6322	ENSG00000164291	ARSK	Hsa5	94916608	ENSMUSG00000021592	Arsk	Mmu13	76526714
6347	ENSG00000175745	NR2F1	Hsa5	92944799	ENSMUSG00000069171	Nr2f1	Mmu13	78652996
9651	ENSG00000157470	FAM81A	Hsa15	57517664	ENSMUSG00000032224	6430514L14Ri	Mmu9	69889931
9651	ENSG00000153347	FAM81B	Hsa5	94754247	ENSMUSG00000032224	6430514L14Ri	Mmu9	69889931
10801	ENSG00000092010	PSME1	Hsa14	23661207	ENSMUSG00000022216	Psme1	Mmu14	54532646
10801	ENSG00000100911	PSME2	Hsa14	23682449	ENSMUSG00000022216	Psme1	Mmu14	54532646
10801	ENSG00000174144		Hsa5	97574862	ENSMUSG00000022216	Psme1	Mmu14	54532646
