# Supplementary material for: Consequences of Lineage-Specific Gene Loss on Functional Evolution of Surviving Paralogs: ALDH1A and Retinoic Acid Signaling in Vertebrate Genomes
Source: PLoS Genet. 2009 May 29;5(5):e1000496. doi: 10.1371/journal.pgen.1000496 (PMC2682703; doi:10.1371/journal.pgen.1000496)
Supplement: Table S3 — Supplementary information for dot-plots on Figure 4A–C. (0.16 MB PDF) [file pgen.1000496.s005.pdf]

Table S3. Supplementary information for dot-plots on Figure 4A-C

Genomic information of teleost orthologs of the human ALDH1A1 gene neighbors.								
Genomic region analyzed: human chromosome 9 (69.000 KB - 79.000 KB)								
Zebrafish								
Pred. Chrom.	Pred Start Base	Pred Gene ID	Pred Ext ID	Query Chromosome	Query Start Base	Query Gene ID	Query Ext ID	Group ID
Hsa9	69137176	ENSG00000197550	Q9UC05_HUMAN					0
Hsa9	69433592	ENSG00000204780	644704					0
Hsa9	69465527	ENSG00000204779	XR_016691.1					0
Hsa9	69666443	ENSG00000184659	FOXD4L4					0
Hsa9	69671821	ENSG00000172785	CBWD5					0
Hsa9	70046217	ENSG00000196873	CBWD6					0
Hsa9	70107603	ENSG00000187559	FOXD4L3					0
Hsa9	70161635	ENSG00000154330	PGM5	Dre8	29046576	ENSDARG00000060745	LOC560297	6724
Hsa9	70510436	ENSG00000107242	PIP5K1B	Dre8	6070209	ENSDARG00000006508	zgc:92316	6667
Hsa9	70510436	ENSG00000107242	PIP5K1B	Dre10	5932600	ENSDARG00000044295	zgc:101046	6667
Hsa9	70584784	ENSG00000187866	FAM122A	Dre14	35767045	ENSDARG00000036500	zgc:91872	6584
Hsa9	70817241	ENSG00000165059	PRKACG					0
Hsa9	70839995	ENSG00000165060	FXN	Dre8	6161096	ENSDARG00000069502	zgc:158218	6550
Hsa9	70956382	ENSG00000119139	TJP2	Dre10	5869777	ENSDARG00000063309	LOC565446	6623
Hsa9	71130168	ENSG00000135063	C9orf61	Dre5	43146668	ENSDARG00000059671	si:ch211-62b4.3	6585
Hsa9	71235022	ENSG00000107282	APBA1	Dre5	43195749	ENSDARG00000014105	A2BIL2_DANRE	6553
Hsa9	71514261	ENSG00000188647	PTAR1	Dre5	43052740	ENSDARG00000067695	A3KPW7_DANRE	6679
Hsa9	71514261	ENSG00000188647	PTAR1	Dre5	43287264	ENSDARG00000067693	Q08CA2_DANRE	6679
Hsa9	71848317	ENSG00000165072	MAMDC2	Dre5	42964994	ENSDARG00000059677	A3KPW6_DANRE	6752
Hsa9	72063757	ENSG00000198887	SMC5					0
Hsa9	72189323	ENSG00000119138	KLF9	Dre2	4170986	ENSDARG00000069342	zgc:153115	6406
Hsa9	72339769	ENSG00000083067	TRPM3	Dre5	19484954	ENSDARG00000039181	A2BGS8_DANRE	6691
Hsa9	73488102	ENSG00000135048	TMEM2	Dre5	19735877	ENSDARG00000061600	TMEM2_DANRE	6664
Hsa9	73667188	ENSG00000107362	C9orf77	Dre5	19795084	ENSDARG00000035571	zgc:100937	6448
Hsa9	73667188	ENSG00000107362	C9orf77	Dre8	16997383	ENSDARG00000043084	LOC100003419	6448
Hsa9	73667188	ENSG00000107362	C9orf77	Dre7	5634536	ENSDARG00000058452	zgc:55468	6448
Hsa9	73716243	ENSG00000155621	C9orf85	Dre5	19814541	ENSDARG00000035570	zgc:101016	6685
Hsa9	73954113	ENSG00000119125	GDA	Dre5	19825227	ENSDARG00000002986	zgc:112282	6649
Hsa9	74159194	ENSG00000107372	ZFAND5	Dre10	5654373	ENSDARG00000002271	zfand5b	6671
Hsa9	74159194	ENSG00000107372	ZFAND5	Dre5	19853930	ENSDARG00000018898	zfand5a	6671
Hsa9	74159194	ENSG00000107372	ZFAND5	Dre8	17021016	ENSDARG00000027832	zgc:77486	6671
Hsa9	74326537	ENSG00000165091	TMC1	Dre5	19881047	ENSDARG00000056386	A2BFV3_DANRE	6435
Hsa9	74705408	ENSG00000165092	ALDH1A1					0
Hsa9	74956493	ENSG00000135046	ANXA1	Dre5	19965166	ENSDARG00000026726	anxa1a	6694
Hsa9	74956493	ENSG00000135046	ANXA1	Dre5	58054449	ENSDARG00000043936	zgc:110283	6694
Hsa9	74956493	ENSG00000135046	ANXA1	Dre5	58089685	ENSDARG00000014594	anxa1b	6694
Hsa9	74956493	ENSG00000135046	ANXA1	Dre5	58077076	ENSDARG00000011168	anxa1c	6694
Hsa9	76302072	ENSG00000198963	RORB	Dre5	20141484	ENSDARG00000033498	NP_001076325.1	6496
Hsa9	76302072	ENSG00000198963	RORB	Dre20	9423323	ENSDARG00000017780	si:ch211-168n16.2	6496
Hsa9	76302072	ENSG00000198963	RORB	Dre8	16394665	ENSDARG00000057231	rorg	6496
Hsa9	76527231	ENSG00000119121	TRPM6	Dre1	102486	ENSDARG00000058478	LOC799765	6505
Hsa9	76527231	ENSG00000119121	TRPM6	Dre18	43561871	ENSDARG00000036232	TRPM7	6505
Hsa9	76785754	ENSG00000156017	C9orf41	Dre5	17001102	ENSDARG00000031102	zgc:112985	6430
Hsa9	76865309	ENSG00000106733	C9orf95	Dre3	32364715	ENSDARG00000037913	zgc:113113	6595
Hsa9	76893210	ENSG00000134996	OSTF1	Dre5	2269383	ENSDARG00000007120	ostf1	6718
Hsa9	76893210	ENSG00000134996	OSTF1	Dre5	2345148	ENSDARG00000045096	ostf1	6718
Hsa9	77222244	ENSG00000189225	NULL					0
Hsa9	77695406	ENSG00000099139	PCSK5	Dre5	65117737	ENSDARG00000067537	NP_001071050.1	6693
Hsa9	77695406	ENSG00000099139	PCSK5	Dre23	44611446	ENSDARG00000060518	NP_001077298.1	6693
Hsa9	78190253	ENSG00000135002	RFK	Dre23	44526206	ENSDARG00000060522	RFK	6574
Hsa9	78263966	ENSG00000187210	GCNT1					0
Hsa9	78419138	ENSG00000106772	KIAA0367	Dre5	65073507	ENSDARG00000059423	A2BHH5_DANRE	6766
Hsa9	78824391	ENSG00000204612	FOXB2					0
Hsa9	78845137	ENSG00000186390	645225					0
Hsa9	78982181	ENSG00000197969	VPS13A					0
Stickelback								
Pred Chrom.	Pred Start Base	Pred Gene ID	Pred Ext ID	Query Chromosome	Query Start Base	Query Gene ID	Query Ext ID	Group ID
Hsa9	69066931	ENSG00000156755	442423					0
Hsa9	69433592	ENSG00000204780	644704					0
Hsa9	69465527	ENSG00000204779	XR_016691.1					0
Hsa9	69634070	ENSG00000204776	644731					0
Hsa9	69666443	ENSG00000184659	FOXD4L4					0
Hsa9	69671821	ENSG00000172785	CBWD5					0
Hsa9	70046217	ENSG00000196873	CBWD6					0
Hsa9	70107603	ENSG00000187559	FOXD4L3					0
Hsa9	70161635	ENSG00000154330	PGM5	GacgroupXIII	6163585	ENSGACG00000007106	PGM5P1	7303
Hsa9	70510436	ENSG00000107242	PIP5K1B	GacgroupXIV	10609220	ENSGACG00000017952	PIP5K1B (2 of 2)	7182

Hsa9	70510436	ENSG00000107242	PIP5K1B	GacgroupXIII	9774455	ENSGACG00000010008	PIP5K1B (1 of 2)	7182
Hsa9	70584784	ENSG00000187866	FAM122A	GacgroupIV	15949511	ENSGACG00000018631	FAM122B	7479
Hsa9	70817241	ENSG00000165059	PRKACG					0
Hsa9	70956382	ENSG00000119139	TJP2	GacgroupXIV	10620803	ENSGACG00000017956	TJP2	7365
Hsa9	71130168	ENSG00000135063	C9orf61					0
Hsa9	71235022	ENSG00000107282	APBA1					0
Hsa9	71514261	ENSG00000188647	PTAR1	GacgroupXIII	2754573	ENSGACG00000005110	PTAR1	7505
Hsa9	71848317	ENSG00000165072	MAMDC2	GacgroupXIII	2730005	ENSGACG00000005103	MAMDC2 (1 of 2)	7366
Hsa9	71848317	ENSG00000165072	MAMDC2	GacgroupXIV	10665556	ENSGACG00000017970	MAMDC2 (2 of 2)	7366
Hsa9	72063757	ENSG00000198887	SMC5	GacgroupXII	10418164	ENSGACG00000008208	SMC5	7566
Hsa9	72189323	ENSG00000119138	KLF9	GacgroupIII	9147903	ENSGACG00000016005		7455
Hsa9	72189323	ENSG00000119138	KLF9	GacgroupXIII	11679463	ENSGACG00000010668		7455
Hsa9	72339769	ENSG00000083067	TRPM3	GacgroupXIII	11689075	ENSGACG00000010674	TRPM3	7187
Hsa9	73488102	ENSG00000135048	TMEM2	GacgroupXIII	11802201	ENSGACG00000010690	TMEM2	7297
Hsa9	73667188	ENSG00000107362	C9orf77	GacgroupXIII	11824166	ENSGACG00000010702	FAM108B1	7497
Hsa9	73667188	ENSG00000107362	C9orf77	GacgroupII	133721	ENSGACG00000013938	FAM108C1	7497
Hsa9	73716243	ENSG00000155621	C9orf85	GacgroupXIII	11834354	ENSGACG00000010713	C9orf85	7113
Hsa9	73954113	ENSG00000119125	GDA	GacgroupXIII	11838527	ENSGACG00000010716	GDA	7361
Hsa9	74159194	ENSG00000107372	ZFAND5	GacgroupXIII	12977894	ENSGACG00000011494	ZFAND5 (1 of 2)	7469
Hsa9	74159194	ENSG00000107372	ZFAND5	GacgroupXIV	10700890	ENSGACG00000018012	ZFAND5 (2 of 2)	7469
Hsa9	74159194	ENSG00000107372	ZFAND5	GacgroupVIII	15616208	ENSGACG00000012323	ZFAND6	7469
Hsa9	74326537	ENSG00000165091	TMC1	GacgroupXIII	12990197	ENSGACG00000011501	TMC1	7176
Hsa9	74705408	ENSG00000165092	ALDH1A1					0
Hsa9	74956493	ENSG00000135046	ANXA1	GacgroupXIII	13024512	ENSGACG00000011546	ANXA1 (1 of 2)	7453
Hsa9	74956493	ENSG00000135046	ANXA1	GacgroupXIV	10682406	ENSGACG00000017986	ANXA1 (2 of 2)	7453
Hsa9	76302072	ENSG00000198963	RORB	GacgroupXIII	13107695	ENSGACG00000011556	RORB	7449
Hsa9	76302072	ENSG00000198963	RORB	GacgroupVIII	15473521	ENSGACG00000012280		7449
Hsa9	76527231	ENSG00000119121	TRPM6	GacgroupXIII	13115208	ENSGACG00000011569	TRPM6	7555
Hsa9	76785754	ENSG00000156017	C9orf41	GacgroupXIII	13135353	ENSGACG00000011583	C9orf41	7257
Hsa9	76865309	ENSG00000106733	C9orf95	GacgroupXIII	5014235	ENSGACG00000006361	C9orf95	7340
Hsa9	76893210	ENSG00000134996	OSTF1	GacgroupXIII	4982724	ENSGACG00000006313	OSTF1	7382
Hsa9	77222244	ENSG00000189225	NULL					0
Hsa9	77695406	ENSG00000099139	PCSK5					0
Hsa9	78190253	ENSG00000135002	RFK	GacgroupXIII	8084061	ENSGACG00000008000	RFK	7538
Hsa9	78263966	ENSG00000187210	GCNT1	GacgroupXIII	8044620	ENSGACG00000007993	GCNT1	7363
Hsa9	78354018	ENSG00000184468	XR_018300.1					0
Hsa9	78419138	ENSG00000106772	KIAA0367	GacgroupXIV	3316483	ENSGACG00000016173	BMCC1_HUMAN (1 of 2)	7189
Hsa9	78419138	ENSG00000106772	KIAA0367	GacgroupXIII	8036155	ENSGACG00000007984	BMCC1_HUMAN (2 of 2)	7189
Hsa9	78593080	ENSG00000156035	C9orf65	GacgroupXIII	8014429	ENSGACG00000007980		7481
Hsa9	78824391	ENSG00000204612	FOXB2					0
Hsa9	78845137	ENSG00000186390	645225					0
Hsa9	78982181	ENSG00000197969	VPS13A	GacgroupXIII	7967387	ENSGACG00000007925	VPS13A	7302
Medaka								
Pred Chrom.	Pred Start Base	Pred Gene ID	Pred Ext ID	Query Chromosome	Query Start Base	Query Gene ID	Query Ext ID	Group ID
Hsa9	69066931	ENSG00000156755	442423					0
Hsa9	69433592	ENSG00000204780	644704					0
Hsa9	69465527	ENSG00000204779	XR_016691.1					0
Hsa9	69634070	ENSG00000204776	644731					0
Hsa9	69666443	ENSG00000184659	FOXD4L4					0
Hsa9	70107603	ENSG00000187559	FOXD4L3					0
Hsa9	70161635	ENSG00000154330	PGM5	Ola9	8468682	ENSORLG00000004500	PGM5	6706
Hsa9	70341316	ENSG00000181778	C9orf71	Ola9	8493715	ENSORLG00000004507	C9orf71	6596
Hsa9	70510436	ENSG00000107242	PIP5K1B	Ola9	13597905	ENSORLG00000009286	PIP5K1B (1 of 2)	7003
Hsa9	70510436	ENSG00000107242	PIP5K1B	Ola12	24959474	ENSORLG00000013413	PIP5K1B (2 of 2)	7003
Hsa9	70584784	ENSG00000187866	FAM122A	Ola10	23363320	ENSORLG00000009949	FAM122A	6897
Hsa9	70817241	ENSG00000165059	PRKACG					0
Hsa9	70839995	ENSG00000165060	FXN	Olascaffold549	164981	ENSORLG00000018621	FXN	6833
Hsa9	70956382	ENSG00000119139	TJP2	Ola12	25015498	ENSORLG00000013474	TJP2	7018
Hsa9	71130168	ENSG00000135063	C9orf61	Ola3	16947492	ENSORLG00000006322		6762
Hsa9	71235022	ENSG00000107282	APBA1	Ola9	30595598	ENSORLG00000017317	APBA1 (2 of 2)	6763
Hsa9	71514261	ENSG00000188647	PTAR1	Olascaffold2180	6660	ENSORLG00000020189	PTAR1	6795
Hsa9	71848317	ENSG00000165072	MAMDC2	Ola12	25098496	ENSORLG00000013510	MAMDC2	6623
Hsa9	72063757	ENSG00000198887	SMC5	Ola7	11291820	ENSORLG00000006574	SMC5	6635
Hsa9	72189323	ENSG00000119138	KLF9	Ola17	13941628	ENSORLG00000008372		6812
Hsa9	72339769	ENSG00000083067	TRPM3	Ola9	16294768	ENSORLG00000010741	TRPM3	6755
Hsa9	73488102	ENSG00000135048	TMEM2	Ola9	16464961	ENSORLG00000010792	TMEM2	6883
Hsa9	73667188	ENSG00000107362	C9orf77	Ola9	16492835	ENSORLG00000010800	FAM108B1	6876
Hsa9	73716243	ENSG00000155621	C9orf85	Ola9	16511736	ENSORLG00000010809	C9orf85	6691
Hsa9	73954113	ENSG00000119125	GDA	Ola9	16520074	ENSORLG00000010864	GDA	6934
Hsa9	74159194	ENSG00000107372	ZFAND5	Ola9	18184802	ENSORLG00000012307	ZFAND5 (2 of 2)	6760
Hsa9	74159194	ENSG00000107372	ZFAND5	Ola12	25262226	ENSORLG00000013620	ZFAND5 (1 of 2)	6760
Hsa9	74159194	ENSG00000107372	ZFAND5	Ola4	31317043	ENSORLG00000016444	ZFAND6	6760
Hsa9	74326537	ENSG00000165091	TMC1	Ola9	18206499	ENSORLG00000012318	TMC1	6875
Hsa9	74705408	ENSG00000165092	ALDH1A1					0

Hsa9	74956493	ENSG00000135046	ANXA1	Ola9	18265548	ENSORLG00000012381	O93446_ORYLA	6666
Hsa9	74956493	ENSG00000135046	ANXA1	Ola12	25135176	ENSORLG00000013570	ANXA1 (2 of 2)	6666
Hsa9	76302072	ENSG00000198963	RORB	Ola9	18383631	ENSORLG00000012441	RORB	6780
Hsa9	76302072	ENSG00000198963	RORB	Ola4	7102771	ENSORLG00000003765		6780
Hsa9	76527231	ENSG00000119121	TRPM6					0
Hsa9	76785754	ENSG00000156017	C9orf41	Ola9	18416990	ENSORLG00000012507	C9orf41	6759
Hsa9	76865309	ENSG00000106733	C9orf95					0
Hsa9	76893210	ENSG00000134996	OSTF1	Ola9	6794902	ENSORLG00000003643	OSTF1	6848
Hsa9	77222244	ENSG00000189225	NULL					0
Hsa9	77695406	ENSG00000099139	PCSK5	Ola9	11336698	ENSORLG00000005789	PCSK5	6746
Hsa9	78190253	ENSG00000135002	RFK	Ola9	11310260	ENSORLG00000005753	RFK	6588
Hsa9	78263966	ENSG00000187210	GCNT1	Ola9	11299727	ENSORLG00000005742	GCNT1	6726
Hsa9	78419138	ENSG00000106772	KIAA0367	Ola12	7633324	ENSORLG00000004782		6764
Hsa9	78419138	ENSG00000106772	KIAA0367	Ola9	11281571	ENSORLG00000005730		6764
Hsa9	78824391	ENSG00000204612	FOXB2					0
Hsa9	78845137	ENSG00000186390	645225					0
Hsa9	78982181	ENSG00000197969	VPS13A					0

Genomic information of teleost orthologs of the human ALDH1A2 gene neighbors.

Genomic region analyzed: human chromosome 15 (51.000 KB - 61.000 KB)

Zebrafish:

Pred Chrom.	Pred Start Base	Pred Gene ID	Pred Ext ID	Query Chrom.	Query Start Base	Query Gene ID	Query Ext ID	Group ID
Hsa15	51594673	ENSG00000166415	WDR72					0
Hsa15	52314521	ENSG00000137766	UNC13C					0
Hsa15	53260813	ENSG00000137876	C15orf15	Dre25	4255160	ENSDARG00000040439	c15orf15	8015
Hsa15	53283094	ENSG00000069974	RAB27A	Dre18	1344790	ENSDARG00000040532	zgc:114135	7987
Hsa15	53398741	ENSG00000069943	PIGB	Dre18	1320599	ENSDARG00000022744	zgc:63658	7758
Hsa15	53434741	ENSG00000128845	CCPG1	DreZv7_NA2050	590	ENSDARG00000063643	CCPG1	7964
Hsa15	53625513	ENSG00000171016	PYGO1	Dre18	3059519	ENSDARG00000044963	LOC797729	7753
Hsa15	53699353	ENSG00000166450	PRTG	Dre18	3079266	ENSDARG00000069702		7709
Hsa15	53906423	ENSG00000069869	NEDD4	Dre18	612459	ENSDARG00000039778	nedd4a	7749
Hsa15	54085203	ENSG00000166466	440280					0
Hsa15	54166958	ENSG00000181827	RFXDC2					0
Hsa15	54444936	ENSG00000151575	TEX9	Dre7	28987644	ENSDARG00000010586	Q1JQ25_DANRE	7819
Hsa15	54508299	ENSG00000138587	MNS1	Dre7	30346964	ENSDARG00000060169	zgc:65845	7945
Hsa15	54709671	ENSG00000137871	SUHW4					0
Hsa15	54925830	ENSG00000140238	650875					0
Hsa15	54998125	ENSG00000140262	TCF12	Dre7	49137965	ENSDARG00000004714	zgc:85956	8011
Hsa15	55455997	ENSG00000128849	CGNL1					0
Hsa15	55671406	ENSG00000137878	GRINL1A					0
Hsa15	56032920	ENSG00000128918	ALDH1A2	Dre7	33219016	ENSDARG00000053493	aldh1a2	7742
Hsa15	56217771	ENSG00000103569	AQP9	Dre7	33197770	ENSDARG00000053480	LOC570191	7835
Hsa15	56511467	ENSG00000166035	LIPC	Dre7	33180017	ENSDARG00000053476	lipc	7739
Hsa15	56511467	ENSG00000166035	LIPC	Dre25	28204278	ENSDARG00000070418		7739
Hsa15	56675802	ENSG00000137845	ADAM10	Dre7	33094512	ENSDARG00000053468	zgc:64203	7747
Hsa15	56675802	ENSG00000137845	ADAM10	Dre25	28209889	ENSDARG00000015502	zgc:64203	7747
Hsa15	56675802	ENSG00000137845	ADAM10	Dre22	13166833	ENSDARG00000057590	Q1LV82_DANRE	7747
Hsa15	56765902	ENSG00000182625	NULL					0
Hsa15	56770594	ENSG00000205527	HSP90AB4P					0
Hsa15	56850806	ENSG00000128923	FAM63B	Dre7	33053240	ENSDARG00000017431	si:busm1-199m19.4	7777
Hsa15	56958537	ENSG00000137776	SLTM	Dre7	33026135	ENSDARG00000017150	sltm	7952
Hsa15	57067157	ENSG00000157450	RNF111					0
Hsa15	57184612	ENSG00000157456	CCNB2	Dre7	32955414	ENSDARG00000036180	ccnb2	7892
Hsa15	57184612	ENSG00000157456	CCNB2	Dre18	35708382	ENSDARG00000055250	si:ch211-132b12.8	7892
Hsa15	57215461	ENSG00000157483	MYO1E	Dre16	34974086	ENSDARG00000009133	zgc:64042	7836
Hsa15	57215461	ENSG00000157483	MYO1E	Dre7	32892667	ENSDARG00000036179	MYO1E	7836
Hsa15	57286314	ENSG00000171989	LDHAL6B					0
Hsa15	57517664	ENSG00000157470	FAM81A					0
Hsa15	57691415	ENSG00000140297	GCNT3	DreZv7_NA1167	34389	ENSDARG00000060471	LOC100008352	7724
Hsa15	57718358	ENSG00000140307	GTF2A2	Dre7	34233565	ENSDARG00000053685	zgc:110584	8022
Hsa15	57742356	ENSG00000140299	BNIP2	DreZv7_NA1167	3024	ENSDARG00000013397	bnip2	7772
Hsa15	57847843	ENSG00000187899	NULL					0
Hsa15	58084427	ENSG00000171956	FOXB1	Dre7	34127346	ENSDARG00000053650	foxb1.1	7915
Hsa15	58084427	ENSG00000171956	FOXB1	Dre25	27987689	ENSDARG00000045569	foxb1.2	7915
Hsa15	58084427	ENSG00000171956	FOXB1	Dre23	44466626	ENSDARG00000037475	LOC559714	7915
Hsa15	58084427	ENSG00000171956	FOXB1	Dre23	44780470	ENSDARG00000069939	LOC559714	7915
Hsa15	58426643	ENSG00000182718	ANXA2	Dre25	28116676	ENSDARG00000003216	anxa2a	7789
Hsa15	58426643	ENSG00000182718	ANXA2	Dre7	34055659	ENSDARG00000053625	anxa2b	7789
Hsa15	58576755	ENSG00000069667	RORA	Dre7	34018053	ENSDARG00000001910	rorab	7788
Hsa15	58576755	ENSG00000069667	RORA	Dre25	28176695	ENSDARG00000031768	Q1L682_DANRE	7788
Hsa15	59931884	ENSG00000129003	VPS13C					0
Hsa15	60146506	ENSG00000198535	NP_997205.1					0
Hsa15	60243294	ENSG00000205502	NP_001007596					0
Hsa15	60726802	ENSG00000171914	TLN2	Dre7	33645080	ENSDARG00000017901	tln1l	7859

Stickelback

Pred Chrom.	Pred Start Base	Pred Gene ID	Pred Ext ID	Query Chrom.	Query Start Base	Query Gene ID	Query Ext ID	Group ID
Hsa15	51594673	ENSG00000166415	WDR72					0
Hsa15	52314521	ENSG00000137766	UNC13C	GacgroupII	15803295	ENSGACG00000016527	UNC13C	8789
Hsa15	53260813	ENSG00000137876	C15orf15	GacgroupXIX	7436490	ENSGACG00000006058	C15orf15	8817
Hsa15	53283094	ENSG00000069974	RAB27A	GacgroupII	11586268	ENSGACG00000015855	RAB27A	8917
Hsa15	53398741	ENSG00000069943	PIGB	GacgroupII	11579412	ENSGACG00000015853	PIGB	8662
Hsa15	53434741	ENSG00000128845	CCPG1	GacgroupII	11572026	ENSGACG00000015850	CCPG1	8966
Hsa15	53625513	ENSG00000171016	PYGO1					0
Hsa15	53699353	ENSG00000166450	PRTG	GacgroupII	11545338	ENSGACG00000015845		8705
Hsa15	53699353	ENSG00000166450	PRTG	GacgroupXIX	7441826	ENSGACG00000006072		8705
Hsa15	53906423	ENSG00000069869	NEDD4	GacgroupII	11521136	ENSGACG00000015840	NEDD4	8625
Hsa15	54085203	ENSG00000166466	440280					0
Hsa15	54166958	ENSG00000181827	RFXDC2	GacgroupXIX	7458419	ENSGACG00000006090	RFXDC2 (1 of 2)	8827
Hsa15	54444936	ENSG00000151575	TEX9	GacgroupII	11498908	ENSGACG00000015835	TEX9	8930

Hsa15	54508299	ENSG00000138587	MNS1	GacgroupII	11495261	ENSGACG00000015833	MNS1	8612
Hsa15	54709671	ENSG00000137871	SUHW4					0
Hsa15	54925830	ENSG00000140238	650875					0
Hsa15	54998125	ENSG00000140262	TCF12	GacgroupII	11422567	ENSGACG00000015831	TCF12 (1 of 2)	8929
Hsa15	54998125	ENSG00000140262	TCF12	GacgroupXIX	7477087	ENSGACG00000006101	TCF12 (2 of 2)	8929
Hsa15	55455997	ENSG00000128849	CGNL1	GacgroupII	11394568	ENSGACG00000015830	CGNL1	8626
Hsa15	55671406	ENSG00000137878	GRINL1A	GacgroupII	11375629	ENSGACG00000015829	GRINL1B	8685
Hsa15	56032920	ENSG00000128918	ALDH1A2	GacgroupII	11330738	ENSGACG00000015825	ALDH1A2	8692
Hsa15	56217771	ENSG00000103569	AQP9	GacgroupII	11320539	ENSGACG00000015823	AQP9 (1 of 2)	8922
Hsa15	56217771	ENSG00000103569	AQP9	GacgroupXIX	7496834	ENSGACG00000006120	AQP9 (2 of 2)	8922
Hsa15	56511467	ENSG00000166035	LIPC	GacgroupII	11310523	ENSGACG00000015821	LIPC	8774
Hsa15	56675802	ENSG00000137845	ADAM10	GacgroupXIX	7504978	ENSGACG00000006135	ADAM10 (1 of 2)	8703
Hsa15	56675802	ENSG00000137845	ADAM10	GacgroupII	11297810	ENSGACG00000015819	ADAM10 (2 of 2)	8703
Hsa15	56765902	ENSG00000182625	NULL					0
Hsa15	56770594	ENSG00000205527	HSP90AB4P					0
Hsa15	56850806	ENSG00000128923	FAM63B	GacgroupII	11281679	ENSGACG00000015817	FAM63B	8616
Hsa15	56958537	ENSG00000137776	SLTM	GacgroupII	11267841	ENSGACG00000015814		8753
Hsa15	57067157	ENSG00000157450	RNF111	GacgroupII	11246365	ENSGACG00000015811	RNF111	8620
Hsa15	57184612	ENSG00000157456	CCNB2	GacgroupII	22325029	ENSGACG00000017487	CCNB2 (1 of 2)	8649
Hsa15	57184612	ENSG00000157456	CCNB2	GacgroupII	21572074	ENSGACG00000017292	CCNB2 (2 of 2)	8649
Hsa15	57215461	ENSG00000157483	MYO1E	GacgroupII	11208619	ENSGACG00000015802	MYO1E	8820
Hsa15	57215461	ENSG00000157483	MYO1E	GacgroupXX	559381	ENSGACG00000003217		8820
Hsa15	57286314	ENSG00000171989	LDHAL6B					0
Hsa15	57691415	ENSG00000140297	GCNT3	GacgroupXIX	12497301	ENSGACG00000010729	GCNT3 (1 of 2)	8630
Hsa15	57691415	ENSG00000140297	GCNT3	GacgroupXIX	12488430	ENSGACG00000010725	GCNT3 (2 of 2)	8630
Hsa15	57718358	ENSG00000140307	GTF2A2	GacgroupII	22813344	ENSGACG00000017600	GTF2A2	8859
Hsa15	57742356	ENSG00000140299	BNIP2	GacgroupXIX	12479665	ENSGACG00000010705	BNIP2	8619
Hsa15	57847843	ENSG00000187899	NULL					0
Hsa15	58084427	ENSG00000171956	FOXB1	GacgroupXIX	12450291	ENSGACG00000010700		8949
Hsa15	58084427	ENSG00000171956	FOXB1	GacgroupII	22786164	ENSGACG00000017597	FOXB1	8949
Hsa15	58084427	ENSG00000171956	FOXB1	GacgroupXIII	8007189	ENSGACG00000007973		8949
Hsa15	58426643	ENSG00000182718	ANXA2	GacgroupII	22737840	ENSGACG00000017591		8650
Hsa15	58426643	ENSG00000182718	ANXA2	GacgroupXIX	12416541	ENSGACG00000010689		8650
Hsa15	58426643	ENSG00000182718	ANXA2	GacgroupX	3092584	ENSGACG00000002726	ANXA9	8650
Hsa15	58499100	ENSG00000128915	NARG2	GacgroupXIX	12408710	ENSGACG00000010682	NARG2	8937
Hsa15	58576755	ENSG00000069667	RORA	GacgroupXIX	12393533	ENSGACG00000010672	RORA	8693
Hsa15	58576755	ENSG00000069667	RORA	GacgroupXX	13371921	ENSGACG00000012239	RORC	8693
Hsa15	59931884	ENSG00000129003	VPS13C					0
Hsa15	60146506	ENSG00000198535	NP_997205.1					0
Hsa15	60243294	ENSG00000205502	NP_001007596					0
Hsa15	60726802	ENSG00000171914	TLN2	GacgroupII	15069480	ENSGACG00000016368	TLN2 (2 of 2)	8886
Hsa15	60726802	ENSG00000171914	TLN2	GacgroupXIX	12148554	ENSGACG00000010642	TLN2 (1 of 2)	8886

Medaka

Pred Chrom.	Pred Start Base	Pred Gene ID	Pred Ext ID	Query Chrom.	Query Start Base	Query Gene ID	Query Ext ID	Group ID
Hsa15	51594673	ENSG00000166415	WDR72					0
Hsa15	52314521	ENSG00000137766	UNC13C	Ola3	26422346	ENSORLG00000012968	UNC13C	8284
Hsa15	53260813	ENSG00000137876	C15orf15	Ola6	10263491	ENSORLG00000005376	C15orf15	8049
Hsa15	53283094	ENSG00000069974	RAB27A	Ola3	20383618	ENSORLG00000008731	RAB27A	7977
Hsa15	53398741	ENSG00000069943	PIGB	Ola3	20353655	ENSORLG00000008651	PIGB	8071
Hsa15	53434741	ENSG00000128845	CCPG1	Ola3	20348159	ENSORLG00000008634	CCPG1	8250
Hsa15	53488038	ENSG00000205548		Ola6	23202457	ENSORLG00000015479	C9orf116	8236
Hsa15	53625513	ENSG00000171016	PYGO1					0
Hsa15	53699353	ENSG00000166450	PRTG	Ola3	20322845	ENSORLG00000008619	PRTG (1 of 2)	8173
Hsa15	53699353	ENSG00000166450	PRTG	Ola6	10249759	ENSORLG00000005369	PRTG (2 of 2)	8173
Hsa15	53906423	ENSG00000069869	NEDD4					0
Hsa15	54085203	ENSG00000166466	440280					0
Hsa15	54166958	ENSG00000181827	RFXDC2	Ola6	10233133	ENSORLG00000005342	RFXDC2 (1 of 2)	7993
Hsa15	54166958	ENSG00000181827	RFXDC2	Ola3	20272309	ENSORLG00000008583	RFXDC2 (2 of 2)	7993
Hsa15	54444936	ENSG00000151575	TEX9	Ola3	20258552	ENSORLG00000008566	TEX9	8131
Hsa15	54508299	ENSG00000138587	MNS1	Ola3	20253265	ENSORLG00000008553	MNS1	7986
Hsa15	54709671	ENSG00000137871	SUHW4	Ola3	20241741	ENSORLG00000008529		8233
Hsa15	54925830	ENSG00000140238	650875					0
Hsa15	54998125	ENSG00000140262	TCF12	Ola3	20170084	ENSORLG00000008508	TCF12 (1 of 2)	8231
Hsa15	54998125	ENSG00000140262	TCF12	Ola6	10212559	ENSORLG00000005330	TCF12 (2 of 2)	8231
Hsa15	54998125	ENSG00000140262	TCF12	Ola12	28895603	ENSORLG00000015062	TCF4	8231
Hsa15	55455997	ENSG00000128849	CGNL1	Ola3	20131587	ENSORLG00000008414	CGNL1 (1 of 2)	8087
Hsa15	55671406	ENSG00000137878	GRINL1A	Ola3	20105466	ENSORLG00000008354	GRINL1A	8095
Hsa15	56032920	ENSG00000128918	ALDH1A2	Ola3	20046916	ENSORLG00000008319	ALDH1A2	8053
Hsa15	56217771	ENSG00000103569	AQP9	Ola3	20035808	ENSORLG00000008259	AQP9	8062
Hsa15	56511467	ENSG00000166035	LIPC	Ola3	20025818	ENSORLG00000008243	LIPC	8070
Hsa15	56675802	ENSG00000137845	ADAM10	Ola6	10173425	ENSORLG00000005304	ADAM10 (1 of 2)	8123
Hsa15	56675802	ENSG00000137845	ADAM10	Ola3	20004025	ENSORLG00000008215	ADAM10 (2 of 2)	8123
Hsa15	56675802	ENSG00000137845	ADAM10	Ola4	32834561	ENSORLG00000017020		8123

Hsa15	56765902	ENSG00000182625	NULL					0
Hsa15	56770594	ENSG00000205527	HSP90AB4P					0
Hsa15	56850806	ENSG00000128923	FAM63B	Ola3	19983075	ENSORLG00000008169	FAM63B	8319
Hsa15	56958537	ENSG00000137776	SLTM	Ola3	19961825	ENSORLG00000008161		8324
Hsa15	57067157	ENSG00000157450	RNF111	Ola3	19935319	ENSORLG00000008128	RNF111	8291
Hsa15	57184612	ENSG00000157456	CCNB2	Ola3	4509483	ENSORLG00000000615	CCNB2_ORYLA	8185
Hsa15	57215461	ENSG00000157483	MYO1E	Ola3	19891389	ENSORLG00000008091	MYO1E	8204
Hsa15	57215461	ENSG00000157483	MYO1E	Olaultracontig188	307254	ENSORLG00000020851		8204
Hsa15	57286314	ENSG00000171989	LDHAL6B					0
Hsa15	57691415	ENSG00000140297	GCNT3					0
Hsa15	57718358	ENSG00000140307	GTF2A2	Olaultracontig49	134702	ENSORLG00000018367	GTF2A2	8309
Hsa15	57742356	ENSG00000140299	BNIP2	Ola6	12024079	ENSORLG00000007570	BNIP2	8192
Hsa15	57847843	ENSG00000187899	NULL					0
Hsa15	58084427	ENSG00000171956	FOXB1	Ola6	12060296	ENSORLG00000007576	FOXB1	8149
Hsa15	58084427	ENSG00000171956	FOXB1	Ola9	11246111	ENSORLG00000005726		8149
Hsa15	58426643	ENSG00000182718	ANXA2	Ola6	12095623	ENSORLG00000007609		8217
Hsa15	58426643	ENSG00000182718	ANXA2	Olaultracontig49	216179	ENSORLG00000018369		8217
Hsa15	58426643	ENSG00000182718	ANXA2	Ola11	20070627	ENSORLG00000010101	ANXA9	8217
Hsa15	58499100	ENSG00000128915	NARG2	Ola6	12099774	ENSORLG00000007629	NARG2	8211
Hsa15	58576755	ENSG00000069667	RORA	Ola6	12115104	ENSORLG00000007645	RORA	8259
Hsa15	59931884	ENSG00000129003	VPS13C	Ola3	4184930	ENSORLG00000000514	VPS13C	8111
Hsa15	60146506	ENSG00000198535	NP_997205.1					0
Hsa15	60243294	ENSG00000205502	NP_001007596					0
Hsa15	60726802	ENSG00000171914	TLN2	Ola3	25421704	ENSORLG00000012272	TLN2 (1 of 2)	8245
Hsa15	60726802	ENSG00000171914	TLN2	Ola6	12424053	ENSORLG00000007786	TLN2 (2 of 2)	8245

Genomic information of teleost orthologs of the human ALDH1A3 gene neighbors.								
Genomic region analyzed: human chromosome 15 (90.000 KB - 100.000 KB)								
zebrafish								
Pred Chrom.	Pred Start Base	Pred Gene ID	Pred Ext ID	Query Chrom.	Query Start Base	Query Gene ID	Query Ext ID	Group ID
Hsa15	90197950	ENSG00000176463	SLCO3A1	Dre18	22338102	ENSDARG00000018726	slco3a1	7727
Hsa15	90197950	ENSG00000176463	SLCO3A1	DreZv7_scaffold2582	85646	ENSDARG00000068393	LOC570448	7727
Hsa15	90197950	ENSG00000176463	SLCO3A1	Dre7	49513448	ENSDARG00000068296	LOC570448	7727
Hsa15	90738144	ENSG00000140557	ST8SIA2	Dre18	22463125	ENSDARG00000018788	st8sia2	7938
Hsa15	90961685	ENSG00000185442	NP_997329.1	Dre18	22507973	ENSDARG00000068709	si:ch211-196l7.3	7822
Hsa15	91244423	ENSG00000173575	CHD2	Dre18	22526310	ENSDARG00000060687	chd2	7959
Hsa15	91387651	ENSG00000182175	RGMA	Dre18	22582911	ENSDARG00000012248	rgma	7824
Hsa15	92575955	ENSG00000140563	MCTP2	Dre18	10681674	ENSDARG00000001414	si:ch211-214d15.1	7712
Hsa15	94674950	ENSG00000185551	NR2F2	Dre18	23645970	ENSDARG00000040926	nr2f2	8000
Hsa15	96304947	ENSG00000140450	ARRDC4					0
Hsa15	96800383	ENSG00000185087	NP_872368.2					0
Hsa15	97010302	ENSG00000140443	IGF1R	Dre18	13129824	ENSDARG00000027423	igf1ra	7867
Hsa15	97010302	ENSG00000140443	IGF1R	Dre7	7604579	ENSDARG00000034434	igf1rb	7867
Hsa15	97328859	ENSG00000183571	145814					0
Hsa15	97462771	ENSG00000182253	DMN	Dre18	13307442	ENSDARG00000062350	si:dkey-25f3.3	7866
Hsa15	97494052	ENSG00000103852	TTC23					0
Hsa15	97609175	ENSG00000168904	LRRC28	Dre7	7777720	ENSDARG00000057477	LRRC28	7885
Hsa15	97616372	ENSG00000205141	HSP90B2P					0
Hsa15	97923712	ENSG00000068305	MEF2A	Dre18	10776964	ENSDARG00000031756	mef2a	7834
Hsa15	97923712	ENSG00000068305	MEF2A	Dre7	7836687	ENSDARG00000057527		7834
Hsa15	98073429	ENSG00000183060	LYSMD4	Dre19	42828669	ENSDARG00000039058	zgc:77861	7894
Hsa15	98073429	ENSG00000183060	LYSMD4	Dre18	22940853	ENSDARG00000068702	LYSMD4 (1 of 2)	7894
Hsa15	98758124	ENSG00000154227	LASS3					0
Hsa15	98960337	ENSG00000183475	ASB7	Dre7	7985151	ENSDARG00000006494	asb7	7843
Hsa15	99237580	ENSG00000184254	ALDH1A3	Dre7	8113850	ENSDARG00000038135	aldh1a3	7908
Hsa15	99277073	ENSG00000154237	LRRK1	DreZv7_NA891	40243	ENSDARG00000007398	LRRK1	7923
Hsa15	99533456	ENSG00000131873	CHSY1					0
Hsa15	99628737	ENSG00000131871	SELS_HUMAN	Dre7	3580258	ENSDARG00000070097	zgc:136970	7988
Hsa15	99639240	ENSG00000131876	SNRPA1	Dre7	3596477	ENSDARG00000024651	SNRPA1	7764
Hsa15	99661657	ENSG00000140479	PCSK6					0
Hsa15	99999580	ENSG00000184277	TM2D3					0
stickleback								
Pred Chrom.	Pred Start Base	Pred Gene ID	Pred Ext ID	Query Chrom.	Query Start Base	Query Gene ID	Query Ext ID	Group ID
Hsa15	90197950	ENSG00000176463	SLCO3A1	GacgroupXIX	12610128	ENSGACG00000010749		8914
Hsa15	90197950	ENSG00000176463	SLCO3A1	GacgroupII	5139657	ENSGACG00000014835		8914
Hsa15	90738144	ENSG00000140557	ST8SIA2	GacgroupXIX	12597193	ENSGACG00000010746	ST8SIA2	8706
Hsa15	90961685	ENSG00000185442	NP_997329.1	GacgroupXIX	17516124	ENSGACG00000013258	NP_997329.2 (1 of 2)	8642
Hsa15	91244423	ENSG00000173575	CHD2	GacgroupXIX	17490251	ENSGACG00000013246		8660
Hsa15	91387651	ENSG00000182175	RGMA	GacgroupXIX	17482812	ENSGACG00000013241	RGMA	8815
Hsa15	91387651	ENSG00000182175	RGMA	GacgroupVIII	16469522	ENSGACG00000012876		8815
Hsa15	92575955	ENSG00000140563	MCTP2	GacgroupII	5193703	ENSGACG00000014848	MCTP2 (1 of 2)	8970
Hsa15	93199707	ENSG00000176070	NULL					0
Hsa15	94674950	ENSG00000185551	NR2F2	GacgroupXIX	17251262	ENSGACG00000013235		8846
Hsa15	94674950	ENSG00000185551	NR2F2	GacgroupII	5172182	ENSGACG00000014846	NR2F2	8846
Hsa15	96304947	ENSG00000140450	ARRDC4					0
Hsa15	96800383	ENSG00000185087	NP_872368.2					0
Hsa15	97010302	ENSG00000140443	IGF1R	GacgroupXIX	16907878	ENSGACG00000013170		8721
Hsa15	97010302	ENSG00000140443	IGF1R	GacgroupII	4540717	ENSGACG00000014729		8721
Hsa15	97328859	ENSG00000183571	145814					0
Hsa15	97494052	ENSG00000103852	TTC23					0
Hsa15	97609175	ENSG00000168904	LRRC28	GacgroupII	4531459	ENSGACG00000014725	LRRC28	8951
Hsa15	97616372	ENSG00000205141	HSP90B2P					0
Hsa15	97923712	ENSG00000068305	MEF2A	GacgroupXIX	17024766	ENSGACG00000013182	MEF2A (1 of 2)	8950
Hsa15	98073429	ENSG00000183060	LYSMD4	GacgroupXIX	17415854	ENSGACG00000013238	LYSMD4	8901
Hsa15	98147884	ENSG00000182397	C15orf51					0
Hsa15	98331993	ENSG00000140470	ADAMTS17	Gacscaffold_98	212590	ENSGACG00000012531	ADAMTS17	8777
Hsa15	98758124	ENSG00000154227	LASS3					0
Hsa15	98960337	ENSG00000183475	ASB7	GacgroupII	220883	ENSGACG00000013982	ASB7	8811
Hsa15	99237580	ENSG00000184254	ALDH1A3	GacgroupII	251899	ENSGACG00000013986	ALDH1A3	8645
Hsa15	99277073	ENSG00000154237	LRRK1	GacgroupII	288668	ENSGACG00000014066	LRRK1	8737
Hsa15	99533456	ENSG00000131873	CHSY1	GacgroupII	337719	ENSGACG00000014084	CHSY1	8697
Hsa15	99628737	ENSG00000131871	SELS_HUMAN	GacgroupII	372697	ENSGACG00000014089	SELS_HUMAN	8730
Hsa15	99639240	ENSG00000131876	SNRPA1	GacgroupII	377233	ENSGACG00000014092	SNRPA1	8868
Hsa15	99661657	ENSG00000140479	PCSK6	GacgroupII	3557486	ENSGACG00000014499	PCSK6	8761
Hsa15	99999580	ENSG00000184277	TM2D3	GacgroupII	4231521	ENSGACG00000014624	TM2D3	8802
medaka								
Pred Chrom.	Pred Start Base	Pred Gene ID	Pred Ext ID	Query Chrom.	Query Start Base	Query Gene ID	Query Ext ID	Group ID
Hsa15	90197950	ENSG00000176463	SLCO3A1	Ola6	13137486	ENSORLG00000008355	Q76KD1_ORYLA	8167
Hsa15	90197950	ENSG00000176463	SLCO3A1	Ola3	32133573	ENSORLG00000014898	SLCO3A1 (1 of 2)	8167
Hsa15	90738144	ENSG00000140557	ST8SIA2	Ola6	13209297	ENSORLG00000008368	ST8SIA2	8010
Hsa15	91055981	ENSG00000157576	XR_018454.1					0
Hsa15	91244423	ENSG00000173575	CHD2	Ola6	13238854	ENSORLG00000008403		8073

Hsa15	91387651	ENSG00000182175	RGMA	Ola6	13253996	ENSORLG00000008413	RGMA	8008
Hsa15	91387651	ENSG00000182175	RGMA	Ola4	8014819	ENSORLG00000004588		8008
Hsa15	92575955	ENSG00000140563	MCTP2	Ola6	13751702	ENSORLG00000008458	MCTP2 (2 of 2)	8296
Hsa15	93199707	ENSG00000176070	NULL					0
Hsa15	94674950	ENSG00000185551	NR2F2	Ola6	13566630	ENSORLG00000008429	NR2F2	8032
Hsa15	96304947	ENSG00000140450	ARRDC4					0
Hsa15	97010302	ENSG00000140443	IGF1R	Ola6	13898421	ENSORLG00000008532		8141
Hsa15	97010302	ENSG00000140443	IGF1R	Ola3	31746669	ENSORLG00000014732		8141
Hsa15	97328859	ENSG00000183571	145814					0
Hsa15	97462771	ENSG00000182253	DMN					0
Hsa15	97609175	ENSG00000168904	LRRC28	Ola3	31800920	ENSORLG00000014753	LRRC28	8326
Hsa15	97616372	ENSG00000205141	HSP90B2P					0
Hsa15	97923712	ENSG00000068305	MEF2A	Ola6	13788320	ENSORLG00000008489	MEF2A (1 of 2)	8238
Hsa15	97923712	ENSG00000068305	MEF2A	Ola3	32270339	ENSORLG00000014967	MEF2A (2 of 2)	8238
Hsa15	98073429	ENSG00000183060	LYSMD4					0
Hsa15	98147884	ENSG00000182397	C15orf51					0
Hsa15	98331993	ENSG00000140470	ADAMTS17					0
Hsa15	98758124	ENSG00000154227	LASS3					0
Hsa15	98926957	ENSG00000140471	LINS1	Olascaffold572	22195	ENSORLG00000019457	LINS1	8283
Hsa15	98960337	ENSG00000183475	ASB7	Olascaffold572	35277	ENSORLG00000019464	ASB7	8214
Hsa15	99237580	ENSG00000184254	ALDH1A3					0
Hsa15	99277073	ENSG00000154237	LRRK1	Olascaffold572	61571	ENSORLG00000019481		manual annot
Hsa15	99533456	ENSG00000131873	CHSY1	Olascaffold572	113422	ENSORLG00000019490		8306
Hsa15	99628737	ENSG00000131871	SELS_HUMAN	Olascaffold572	119261	ENSORLG00000019500		8276
Hsa15	99639240	ENSG00000131876	SNRPA1	Olascaffold572	130099	ENSORLG00000019509	SNRPA1	8208
Hsa15	99661657	ENSG00000140479	PCSK6	Olascaffold2005	4099	ENSORLG00000020313	PCSK6	8027

Genomic information of teleost orthologs of the human ALDH1A3-ogm gene neighbors.

Genomic regiona analyzed: human chromosome 5 (90000 KB - 100000 KB)

zebrafish

Pred Chrom.	Pred Start Base	Pred Gene ID	Pred Ext ID	Query Chrom.	Query Start Base	Query Gene ID	Query Ext ID	Group ID
Hsa5	90700299	ENSG00000113369	ARRDC3	Dre5	47557197	ENSDARG00000052690	zgc:136956	10229
Hsa5	90700299	ENSG00000113369	ARRDC3	Dre10	41349724	ENSDARG00000036028	zgc:92034	10229
Hsa5	90700299	ENSG00000113369	ARRDC3	Dre8	22670218	ENSDARG00000039443	zgc:110353	10229
Hsa5	90700299	ENSG00000113369	ARRDC3	Dre5	33943005	ENSDARG00000032444	A2BIP7_DANRE	10229
Hsa5	90700299	ENSG00000113369	ARRDC3	Dre5	33944603	ENSDARG00000053159	zgc:110626	10229
Hsa5	90700299	ENSG00000113369	ARRDC3	Dre5	47524283	ENSDARG00000052693	XR_029231.1	10229
Hsa5	92944799	ENSG00000175745	NR2F1	Dre5	46801603	ENSDARG00000052695	nr2f1	10245
Hsa5	92944799	ENSG00000175745	NR2F1	Dre10	41312859	ENSDARG00000017168	nr2f1l	10245
Hsa5	92944799	ENSG00000175745	NR2F1	Dre16	43143836	ENSDARG00000033172	nr2f5	10245
Hsa5	92979536	ENSG00000113391	C5orf21	Dre5	46600628	ENSDARG00000052697	zgc:64188	10209
Hsa5	92979536	ENSG00000113391	C5orf21	Dre1	22977232	ENSDARG00000058982	LOC131909	10209
Hsa5	93979808	ENSG00000133302	ANKRD32					0
Hsa5	94068601	ENSG00000175471	MCTP1					0
Hsa5	94754247	ENSG00000153347	FAM81B	Dre5	45665124	ENSDARG00000035193	LOC797755	10347
Hsa5	94825355	ENSG00000198677	KIAA0372					0
Hsa5	94916608	ENSG00000164291	ARSK	Dre5	45566838	ENSDARG00000059714	zgc:153019	10391
Hsa5	95008239	ENSG00000175449	RFESD	Dre8	31077121	ENSDARG00000042159	LOC559859	10118
Hsa5	95008239	ENSG00000175449	RFESD	Dre10	42001294	ENSDARG00000058725	zgc:112118	10118
Hsa5	95008239	ENSG00000175449	RFESD	Dre22	21384235	ENSDARG00000071333	si:ch211-212d10.2	10118
Hsa5	95175309	ENSG00000173221	GLRX	Dre21	3911176	ENSDARG00000028106	glrx	10470
Hsa5	95246558	ENSG00000118985	ELL2	Dre21	3920295	ENSDARG00000006251	ell2	10159
Hsa5	95246558	ENSG00000118985	ELL2	Dre20	45159823	ENSDARG00000069258	ELL (2 of 2)	10159
Hsa5	95751875	ENSG00000175426	PCSK1	Dre21	3971002	ENSDARG00000002600	LOC100005716	10185
Hsa5	96023533	ENSG00000153113	CAST					0
Hsa5	96122277	ENSG00000164307	ARTS1_HUMAN	Dre5	19301494	ENSDARG00000021859	A2BGR0_DANRE	10288
Hsa5	96237474	ENSG00000164308	NP_071745.1					0
Hsa5	96296924	ENSG00000113441	LNPEP	Dre10	1324483	ENSDARG00000013024	ARTS1_HUMAN (2 of 2)	10310
Hsa5	96296924	ENSG00000113441	LNPEP	DreZv7_scaffold2562	131332	ENSDARG00000014403	zgc:66103	10310
Hsa5	96453330	ENSG00000145721	LIX1	Dre5	57542588	ENSDARG00000052154	A2BF96_DANRE	10261
Hsa5	96522327	ENSG00000058729	RIOK2	Dre5	38676526	ENSDARG00000035264	riok2	10487
Hsa5	97574862	ENSG00000174144	NULL					0
Hsa5	98132900	ENSG00000174136	RGMB	DreZv7_scaffold2562	68524	ENSDARG00000068225	rgmb	10522
Hsa5	98132900	ENSG00000174136	RGMB	Dre5	42710202	ENSDARG00000002987	rgmb	10522
Hsa5	98132900	ENSG00000174136	RGMB	Dre22	19632702	ENSDARG00000034013	zgc:66326	10522
Hsa5	98218819	ENSG00000153922	CHD1	Dre10	40065060	ENSDARG00000014878	LOC563236	10493
Hsa5	99898908	ENSG00000174132	TMEM157					0

stickelback

Pred Chrom.	Pred Start Base	Pred Gene ID	Pred Ext ID	Query Chromosome	Query Start Base	Query Gene ID	Query Ext ID	Group ID
Hsa5	90700299	ENSG00000113369	ARRDC3	GacgroupXIII	9952936	ENSGACG00000010182		11844
Hsa5	90700299	ENSG00000113369	ARRDC3	GacgroupXIII	9949000	ENSGACG00000010177		11844
Hsa5	90700299	ENSG00000113369	ARRDC3	GacgroupXIII	10704836	ENSGACG00000010375		11844
Hsa5	90700299	ENSG00000113369	ARRDC3	GacgroupXIII	11388153	ENSGACG00000010472	ARRDC3	11402
Hsa5	90700299	ENSG00000113369	ARRDC3	GacgroupXIV	330064	ENSGACG00000015552		11402
Hsa5	92944799	ENSG00000175745	NR2F1	GacgroupXIII	10982431	ENSGACG00000010385	NR2F1	11396
Hsa5	92979536	ENSG00000113391	C5orf21	GacgroupXIII	11046014	ENSGACG00000010397	C5orf21	11527
Hsa5	93515468	ENSG00000198112	Q6ZNN2_HUMAN					0
Hsa5	93774435	ENSG00000175483	O94914_HUMAN					0
Hsa5	93880683	ENSG00000185261	NP_775936.1					0
Hsa5	93979808	ENSG00000133302	ANKRD32					0
Hsa5	94068601	ENSG00000175471	MCTP1	GacgroupXIII	11261796	ENSGACG00000010419	MCTP1 (1 of 2)	11487
Hsa5	94068601	ENSG00000175471	MCTP1	GacgroupXIV	336867	ENSGACG00000015555	MCTP1 (2 of 2)	11487
Hsa5	94068601	ENSG00000175471	MCTP1	GacgroupXIX	17062801	ENSGACG00000013219	MCTP2 (2 of 2)	11487
Hsa5	94825355	ENSG00000198677	KIAA0372	GacgroupXIII	11372087	ENSGACG00000010433	KIAA0372	11640
Hsa5	94916608	ENSG00000164291	ARSK	GacgroupXIII	11384290	ENSGACG00000010462	ARSK	11804
Hsa5	94981538	ENSG00000178015	GPR150					0
Hsa5	95008239	ENSG00000175449	RFESD	GacgroupXIII	8172818	ENSGACG00000008080	RFESD (1 of 2)	11656
Hsa5	95008239	ENSG00000175449	RFESD	GacgroupXVII	8481021	ENSGACG00000008894	RFESD (2 of 2)	11656
Hsa5	95008239	ENSG00000175449	RFESD	GacgroupVIII	16813303	ENSGACG00000013034		11656
Hsa5	95092635	ENSG00000164292	RHOBTB3	GacgroupXVII	8485457	ENSGACG00000008899	RHOBTB3	11571
Hsa5	95175309	ENSG00000173221	GLRX	GacgroupXVII	8504518	ENSGACG00000008905	GLRX	11821
Hsa5	95246558	ENSG00000118985	ELL2	GacgroupXVII	8508767	ENSGACG00000008906	ELL2	11632
Hsa5	95751875	ENSG00000175426	PCSK1	GacgroupXVII	8529588	ENSGACG00000008926	PCSK1	11661
Hsa5	96023533	ENSG00000153113	CAST					0
Hsa5	96122277	ENSG00000164307	ARTS1_HUMAN					0
Hsa5	96237474	ENSG00000164308	NP_071745.1	GacgroupXIII	9555534	ENSGACG00000009777	NP_071745.1	11726
Hsa5	96237474	ENSG00000164308	NP_071745.1	GacgroupXIII	16842384	ENSGACG00000013677	ARTS1_HUMAN	11726
Hsa5	96296924	ENSG00000113441	LNPEP	GacgroupXIII	9567423	ENSGACG00000009799	LNPEP	11528
Hsa5	96453330	ENSG00000145721	LIX1	GacgroupXIII	9502167	ENSGACG00000009749	LIX1	11577
Hsa5	96522327	ENSG00000058729	RIOK2	GacgroupXIII	9506321	ENSGACG00000009754	RIOK2	11471
Hsa5	97574862	ENSG00000174144	NULL					0
Hsa5	98132900	ENSG00000174136	RGMB	GacgroupXIII	9546174	ENSGACG00000009766	RGMB	11863
Hsa5	98218819	ENSG00000153922	CHD1	GacgroupXIV	14982279	ENSGACG00000018644		11696
Hsa5	99898908	ENSG00000174132	TMEM157					0

medaka								
Pred Chrom.	Pred Start Base	Pred Gene ID	Pred Ext ID	Query Chromosome	Query Start Base	Query Gene ID	Query Ext ID	Group ID
Hsa5	90700299	ENSG00000113369	ARRDC3	Ola9	15904318	ENSORLG00000010381	ARRDC3	10670
Hsa5	90700299	ENSG00000113369	ARRDC3	Ola12	1114417	ENSORLG00000001039		10670
Hsa5	90700299	ENSG00000113369	ARRDC3	Ola9	13908136	ENSORLG00000009832		10670
Hsa5	90700299	ENSG00000113369	ARRDC3	Ola9	13867062	ENSORLG00000009825		10670
Hsa5	92944799	ENSG00000175745	NR2F1	Ola16	26934023	ENSORLG00000016315		11047
Hsa5	92944799	ENSG00000175745	NR2F1	Ola9	15383514	ENSORLG00000010191	NR2F1	11047
Hsa5	92979536	ENSG00000113391	C5orf21	Ola9	15412328	ENSORLG00000010210	C5orf21	10580
Hsa5	93515468	ENSG00000198112	Q6ZNN2_HUMAN					0
Hsa5	93774435	ENSG00000175483	O94914_HUMAN	Ola9	15631554	ENSORLG00000010225		10775
Hsa5	93880683	ENSG00000185261	NP_775936.1	Ola9	15667648	ENSORLG00000010234	C5orf36	10757
Hsa5	93979808	ENSG00000133302	ANKRD32					0
Hsa5	94068601	ENSG00000175471	MCTP1	Ola9	15718278	ENSORLG00000010281	MCTP1 (1 of 2)	10799
Hsa5	94068601	ENSG00000175471	MCTP1	Ola12	1132486	ENSORLG00000001062	MCTP1 (2 of 2)	10799
Hsa5	94068601	ENSG00000175471	MCTP1	Ola3	32218831	ENSORLG00000014928	MCTP2 (1 of 2)	10799
Hsa5	94825355	ENSG00000198677	KIAA0372	Ola9	15882295	ENSORLG00000010323	KIAA0372	11085
Hsa5	94916608	ENSG00000164291	ARSK	Ola9	15899709	ENSORLG00000010351	ARSK	10621
Hsa5	94981538	ENSG00000178015	GPR150					0
Hsa5	95008239	ENSG00000175449	RFESD	Ola9	11417182	ENSORLG00000005873	RFESD (1 of 2)	10984
Hsa5	95008239	ENSG00000175449	RFESD	Ola5	16301820	ENSORLG00000008675	RFESD (2 of 2)	10984
Hsa5	95092635	ENSG00000164292	RHOBTB3	Ola5	16274304	ENSORLG00000008666	RHOBTB3	10696
Hsa5	95175309	ENSG00000173221	GLRX	Ola5	16266212	ENSORLG00000008650	GLRX	10689
Hsa5	95246558	ENSG00000118985	ELL2	Ola5	16247398	ENSORLG00000008642	ELL2	10716
Hsa5	95246558	ENSG00000118985	ELL2	Ola6	13011124	ENSORLG00000008312	ELL3 (2 of 2)	10716
Hsa5	95246558	ENSG00000118985	ELL2	Ola22	8137437	ENSORLG00000013871	ELL (2 of 2)	10716
Hsa5	95751875	ENSG00000175426	PCSK1	Ola5	16221349	ENSORLG00000008605	PCSK1	10598
Hsa5	96023533	ENSG00000153113	CAST					0
Hsa5	96122277	ENSG00000164307	ARTS1_HUMAN	Ola9	3000703	ENSORLG00000001321	ARTS-1	10995
Hsa5	96237474	ENSG00000164308	NP_071745.1	Ola9	13343553	ENSORLG00000008714	LRAP	10937
Hsa5	96296924	ENSG00000113441	LNPEP	Ola9	13356719	ENSORLG00000008772	LNPEP	10874
Hsa5	96453330	ENSG00000145721	LIX1	Ola9	13279508	ENSORLG00000008629	LIX1	10667
Hsa5	96522327	ENSG00000058729	RIOK2	Ola9	13285382	ENSORLG00000008661	RIOK2	10833
Hsa5	97574862	ENSG00000174144	NULL					0
Hsa5	98132900	ENSG00000174136	RGMB	Ola9	13331899	ENSORLG00000008678	RGMB	10732
Hsa5	98218819	ENSG00000153922	CHD1	Ola12	27099078	ENSORLG00000014570		10998
Hsa5	99898908	ENSG00000174132	TMEM157					0
